# Cohesin prevents local mixing of condensed euchromatic domains in living human cells

**DOI:** 10.1038/s41588-026-02736-2

**Published:** 2026-09-08

**Authors:** Masa A. Shimazoe, Shiori Iida, Katsuhiko Minami, Koichi Higashi, Sachiko Tamura, Yoshiaki Kobayashi, Shin Fujishiro, Le Xiong, Kako Nakazato, S. S. Ashwin, Tomoko Nishiyama, Yu Nagata, Masato T. Kanemaki, Akane Kawaguchi, Yasuyuki Ohkawa, Lothar Schermelleh, Atsushi Toyoda, Liangqi Xie, Ken Kurokawa, Hiroshi Ochiai, Masaki Sasai, Kazuhiro Maeshima

**Affiliations:** 1 Genome Dynamics Laboratory, National Institute of Genetics, ROIS, Mishima, Japan; 2 Graduate Institute for Advanced Studies, Graduate University for Advanced Studies (SOKENDAI), Mishima, Japan; 3 Genome Evolution Laboratory, National Institute of Genetics, Mishima, Japan; 4 Division of Gene Expression Dynamics, Medical Institute of Bioregulation, Kyushu University, Fukuoka, Japan; 5 Fukui Institute for Fundamental Chemistry, Kyoto University, Kyoto, Japan; 6 Department of Cancer Biology, Lerner Research Institute, Cleveland Clinic, Cleveland, OH USA; 7 Department of Physics, Gandhi Institute of Technology and Management (GITAM) University, Bengaluru, India; 8 Division of Biological Sciences, Graduate School of Science, Kyoto University, Kyoto, Japan; 9 Molecular Cell Engineering Laboratory, National Institute of Genetics, ROIS, Mishima, Japan; 10 Department of Biological Science, Graduate School of Science, The University of Tokyo, Tokyo, Japan; 11 Laboratory for Molecular Life History, National Institute of Genetics, ROIS, Mishima, Japan; 12 Division of Transcriptomics, Medical Institute of Bioregulation, Kyushu University, Fukuoka, Japan; 13 Department of Biochemistry, University of Oxford, Oxford, UK; 14 Comparative Genomics Laboratory, National Institute of Genetics, ROIS, Mishima, Japan; 15 Department of Complex Systems Science, Nagoya University, Nagoya, Japan; 16 Present Address: Department of Cell and Tissue Dynamics, Max Planck Institute for Molecular Biomedicine, Münster, Germany; 17 Present Address: Department of Molecular and Cellular Biology, Harvard University, Cambridge, MA USA

**Keywords:** Biophysics, Cell biology

## Abstract

The human genome is folded into chromatin loops by the cohesin complex, forming functional chromatin domains that underlie transcription and DNA replication/repair. However, how cohesin organizes these domains in living cells, especially in active euchromatin, remains elusive. Here, to address this question, we combined single-nucleosome imaging/tracking and super-resolution three-dimensional structured illumination microscopy with euchromatin-specific labeling of histone variant H3.3. Using this nanoscopic approach, we revealed that euchromatin forms condensed domains that are constrained by cohesin-mediated loops. This organization refines the classical view of euchromatin as largely open, in line with emerging evidence. Transcription machinery appears to be located near the condensed domain surfaces/borders. Cohesin loss increased nucleosome-level fluidity within these domains without altering their overall compaction, leading to local mixing of domains and compromising transcriptional insulation. These findings suggest a physical role of cohesin in maintaining the integrity of condensed euchromatic domains and ensuring proper higher-order regulation of gene expression.

## Abstract (web summary)

Single-nucleosome imaging/tracking and super-resolution three-dimensional structured illumination microscopy within euchromatic regions show that cohesin-mediated loops constrain condensed euchromatic domains. Cohesin loss increases their fluidity without affecting their overall compaction, leading to local domain mixing and compromised transcriptional insulation.

## Main

Two meters of genomic DNA in human cells are wrapped around core histones to form nucleosomes^1,2^. Various imaging studies have long suggested that a string of nucleosomes is folded irregularly into chromatin domains in the interphase nucleus^3–12^. Genomics analyses, such as Hi–C^13^, have also identified similar domains or topologically associating domains (TADs) with distinct epigenetic marks^14–17^. The classic view of genomic chromatin describes that transcriptionally inactive heterochromatin forms condensed domains, whereas active euchromatin forms open ones, thereby supposedly making euchromatin accessible to transcription machinery^18,19^. However, the expression profile of euchromatic genes varies greatly from cell to cell^20,21^, implying that transcriptional regulation may be governed by higher-order chromatin organization rather than a simple ‘open-or-closed’ binary model. Consistent with this, recent evidence challenges this established view, suggesting that euchromatin is also organized into largely condensed domains, which may contribute to higher-order gene regulation^7,8,10,22–24^.

While interactions between genomic sequences within and between domains are well-recognized as crucial for transcriptional regulation^15,24–26^, DNA replication^27^ and repair^28^, the physical properties of chromatin domains have been comparatively overlooked. Yet, they are equally important. Chromatin dynamics and their physical properties directly influence genomic accessibility^29,30^ and can modulate the efficiency and probability of these biochemical reactions. Therefore, elucidating how the physical properties of chromatin domains are regulated is crucial.

A key factor influencing both chromatin organization and dynamics is the cohesin complex, composed of four subunits (SMC1, SMC3, RAD21 and STAG1/STAG2)^31–35^. Cohesin has two main roles in organizing genomic chromatin. One is to hold replicated sister chromatids together for their proper segregation during cell division (‘cohesion’) and the other is to form chromatin loops within individual chromosomes to create the domains described above^36–38^. Cohesin may also regulate the physical properties of chromatin in living cells. Indeed, pioneering work using the *lacO*/EGFP-LacI system in budding yeast demonstrated that cohesin loss increases chromatin motion, presumably through the loss of sister chromatid cohesion^39,40^. Subsequent studies revealed that, in higher eukaryotic cells, cohesin depletion also increases chromatin motion^5,21,41–43^. However, it is still unclear how cohesin constrains chromatin, whether primarily through sister chromatid cohesion or loop formation, and which chromatin domains, euchromatin or heterochromatin, are most constrained. Furthermore, how cohesin-mediated constraints affect the physical properties of chromatin domains and their genome functions such as transcriptional regulation remains poorly understood.

To address these questions, we developed a combined approach of single-nucleosome imaging^5,29,44,45^ and three-dimensional structured illumination microscopy (3D-SIM)^7,46–48^, incorporating a specific labeling strategy with the histone variant H3.3, which is enriched in active chromatin regions^49–51^. Using these advanced nanoscopic imaging techniques, we uncovered condensed euchromatic domains in living human cells, providing further critical evidence that challenges the established view of euchromatin as largely open. We also demonstrated that cohesin governs the fluidity of these euchromatic domains without affecting their overall compaction. Together with genomic data and computational modeling, these findings highlight a previously underappreciated role of cohesin in preventing local mixing of condensed euchromatin domains, thereby contributing to effective transcriptional insulation^52,53^ among them.

## Results

### Acute cohesin loss increases local nucleosome motion

**Fig. 1:**
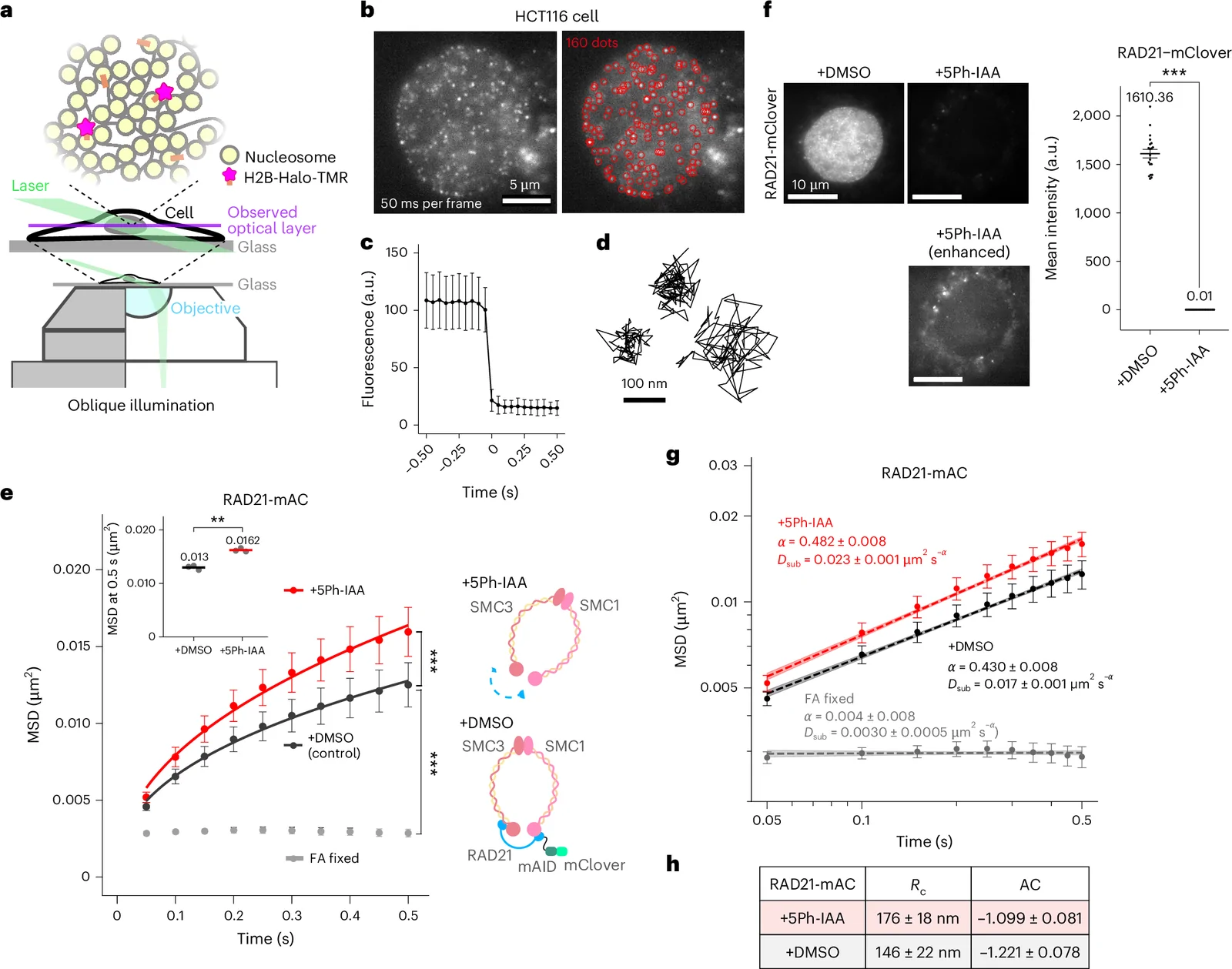
Single-nucleosome imaging revealed increased nucleosome motion upon cohesin depletion. **a**, Top: a small fraction of H2B-Halo was fluorescently labeled with the TMR-HaloTag ligand (magenta star) and used to track nucleosome movements at super-resolution. Bottom: oblique illumination microscopy. The illumination laser (green) excites fluorescent molecules within a thin optical layer (purple) of the nucleus, reducing background noise. **b**, Single-nucleosome (H2B-Halo-TMR) image of a living HCT116 nucleus after background subtraction. Red circles highlight the 160 tracked dots. Dots observed for more than three frames were tracked. **c**, Single-step photobleaching of nucleosome (H2B-Halo-TMR) dots. The vertical axis shows fluorescence intensity of individual TMR dots (±s.d. among nucleosomes); the horizontal axis is the tracking time series (*n* = 30 nucleosomes). **d**, Representative trajectories of three tracked single nucleosomes. **e**, MSD plots (±s.d. among cells) of H2B-Halo in HCT116 RAD21-mAC cells under the indicated conditions—DMSO (black, *n* = 30 cells), 5Ph-IAA (ΔRAD21, red, *n* = 30 cells) and FA fixed (gray, *n* = 15 cells). ***P = 8.47 × 10^−10^ (DMSO versus 5Ph-IAA) and *P* = 5.8 × 10^−12^ (DMSO versus FA fixed) by two-sided Kolmogorov–Smirnov test. Inner panel shows replicate-aware quantification of MSD at 0.5 s. Each dot represents an independent biological replicate (*n* = 3). Bars indicate the mean. **P < 0.01 (*P* = 1.6 × 10^−3^) by a two-sided paired *t* test. Statistical details are provided in Supplementary Table 1. **f**, Verification of RAD21 depletion by mClover signal in live nuclei. Right: individual dots represent cells; horizontal lines and error bars indicate the mean ± s.e.m. across cells. Mean intensity values—1.6 × 10^3^ for DMSO (*n* = 20 cells) and 0.010 for +5Ph-IAA (ΔRAD21, *n* = 20 cells). ***P < 0.0001 (*P* = 1.1 × 10^−8^) by two-sided Wilcoxon rank-sum test. **g**, log–log plots of MSD from **e**. The plots were fitted linearly (dashed lines), and the diffusion exponent *α* and the coefficient of subdiffusion *D*_sub_ were estimated from the slopes and intercepts of the fitted lines. Please note the nonoverlapping 95% confidence intervals of the linear fits (shades) across conditions. **h**, *R*_c_ and AC under the indicated conditions (mean ± s.d. among cells)—DMSO (*n* = 30 cells), 5Ph-IAA (ΔRAD21, *n* = 30 cells), calculated as in Extended Data Fig. 1g–i. For *R*_c_, *P* = 4.32 × 10^−8^ by two-sided Kolmogorov–Smirnov test. For AC, *P* = 3.68 × 10^−7^ by Holm-adjusted Welch’s *t* test. Replicate-aware statistical details for *R*_c_, AC and other metrics for all figures are provided in Supplementary Table 1. DMSO, dimethyl sulfoxide; *R*_c_, radius of constraint; AC, asymmetry coefficient. Source data

**Extended Data Fig. 1:**
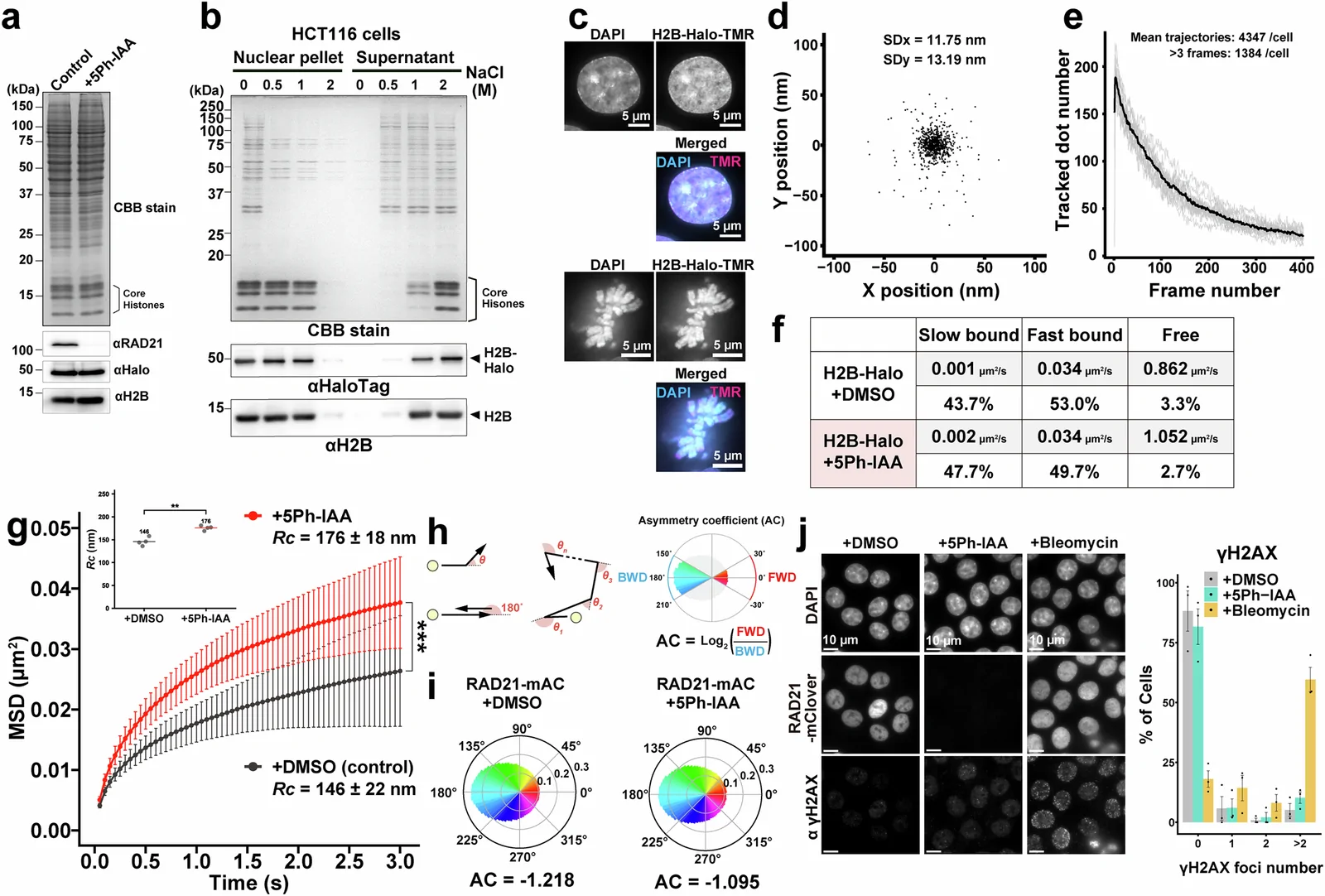
Single-nucleosome imaging using HCT116 cells with RAD21-AID2. **a**, Immunoblotting of HCT116 cells expressing H2B-HaloTag (H2B-Halo) and RAD21-mAID-mClover (mAC), and verification of RAD21 depletion by AID2. The experiment was independently repeated at least three times with similar results. **b**, Stepwise salt washing of nuclei expressing H2B-Halo. Nuclear pellets and supernatants were analyzed by SDS–PAGE, Coomassie staining, and immunoblotting for H2B and HaloTag. Endogenous H2B and H2B-Halo began to dissociate from chromatin at 1 M NaCl and were detected in the supernatant, indicating similar nucleosome stability. The image shown was obtained from a single experiment. **c**, HCT116 cells expressing H2B-Halo labeled with DAPI and excess TMR-HaloTag ligand. Top, interphase cell. Bottom, mitotic cell. **d**, Position-determination accuracy of H2B-Halo-TMR. n = 10 nucleosomes in an FA-fixed cell. **e**, Number of detected dots per frame (n = 15 cells). Dot counts decrease because of photobleaching, while new dots occasionally appear by entering the focal plane. Please note that the average number of trajectories does not correspond to individual nucleosomes, as some trajectories may be fragmented. **f**, Spot-on estimates of diffusion coefficients and fractions for H2B-Halo-TMR imaged at 10 ms/frame. Freely diffusing fractions were negligible. **g**, MSD plots (mean ± s.d. among cells) for DMSO (black, n = 30 cells) and 5Ph-IAA (ΔRAD21, red, n = 30 cells). ***P = 4.32 × 10^−8^ by two-sided Kolmogorov–Smirnov test. *Rc*: DMSO, 146 ± 22 nm; 5Ph-IAA, 176 ± 18 nm. Inset: replicate-aware *Rc* quantification; dots represent independent biological replicates (n = 4), bars indicate mean, paired two-sided *t*-test, **P < 0.01. **h**, Schematic for angle-distribution and asymmetry coefficient (AC) analyses. AC indicates deviation from a homogeneous distribution and is negative for angular distributions where the pulling-back force is dominant. **i**, Angle distributions of DMSO (816,941 angles) and 5Ph-IAA (828,733 angles). **j**, Verification of no significant DNA damage induced by RAD21 depletion. Right, fractions of cells with the indicated numbers of γH2AX immunostaining foci. Bars indicate mean ± s.e.m. across three independent biological replicates; dots indicate biological replicates (n = 3). Statistical details are provided in Supplementary Table 1. Source data

To explore how cohesin organizes chromatin domains in living human cells, we combined single-nucleosome imaging/tracking^5,29,44,45,54^ with rapid cohesin depletion using the auxin-inducible degron (AID2) system^55^. Single-nucleosome imaging/tracking can sensitively detect changes in the chromatin state in living cells. We used HCT116 cells expressing H2B-HaloTag (H2B-Halo)^45^ and RAD21-mAID-mClover (mAC^55^; Extended Data Fig. 1a,b). H2B-Halo was labeled with tetramethylrhodamine (TMR) to visualize nucleosomes genome wide, including those in both euchromatin and heterochromatin (Extended Data Fig. 1c). Oblique illumination (highly inclined and laminated optical sheet, HILO) microscopy^56^ enabled imaging of individual nucleosomes as TMR-labeled dots of H2B-Halo (Fig. 1a–c), which were precisely tracked in two-dimensional (2D) at 50 ms per frame for a total of 15–20 s, yielding ~1,400 trajectories per cell (Fig. 1b,d and Extended Data Fig. 1d,e; see Methods for further details). Position-determination accuracy is 12.5 nm (Extended Data Fig. 1d). Tracking was specific to nucleosome-incorporated H2B-Halo, as the small free H2B-Halo pool (3.3%) diffused too fast to be observed (Supplementary Video 1 and Extended Data Fig. 1f). The mean squared displacement (MSD) analysis revealed subdiffusive nucleosome motion with an anomalous exponent (α) of 0.43, suggesting constrained motion (Fig. 1e,g, black plots). Fixation nearly abolished this motion (gray plot, Fig. 1e,g).

Cohesin depletion through 5-phenyl-indole-3-acetic acid (5Ph-IAA) treatment for 1 h (Fig. 1f and Extended Data Fig. 1a) significantly increased motion across biological replicates, with the increased MSD exponent (0.48) and *D*_sub_ (subdiffusion coefficient; Fig. 1e,g and Supplementary Video 2). The radius of constraint (*R*_c_) metric^57^, which reports the spatial extent of nucleosome motion, was also significantly increased (Fig. 1h and Extended Data Fig. 1g). Turning angle distributions of nucleosome trajectories and asymmetry coefficient analysis^45,58^ showed a significant reduction in the pulling-back forces of nucleosomes after cohesin loss, suggesting that cohesin constrains nucleosome movement (Fig. 1h, Extended Data Fig. 1h,i and Supplementary Table 1). RAD21 depletion did not induce DNA damage (Extended Data Fig. 1j). These findings confirm that cohesin constrains local nucleosome motion in living cells, consistent with previous reports^5,21,42,43^.

### Cohesin constrains chromatin mainly through loop formation

**Fig. 2:**
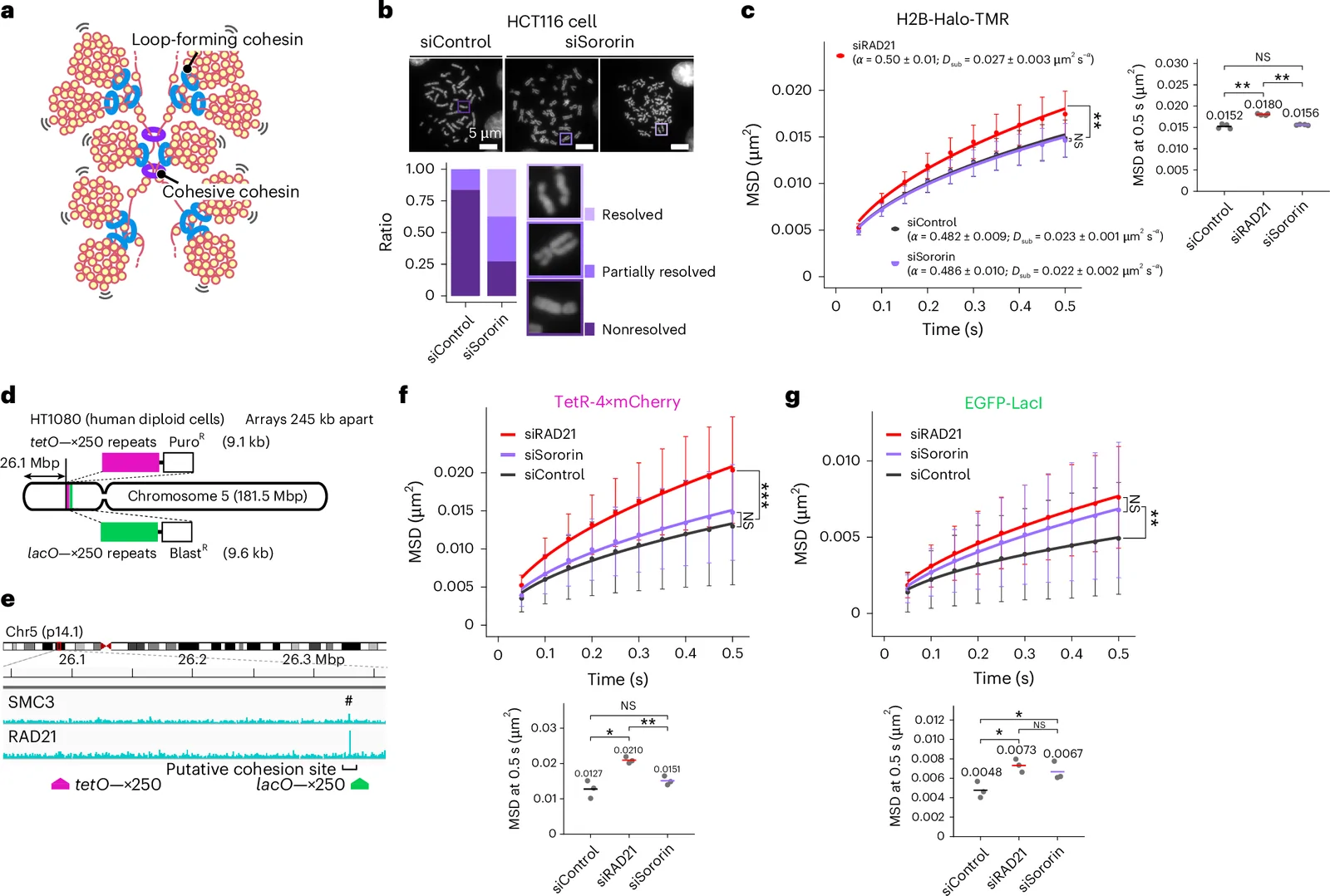
Sister chromatid cohesion does not affect global chromatin motion. **a**, Schematic of loop-forming cohesin and cohesive cohesin. **b**, Chromosome spreads of HCT116 cells with siControl or siSororin. Bottom: fraction of chromosomes with the three morphologies. **c**, MSD plots (±s.d. among cells) of H2B-Halo in late S–G2 synchronized HCT116 cells under the indicated conditions—siControl (black, *n* = 20 cells), siSororin (purple, *n* = 20 cells) and siRAD21 (red, *n* = 15 cells). *P* = 6.5 × 10^−5^ by the Kruskal–Wallis test, indicating significant overall differences among the groups. **P < 0.01 by Holm-adjusted Dunn’s test for siControl versus siRAD21 (*P* = 1.1 × 10^−3^). NS for siControl versus siSororin (*P* = 0.47). Right: replicate-aware quantification of MSD at 0.5 s. Each dot represents an independent biological replicate (*n* = 4). Bars indicate the mean. Statistical significance across biological replicates was assessed using a paired two-sided *t* test. **P < 0.01. Statistical details are provided in Supplementary Table 1. **d**, Schematic depicting the locations of *tetO* (×250) and *lacO* (×250) repeats inserted into human chromosome 5. **e**, ChIP–seq data showing the distribution of SMC3 and RAD21 across the genomic region around the integration sites of *lacO* and *tetO*. ChIP–seq data are from published data^63^. A hash (#) indicates the position of SMC3 and RAD21 peaks. **f**, MSD plots (±s.d. among cells) of *tetO*–TetR-4×mCherry in late S–G2 synchronized HT1080 cells under the indicated conditions—siControl (black, *n* = 34 cells), siSororin (purple, *n* = 36 cells) and siRAD21 (red, *n* = 37 cells). *P* = 3.7 × 10^−5^ by the Kruskal–Wallis test, indicating significant overall differences among the groups. ***P < 0.001 by Holm-adjusted Dunn’s test for siControl versus siRAD21 (*P* = 1.9 × 10^−5^). NS for siControl versus siSororin (*P* = 0.10). Please note that the following two factors make locus MSD noisier than single-nucleosome imaging/tracking. First, each locus provides only one trajectory per cell, so sampling is sparse. Second, the *lacO*/EGFP-LacI and *tetO*/TetR-4×mCherry foci are multi-emitter spots (many LacI/TetR) whose shape and intensity fluctuate, reducing localization precision. **g**, MSD plots (±s.d. among cells) of *lacO*–EGFP-LacI in late S–G2 synchronized HT1080 cells under the indicated conditions—siControl (black, *n* = 22 cells), siSororin (purple, *n* = 31 cells) and siRAD21 (red, *n* = 26 cells). *P* = 1.1 × 10^−2^ by the Kruskal–Wallis test, indicating significant overall differences among the groups. **P < 0.01 by Holm-adjusted Dunn’s test for siControl versus siRAD21 (*P* = 4.0 × 10^−3^). NS for siRAD21 versus siSororin (*P* = 0.11). Bottom: replicate-aware quantification of MSD at 0.5 s (*n* = 3), whereas MSD curves are shown from pooled cell data (**f**,**g**). Statistical significance across biological replicates was assessed using a paired two-sided *t* test. *P < 0.05, **P < 0.01. Full statistical details are provided in Supplementary Table 1. NS, not significant. Source data

**Extended Data Fig. 2:**
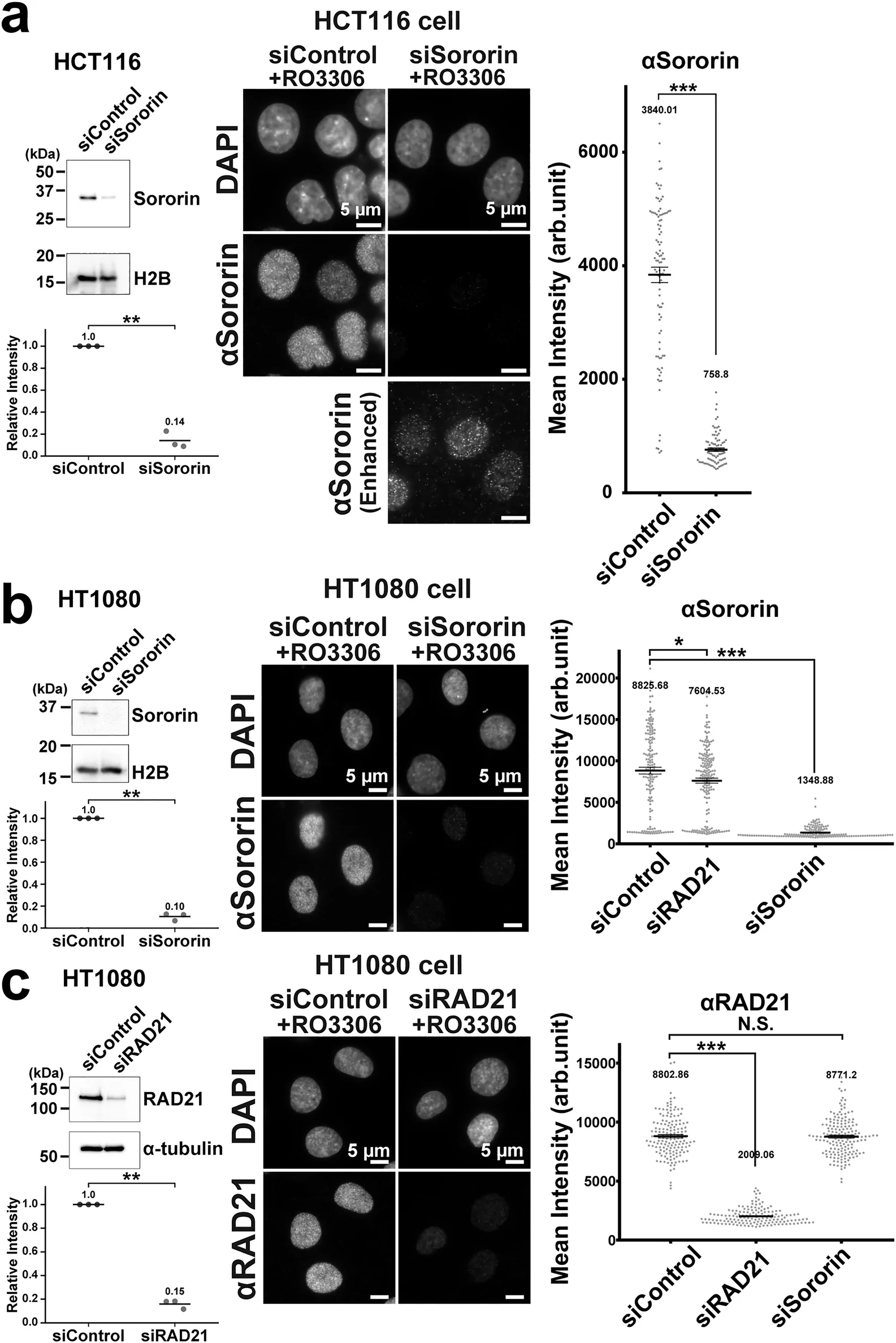
Validation of knockdown efficiency in HCT116 and HT1080 cells. **a**, Verification of the Sororin knockdown by immunoblotting (left: 86.0 ± 7.6% reduction (mean ± s.d.), each dot represents an independent biological replicate and bars indicate the mean (*n* = 3), **P < 0.01 (*P* = 2.57 × 10^−3^) by two-sided one-sample *t*-test) and immunostaining (mid, right). Right, individual dots represent cells; horizontal lines and error bars indicate the mean ± standard error of the mean (s.e.m.) across cells. Mean intensity values in immunostaining: 3.8 × 10^3^ for siControl (*n* = 97 cells) and 7.6 × 10^2^ for siSororin (*n* = 82 cells). ***P < 0.0001 (*P* = 3.2 × 10^−28^) by two-sided Wilcoxon rank-sum test. **b**, Verification of the Sororin knockdown by immunoblotting (left: 89.5 ± 3.3% reduction (mean ± s.d.), *n* = 3, **P < 0.01 (*P* = 4.56 × 10^−4^) by two-sided one-sample *t*-test) and immunostaining (mid, right). Mean intensity values in immunostaining: 8.8 × 10^3^ for siControl (*n* = 160 cells), 7.6 × 10^3^ for siRAD21 (*n* = 183 cells), and 1.3 × 10^3^ for siSororin (*n* = 157 cells). ***P < 0.0001 by two-sided Wilcoxon rank-sum test for siControl vs siSororin (*P* = 5.0 × 10^−41^). *P < 0.05 for siControl vs siRAD21 (*P* = 0.030). No adjustment was made for multiple comparisons. **c**, Verification of the RAD21 knockdown by immunoblotting (left: 84.0 ± 3.8% reduction (mean ± s.d.), *n* = 3, **P < 0.01 (*P* = 6.71 × 10^−4^) by two-sided one-sample *t*-test) and immunostaining (mid, right). Mean intensity values in immunostaining: 8.8 × 10^3^ for siControl (*n* = 154 cells), 2.0 × 10^3^ for siRAD21 (*n* = 153 cells), and 8.8 × 10^3^ for siSororin (*n* = 171 cells). ***P < 0.0001 by two-sided Wilcoxon rank-sum test for siControl vs siRAD21 (*P* = 7.9 × 10^−52^). NS, not significant for siControl vs siSororin (*P* = 0.90). No adjustment was made for multiple comparisons. Source data

To determine whether cohesin constrains chromatin through loop domains or sister chromatid cohesion (Fig. 2a), we knocked down Sororin, which is required for sister chromatid cohesion but not for loop formation^59–61^. Knockdown of Sororin disrupted cohesion in late S–G2 synchronized HCT116 cells (Fig. 2b and Extended Data Fig. 2a) but did not alter nucleosome motion (Fig. 2c), indicating that global constraints arise from loop formation, rather than sister chromatid cohesion. To examine whether cohesin affects local nucleosome motion near cohesion sites, we used a genome labeling system with *tetO*/*lacO* arrays 245 kb apart in chromosome 5 of HT1080 cells (Fig. 2d)^62^. In this system, the *lacO* array, but not *tetO* array, was located near a cohesin-enriched site (Fig. 2e)^63^. The *lacO* array more frequently appeared as a single dot than the *tetO* array, suggesting that the *lacO* array lies near a cohesion site^62^. These arrays were visualized with TetR-4×mCherry and EGFP-LacI, respectively (Fig. 2d,e). Sororin knockdown (Extended Data Fig. 2b) significantly increased EGFP-LacI mobility across biological replicates but not TetR-4×mCherry (Fig. 2f,g), whereas RAD21 depletion (Extended Data Fig. 2c) affected both (Fig. 2f,g), suggesting that cohesin constrains local regions near cohesion sites as previously reported in yeast^40^, while loop-forming cohesin globally constrains chromatin.

**Fig. 3:**
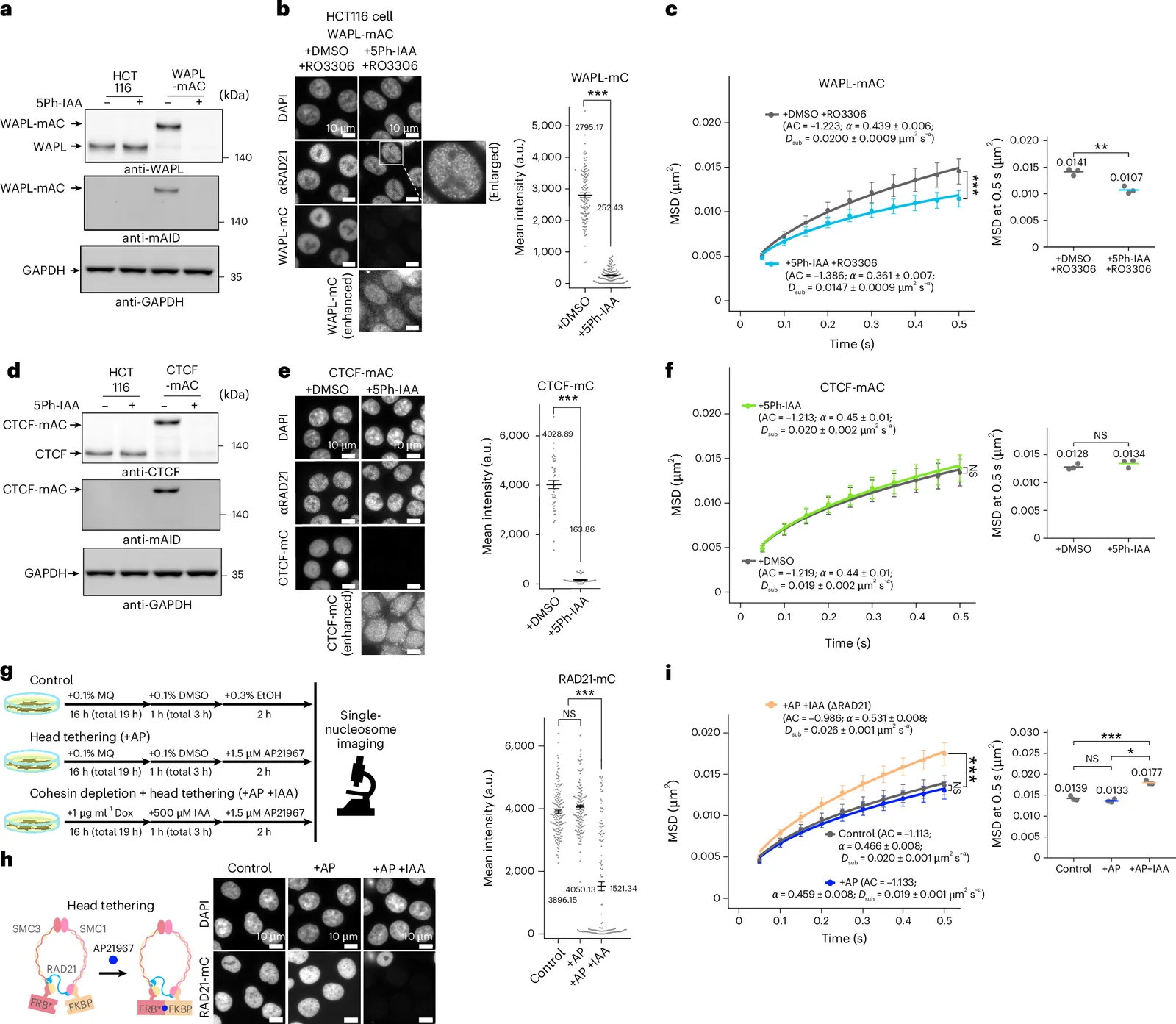
Nucleosome motion changes in response to altered cohesin activity. **a**,**b**, Verification of WAPL depletion by western blot (**a**) and mClover (mC) fluorescence signal (**b**). The enlarged image in **b** highlights the vermicelli-like structure in RAD21 staining, consistent with ref. ^64^. Right: individual dots represent cells; horizontal lines and error bars indicate the mean ± s.e.m. across cells. Mean intensity values—2.8 × 10^3^ for DMSO (*n* = 118 cells) and 2.5 × 10^2^ for 5Ph-IAA (ΔWAPL, *n* = 124 cells). ***P < 0.0001 (*P* = 4.7 × 10^−41^) by two-sided Wilcoxon rank-sum test. **c**, MSD plots (±s.d. among cells) of H2B-Halo in late S–G2 synchronized HCT116 cells (*n* = 30 cells) with indicated conditions—DMSO (black) or 5Ph-IAA (light blue). ***P = 8.3 × 10^−12^ for DMSO versus 5Ph-IAA by the two-sided Kolmogorov–Smirnov test. Right: replicate-aware quantification of MSD at 0.5 s. Each dot represents an independent biological replicate (*n* = 3). Bars indicate the mean. Statistical significance across biological replicates was assessed using a paired two-sided *t* test. **P < 0.01. Statistical details are provided in Supplementary Table 1. **d**,**e**, Verification of CTCF depletion by western blot (**d**) and mC fluorescence signal (**e**). Mean intensity values—4.0 × 10^3^ for DMSO (*n* = 47 cells) and 1.6 × 10^2^ for 5Ph-IAA (*n* = 46 cells). ***P < 0.0001 (*P* = 1.0 × 10^−16^) by two-sided Wilcoxon rank-sum test. **f**, MSD plots (±s.d. among cells) of H2B-Halo in HCT116 cells with indicated conditions—DMSO (black, *n* = 21 cells) or 5Ph-IAA (light green, *n* = 20 cells). NS for DMSO versus 5Ph-IAA (*P* = 0.67) by the two-sided Kolmogorov–Smirnov test. Right: replicate-aware quantification of MSD at 0.5 s (*n* = 3). Bars indicate the mean. Statistical significance across biological replicates was assessed using a paired two-sided *t* test. Statistical details are provided in Supplementary Table 1. **g**, Time course of head-tethering experiment. MQ, Milli-Q water. **h**, Verification of RAD21 depletion by mC signal. Right: individual dots represent cells; horizontal lines and error bars indicate the mean ± s.e.m. across cells. Mean intensity values—3.9 × 10^3^ for control (*n* = 184 cells), 4.1 × 10^3^ for AP21967 (AP; *n* = 161 cells) and 1.5 × 10^3^ for AP + IAA (IAA, *n* = 157 cells, Dox pretreated for the expression of OsTIR1 ubiquitin ligase). NS for control versus AP (*P* = 0.073). ***P < 0.0001 for control versus AP + IAA (*P* = 2.3 × 10^−28^) by two-sided Wilcoxon rank-sum test. No adjustment was made for multiple comparisons. Please note that only half of the population depleted RAD21. For single-nucleosome imaging, we selected depleted cells for analysis. **i**, MSD plots (±s.d. among cells) of H2B-Halo in HCT116 cells with indicated conditions—control (black, *n* = 20 cells), AP (blue, *n* = 20 cells) and AP + IAA (orange, *n* = 15 cells). *P* = 1.43 × 10^−7^ by Kruskal–Wallis test, indicating significant overall differences among the groups. ***P < 0.001 by Holm-adjusted Dunn’s test for control versus AP + IAA (*P* = 1.4 × 10^−5^). NS for control versus AP (*P* = 0.11). Right: replicate-aware quantification of MSD at 0.5 s (*n* = 3). Bars indicate the mean. Statistical significance across biological replicates was assessed using a paired two-sided *t* test. *P < 0.05, ***P < 0.001. Statistical details are provided in Supplementary Table 1. Dox, doxycycline. Source data

**Extended Data Fig. 3:**
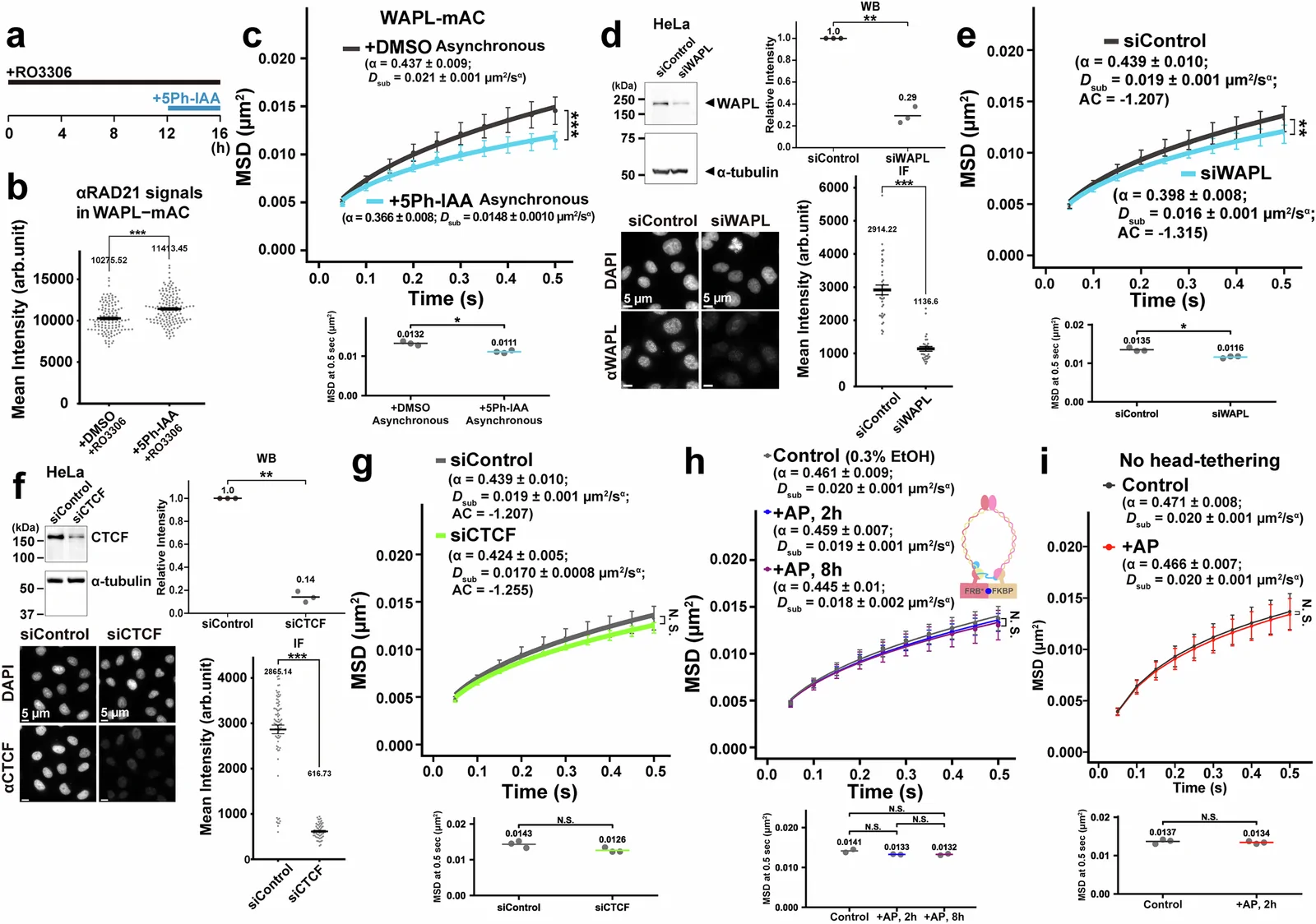
WAPL depletion, CTCF depletion, and cohesin head-tethering. **a**, Treatment timeline with RO3306 and 5Ph-IAA. **b**, RAD21 intensity in WAPL-depleted cells by immunostaining. Mean intensity values for DMSO (n = 125 cells) and 5Ph-IAA (n = 146 cells) are indicated. ***P = 6.1 × 10^−7^ by two-sided Wilcoxon rank-sum test. **c**, MSD plots (mean ± s.d. among cells) of H2B-Halo in asynchronous HCT116 cells: DMSO (black, n = 30 cells) and 5Ph-IAA (light blue, n = 30 cells). ***P = 8.25 × 10^−12^ by two-sided Kolmogorov–Smirnov test. Bottom, replicate-aware MSD at 0.5 s; dots indicate independent biological replicates (n = 3), bars indicate mean, paired two-sided t-test, *P < 0.05. **d**, Verification of WAPL depletion. Immunoblotting showed 70.7 ± 7.5% reduction (mean ± s.d., n = 3, **P = 3.77 × 10^−3^ by two-sided one-sample *t*-test). Immunostaining: siControl, 2.9 × 10^3^ (n = 37 cells); siWAPL, 1.1 × 10^3^ (n = 31 cells). ***P = 4.1 × 10^−16^ by two-sided Wilcoxon rank-sum test. **e**, MSD plots of H2B-Halo in HeLa cells: siControl (black, n = 15 cells) and siWAPL (blue, n = 15 cells). **P = 7.7 × 10^−3^ by two-sided Kolmogorov–Smirnov test. Bottom, replicate-aware MSD at 0.5 s, n = 3 biological replicates, paired two-sided *t*-test, *P < 0.05. **f**, Verification of CTCF depletion. Immunoblotting showed 85.9 ± 4.7% reduction (n = 3, **P = 1.01 × 10^−3^). Immunostaining: siControl, 2.9 × 10^3^ (n = 80 cells); siCTCF, 6.1 × 10^2^ (n = 60 cells). ***P = 1.6 × 10^−22^. **g**, MSD plots for siControl (n = 15 cells) and siCTCF (n = 15 cells); NS, *P* = 0.18. Bottom, replicate-aware MSD at 0.5 s, n = 3 biological replicates, paired two-sided *t*-test. **h**, MSD plots for head-tethered cohesin after AP21967 (AP) treatment (n = 20 cells per condition); NS for control vs AP 2 h (P = 0.17) and control vs AP 8 h (*P* = 0.081). **i**, MSD plots in cells without head-tethering (n = 30 cells per condition); NS, *P* = 0.59. Statistical details are provided in Supplementary Table 1. Source data

We tested whether increasing the number of loops enhances constraint. Because WAPL releases cohesin from chromatin, WAPL depletion increases the number of loops^64–66^, particularly when depletion is induced after the completion of DNA replication. WAPL depletion in HCT116-WAPL-mAC cells (Fig. 3a)^67^ synchronized in late S–G_2_ phase (Extended Data Fig. 3a) reduced the WAPL-mClover signal (Fig. 3b) and increased RAD21 chromatin binding (Extended Data Fig. 3b), which led to significantly decreased MSD and asymmetry coefficient values across biological replicates (Fig. 3c and Supplementary Table 1). A similar finding was observed in asynchronous HCT116 cells and siRNA-treated asynchronous HeLa cells (Extended Data Fig. 3c–e). Thus, more cohesin loops constrain chromatin more strongly. To assess whether loop boundaries contribute to chromatin constraint, we acutely depleted CTCF in HCT116 cells (Fig. 3d,e)^55^. Despite its role in TAD boundary formation^68^, CTCF loss did not affect nucleosome motion (Fig. 3f and Extended Data Fig. 3f,g), consistent with unchanged cohesin binding levels^52,69^ and previous genomic array labeling systems^42,43^.

Finally, to examine the possible involvement of active loop extrusion^34,70^ in the constraining process, we used a head-tethered cohesin system^71^ that stabilizes cohesin through forced head domain closure using AP21967 (Fig. 3g,h)^72,73^. Head tethering weakened loop-extrusion activity in vitro^71^. Head tethering alone did not change nucleosome motion on the 0.5 s timescale, but head tethering after RAD21 depletion led to the loss of the entire cohesin complex^71^ and increased motion (Fig. 3i and Extended Data Fig. 3h,i). These results suggest that chromatin constraint by cohesin may not require active loop extrusion. Also, the difference between head tethering and WAPL depletion explains the distinct outcomes. WAPL depletion suppresses cohesin dissociation, allowing cohesin to remain bound and to continue to accumulate on chromatin. By contrast, head-tethered cohesin is immobilized at existing sites and does not promote new chromatin loading. Consequently, WAPL depletion yields much higher cohesin occupancy and stronger chromatin constraint than head tethering (Fig. 3c,i).

**Extended Data Fig. 4:**
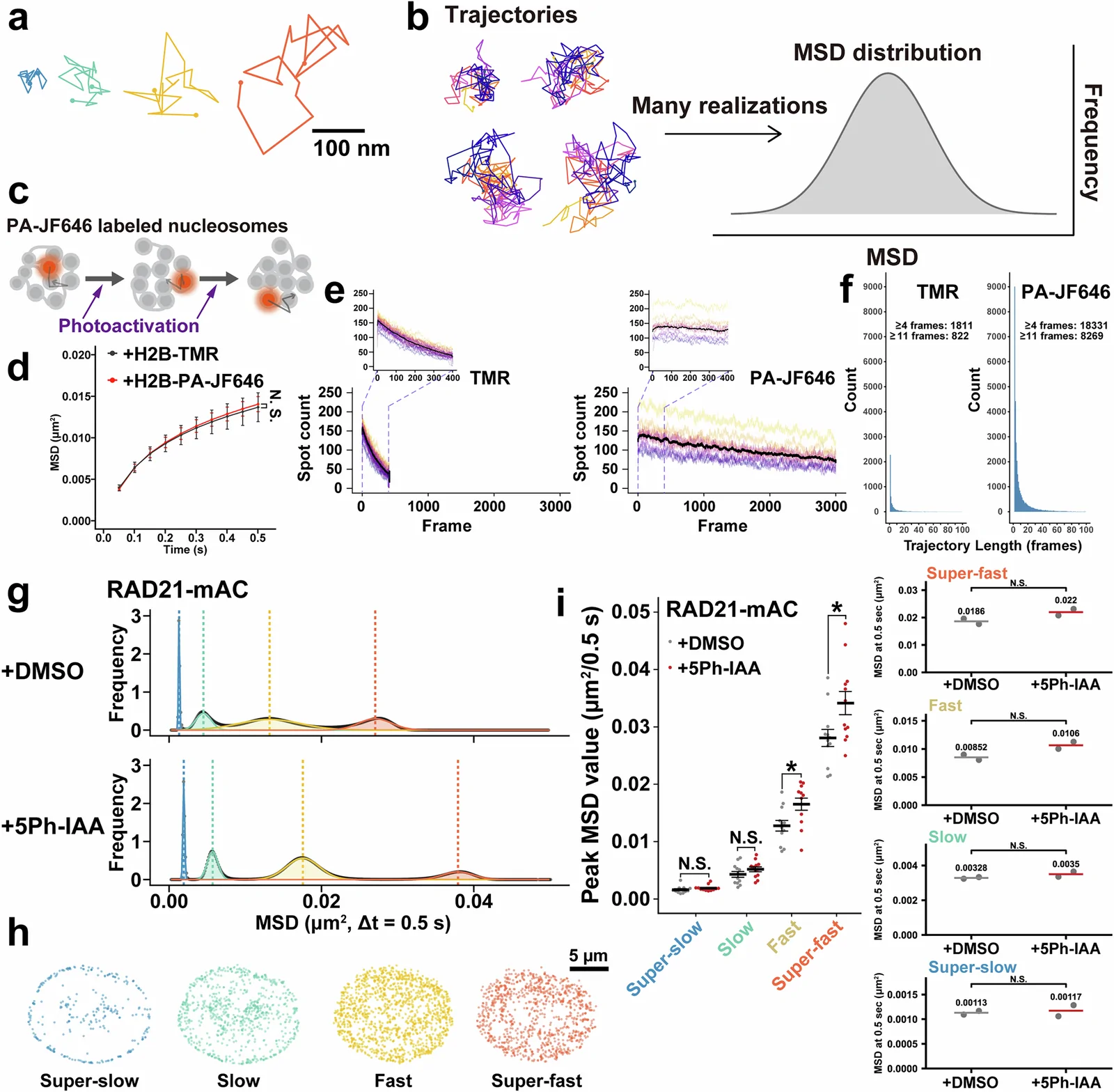
RL algorithm analysis of single-nucleosome trajectories after cohesin depletion. **a**, Representative trajectories of four tracked single nucleosomes, each belonging to a different subpopulation. **b**, Schematic of the RL algorithm. **c**, Schematic for PA-JF646 labeling and photoactivation of labeled nucleosomes. **d**, MSD plots (mean ± s.d. among cells) of H2B-Halo in HCT116 cells, labeled with TMR (black, *n* = 30 cells); PA-JF646 (red, *n* = 20 cells). NS, not significant (*P* = 0.09) by the two-sided Kolmogorov–Smirnov test, indicating that a similar nucleosome motion was observed with two fluorescent dyes. **e**, Number of detected dots per frame in H2B-Halo-TMR (left, *n* = 30 cells) and H2B-Halo-PA-JF646 (right, *n* = 11 cells). Both conditions showed a similar initial dot count. The number of detected dots decreased over time due to photobleaching in both cases; however, in the PA-JF646 condition, newly appearing dots from photoactivation led to a milder decrease. **f**, Distribution of trajectory lengths for TMR-labeled and PA-JF646-labeled H2B-Halo in a representative nucleus. Approximately 10-fold more trajectories were obtained with PA-JF646 labeling. **g**, Distribution of MSD (Δt = 0.5 s) from H2B-Halo trajectories in a single cell was denoised using the RL algorithm and fitted with four Gaussian components: ‘superslow’ (blue), ‘slow’ (green), ‘fast’ (yellow), and ‘superfast’ (orange). Black lines indicate the denoised raw data, while colored lines represent the fitted Gaussians. The peak of each Gaussian is marked with a dotted line. In this single-cell analysis, only the ‘fast’ (yellow) and ‘superfast’ (orange) peaks appear to shift rightward upon cohesin depletion (+5Ph-IAA), suggesting further acceleration of these subpopulations. **h**, Nuclear localization of nucleosomes categorized into each subpopulation. Each dot represents the centroid of a trajectory. ‘Superfast’ and ‘fast’ trajectories appear biased in nucleoplasm, presumably corresponding to euchromatin. **i**, Left, peak MSD values of the four Gaussian components. Each dot represents data from a single nucleus. *n* = 12 cells per condition. *P < 0.05 for ‘superfast’ (*P* = 0.025) and for ‘fast’ (*P* = 0.015). NS, not significant for ‘slow’ (*P* = 0.19) and ‘superslow’ (*P* = 0.24) by the two-sided Student’s *t*-test. Right, replicate-aware quantification of each subpopulation at 0.5 s (*n* = 2). Each dot represents an independent biological replicate. Bars indicate the mean. Please note that reproducible trends toward increases were observed in the ‘superfast’ and ‘fast’ fractions, but these differences were not statistically significant. NS, not significant. Statistical details are provided in Supplementary Table 1. Source data

We previously observed heterogeneous nucleosome motions (Extended Data Fig. 4a,b), which suggested the presence of domain subpopulations, potentially linked to chromatin context^26,30^. To get a clue of whether cohesin preferentially constrains euchromatin or heterochromatin, we used motion classification with the Bayesian-based Richardson–Lucy (RL) algorithm^74^. For RL algorithm analysis, we obtained a tenfold increase in H2B-Halo-nucleosome trajectory data using the PA-JF646 HaloTag ligand^75^ and constant photoactivation with weak 405 nm illumination (Extended Data Fig. 4c–f). We then classified H2B-Halo trajectories into the following four motion types in each cell: ‘superslow’, ‘slow’, ‘fast’ and ‘superfast’ (Extended Data Fig. 4g and Supplementary Video 3). The ‘fast’ and ‘superfast’ subpopulations are likely euchromatic, localized within the nucleoplasm (Extended Data Fig. 4h and Supplementary Video 3). In this single-cell analysis, cohesin depletion was associated with a rightward shift of these populations (Extended Data Fig. 4g,i), implying that cohesin constrains movements of nucleosomes mainly in euchromatin.

### H3.3 labeling reveals cohesin-constrained euchromatin

**Fig. 4:**
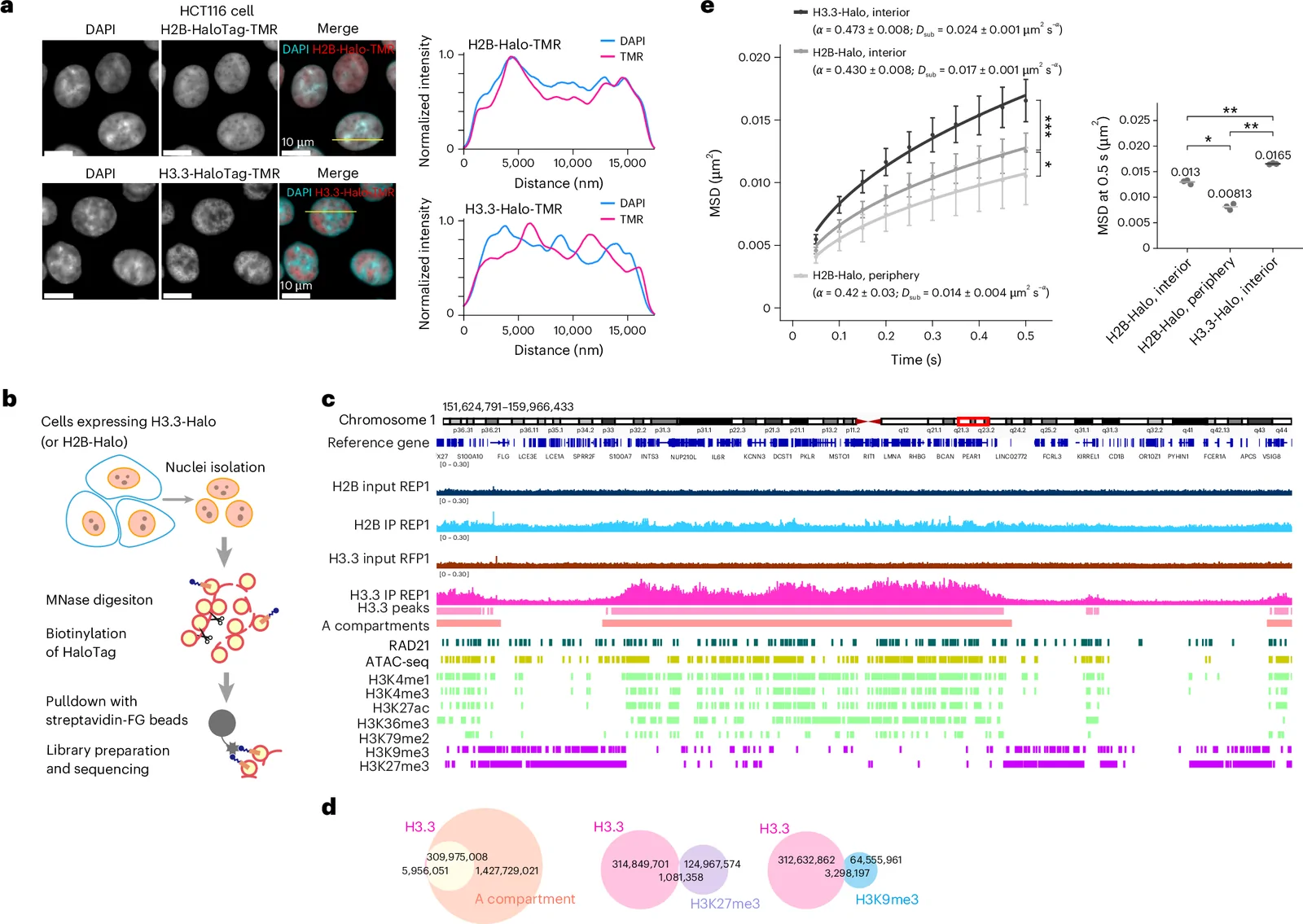
H3.3-Halo is enriched in euchromatin (Hi–C A compartment). **a**, Top: HCT116 cells expressing H2B-Halo labeled with DAPI and TMR. Top right: intensity line profiles of DAPI and H2B-Halo-TMR at the yellow line of the merged image. Please note that the localization of H2B-Halo-TMR is similar to the DAPI-stained DNA. Bottom: HCT116 cells expressing H3.3-Halo labeled with DAPI and TMR. Bottom right: intensity line profiles of DAPI and H3.3-Halo-TMR at the yellow line of the merged image. Please note that H3.3-Halo-TMR is enriched at DAPI-less (euchromatin) regions. The experiment was independently repeated at least thrice with similar results. **b**, A purification scheme for nucleosomes with H3.3-Halo or H2B-Halo and their associated genomic DNA. The enriched DNA fractions were then indexed, amplified and sequenced. FG, ferrite–glycidyl methacrylate. **c**, H3.3-Halo-incorporated genomic regions were enriched with active histone marks and excluded from an inactive mark—view from chr1: 151,624,791–159,966,433 (also see Extended Data Fig. 5d). **d**, Overlap between H3.3-Halo peaks and the A compartment^106^, between H3.3-Halo peaks and H3K27me3 regions and between H3.3-Halo peaks and H3K9me3 regions. **e**, MSD plots (±s.d. among cells) of H2B-Halo (nuclear interior; *n* = 30 cells), H2B-Halo (nuclear periphery (basal nuclear surface); *n* = 8 cells) or H3.3-Halo (nuclear interior; *n* = 30 cells) in HCT116 cells. ***P = 8.3 × 10^−12^ for H2B-Halo, interior versus H3.3-Halo, interior by the two-sided Kolmogorov–Smirnov test, *P = 4.2 × 10^−3^ for H2B-Halo, interior versus H2B-Halo, periphery by the two-sided Kolmogorov–Smirnov test. Spot-on analysis of H3.3-Halo is shown in Extended Data Fig. 5t. Right: replicate-aware quantification of MSD at 0.5 s. Each dot represents an independent biological replicate (*n* = 3). Bars indicate the mean. Statistical significance across biological replicates was assessed using a paired two-sided *t* test. *P < 0.05, **P < 0.01. Statistical details are provided in Supplementary Table 1. Source data

**Extended Data Fig. 5:**
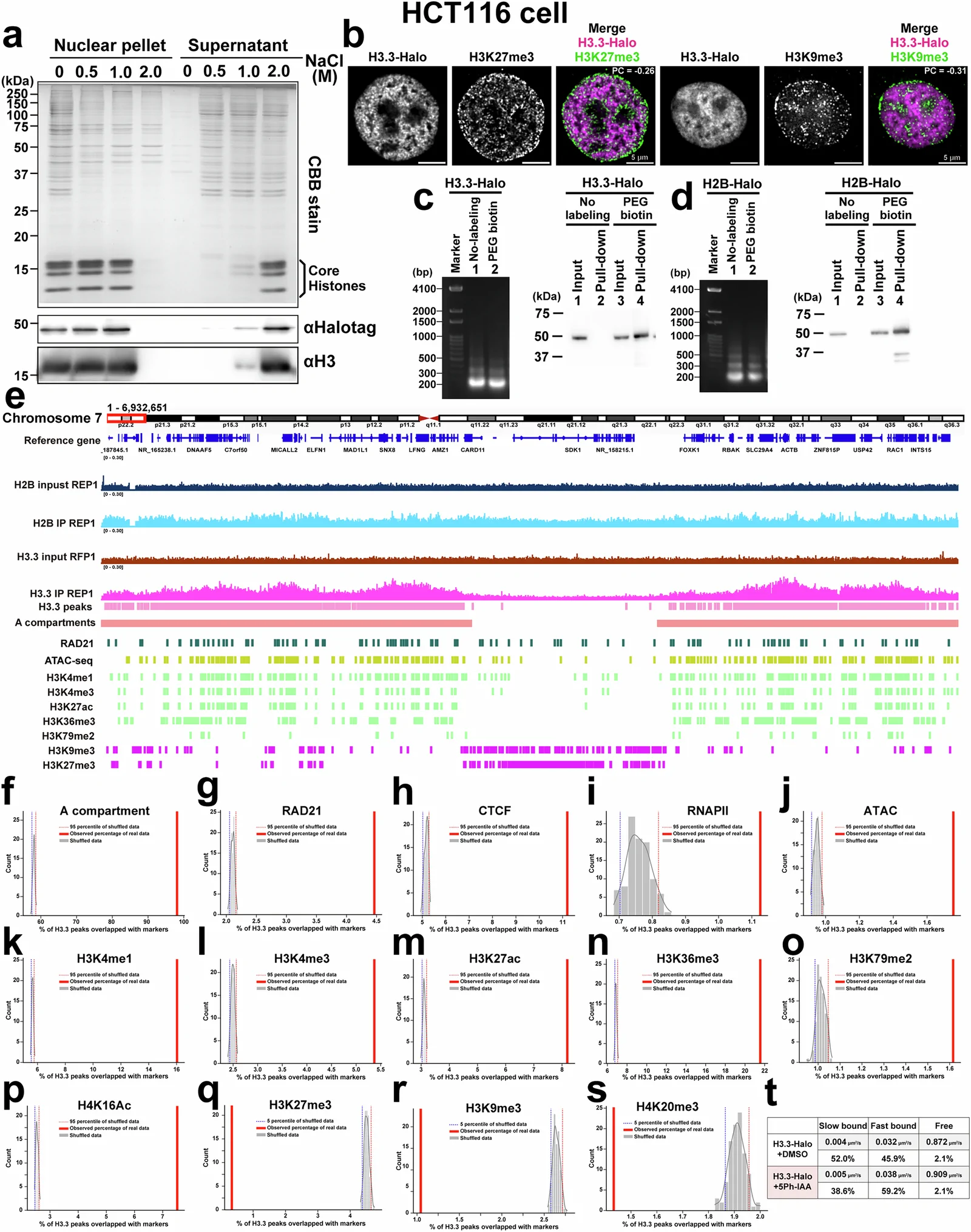
H3.3-Halo pulldown shows H3.3-Halo nucleosomes are enriched in euchromatin. **a**, Stepwise salt washing of nuclei expressing H3.3-Halo. The nuclei isolated from HCT116 cells expressing H3.3-Halo were washed with increasing concentrations of NaCl. The resulting nuclear pellets (left) and supernatants (right) were analyzed by SDS–PAGE, followed by Coomassie brilliant blue staining (top) or immunoblotting for H3 and HaloTag (bottom). Endogenous H3 and H3.3-Halo started to dissociate from chromatin with 1 M NaCl and were detected in the supernatant fraction, suggesting that nucleosomes containing H3.3-Halo have similar stability to those containing endogenous H3. The image shown was obtained from a single experiment. **b**, HCT116 cells expressing H3.3-Halo, labeled with JFX650 and immunostained for H3K27me3 (left) or H3K9me3 (right). PC, Pearson’s correlation for the images. Pearson’s correlation coefficient R = −0.28 ± 0.27 (mean ± s.d. among cells, *n* = 9 cells) between H3.3-Halo and H3K27me3, and R = −0.29 ± 0.08 (mean ± s.d. among cells, *n* = 11 cells) between H3.3-Halo and H3K9me3. The larger variation for H3K27me3 is consistent with its generally more variable nuclear distribution compared with H3K9me3. The experiment was independently repeated three times with similar results. **c**, Left, agarose gel electrophoresis of nucleosomal DNA purified from HCT116 cells expressing H3.3-Halo. Please note that the major fractions are mononucleosomal DNA. Right, enrichment of the pulldown nucleosomes validated by western blotting with an anti-HaloTag antibody. **d**, Left, agarose gel electrophoresis of nucleosomal DNA purified from HCT116 cells expressing H2B-Halo. Please note that the major fractions are mononucleosomal DNA. Right, enrichment of the pulldown nucleosomes validated by western blotting with an anti-HaloTag antibody. For **c** and **d**, the experiment was independently repeated two times with similar results. **e**, Another example of H3.3-Halo-nucleosome regions: chr7: 1–6,932,651. **f**–**s**, Distributions of the percentages of (**f**) A compartment, (**g**) RAD21, (**h**) CTCF, (**i**) RNAP II, (**j**) ATAC-seq, active histone marks, (**k**) H3K4me1, (**l**) H3K4me3, (**m**) H3K27ac, (**n**) H3K36me3, (**o**) H3K79me2, (**p**) H4K16Ac, and the inactive histone mark (**q**) H3K27me3, (**r**) H3K9me3, and (**s**) H4K20me3 overlapped with 100 randomly shuffled datasets of H3.3-Halo peaks. The red solid line indicates the percentage observed in the real H3.3-Halo dataset, while the dotted red and blue lines indicate the 95th and 5th percentiles of the shuffled distribution, respectively. **t**, Diffusion coefficients and percentages of fractions estimated by Spot-on. H3.3-Halo in HCT116 RAD21-mAC labeled with TMR is examined by 10 ms/frame imaging. Please note that free diffusing fractions are almost negligible in all cases. Source data

**Extended Data Fig. 6:**
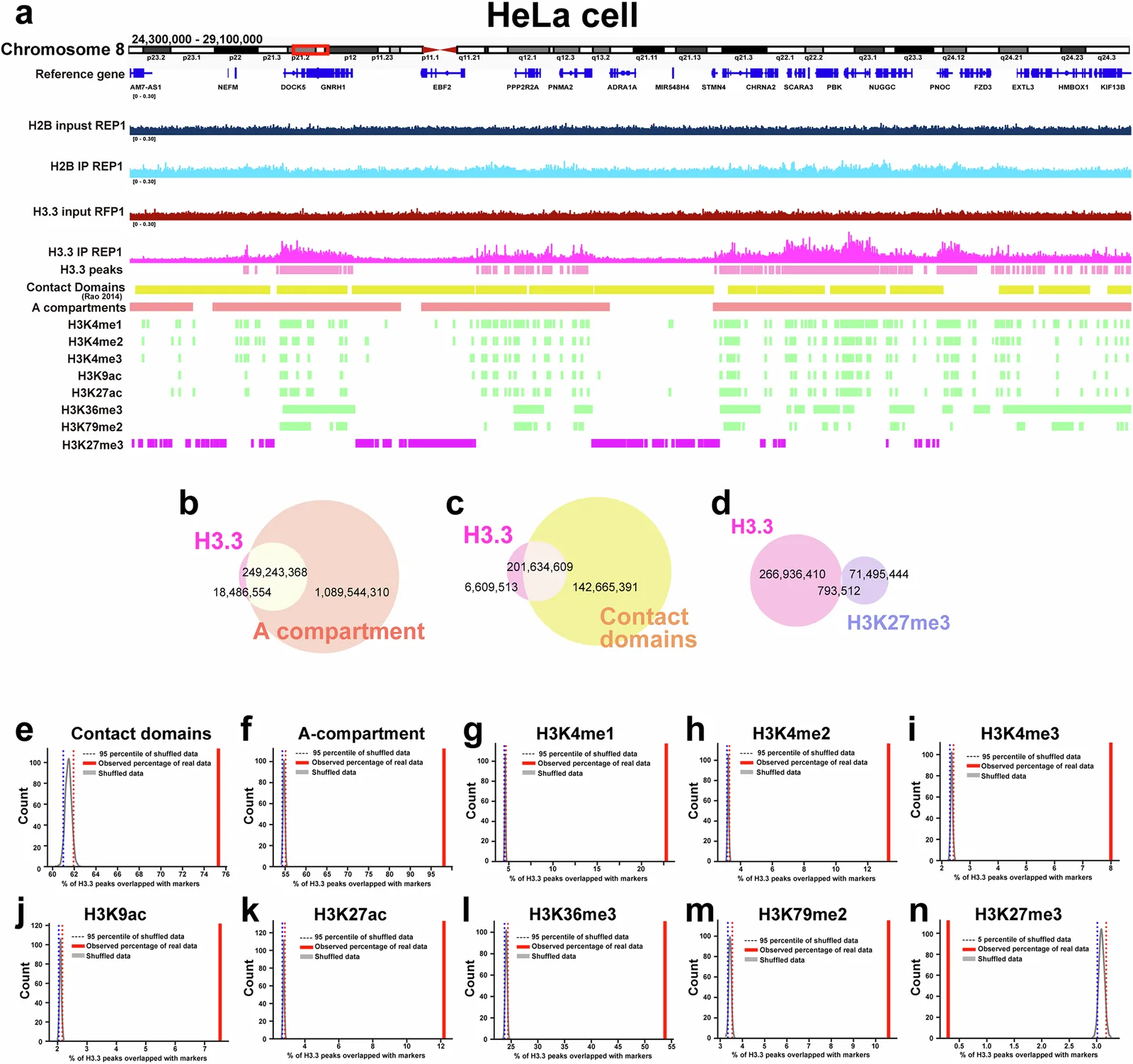
H3.3-Halo nucleosomes are also enriched in euchromatin in HeLa cells. **a**, Examples of H3.3-Halo-nucleosome regions: chr8: 24,300,000–29,100,000. **b**–**d**, Venn diagrams showing the overlap (in bp) between H3.3-Halo-nucleosome regions and A compartment (**b**), between H3.3-Halo-nucleosome regions and contact domains (**c**), and between H3.3-Halo-nucleosome regions and H3K27me3 regions (**d**). **e**–**n**, Distributions of the percentages of (**e**) contact domains, (**f**) A compartment, active histone marks, (**g**) H3K4me1, (**h**) H3K4me2, (**i**) H3K4me3, (**j**) H3K9ac, (**k**) H3K27ac, (**l**) H3K36me3, (**m**) H3K79me2, and the inactive histone mark (**n**) H3K27me3 overlapped with 1,000 randomly shuffled datasets of H3.3-Halo peaks. The red solid line indicates the percentage observed in the real H3.3-Halo dataset, while the dotted red and blue lines indicate the 95th and 5th percentiles of the shuffled distribution, respectively. Source data

To investigate euchromatin more specifically, we labeled nucleosomes using a euchromatin-specific histone variant H3.3 (refs. ^49–51^). We stably expressed H3.3-Halo in HCT116 cells (Fig. 4a). Expressed H3.3-Halo was properly incorporated into the nucleosome (Extended Data Fig. 5a) and localized to DAPI-poor regions in nuclei (Fig. 4a) and to regions largely complementary to repressive histone marks (Extended Data Fig. 5b). Pulldown of H3.3-Halo-incorporated nucleosomes and genomic sequencing (Fig. 4b and Extended Data Fig. 5c,d) confirmed enrichment in Hi–C A compartments (98.1%) with active histone marks, and depletion from repressive marks (Fig. 4c,d and Extended Data Fig. 5e–s). We observed a similar trend in HeLa cells stably expressing H3.3-Halo as well (Extended Data Fig. 6).

**Extended Data Fig. 7:**
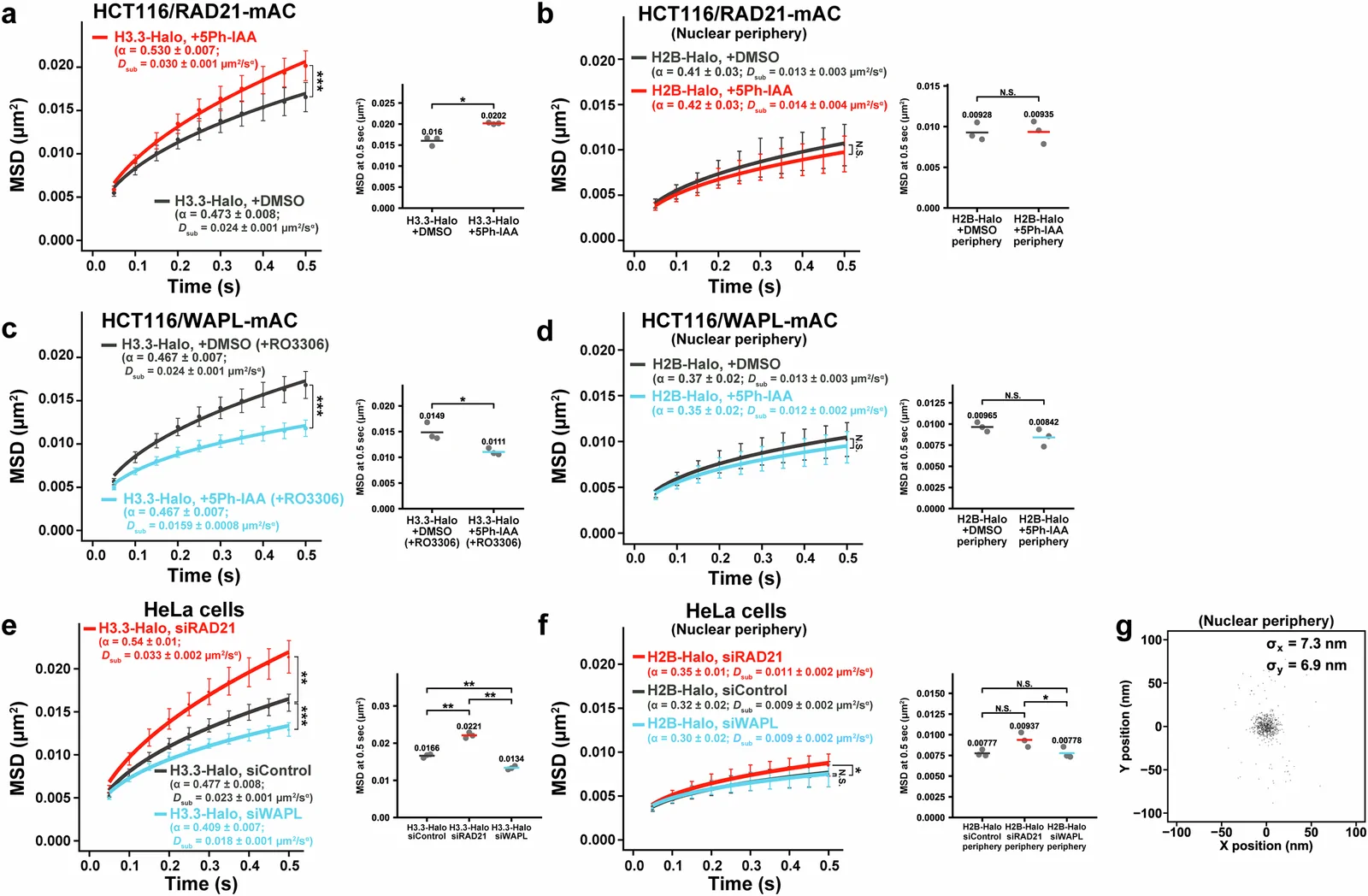
Changes in the motion of euchromatic nucleosomes and peripheral nucleosomes upon cohesin depletion. **a**–**f**, Right, replicate-aware quantification of MSD at 0.5 s. Each dot represents an independent biological replicate (*n* = 3). Bars indicate the mean. Statistical significance across biological replicates was assessed using a paired two-sided *t*-test. NS, not significant; *P < 0.05, **P < 0.01. Statistical details are provided in Supplementary Table 1. **a**, MSD plots (mean ± s.d. among cells) of H3.3-Halo in HCT116 cells with indicated conditions: DMSO (black, *n* = 30 cells) or 5Ph-IAA (ΔRAD21, red, *n* = 28 cells). ***P < 0.001 by the two-sided Kolmogorov–Smirnov test (*P* = 8.2 × 10^−12^). **b**, MSD plots (mean ± s.d. among cells) of H2B-Halo at the nuclear periphery in HCT116 cells with indicated conditions: DMSO (black, *n* = 8 cells) or 5Ph-IAA (ΔRAD21, red, *n* = 9 cells). NS, not significant by the two-sided Kolmogorov–Smirnov test (*P* = 0.89). **c**, MSD plots (mean ± s.d. among cells) of H3.3-Halo in HCT116 cells with indicated conditions: DMSO (black, *n* = 30 cells) or 5Ph-IAA (ΔWAPL, light blue, *n* = 30 cells). ***P < 0.001 by the two-sided Kolmogorov–Smirnov test (*P* = 1.7 × 10^−17^). **d**, MSD plots (mean ± s.d. among cells) of H2B-Halo at the nuclear periphery in HCT116 cells with indicated conditions: DMSO (black, *n* = 13 cells) or 5Ph-IAA (ΔWAPL, light blue, *n* = 14 cells). NS, not significant by the two-sided Kolmogorov–Smirnov test (*P* = 0.26). **e**, MSD plots (mean ± s.d. among cells) of H3.3-Halo in HeLa cells with indicated conditions: siControl (black, *n* = 15 cells), siRAD21 (red, *n* = 15 cells), and siWAPL (light blue, *n* = 15 cells). *P* = 1.0 × 10^−8^ by Kruskal–Wallis test, indicating significant overall differences among groups. **P < 0.01 for siControl vs siRAD21 (*P* = 1.4 × 10^−3^) and siControl vs siWAPL (*P* = 2.5 × 10^−3^). **f**, MSD plots (mean ± s.d. among cells) of H2B-Halo at the nuclear periphery (basal nuclear surface) in HeLa cells with indicated conditions: siControl (black, *n* = 15 cells), siRAD21 (red, *n* = 15 cells), and siWAPL (light blue, *n* = 15 cells). *P* = 4.9 × 10^−3^ by Kruskal–Wallis test, indicating significant overall differences among groups. **P < 0.01 for siControl vs siRAD21 (*P* = 7.0 × 10^−3^). NS, not significant for siControl vs siWAPL (*P* = 0.40). **g**, Position determination for the accuracy of H2B-Halo-TMR on the nuclear periphery. Distribution of nucleosome displacements from their centroid in the x–y plane in the 50-ms interval. *n* = 11 nucleosomes in an FA-fixed cell. SDx and SDy were 7.3 nm and 6.9 nm, respectively. Source data

Single-nucleosome imaging in HCT116 cells showed that H3.3-Halo nucleosomes were significantly more mobile than H2B-Halo ones across biological replicates (Fig. 4e) and had a stronger response to RAD21 or WAPL depletion (Extended Data Fig. 7a,c)^55,67^, indicating a greater cohesin impact in euchromatin. In contrast, H2B-labeled nucleosomes around the nuclear periphery (basal nuclear surface; for details, see Methods)^76,77^, which is enriched with constitutive heterochromatin, or lamin-associated domains^78^, remained largely unaffected (Extended Data Fig. 7b,d). A similar observation was found in depletion of RAD21 and WAPL through siRNA in HeLa cells expressing H3.3-Halo (Extended Data Fig. 7e) and H2B-Halo around the nuclear periphery (Extended Data Fig. 7f). These results suggest that cohesin primarily constrains euchromatic regions, while our results do not exclude the possibility that cohesin may also influence facultative heterochromatin regions under certain conditions.

### Super-resolution imaging uncovers condensed euchromatin

**Fig. 5:**
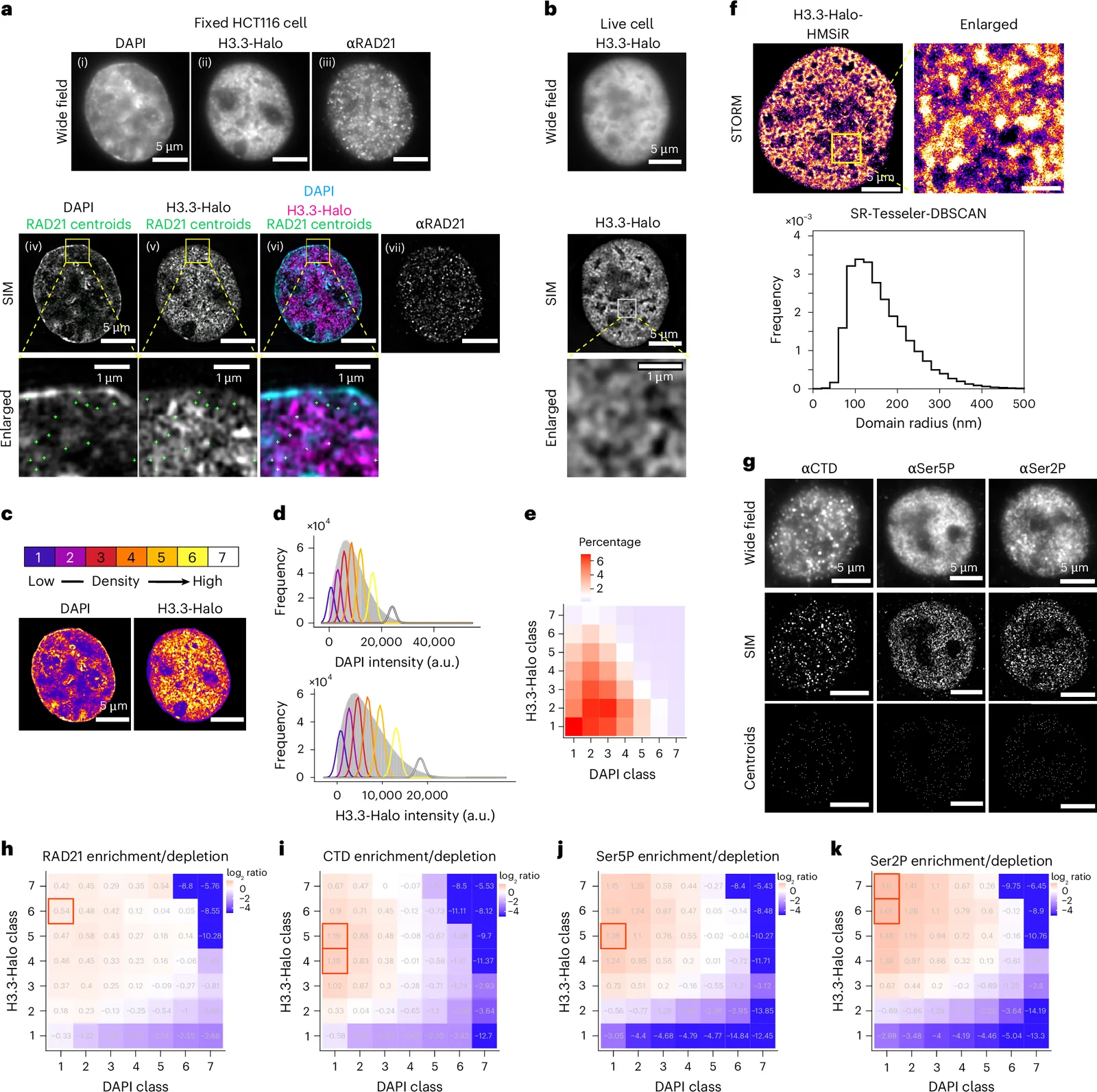
3D-SIM imaging revealed condensed euchromatic domains in human cells and localization of cohesin and RNAP II. **a**, Representative wide-field view ((i)–(iii)) and 3D-SIM reconstructed view ((iv)–(vi)) of FA-fixed HCT116 cells. Chromatin was labeled with DAPI ((i) and (iv)) and H3.3-Halo-TMR ((ii) and (v)). A wide-field immunostaining image of RAD21 is also shown ((iii)); (vi) is a merged image of (iv) and (v). Bottom images are enlarged views of the boxed regions in (iv)–(vi); (vii) is a SIM image of RAD21 immunostaining. The wide-field image ((i)–(iii)) represents the total intensity projection of 15 raw SIM frames. Green dots in the enlarged images indicate the centroids of RAD21 immunostaining signals from (vii). **b**, Representative wide-field view (top) and 3D-SIM reconstructed view (bottom) of live HCT116 cells. **c**, Heatmap of DAPI or H3.3-Halo-TMR compaction proxies, based on the intensity classification into seven classes^107^. The color code assigns DAPI or H3.3-Halo-TMR signals into seven classes with equal intensity variance. This approach enables threshold-independent signal intensity classification based on the intensity of individual voxels. **d**, Histogram of the DAPI (top) or H3.3-Halo-TMR (bottom) intensity from a representative nucleus image (gray), overlaid with probability density functions of the seven Gaussians generated by the classification analysis. **e**, Proportion of nuclear volume across 7 × 7 categories based on DAPI and H3.3-Halo-TMR intensity classes in a representative nucleus. **f**, Representative STORM reconstructed view of H3.3-Halo**-**HMSiR. Histogram of estimated radius of euchromatic domains (bottom) by SR-Tesseler-DBSCAN pipeline (Methods). **g**, Representative images of immunostaining signals of various RNAP II antibodies. Wide-field view (top), 3D-SIM reconstructed view (middle) and detected centroids of immunostaining signals (bottom). The wide-field image represents the total intensity projection of 15 raw SIM frames. Please note that 3D-SIM reconstruction efficiently suppresses out-of-focus blur and therefore provides highly confined optical sectioning with a narrow depth of field. For **a**, **b** and **f**, the experiment was independently repeated at least thrice with similar results. For **g**, the image shown was obtained from a single experiment. **h**–**k**, Each voxel within a single nucleus was classified into 7 × 7 categories based on DAPI and H3.3-Halo-TMR intensity classes. Target signal enrichment/depletion is shown as the log_2_ ratio of the signal in each class to the relative volume of that class. The displayed values represent the median across multiple cells. For categories with no detected signal, a small constant was added to avoid undefined log values; these categories, with resulting values below –5, are shown in blue. RAD21 (**h**), RNAP II CTD (**i**), RNAP II Ser5P (**j**) and RNAP II Ser2P enrichment/depletion (**k**). The same data are used as in Extended Data Fig. 8i–l. Source data

**Extended Data Fig. 8:**
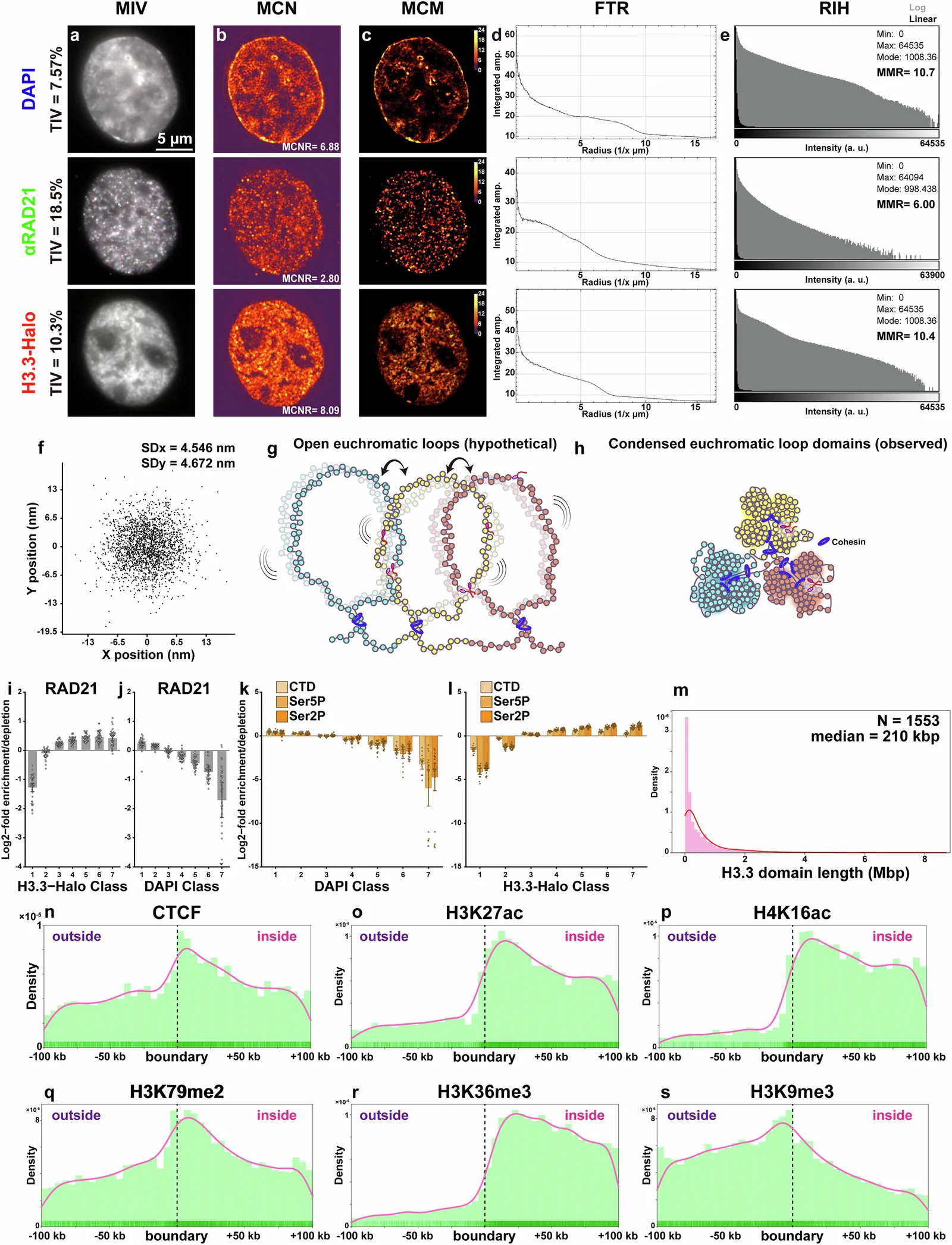
Quality control of 3D-SIM and STORM imaging, and localization of chromatin-associated factors relative to H3.3-Halo domains. **a**–**e**, Quality of 3D-SIM images and reconstructions assessed with SIMcheck^79^. The images also achieved the best N-SIM score (8 on a 1–8 scale). Results for the three channels (DAPI, anti-RAD21, H3.3-Halo-TMR) are shown. **a**, TIV: total intensity variation; MIV: motion & illumination variation. TIV and MIV indicate minimal motion and uniform illumination in the raw images. **b**, MCN: modulation contrast to noise; MCNR: modulation contrast-to-noise ratio. MCN maps the local stripe modulation contrast in the raw data. MCNR represents the average value of features. **c**, MCM: modulation contrast map. MCM indicates the high underlying modulation contrast of the features in the reconstructed image. **d**, FTR: radially averaged Fourier transform. FTR amplitude profile plots indicate an effective lateral resolution of ~100 nm for the blue channel and ~140 nm for the green and red channels. **e**, RIH: intensity histogram of the reconstructions; MMR: max-to-min intensity ratio. Both indicate sufficiently high reconstruction contrast. **f**, Localization precision for STORM (H2B-Halo-HMSiR). Distribution of nucleosome displacements from their centroid in the x–y plane at 10-ms intervals in an FA-fixed cell (*n* = 360 nucleosomes). SDx = 4.546 nm; SDy = 4.672 nm. **g**,**h**, Hypothetical open euchromatic loops (**g**) may provide weaker transcriptional insulation between domains. By contrast, the condensed euchromatic loop domains observed here (**h**) can provide stronger insulation between domains. **i**,**j**, Bar plots of the log_2_-fold enrichment or depletion of RAD21 in each segmented chromatin class relative to class volume for H3.3-Halo-TMR classes (**i**) and DAPI classes (**j**). Individual dots represent cells, bars show the mean, and error bars indicate 95% CI across individual cells (n = 37 cells). **k**,**l**, Bar plots of the log_2_-fold enrichment or depletion of RNAP II forms in each segmented chromatin class relative to class volume. **k** and **l** are based on DAPI classes and H3.3-Halo-TMR classes, respectively. Individual dots represent cells, bars show the mean, and error bars indicate s.d. across individual cells (RNAP II form: CTD, n = 15 cells; Ser5P, n = 19 cells; Ser2P, n = 23 cells). **m**, Distribution of detected H3.3 domain lengths. The red line shows a kernel density estimate (KDE) overlaid on the histogram. **n**–**s**, Distributions of (**n**) CTCF^105^, (**o**) H3K27ac^91^, (**p**) H4K16ac^91^, (**q**) H3K79me2 (ref. ^91^), (**r**) H3K36me3 (ref. ^91^), and (**s**) H3K9me3 (ref. ^91^) relative to the closest boundary of an H3.3 domain. Positive and negative values indicate positions inside and outside H3.3 domains, respectively. The pink line shows a KDE overlaid on the histogram. Source data

The finding that cohesin constrains euchromatic nucleosome motion motivated us to explore how cohesin affects the structure and physical properties of euchromatic domains. For this purpose, we used 3D-SIM^7,46–48^ on HCT116 cells expressing H3.3-Halo and visualized euchromatic domain structures in live or formaldehyde (FA)-fixed cells at super-resolution. The 3D-SIM enables fast multicolor 3D acquisition of whole cells with higher resolution (*xy* = ~130 nm; *z* = ~250 nm) and strongly enhanced contrast compared to conventional fluorescence microscopy (Fig. 5a(i),(ii),(iv),(v) and Supplementary Video 4)^7,47^. Using quality-controlled 3D-SIM (Extended Data Fig. 8a–e; Methods)^79^, we found that euchromatin in live HCT116 cells appeared as connected, condensed chromatin domains with irregular shapes and diameters in the range of a few hundred nanometers (Fig. 5b and Supplementary Video 5). A similar architecture was observed in FA-fixed cells labeled with H3.3-Halo (Fig. 5a(v)).

Furthermore, we used stochastic optical reconstruction microscopy (STORM) imaging of H3.3-Halo-HMSiR in HCT116 cells, another super-resolution method with ~5 nm resolution (Extended Data Fig. 8f)^5,80,81^, to investigate euchromatic domain organization. Reconstructed STORM images revealed condensed euchromatin domains (Fig. 5f) similar to those observed by 3D-SIM. Clustering analysis of nucleosomes using SR-Tesseler-DBSCAN pipeline^82^ yielded a peak domain radius of ~100–120 nm (diameter of ~200 to 240 nm; Fig. 5f). These findings of condensed euchromatic domains in human cells challenge the established view of euchromatin as largely open (Extended Data Fig. 8g,h).

### Preferential localization of cohesin to euchromatin domains

We investigated the spatial distributions of cohesin and active transcription sites within the nucleus by combining 3D-SIM with immunostaining. RAD21 immunostaining revealed that cohesin appeared as punctate foci in SIM images (Fig. 5a(vii)), likely representing chromatin-bound cohesin. The centroids of these foci were frequently located near the edges of H3.3-Halo domains, suggesting that cohesin localizes to the surfaces of euchromatin domains (enlarged images in Fig. 5a). Classification analysis of signal density in 3D-SIM (Fig. 5c–e)^7,83^ confirmed that cohesin is enriched in high-density H3.3 regions (Fig. 5h, classes 3–7, and Extended Data Fig. 8i), indicating a preferential localization of cohesin to euchromatin domains. Consistent with this, cohesin enrichment was observed in low-density DAPI regions (classes 1 and 2) and depleted in denser regions (Extended Data Fig. 8j), in good agreement with previous observations^7^. This spatial distribution of cohesin also supports our finding that euchromatic nucleosome motion is more sensitive to cohesin depletion (Extended Data Fig. 7a,b).

### Active RNA polymerase II (RNAP II) localizes around euchromatic domain surfaces

We then examined the localization of active transcription sites. We performed immunostaining using antibodies against the C-terminal domain (CTD) of RNAP II and its phosphorylated forms at serine 5 (Ser5P) and serine 2 (Ser2P) to visualize RNAP II signals composed predominantly of inactive, active paused, and elongating RNAP II, respectively (Fig. 5g). All signals appeared as punctate foci (Fig. 5g). These signals were enriched in DAPI-poor regions (Extended Data Fig. 8k, classes 1 and 2) and in H3.3-Halo-rich regions (Extended Data Fig. 8l, classes 4–7), indicating a euchromatin-biased localization.

We observed distinct differences in nuclear localization among cohesin and RNAP II forms. Cohesin showed a relatively uniform distribution with a preference for euchromatin (Fig. 5h). In contrast, RNAP II showed increased localization to H3.3-denser areas upon transcriptional activation (Fig. 5i–k). Within the H3.3-Halo classification, CTD signals peaked at classes 4 and 5 (Fig. 5i), Ser5P at class 5 (Fig. 5j) and Ser2P at classes 6 and 7 (Fig. 5k). These patterns suggest that inactive RNAP II remains at the periphery (or outside) of euchromatic domains, paused active RNAP II localizes near the domain surface and elongating RNAP II may penetrate the domain.

**Fig. 6:**
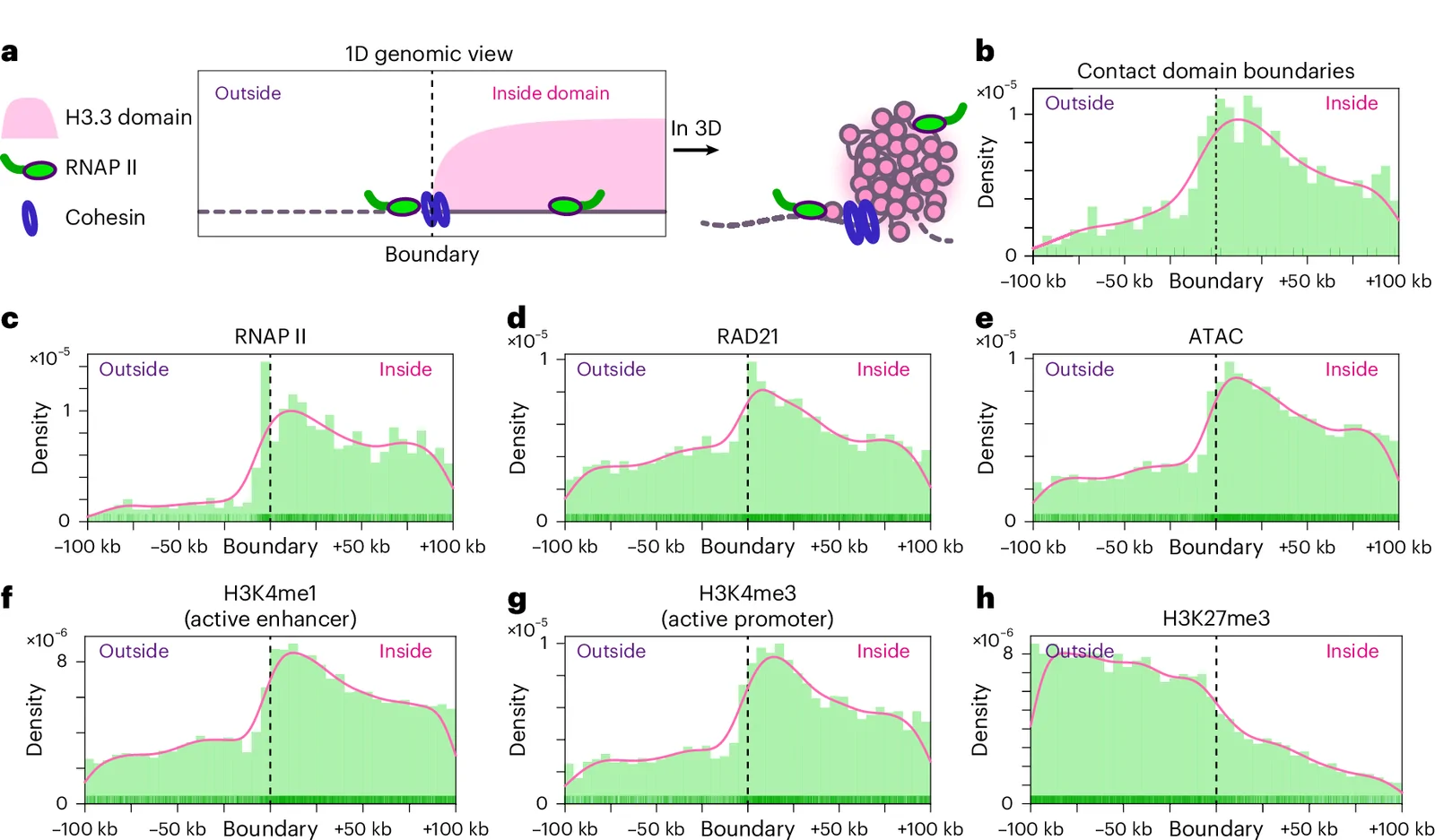
RNAP II and cohesin localize around the boundary, which is the surface of condensed euchromatic domains. **a**, Relative position of RNAP II, cohesin or other markers in the boundary of H3.3 domains (median = 210 kb; Extended Data Fig. 8m) is examined. The H3.3 domain is detected from the pulldown experiment (Fig. 4). Molecules around the boundary of an H3.3 domain in 1D will be localized on the surface of a condensed euchromatic chromatin domain in 3D. **b**–**h**, Distribution of contact domain (or TAD) boundaries^91^ (**b**), RNAP II^105^ (**c**), RAD21 (ref. ^105^; **d**), ATAC–seq peaks (**e**), H3K4me1 (ref. ^91^; **f**), H3K4me3 (ref. ^91^; **g**) and H3K27me3 (ref. ^91^; **h**) relative to the closest boundary of an H3.3 domain. Positive and negative values indicate inside and outside of H3.3 domains, respectively. The pink line is a kernel density estimation on the distribution histogram. See also Extended Data Fig. 8n–s for other markers. 1D, one dimensional. Source data

Furthermore, we investigated the relative genomic locations of RNAP II, cohesin and other histone marks to H3.3-Halo domains (Fig. 6 and Extended Data Fig. 8m–s). Consistent with the 3D-SIM imaging data, RNAP II, cohesin and active enhancers/promoters are preferentially located around the boundary of the H3.3 domain, which corresponds to the surface of condensed euchromatic domains in 3D (Fig. 6a).

### Euchromatin domains retain compaction after cohesin loss

**Fig. 7:**
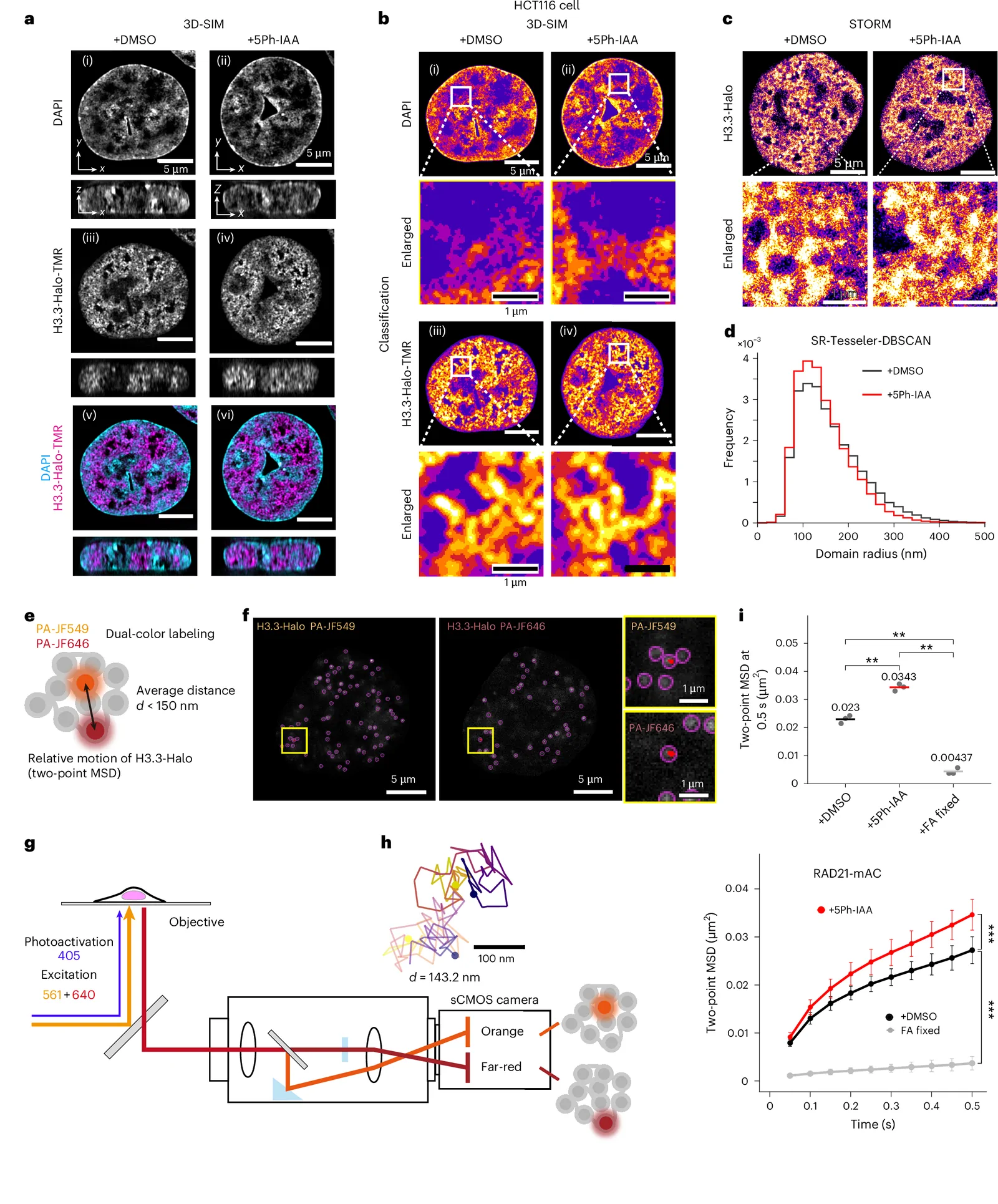
3D-SIM and STORM imaging and two-point MSD analysis of condensed euchromatic domains. **a**, Representative single *z* slices of 3D-SIM images on chromatin labeled with DAPI ((i) and (ii)) or H3.3-Halo-TMR ((iii) and (iv)) with DMSO treatment ((i) and (iii)) or cohesin depletion ((ii) and (iv)). (v) and (vi) are merged images. Their *z*–*x* views are also shown below each image. **b**, Heatmaps of DAPI or H3.3-Halo-TMR compaction proxies, based on the intensity classification into seven classes. (i)–(iv) correspond to the same panel number in **a**. Enlarged images from the corresponding squared regions in (i)–(iv) are also shown below each panel. **c**, Representative STORM reconstructed view of FA-fixed HCT116 RAD21-mAC H3.3-Halo cells. Chromatin was labeled with H3.3-Halo-HMSiR with DMSO treatment (+DMSO) or cohesin depletion (+5Ph-IAA). Bottom: enlarged views of the boxed regions in the top panels. Euchromatin is condensed. **d**, Histogram of estimated radius of euchromatic domains by SR-Tesseler-DBSCAN pipeline (Methods). **e**, Scheme for dual-color imaging of H3.3-Halo and two-point MSD. **f**, A single-nucleosome (H3.3-Halo-PA-JF dyes) image of a living HCT116 nucleus after background subtraction. Magenta circles highlight the tracked dots. A pair of dots with an average interpoint distance *d* < 150 nm (square) is shown in the zoomed-in panel with red trajectories. The experiment was independently repeated at least thrice with similar results. **g**, Schematic for dual-color imaging with a beam splitter system (W-VIEW GEMINI, Hamamatsu Photonics). The images of two single nucleosomes with different colors were acquired with a single sCMOS camera (top half, orange; bottom half, far-red). **h**, Representative pair trajectories with average distance *d* < 150 nm. Darker color indicates PA-JF549-labeled H3.3-Halo; lighter color indicates PA-JF646-labeled H3.3-Halo. Trajectories are shown with a yellow-to-navy gradation from starting to ending points. **i**, Bottom: two-point MSD plots (*d* < 150 nm; ±s.d. among cells) of H3.3-Halo in HCT116 cells with indicated conditions—DMSO (black, *n* = 20 cells), 5Ph-IAA (ΔRAD21, red, *n* = 20 cells) and FA fixed (gray, *n* = 25 cells). ***P < 0.001 by two-sided two-sample Kolmogorov–Smirnov tests at 0.5 s (Holm-adjusted *P* = 1.43 × 10^−7^ for DMSO versus 5Ph-IAA and *P* = 1.26 × 10^−12^ for DMSO versus FA fixed). Top: replicate-aware quantification of two-point MSD at 0.5 s. Each dot represents an independent biological replicate (*n* = 3). Bars indicate the mean. Statistical significance across biological replicates was assessed using a paired two-sided *t* test. **P < 0.01. Statistical details are provided in Supplementary Table 1. Source data

**Extended Data Fig. 9:**
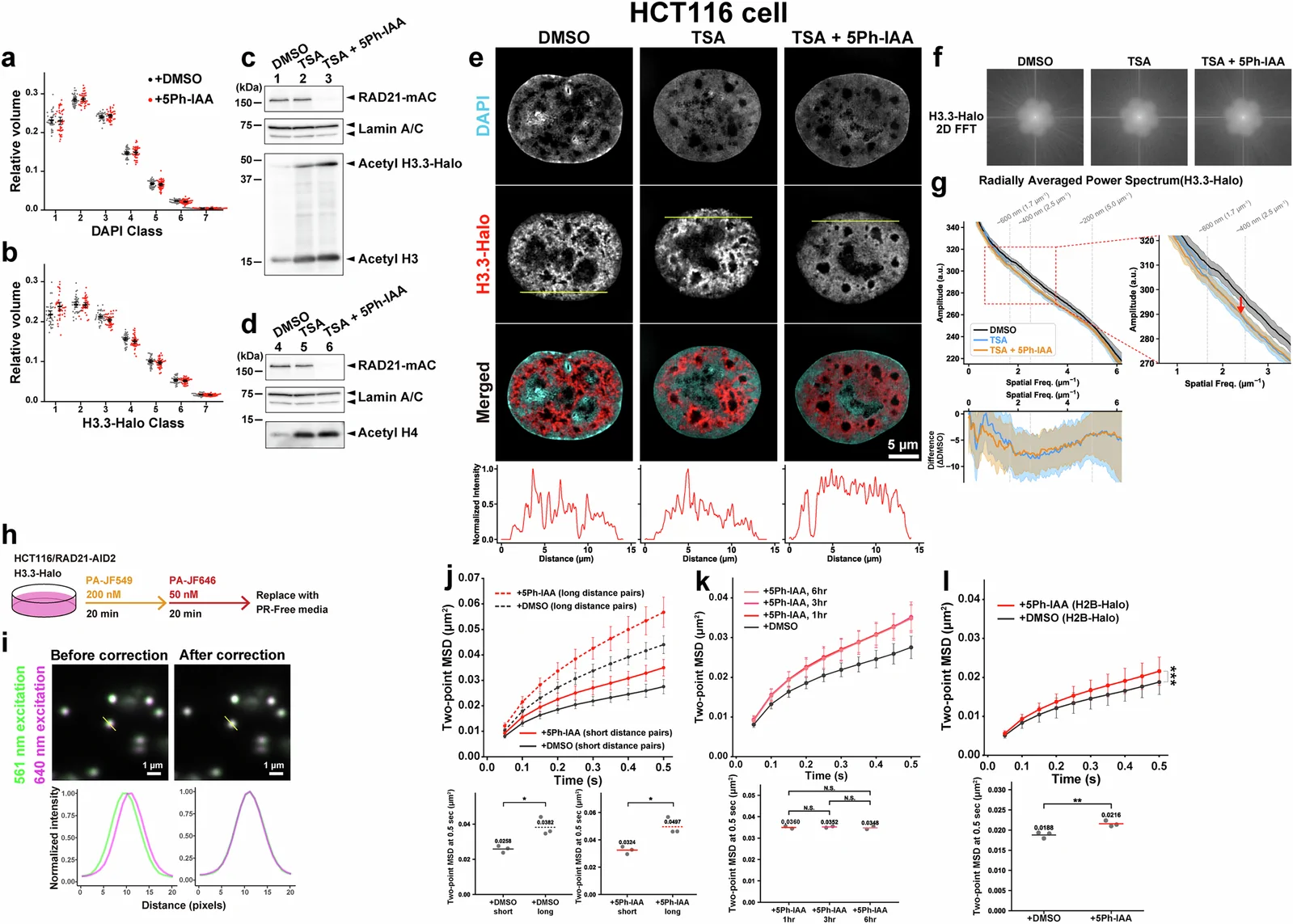
‘Melting’ of chromatin domains and dual-color single-nucleosome imaging. **a**,**b**, Proportion of nuclear volume for each class of DAPI (**a**) and H3.3-Halo-TMR (**b**), showing no significant change after cohesin depletion (mean ± 95% CI). Each dot represents a single nucleus; DMSO, n = 35 cells; 5Ph-IAA, n = 39 cells. **c**,**d**, Immunoblotting of HCT116 RAD21-mAID-mClover cells treated with DMSO, TSA, or TSA + 5Ph-IAA to verify RAD21 depletion and histone hyperacetylation. **e**, Representative single z-slices of 3D-SIM images of DAPI- or H3.3-Halo-TMR-labeled chromatin after DMSO, TSA, or TSA + 5Ph-IAA treatment. Bottom, H3.3-Halo-TMR intensity line profiles. TSA treatment with cohesin depletion causes ‘melting’ of H3.3-Halo chromatin domains. **f**, Representative 2D FFT images of H3.3-Halo-TMR images shown in **e**. **g**, Radially averaged power spectrum of H3.3-Halo-TMR images; shaded regions represent s.d. among cells (DMSO, n = 13 cells; TSA, n = 6 cells; TSA + 5Ph-IAA, n = 10 cells). The decrease in the 300–600 nm range indicates domain-scale melting. **h**, Dual-color labeling strategy. PR, phenol red. **i**, Chromatic aberration correction using interchannel shifts determined from fluorescent beads. Transformation parameters were obtained with the ImageJ plugin ‘descriptor-based registration (2D/3D)’ and applied to trajectory data; representative images before and after correction are shown. **j**–**l**, Two-point MSD plots (mean ± s.d. among cells) and replicate-aware quantification of MSD at 0.5 s. Dots indicate independent biological replicates (n = 2 or 3), bars indicate mean, paired two-sided *t*-test; NS, not significant; *P < 0.05, **P < 0.01. **j**, H3.3-Halo pairs at short distance (d < 150 nm) or long distance (300 nm < d < 1000 nm) in DMSO or 5Ph-IAA-treated cells (n = 20 cells each). Short-distance pairs represent neighboring nucleosome pairs, whereas long-distance pairs represent unrelated pairs. **k**, Short-distance H3.3-Halo pairs after DMSO or 5Ph-IAA for 1, 3, or 6 h (n = 20 cells each). **l**, Short-distance H2B-Halo pairs after DMSO or 5Ph-IAA (n = 20 cells each); ***P = 2.70 × 10^−4^ by two-sided Kolmogorov–Smirnov test. Statistical details are provided in Supplementary Table 1. Source data

To assess the effect of cohesin depletion on chromatin organization, especially euchromatic domains, we acutely depleted RAD21 in HCT116 cells expressing H3.3-Halo and visualized their chromatin organization using 3D-SIM. Interestingly, no obvious qualitative changes were observed in the overall chromatin organization (Fig. 7a, DAPI, and Supplementary Videos 6 and 7) or in the euchromatic domains (H3.3-Halo; Fig. 7a) upon cohesin depletion. For quantitative analysis, we classified DAPI and H3.3-Halo signals. Cohesin depletion did not change the relative volumes of each class, that is, the compaction levels of DAPI-stained chromatin (Fig. 7b and Extended Data Fig. 9a) and H3.3-Halo-labeled euchromatin domains (Fig. 7b and Extended Data Fig. 9b), in agreement with previous findings^7^.

Reconstructed STORM images after cohesin depletion showed a similar condensed domain organization (Fig. 7c). SR-Tesseler-DBSCAN analysis^82^ of the reconstructed images further reveals that cohesin depletion slightly increased the fraction of domains with smaller radii (~100 to 120 nm) and decreased the large-radius tail (~200 to 400 nm; Fig. 7d), consistent with domain splitting upon cohesin loss^8,22^. The modal radius was largely maintained. Together, these super-resolution results indicate that cohesin depletion retains overall domain compaction while increasing nucleosome mobility.

### Nucleosome interactions help condense euchromatin domains

To investigate which factors condense euchromatic domains, we treated the cells with the histone deacetylase inhibitor trichostatin A (TSA)^84^, which induces hyperacetylation of histone tails (Extended Data Fig. 9c,d)^85^. Tail hyperacetylation weakens local nucleosome–nucleosome interactions^86^ and decondenses chromatin^5,87,88^. Indeed, mitotic chromosomes were highly decondensed and expanded in volume in condensin-depleted and TSA-treated cells^89,90^. After treatment with TSA for 7 h and acute depletion of RAD21 (Extended Data Fig. 9c,d), apparent decondensation of H3.3-Halo domains was observed by 3D-SIM (Extended Data Fig. 9e). Power-spectrum analysis showed that the amplitude at spatial scales of ~300 to 600 nm (domain scale) decreased substantially (Extended Data Fig. 9f,g). Together, these results indicate that electrostatic interactions between positively charged histone tails and DNA/histones, together with cohesin, contribute to condensed domain formation. Moreover, these results show that our 3D-SIM was able to detect chromatin decondensation and confirm that the condensed euchromatic domains were not an imaging artifact. Our observations provide compelling evidence that challenges the established view of euchromatin as largely open (Extended Data Fig. 8g,h).

### Cohesin regulates nucleosome-level fluidity in euchromatin

Although euchromatic nucleosome movement increases upon cohesin depletion, euchromatic domain compaction remains largely unchanged. This raised the question of whether nucleosomes gain mobility within the domains. To address this, we introduced dual-color labeling (Fig. 7e and Extended Data Fig. 9h,i) and tracking of two spatially neighboring single H3.3-Halo nucleosomes (Fig. 7f–h and Supplementary Video 8). Because simple single-nucleosome imaging cannot distinguish whether individual nucleosomes fluctuate within the domain or whether the domain itself moves, we measured the distances between two neighboring H3.3-Halo nucleosomes within euchromatic domains or possibly across adjacent domains (average distance *d* < 150 nm; Fig. 7e)^8^ and obtained the MSD of their relative positions (two-point MSD)^8,30,42^. It should also be noted that two-point MSD provides not only the magnitude of local nucleosome motion but also the extent of local intermingling across nearby nucleosomes. As a control, we analyzed two-point MSD for unrelated (long-distance) H3.3-Halo pairs (300 nm < *d* < 1,000 nm; Extended Data Fig. 9j).

Two-point MSD analysis showed much greater motion between neighboring H3.3-Halo nucleosomes in living cells than in FA-fixed cells (Fig. 7i), suggesting that nucleosomes fluctuate within the domain. Notably, two-point MSD significantly increased upon acute cohesin depletion (1 h 5Ph-IAA) across biological replicates (Fig. 7i and Extended Data Fig. 9j–l). Longer depletions (3 h or 6 h 5Ph-IAA) did not further change the two-point MSD (Extended Data Fig. 9k). These results indicate that cohesin depletion increases nucleosome mobility within condensed euchromatic domains and local intermingling across nearby nucleosomes. These results also suggest that cohesin depletion induces a transition to a more liquid-like state of chromatin, or to an unrestricted state of the chromatin polymer.

Two-point MSD for unrelated (long-distance) control pairs was approximately twice the one-point MSD at matched lags (Extended Data Fig. 9j), as theory predicts for uncorrelated motion. By contrast, neighboring pairs showed lower two-point MSD, consistent with positively correlated motion within domains. In both classes, RAD21 depletion increased two-point MSD. Taken together, neighboring-pair two-point MSD reveals the extent of local physical constraints within euchromatic domains.

### Local domain mixing upon cohesin depletion

**Fig. 8:**
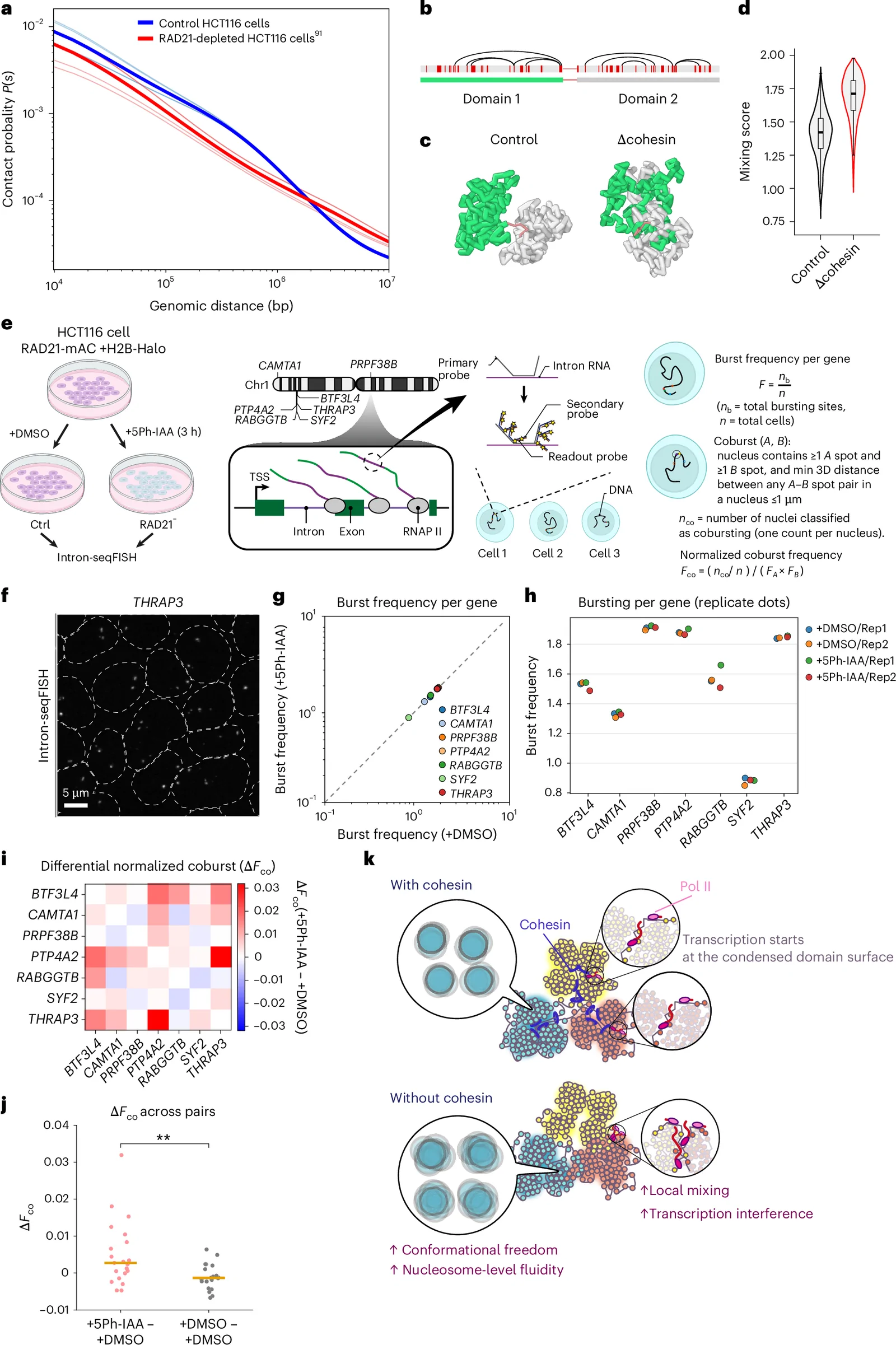
Cohesin prevents local mixing of condensed euchromatic domains. **a**, Hi–C contact probability *P*(*s*) for HCT116 cells with cohesin (control, blue) and after RAD21 depletion (red) was plotted from the Hi–C data in ref. ^91^. Upon RAD21 depletion, short-range (up to ~1.5 Mb) contacts decrease, whereas long-range (several Mb scale) contacts increase, consistent with local mixing of chromatin domains at multimegabase scales after cohesin depletion. **b**, An example chain composed of two 200-kb domains, domain 1 (green) and domain 2 (gray), connected by a short linker. The regions containing acetylated nucleosomes are colored red. In the control case, five cohesin molecules are loaded to form five loops within each domain, represented by arcs. **c**, Snapshots of the configurations of the two consecutive domains (green and gray). Cases with cohesin (control) and without cohesin (∆cohesin). **d**, Distributions of the mixing score (Supplementary Methods). Each distribution comprises *n* = 10,010 time-resolved values nested within ten independent simulation trajectories per condition. Violin widths indicate kernel density; centerlines indicate medians, boxes indicate the 25th–75th percentiles and whiskers extend up to 1.5× IQR (interquartile range) from the box limits. Boxes show the median and the 25–75% width. **e**, Schematic of the intron-seqFISH workflow and the definitions of burst and proximal coburst metrics used in this study. HCT116 RAD21-mAC + H2B-Halo cells were treated with DMSO or 1 µM 5Ph-IAA for 3 h and then subjected to intron-seqFISH. Intronic nascent transcripts from seven genes located on chromosome 1 (*CAMTA1*, *PTP4A2*, *RABGGTB*, *SYF2*, *THRAP3*, *BTF3L4* and *PRPF38B*) were detected (Extended Data Fig. 10g,h). TSS, transcription start site. **f**, Representative image obtained by intron-seqFISH in fixed HCT116 RAD21-mAC H2B-Halo cells. In each hybridization round, nascent intronic RNA from a single target gene was visualized (*THRAP3* for this image). Dashed lines indicate nuclear boundaries determined from DAPI images. **g**, Burst frequency of individual genes before and after RAD21 depletion. **h**, Burst frequency per gene shown for each biological replicate. **i**, Heatmap of differential normalized proximal cobursting (Δ*F*_co_) for all gene pairs. **j**, Distribution of Δ*F*_co_ across all gene pairs. Δ*F*_co_ values comparing RAD21^−^ and control conditions (left) are shown alongside control replicate differences (ctrl/Rep2 − ctrl/Rep1; right) as a baseline. Each dot represents a gene pair (*n* = 21). Bars indicate the median. **P < 0.01 (*P* = 0.008) by two-sided Wilcoxon rank-sum test. **k**, Cohesin holds chromatin fibers together and constrains chromatin motion within condensed euchromatic domains. While cohesin depletion does not alter the compaction level of these domains, it increases nucleosome-level fluidity (conformational freedom). This enhanced fluidity upon cohesin loss may promote mixing across chromatin domains. Thus, cohesin stiffens condensed euchromatic domains, preventing domain intermingling and ensuring effective insulation of transcriptional programs. Source data

**Extended Data Fig. 10:**
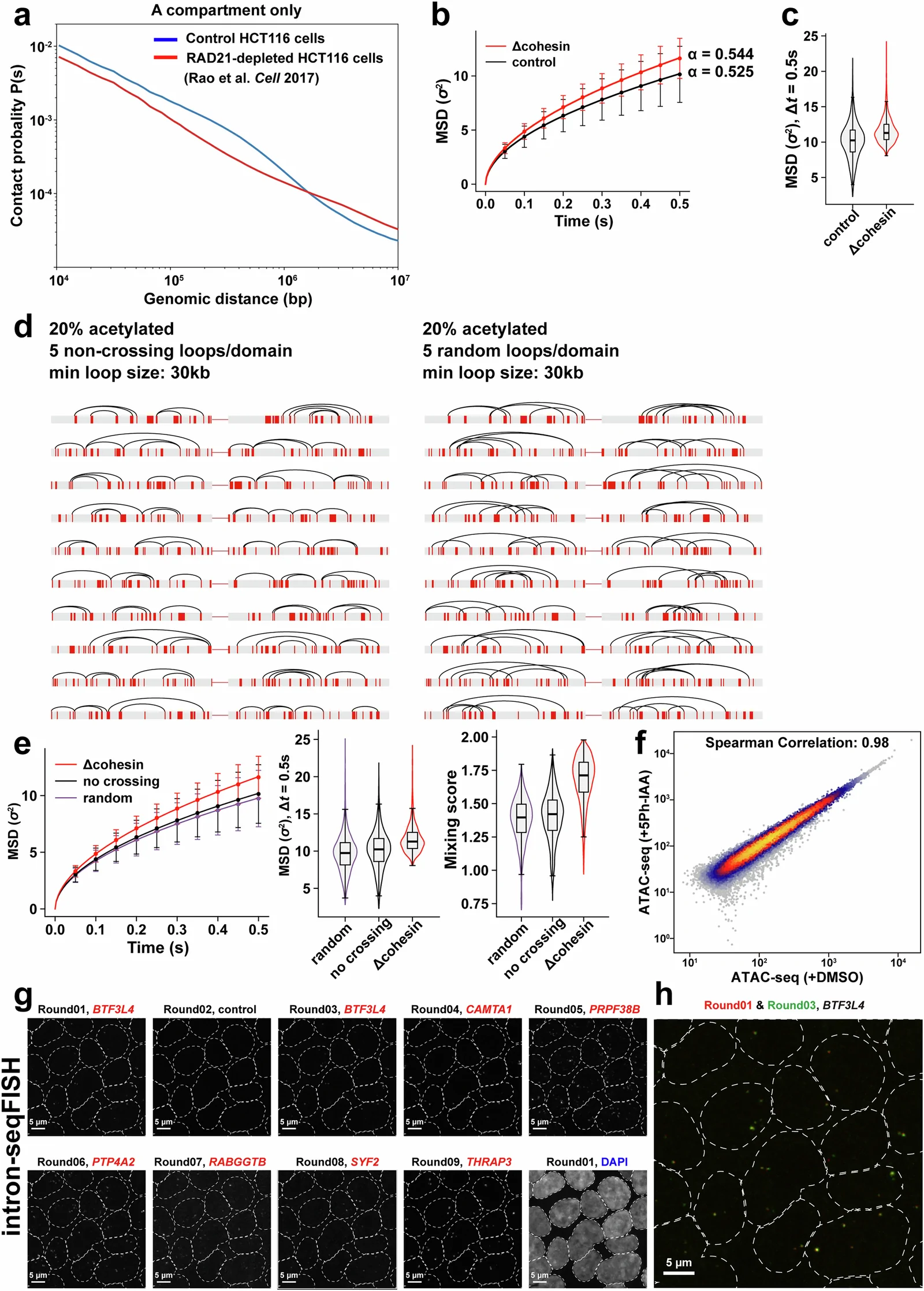
Local domain mixing by cohesin depletion. **a**, Hi–C contact probability P(s) in A-compartment regions of HCT116 cells with cohesin (control, blue) or after RAD21 depletion (red), was plotted from the Hi–C data in ref. ^91^. RAD21 depletion decreased short-range contacts up to ~1.5 Mb and increased long-range contacts at the several-Mb scale. **b**, Temporal MSD variation of simulated 1-kb beads with or without cohesin; lines and error bars indicate mean ± s.d. Data were derived from 10 trajectories, each spanning 50 s (5 × 10^6^ simulation steps) for each condition. **c**, Distributions of MSD of simulated 1-kb beads at Δ*t* = 0.5 s. Boxes show the median and 25–75% width. **d**, Simulation chains with different acetylation and loop patterns. Randomly generated acetylated regions (φ=20%) are shown in red. Five loops connecting 10 sites in acetylated regions were generated with a minimum loop length of 30 kb, either without crossing (left) or allowing crossing (right). **e**, Simulated chromatin movement. Left, temporal MSD of 1-kb beads; middle, MSD distributions; right, mixing-score distributions for two consecutive domains. The control model in Fig. 8c,d and panels **b** and **c** corresponds to the no-crossing model in **d**. After cohesin depletion, the average bead MSD and mixing score increased. For **b**, **c** and **e**, ten independent simulation trajectories were analyzed per condition, each generated from a chain with a different acetylation and loop pattern. MSD analyses used n = 4,050 bead-level values per condition (405 beads in each of 10 trajectories). Mixing-score distributions used n = 10,010 time-resolved values per scenario, nested within the 10 simulation trajectories. For violin plots, widths indicate kernel density estimates, centerlines indicate medians, box limits indicate the 25th and 75th percentiles, and whiskers extend up to 1.5×IQR (interquartile range) from the box limits. **f**, Genome-wide comparison of ATAC-seq signals in DMSO- or 5Ph-IAA-treated HCT116 RAD21-mAC cells. **g**, Representative maximum-intensity-projection images from intron-seqFISH in fixed HCT116 RAD21-mAC cells. Each round detects nascent intronic RNA from one target gene; dashed lines indicate DAPI-defined nuclear boundaries. The experiment was independently repeated twice with similar results. **h**, Overlay of the same gene detected in two independent rounds, showing round-to-round alignment and reproducibility. Source data

As described above, cohesin loss increased nucleosome-level fluidity within condensed euchromatic domains. In this state, domains may become less constrained and more flexible, allowing interdomain mixing. Consistent with this, Hi–C contact probability plot in HCT116 cells^91^ shows that cohesin loss decreases short-range contacts (~0.1 to 1.5 Mb, TAD scale) and increases long-range contacts (Mb scale; Fig. 8a). This effect is slightly enhanced within A compartment regions (Extended Data Fig. 10a). These findings provide genomic evidence of ‘domain mixing’ upon cohesin depletion and align with previous studies^21,92^.

Furthermore, we used a polymer model of chromatin chains to analyze the effects of cohesin depletion on chromatin domain conformation. The model has 1-kb resolution and consists of two domains, each 200 kb long, connected by a short linker (Fig. 8b,c, control). In the simulations, domains appeared more mixed when cohesin was depleted, as quantified by the mixing score (Supplementary Methods, Fig. 8c,d and Supplementary Video 9). The polymer simulations also showed increased bead MSD with cohesin depletion, consistent with the experimental data (Extended Data Fig. 10b–e). These results suggest that local domain mixing upon cohesin depletion could weaken insulation across neighboring transcriptional environments.

Because acute cohesin loss often produces only modest changes in bulk gene expression^91^ and in ATAC–seq signals in HCT116 cells (Extended Data Fig. 10f), we reasoned that transcriptional bursting—particularly its *cis*-coordination between loci—provides a more sensitive functional readout. We therefore quantified this *cis*-coordination of transcriptional bursting in HCT116 using intron-seqFISH (intron sequential fluorescence in situ hybridization; Fig. 8e,f and Extended Data Fig. 10g,h)^93,94^. Here cobursting was defined only when at least one *A* spot and one *B* spot were detected in the same nucleus and the minimum 3D distance between the *A*–*B* spot pair was ≤1 µm, thereby focusing the analysis on spatially proximal cobursting events (Supplementary Methods). We selected seven genes on chromosome 1 whose per-gene burst frequency changed minimally upon RAD21 depletion (Fig. 8g,h and Supplementary Methods), enabling us to assess changes in cobursting independently of large shifts in single-gene activity. Notably, the proximal coburst frequency of gene pairs significantly increased upon cohesin depletion (Fig. 8i,j), indicating increased *cis* burst coupling among spatially proximal gene pairs, consistent with a previous study in mouse embryonic stem cells^21^. Thus, cohesin depletion increases burst coupling among spatially proximal genes even when per-gene bursting remains largely unchanged, consistent with weakened local transcriptional insulation (Fig. 8k).

## Discussion

In this study, we used a combined approach of single-nucleosome imaging/tracking and super-resolution imaging to explore how cohesin regulates euchromatic chromatin organization in living human cells. Our approach provides a more comprehensive view of both global and local changes in chromatin dynamics and organization than previous studies that primarily focused on specific chromatin loci^42,43^. Our results show that cohesin constrains local chromatin motion mainly through loop formation, particularly in euchromatin, rather than through sister chromatid cohesion (Fig. 2). Only minimal constraint effects arise from cohesion sites (Fig. 2), presumably because only a limited number of sister regions are aligned in human interphase cells^95,96^. These findings align with previous studies reporting increased chromatin motion upon cohesin depletion^5,42,43^. Furthermore, our results using head-tethered cohesin suggest that cohesin constrains euchromatin loop domains without requiring active loop extrusion (Fig. 3g–i).

To label euchromatin specifically, we developed euchromatin-specific labeling using histone H3.3-Halo (Fig. 4). The labeled genomic regions correspond to the Hi–C A compartment^17^ with active histone marks in our cells (Fig. 4). Combining this euchromatin labeling with 3D-SIM^7,47,48^ and STORM^80,81^ enabled super-resolution imaging of euchromatic domains in live and fixed HCT116 cells, revealing their physically condensed nature. Nucleosome–nucleosome interactions and cohesin seem to drive the formation of condensed euchromatic domains. Notably, a similar principle applies to condensed mitotic chromosomes, which are organized by condensins and nucleosome–nucleosome interactions^89,90^. Our findings provide critical evidence that challenges the established notion of euchromatin as largely open.

Physically condensed euchromatic domains can provide higher-order regulation of various DNA transactions, including transcription, whereas extended fiber loops cannot (Extended Data Fig. 8g,h). If loop domains were extended or open, unnecessary interactions between domains and misregulation of transcription could occur (Extended Data Fig. 8g). Condensed domains, per se, help avoid transcriptional interference and ensure proper gene regulation (Extended Data Fig. 8h). In addition, condensed euchromatic domains likely hinder the access of transcriptional protein complexes to their target sequences, such as promoters or enhancers located in the inner core of chromatin domains^7^. These findings support the notion of higher-order gene regulation within a domain, such as a ‘buoy’ model^24,97^. Partial decondensation of such condensed domains through histone modifications or nucleosome eviction may also serve as a regulatory mechanism to activate gene transcription^98^. Furthermore, condensed domains are more resistant to radiation than decondensed forms, presumably because condensed chromatin generates lower levels of reactive radicals^99,100^. This feature is especially important for maintaining the integrity of actively transcribed euchromatic regions, which are directly connected to essential genome functions. Together, these three features highlight the biological relevance of condensed euchromatin.

While previous studies suggested that cohesin is involved in long-distance promoter–enhancer interactions, particularly those spanning domain boundaries^101,102^, our findings highlight a previously underappreciated role of cohesin in constraining the conformational freedom of condensed euchromatic domains (Fig. 8k). We revealed how these physical properties of euchromatin domains are regulated by cohesin, and how they are coupled to genome organization and function. Super-resolution imaging of euchromatic domains and two-point MSD measurements across neighboring euchromatic nucleosomes show that, while cohesin depletion does not alter the overall compaction level of euchromatin domains, it increases nucleosome-level fluidity within these domains (Fig. 7i), leading to mixing of chromatin domains (Fig. 8b–d,k). Indeed, Hi–C contact probability plot in HCT116 cells^91^ shows that cohesin loss decreases short-range contacts (~0.1–1.5 Mb, TAD scale) and increases long-range contacts (Mb scale; Fig. 8a). Co-activation of proximal gene transcription was also observed upon cohesin loss (Fig. 8i,j)^21^. Consistent with this, it was reported that cohesin loss increases ‘entropy’ in genome folding and variation in gene expression^92^. Together, domain mixing upon cohesin loss is essentially another readout of weakened insulation across domains. Condensed euchromatic domains constrained by cohesin contribute to effective insulation^52,53^ of transcriptional programs across domains.

### Limitations of the study

Our experiments were performed in somatic human cells (HCT116 and HeLa), where H3.3 incorporation predominantly marks euchromatin. H3.3 has also been reported in the telomeric region^50^, or in facultative heterochromatin in specific contexts, such as embryonic stem cells and during cell-fate transitions^103,104^. The degree of condensation may also differ across cell types. Exploring these contexts will be an interesting next step.

All SIM and STORM analyses were performed on asynchronous populations of HCT116 cells. While cohesin localization patterns and H3.3 distribution did not show noticeable variation across interphase cells, this remains an interesting question for future investigation, as genomic DNA content doubles and nuclear volume increases during cell-cycle progression^45^.

Our data support the idea that cohesin physically constrains condensed euchromatic domains through loop formation. However, the precise physical mechanism by which cohesin constrains chromatin remains unclear and will require further investigation.

Finally, the Hi–C and ChIP–seq evidence discussed here was integrated from previously published datasets^91,105^ rather than generated within the same experimental pipeline as our imaging analyses. Therefore, potential differences in experimental conditions and data processing should be considered, and these cross-platform comparisons are interpreted here as orthogonal support.

## Methods

All experiments complied with relevant ethical regulations. This study used established human cell lines and involved no human participants, human samples or animals; therefore, no human or animal ethics approval was required. Recombinant DNA experiments were approved by the Institutional Recombinant DNA Experiment Safety Committee of the National Institute of Genetics (R7-7). Additional experimental details are provided in Supplementary Methods.

### Cell lines and culture conditions

HeLa S3 cells^108^ and HT1080 cells with *lacO*/EGFP-LacI and *tetO*/TetR-4×mCherry (a clone of TT75, TT165, kindly provided by T. Tanaka, University of Dundee)^62^ were cultured at 37 °C in 5% CO_2_ in DMEM (D5796-500ML, Sigma-Aldrich) supplemented with 10% FBS (FB-1061/500, Biosera). All HCT116 cells (CCL-247, ATCC) with AID2 for rapid depletion^55^ were cultured at 37 °C in 5% CO_2_ in McCoy’s 5 A medium (SH30200.01, HyClone) supplemented with 10% FBS.

### Target protein depletion by AID2

To rapidly degrade RAD21-mAC, CTCF-mAC and WAPL-mAC, HCT116 cells expressing OsTIR1(F74G) were treated with 1 µM 5Ph-IAA for 1 h (RAD21), 2 h (CTCF) or 4 h (WAPL), respectively, except where noted otherwise^55,67^. Control cells were treated with 0.1% dimethyl sulfoxide (D2650-5X5ML, Sigma-Aldrich). Degradation of the target proteins was confirmed by the loss of mClover fluorescence. After treatment, cells were fixed, permeabilized and stained with DAPI as described in the Supplementary Methods—‘Expression and localization of H2B-HaloTag or H3.3-HaloTag’. *Z*-stack images (30 sections at 0.2 µm intervals along the *z* axis) were acquired using a DeltaVision Ultra microscope (Applied Precision) equipped with an Olympus PlanApoN ×60 objective lens (NA 1.42). Nuclear mClover fluorescence intensity was quantified using Fiji, with background signal subtracted.

RAD21-mAC, WAPL-mAC or CTCF-mAC degradation was also confirmed by western blotting. The procedure was the same as described in the Supplementary Methods—‘Expression and localization of H2B-HaloTag or H3.3-HaloTag’. Membranes were probed with mouse anti-RAD21 (1:1,000; 05-908, Upstate), rabbit anti-WAPL (1:1,000; A301-779A-T), rabbit anti-CTCF (1:1,000; 10915-1-AP, PGI Proteintech) and mouse anti-mAID (1:1,000; M214-3, MBL) antibodies, followed by horseradish peroxidase-conjugated goat antimouse secondary antibody (1:5,000; 170-6516, Bio-Rad), or antirabbit IgG DyLight 800 (1:5,000; SA5-35571, Invitrogen). As a loading control, a goat anti-GAPDH antibody (1:2,500; 12004158, Bio-Rad; StarBright Blue700 conjugated) was used.

### Single-nucleosome imaging

Established cell lines were cultured on poly-L-lysine-coated glass-based dishes (3970-035, Iwaki). H2B-Halo or H3.3-Halo incorporated into nucleosomes was fluorescently labeled with 80 pM HaloTag TMR ligand for 20 min at 37 °C in 5% CO_2_, washed thrice with 1× HBSS (H1387, Sigma-Aldrich) and then incubated in the following media overnight before single-nucleosome imaging. HeLa S3 cells were observed in DMEM (21063-029, Thermo Fisher Scientific), and HCT116 cells in McCoy’s 5 A (1-18F23-1, BioConcept). These media were phenol red (PR) free and supplemented with 10% FBS. To increase the number of tracked nucleosomes when applying the RL algorithm for motion classification, H2B-Halo was labeled with 50 nM PA-JF646 (provided by the Lavis Lab, Janelia Research Campus)^75^ overnight using the same labeling procedure.

A live-cell chamber (INU-TIZ-F1, Tokai Hit) and digital gas mixer (GM-8000, Tokai Hit) were used to maintain cell culture conditions (37 °C, 5% CO_2_ and humidity) during microscopy. Single nucleosomes were observed using an inverted Nikon Eclipse Ti microscope equipped with a 100-mW Sapphire 561-nm laser (Coherent) and an sCMOS ORCA-Flash 4.0 or ORCA-Fusion BT camera (Hamamatsu Photonics). Live cells labeled with TMR were excited with the 561-nm laser through an objective lens (×100 PlanApo TIRF, NA 1.49; Nikon) and detected at 575–710 nm. An oblique illumination system with a TIRF unit (Nikon) was used to excite fluorescent nucleosome molecules within a thin area of the cell nucleus and reduce background noise (Fig. 1a). Sequential image frames were acquired using NIS-Elements (Nikon) at a frame rate of 50 ms under continuous illumination. Please note that freely diffusing, non-nucleosomal histones cannot be tracked at this frame rate. For PA-JF646-labeled nucleosome tracking, the cells were continuously photoactivated with weak 405-nm illumination (1.0 mW, 25% AOTF attenuation) together with 640-nm laser excitation.

To visualize the basal nuclear surface (nuclear periphery), we adjusted the angle of laser illumination to efficiently capture a nuclear membrane marker, NUP107-Venus^109^. Almost uniform distributions of NUP107-Venus were observed in the nuclear periphery condition, while the nuclear interior condition showed a rim of NUP107-Venus^76^. As reported previously^76,77^, nucleosome dynamics on the nuclear surface were lower than those of the nuclear interior, which contained more euchromatin regions. Position-determination accuracy is 7.3 nm (Extended Data Fig. 7g).

### Single-nucleosome tracking analysis

To study nucleosome motion within chromatin domains accurately, we mainly focused on the 0–0.5 s time window, which corresponds to the spatial range of typical chromatin domain sizes (up to ~300 nm). At longer time scales, other factors, such as higher-order structures (for example, compartments, territories) and nuclear movements, become more influential (for details, see refs. ^45,110^). To obtain the *R*_c_ value^57^, we also analyzed longer-time data, up to ~3 s.

Image processing, single-nucleosome tracking and single-nucleosome movement analysis were performed as previously described^5,41,45,76^. Briefly, sequential images were converted to 16-bit grayscale, and background noise was subtracted using the rolling-ball background subtraction (radius, 50 pixels) in ImageJ. Nuclear regions in the images were manually extracted. The fluorescence dots were fitted with a 2D Gaussian function^80,111^ and tracked using u-track software^112^, or their centers were determined by LoG detector in Fiji plugin TrackMate^113^.

To assess positional accuracy, we calculated the s.d. of the 2D movement of immobilized nucleosomes per 50 ms in FA-fixed cells (*n* = 10 nucleosomes). We found that 12.5 nm (the mean of SDx and SDy) was the localization accuracy (Extended Data Fig. 1d). Single-step photobleaching profile confirmed that the individual dots represent single nucleosomes (Fig. 1c).

For single-nucleosome imaging/tracking, we calculated displacement and MSD of nucleosomes, because MSD captures the relevant constrained nucleosome dynamics, for the following reasons: the vast majority of the labeled histones are incorporated into nucleosomes constrained along a very long polymer rather than freely diffusing, and state mixing is minimal. In this respect, single-nucleosome imaging is very different from single-molecule tracking of transcription factors. MSD in single-molecule tracking of transcription factors can be error prone because multiple diffusion states (free 3D diffusion, one-dimensional sliding, specific binding) interconvert, and displacement-based analyses are often preferable (for example, ref. ^114^).

For single-nucleosome movement analysis, the displacement and MSD of the fluorescence dots were calculated based on their trajectory using a Python script. In our tracking, trajectories of single-nucleosome dots on the *XY* plane ${\left({{x}}_{i}\right)}_{i=0}^{n}$ were acquired, where *x*_*i*_ indicates *XY* coordinates at the time point *i*. Then, we calculated the MSD for the lag time ∆ by:

1 $${\mathrm{MSD}}\left({\mathrm{lag}}\,{\mathrm{time}}\right)=\frac{1}{n-\Delta}\sum _{i=1}^{n-\Delta}{\left|{x}_{i}-{x}_{i+\Delta}\right|}^{2}$$

The originally calculated MSD was in 2D. To obtain the 3D value, the 2D value was multiplied by 1.5 (4 to 6 *D* × *t*). The calculated MSDs were fitted to a subdiffusive model MSD(*t*) = 6*D* × *t*^*a*^, where *α* is an anomalous diffusion exponent (0 < *α* < 1). Statistical analyses of the obtained single-nucleosome MSD captured through each histone protein were performed using Python.

The anomalous exponents (*α*) and the coefficient of subdiffusion *D*_sub_ were obtained from the slope and the intercept of the log–log MSD plots by linear regression within the 0–0.5 s time window, respectively. Graphs and statistical analyses of single-nucleosome MSD under different conditions were performed using R. For Spot-on analysis^114^, single-nucleosome trajectory data taken with 10 ms per frame were analyzed. The three-state model is used for model fitting.

### Sample preparation for SIM imaging

Two days before labeling, cells were grown on poly-L-lysine-coated, super-resolution-grade coverslips (No. 1S; 0.17-mm thickness; CS01803, Matsunami). H3.3-Halo in HCT116 cells was fluorescently labeled by incubating the cells for more than 1 h with 50 nM TMR. Cell fixation and immunostaining were performed as described in the Supplementary Methods—‘Verification of DNA damage’. Fixed cells were observed at room temperature.

To experimentally ‘melt’ the euchromatin domains, the cells were treated with TSA (203-17561, Wako) for 7 h before fixation. For the double treatment with TSA and acute cohesin depletion, cells were also treated with 1 µM 5Ph-IAA for the final 1 h. Histone hyperacetylation and RAD21 depletion were confirmed by western blotting. The western blotting procedure was described in the Supplementary Methods—‘Expression and localization of H2B-HaloTag or H3.3-HaloTag’. Membranes were probed with rabbit anti-acetyl H3 (1:2,000; 06-599, Millipore), rabbit anti-acetyl H4 (1:2,000; 06-866, Millipore) and mouse anti-RAD21 (1:1,000; 05-908, Upstate) followed by horseradish peroxidase-conjugated goat antirabbit secondary antibody (1:5,000; 170-6515, Bio-Rad) or antimouse IgG secondary antibody (1:5,000; 170-6516, Bio-Rad). As a loading control, a mouse antilamin A/C antibody (1:1,000; sc-7292, Santa Cruz) was used.

For live imaging, cells were grown on poly-L-lysine-coated glass-based dishes. H3.3-Halo incorporated into nucleosomes was fluorescently labeled with 50 nM HaloTag TMR ligand for 1 h at 37 °C in 5% CO_2_, washed thrice with 1× HBSS and then observed in PR-free McCoy’s 5 A at 37 °C in 5% CO_2_.

For immunostaining, cells were incubated with diluted primary antibodies in 1% NGS in HMK for 1 h—mouse anti-RAD21 (1:1,000; 05-908, Upstate), mouse anti-RNAP II CTD (1:1,000; ab817, Abcam), mouse anti-RNAP II Ser5P (1:1,000; provided by Hiroshi Kimura, Science Tokyo) or mouse anti-RNAP II Ser2P (1:1,000; provided by Hiroshi Kimura, Science Tokyo) antibody. After four washes with HMK, cells were incubated with diluted secondary antibodies in 1% NGS in HMK for 1 h—goat antimouse IgG Alexa Fluor 488 (1:500; A11029, Invitrogen) or goat antimouse IgG Alexa Fluor 647 (1:500; A21236, Invitrogen), followed by four additional washes with HMK. DNA staining and mounting were performed as described in the Supplementary Methods—‘Expression and localization of H2B-HaloTag or H3.3-HaloTag’.

### 3D-SIM microscopy

SIM was performed on a Nikon N-SIM S system (Ti-2 stand; Hamamatsu ORCA-Fusion BT camera; Nikon Perfect Focus; Chroma ET525/50 m, ET595/50 m and ET700/75 m emission filters). A Nikon ×100 PlanApo TIRF oil-immersion objective (NA 1.49) was used. NIS-Elements (Nikon) software was used to control the system and acquire 3D-SIM images. Illumination was provided by ZIVA light engines (Lumencor) containing 405, 476, 545 and 637 nm lasers. Light was directed to the sample through a specific filter for each channel.

Here 3D image stacks were acquired over the entire cell volume in *z*, with 15 raw images per plane (five phases and three angles). Optical sectioning images were acquired at 0.12 µm intervals, with a total of 51 sections covering the entire nucleus (6 µm in thickness). Images acquired on the Nikon N-SIM-S system were reconstructed and corrected for chromatic aberrations using stack reconstruction in the NIS-Elements software.

System performance and both raw and reconstructed data quality were carefully assessed and optimized using the SIMcheck ImageJ plugin (Extended Data Fig. 8a–e)^79^. To exclude potential false-positive nuclear marker signals, we applied the modulation contrast-to-noise ratio (MCNR) map function of SIMcheck, which generates a metric of local stripe contrast across different regions of the raw data and directly correlates this with the level of high-frequency information content in the reconstructed data^79^. Only immunofluorescence spot signals with MCNR values above a stringent quality threshold were considered, whereas localizations with low MCNR values were discarded as unreliable SIM signals.

### Classification analysis for 3D-SIM images

The 3D assessment of DAPI intensity classes or H3.3-Halo intensity classes as a proxy for chromatin compaction was performed as previously described^107,115^. First, 1,000 was subtracted from all voxel intensity values to remove background noise, and nuclei were cropped. Nuclear voxels were automatically identified from the DAPI channel using Gaussian filtering and thresholding through the ‘dapimask’ function in the ‘nucim’ R package.

For chromatin quantification, a 3D nuclear mask was generated to define the region for segmenting DAPI or H3.3-Halo signals into seven intensity classes with equal variance, following the method discussed in refs. ^7,107^. In brief, voxel-wise classification was performed using a hidden Markov random field model that combines a finite Gaussian mixture model with a spatial Potts model, implemented in R. This approach enables threshold-independent classification of chromatin compaction states at the voxel level based on the DAPI or H3.3-Halo intensity. Heatmaps of the seven intensity classes in individual nuclei were generated using Fiji and displayed in either color or grayscale. The relative volume of each class was calculated as the volume of that class divided by the total nuclear volume detected by DAPI staining.

### Power-spectrum analysis for 3D-SIM images

Single midsections from individual nuclei were cropped from the original reconstructed 3D-SIM images. The cropped images were then converted to 2D Fourier spectra using Python^116^. The resulting power spectra were radially averaged to analyze the amplitude at specific spatial periodicities.

### STORM imaging

HCT116 RAD21-mAC H3.3-Halo cells were cultured on poly-L-lysine-coated glass-based dishes (3970-035, Iwaki). H3.3-Halo incorporated into nucleosomes was labeled with 100 nM HMSiR-Halo (A201-01, Goryo Chemical) in medium for 3 h at 37 °C in 5% CO_2_. Cells were washed thrice with PR-free McCoy’s 5A medium (PR-free McCoy’s 5A) with 10% FBS and then incubated with PR-free McCoy’s 5A with 10% FBS and 0.1% dimethyl sulfoxide or 10% FBS and 1 µM 5Ph-IAA for 1 h. Cells were fixed with 3.7% FA (064-00406, Wako) in Opti-MEM (31985062, Gibco) at 37 °C in 5% CO_2_ for 15 min, washed thrice with PR-free McCoy’s 5A with 10% FBS and observed in PR-free McCoy’s 5A with 10% FBS.

Single molecules were observed using an inverted Nikon Eclipse Ti-2 microscope with an ILE laser unit (ANDOR) and the sCMOS ORCA-Fusion BT camera. Fluorescent molecules in living cells were excited by the 640-nm laser through an objective lens (×100 Apo TIRF, NA 1.49; Nikon) and detected. An oblique illumination system with a TIRF unit (TI2-LA-TIRF-E, Nikon) was used to excite labeled molecules within a limited thin area in the cell nucleus and reduce the background noise. Sequential 5,000 images were acquired using NIS-Elements (Nikon solutions) at a frame rate of 10 ms under continuous illumination. To maintain cell culture conditions (37 °C, 5% CO_2_ and humidity) under the microscope, a live-cell chamber with a digital gas mixer and a warming box (Tokai Hit) was used.

STORM images were constructed using the Fiji package ThunderSTORM^117^ based on sequential images of HMSiR. For clustering analysis, TrackMate was also used for localization. The estimated localization accuracy is 4.546 nm for *x* and 4.672 nm for *y* (Extended Data Fig. 8f).

To estimate the individual cluster size, we used the SR-Tesseler-DBSCAN pipeline^82^. First, SR-Tesseler is used with a low-density factor (0.7) to extract chromatin regions, then DBSCAN (with 90-nm distance and 100 minimum samples) detects each cluster.

### Dual-color labeling and imaging

HCT116 RAD21-mAC H3.3-Halo cells were cultured on poly-L-lysine-coated glass-based dishes (3970-035, Iwaki). H3.3-Halo incorporated into nucleosomes was sequentially labeled, first with 200 nM PA-JF549 (ref. ^75^) for 20 min and then with 50 nM PA-JF646 (ref. ^75^) for 20 min at 37 °C in 5% CO_2_. Cells were washed thrice with 1× HBSS (H1387, Sigma-Aldrich) and incubated in PR-free McCoy’s 5A medium for more than 1 h before single-nucleosome imaging (Extended Data Fig. 9h,i).

Simultaneous dual-color imaging in live HCT116 cells was performed under the same conditions as described in ‘Single-nucleosome imaging’, except that a beam splitter (W-VIEW GEMINI, Hamamatsu Photonics) was used, equipped with an image-splitting dichroic beam splitter (FF640-FDi01-25 × 36, Hamamatsu Photonics) and bandpass filters (FF01-593/40-25 and FF01-676/29-25, Hamamatsu Photonics). The cells were continuously photoactivated with weak 405-nm illumination (1.0 mW, 25% AOTF attenuation).

### Two-point MSD analysis

In our single-nucleosome tracking, trajectories of PA-JF549-labeled and PA-JF646-labeled nucleosomes on the *XY* plane, ${\left\{{{\mathbf{{S}}}}_{549}\left({t}_{m}\right)\right\}}_{m=0}^{M-1}$ and ${\left({{\mathbf{{S}}}}_{646}\left({t}_{m}\right)\right)}_{m=0}^{M-1}$, were simultaneously acquired, with a time interval of Δ*t* = 0.05 s and *t*_*m*_ = *m*Δ*t*(*m* = 0, 1, 2,…, *M* − 1). To evaluate dynamic fluctuations between two points, we considered the relative vector between two trajectories, **Q** (*t*_*m*_) = **S**_549_ (*t*_*m*_) − **S**_646_ (*t*_*m*_). Then, we calculated the two-point MSD for the lag time *t*_*n*_ = *n*Δ*t* by

$$\mathrm{MSD}\left({t}_{n}\right)={\left\langle \frac{1}{M-n}\mathop{\sum }\limits_{m=0}^{M-1-n}{\left[{\mathbf{{Q}}}\left({t}_{m+n}\right)-{\mathbf{{Q}}}\left({t}_{m}\right)\right]}^{2}\right\rangle }_{{t}_{n}}\times \frac{3}{2}$$

where ${\left\langle \cdot \right\rangle }_{{t}_{n}}$ represents the ensemble average for trajectories at the lag time *t*_*n*_ and the coefficient 3/2 is a correction factor for conversion from 2D to 3D values.

### Statistics and reproducibility

No statistical method was used to predetermine sample size. Sample sizes were based on previous studies using the same imaging and analysis methods^5,30,45^. For cell-based experiments, a biological replicate was defined as an independently performed experiment using a separately prepared cell culture. The numbers of biological replicates and cells analyzed in each replicate, the definition of *n*, the statistical tests used and exact *P* values are provided in the figure legends of Figs. 1–8 and Extended Data Figs. 1–10 and in Supplementary Table 1. For the primary analyses of cell-to-cell heterogeneity (Figs. 1–4 and 7 and Extended Data Figs. 1, 3, 4, 7 and 9), measurements from individual cells pooled across biological replicates were used to compare distributions between conditions using the statistical tests indicated in the figure legends. In parallel, replicate-aware analyses were performed by summarizing individual-cell measurements within each biological replicate and using the resulting biological replicate-level values for statistical testing. These analyses were used to confirm that the differences observed in the pooled cell populations were reproducible across independent experiments. Paired tests were used when control and treatment conditions were measured in parallel within the same biological replicate. Where multiple pairwise comparisons were performed, *P* values were adjusted using the Holm method. All statistical tests were two sided unless otherwise stated. For parametric tests, data were assumed to follow a normal distribution, but this assumption was not formally tested. Data not meeting the technical or quality-control criteria described in the Methods subsections ‘Target protein depletion by AID2’, ‘Single-nucleosome tracking analysis’, ‘3D-SIM microscopy’ and ‘STORM imaging’, or in the Supplementary Methods subsections ‘Head-tethering of the cohesin’, ‘ATAC-seq experiments and data processing’, ‘Intron-seqFISH probe selection and design’ and ‘Intron-seqFISH’, were excluded from the analyses; no other data were excluded. The experiments were not randomized. The investigators were not blinded to allocation during experiments and outcome assessment. The numbers of independent repeats for experiments showing representative images are stated in the corresponding figure legends and Supplementary Table 1.

### Reporting summary

Further information on research design is available in the Nature Portfolio Reporting Summary linked to this article.

## Online content

Any methods, additional references, Nature Portfolio reporting summaries, source data, extended data, supplementary information, acknowledgements, peer review information; details of author contributions and competing interests; and statements of data and code availability are available at https://doi.org/10.1038/s41588-026-02736-2.

## Supplementary information

Supplementary Information Supplementary Methods.

Reporting Summary

Peer Review File

Supplementary Video 1 Video data (50 ms per frame) of single H2B-Halo nucleosomes labeled with TMR in a living HCT116 cell recorded with an sCMOS ORCA-Fusion BT camera (Hamamatsu Photonics). Clearly, well-separated dots showing single-step photobleaching after background subtraction (Fig. 1b) indicate that each dot corresponds to a single H2B-Halo-TMR molecule within a nucleosome. Circles in the right video highlight tracked dots. Dots observed for more than three frames were used for further analysis.

Supplementary Video 2 Video data (50 ms per frame) of single H2B-Halo nucleosomes labeled with TMR in a living HCT116 cell treated with 0.1% DMSO (control; left) or 1 µM 5Ph-IAA (ΔRAD21; right). Please note that nucleosomes move faster when treated with 5Ph-IAA than with DMSO.

Supplementary Video 3 Video data (50 ms per frame) of single H2B-Halo nucleosomes labeled with TMR in a living HCT116 cell. Each moving dot is outlined by a colored circle indicating its RL-classified subpopulation—orange, superfast; yellow, fast; green, slow; blue, superslow. Some dots remain unclassified, as the RL algorithm classifies only trajectories tracked for more than 11 consecutive frames (0.5 s). Please note that superfast does not correspond to free histones. The nuclear region is outlined by a dotted yellow line.

Supplementary Video 4 3D-SIM volume reconstruction of a fixed HCT116 nucleus labeled with DAPI and H3.3-Halo-TMR. Images were acquired with a Nikon N-SIM-S system using a piezo Z-drive at 0.12-µm step size. Images were reconstructed with stack reconstruction in NIS-Elements. The video was generated with the NIS-Elements Volume Viewer (mode: MAX-IP).

Supplementary Video 5 3D-SIM volume reconstruction of a live HCT116 nucleus labeled with H3.3-Halo-TMR.

Supplementary Video 6 3D-SIM volume reconstruction of a fixed HCT116 nucleus treated with 0.1% DMSO (control) and labeled with DAPI and H3.3-Halo-TMR.

Supplementary Video 7 3D-SIM volume reconstruction of a fixed HCT116 nucleus labeled with H3.3-Halo-TMR. The cell was treated with 1 µM 5Ph-IAA (ΔRAD21).

Supplementary Video 8 Video of two closely positioned single nucleosomes (left, labeled with PA-JF549; right, labeled with PA-JF646). The nucleosomes were recorded simultaneously at 50 ms per frame using W-VIEW GEMINI (Hamamatsu Photonics) with a pixel size of 65 nm. Tracked dots are outlined with magenta circles. An example of two dots that coexisted in the same frame and, by chance, had an average interpoint distance of <150 nm is highlighted by a square and enlarged in the inset. Trajectories of these two dots are shown in red. The video was generated using the TrackMate plugin.

Supplementary Video 9 Simulated dynamics of an example chromatin chain consisting of two consecutive domains (green and gray) with cohesin (left, control) and without cohesin (right, Δcohesin; Fig. 8b,c).

Supplementary Tables 1 and 2 Supplementary Table 1: Details and raw values of replicate-aware statistical tests. Supplementary Table 2: List of primary probe oligonucleotides, as well as corresponding secondary and readout probes for intron-seqFISH.

## Source data

Source Data Fig. 1 Numerical source data.

Source Data Fig. 2 Numerical source data.

Source Data Fig. 3 Unprocessed western blots and numerical source data.

Source Data Fig. 4 Numerical source data.

Source Data Fig. 5 Numerical source data.

Source Data Fig. 6 Numerical source data.

Source Data Fig. 7 Numerical source data.

Source Data Fig. 8 Numerical source data.

Source Data Extended Data Fig. 1 Unprocessed western blots and numerical source data.

Source Data Extended Data Fig. 2 Unprocessed western blots and numerical source data.

Source Data Extended Data Fig. 3 Unprocessed western blots and numerical source data.

Source Data Extended Data Fig. 4 Numerical source data.

Source Data Extended Data Fig. 5 Unprocessed western blots and numerical source data.

Source Data Extended Data Fig. 6 Numerical source data.

Source Data Extended Data Fig. 7 Numerical source data.

Source Data Extended Data Fig. 8 Numerical source data.

Source Data Extended Data Fig. 9 Unprocessed western blots and numerical source data.

Source Data Extended Data Fig. 10 Numerical source data.

## Data Availability

The pulldown sequencing data are publicly available in the DDBJ BioProject database under accessions PRJDB17378 (HeLa H2B-Halo; SRA runs DRR527334–DRR527339; https://ddbj.nig.ac.jp/search/entry/bioproject/PRJDB17378/)^118^, PRJDB17380 (HeLa H3.3-Halo; SRA runs DRR527328–DRR527333; https://ddbj.nig.ac.jp/search/entry/bioproject/PRJDB17380/) and PRJDB39674 (HCT116 H2B-Halo and H3.3-Halo; SRA runs DRR801689–DRR801696; https://ddbj.nig.ac.jp/search/entry/bioproject/PRJDB39674/). ATAC–seq data have been deposited in GEO (GSE318385). Source data are provided with this paper.

## Code availability

The scripts for RL-algorithm classification, TrackMate Batch Tracker, SIM image analysis and two-point MSD calculation are available at https://doi.org/10.5281/zenodo.16959115 (ref. ^119^). The codebase for the polymer model of chromatin domains is available at https://doi.org/10.5281/zenodo.18404051 (ref. ^120^).
