# Supplementary material for: Cohesin prevents local mixing of condensed euchromatic domains in living human cells
Source: Nat Genet. 2026 Sep 8;58(9):2335–49. doi: 10.1038/s41588-026-02736-2 (PMC13553310; doi:10.1038/s41588-026-02736-2)
Supplement: Supplementary file 1 [file 41588_2026_2736_MOESM1_ESM.pdf]

nature genetics

Supplementary information

https://doi.org/10.1038/s41588-026-02736-2

Cohesin prevents local mixing of condensed euchromatic domains in living human cells

In the format provided by the authors and unedited

Supplementary Note 1

Supplementary Methods

Establishment of stable cell lines

For the establishment of HeLa cells stably expressing H2B-HaloTag or H3.3-HaloTag, the Flp-In system (V602020; Invitrogen) was used. pFRT-bla was first transfected into HeLa cells to integrate into the genome using the Effectene transfection reagent kit (301425; QIAGEN). Cells containing the FRT site were selected with 5 μg/mL blasticidin S (029-18701; Wako) and used for isolation of stable transformants. The isolation procedure using the Flp-In system was as previously described [1]. pEF1-H2B-Halo-FRT or pEF1-H3.3-Halo-FRT was then transfected into HeLa cells harboring an FRT site, and transformants were selected with 200 μg/mL hygromycin B (10687-010; Invitrogen).

To stably express H2B-Halo or H3.3-Halo in the HCT116 cell line, the PiggyBac transposon system was used. The constructed plasmid pPB-CAG-IB-H2B-HaloTag or pPB-EF1α-H3.3-HaloTag was co-transfected with pCMV-hyPBase (provided by the Sanger Institute under a materials transfer agreement) into HCT116 cells using the Effectene transfection reagent kit. Transfected cells were selected with 10 μg/mL blasticidin S. HCT116 cells expressing OsTIR1(F74G) and endogenously tagged RAD21-mAID-mClover, CTCF-mAID-mClover, or WAPL-mAID-mClover [2,3] were used as parental cells. To stably express H2B-Halo in the HCT116 cell line with the head-tethering cohesin system [4], pPB-CAG-IB-H2B-HaloTag was co-transfected with pCMV-hyPBase into HCT116 cells using the Effectene transfection reagent kit. In this system, endogenous SMC1 is tagged with FK506-binding protein (FKBP) and endogenous SMC3 is tagged with a mutated FKBP–rapamycin-binding domain (FRB*).

Addition of the non-toxic rapamycin analog C16-(S)-7-methylindolerapamycin (AP21967/C16-AiRap), which selectively binds FRB* [5,6], induces forced head domain closure of cohesin (Fig. 3i). Cells expressing H2B-Halo were sorted based on fluorescence, as described in [7]. Two weeks after transfection, cells expressing H2B-Halo were labeled with 50 nM HaloTag TMR ligand overnight at 37 °C in 5% CO_2. HaloTag TMR-positive cells were collected by flow cytometry (SH800S, Sony Biotechnology). The collected cells were subcloned, and clones stably expressing H2B-HaloTag were selected.

Expression and localization of H2B-HaloTag or H3.3-HaloTag

To examine the expression level of H2B-HaloTag or H3.3-HaloTag, transfected cells were lysed in Laemmli sample buffer [8] supplemented with 10% 2-mercaptoethanol (133-1457; Wako) and incubated at 95 °C for 5 min to denature proteins. Cell lysates, equivalent to 1×10^5 cells per well, were subjected to 12.5% SDS–PAGE and subsequent western blotting. For western blotting, the fractionated proteins were transferred to a PVDF membrane (IPVH00010; Millipore) using a semi-dry blotter (BE-320; BIO CRAFT). After blocking with 5% skim milk (190-12865; Fujifilm Wako), the membrane was probed with rabbit anti-H2B (1:10000; ab1790; Abcam), rabbit anti-H3 (1:100000; ab1791; Abcam), or mouse anti-HaloTag (1:1000; G9211; Promega) antibodies, followed by horseradish peroxidase–conjugated secondary antibodies: anti-rabbit (1:5000; 170-6515; Bio-Rad) or anti-mouse (1:5000; 170-6516; Bio-Rad). Chemiluminescence was detected using WBKLS0100 (Millipore) and EZ-Capture MG (AE-9300H-CSP; ATTO).

To examine the cellular localization of the H2B-HaloTag or H3.3-HaloTag, cells grown on poly-L-lysine–coated coverslips (P1524-500MG; Sigma-Aldrich; C018001; Matsunami) were treated

with 5 nM HaloTag TMR ligand (8251; Promega) overnight at 37 °C in 5% CO_2 or with 10 nM JFX650 HaloTag Ligand (HT1070; Promega) for more than 1 hour. The cells were fixed with 1.85% formaldehyde (064-00406; Wako) at room temperature for 15 min, permeabilized with 0.5% Triton X-100 (T-9284; Sigma-Aldrich) for 15 min. For immunostaining, cells were incubated with 10% normal goat serum (NGS; 143-06561; Wako) in HMK for 30 min, then incubated with diluted primary antibodies in 1% NGS in HMK (20 mM HEPES [pH 7.5], 1 mM $MgCl_2$, and 100 mM KCl) for 1 h: mouse anti-H3K27me3 (1:1000; provided by Hiroshi Kimura, Science Tokyo, Japan) antibody, or mouse anti-H3K9me3 (1:1000; provided by Hiroshi Kimura, Science Tokyo, Japan) antibody. After four washes with HMK, cells were incubated with diluted secondary antibodies in 1% NGS in HMK for 1 h: goat anti–mouse IgG Alexa Fluor 594 (1:1000; A11032, Invitrogen), followed by four additional washes with HMK. The cells were stained with 0.5 μg/mL DAPI (10236276001; Roche) for 5 min. Samples were mounted with PPDI (20 mM HEPES [pH 7.4], 1 mM $MgCl_2$, 100 mM KCl, 78% glycerol, and 1 mg/mL para-phenylenediamine [695106-1G; Sigma-Aldrich]) [9]. Z-stack images (0.2 μm steps, 30 sections) were acquired using a DeltaVision Ultra microscope (Applied Precision) equipped with an Olympus PlanApoN 60× objective (NA 1.42), an sCMOS camera, an InsightSSI light source (~50 mW), and a four-color standard filter set. Generally, H2B-Halo shows genome-wide localization, due to the frequent replacement of histone H2B within a few hours [10], while H3.3-Halo shows euchromatic localization [11-13].

Biochemical fractionation of nuclei from cells expressing H2B-HaloTag or H3.3-HaloTag

Nuclei were isolated from HeLa S3 or HCT116 cells expressing H2B-HaloTag or H3.3-HaloTag as described previously [14-16]. Briefly, collected cells were suspended in nuclei isolation buffer (3.75 mM Tris-HCl [pH 7.5], 20 mM KCl, 0.5 mM EDTA, 0.05 mM spermine, 0.125 mM

spermidine, 1 μg/mL Aprotinin [T010A; TaKaRa], and 0.1 mM PMSF [P7626-1G; Sigma-Aldrich]) and centrifuged at 1,936 × *g* for 7 min at room temperature. The cell pellets were resuspended in nuclei isolation buffer and again centrifuged at 1,936 × *g* for 7 min at room temperature. Subsequent steps were performed at 4 °C, unless otherwise noted. Cell pellets were resuspended in nuclei isolation buffer containing 0.025% Empigen (45165-50ML; Sigma-Aldrich; nuclei isolation buffer+) and homogenized immediately with 10 downward strokes of a tight Dounce-pestle (357546, Wheaton). The cell lysates were centrifuged at 4,336 × *g* for 5 min. Nuclear pellets were washed in nuclei isolation buffer+. The nuclei were incubated on ice for 15 min in the following buffers containing various concentrations of salt: HE (10 mM HEPES-NaOH [pH 7.5], 1 mM EDTA, and 0.1 mM PMSF), HE + 100 mM NaCl, HE + 500 mM NaCl, HE + 1 M NaCl, and HE + 2 M NaCl. After each buffer incubation with increasing concentrations of salt, centrifugation was performed to separate the nuclear suspensions into supernatant and pellet fractions. The proteins in the supernatant fractions were precipitated by using 17% trichloroacetic acid (208-08081; Wako) and cold acetone. Both pellets were suspended in a Laemmli sample buffer and subjected to 12.5% SDS-PAGE, followed by Coomassie brilliant blue (031-17922; Wako) staining and western blotting using rabbit anti–H2B (1:10000; ab1790; Abcam), rabbit anti-H3 (1:100,000; ab1791; Abcam) or mouse anti–HaloTag (1:1000; G9211; Promega) antibodies, followed by the appropriate secondary antibody: anti-rabbit (1:5000 dilution; 170-6515; Bio-Rad) or anti-mouse (1:5000 dilution; 170-6516; Bio-Rad) horseradish peroxidase-conjugated goat antibody.

Verification of DNA damage

To verify the level of DNA damage caused by rapid RAD21 depletion, the γH2AX immunostaining signal was quantified. HCT116 RAD21-mAID-mClover cells were treated with

1 μM 5Ph-IAA or 0.1% DMSO. As a positive control, cells were treated with 10 μg/mL bleomycin (4234400D4032; Nippon Kayaku) for 1 h. Immunostaining was performed as follows, with all steps carried out at room temperature. Cells grown on coverslips were fixed and permeabilized as described in the section "Expression and localization of H2B-HaloTag or H3.3-HaloTag." After washing twice with HMK for 5 min, the cells were incubated with 10% NGS in HMK for 30 min. The cells were then incubated for 1 h with rabbit anti-γH2AX (phospho S139) (1:1000, ab2893; Abcam) primary antibody diluted in 1% NGS in HMK. After four washes with HMK, the cells were incubated for 1 h with goat anti–rabbit IgG Alexa Fluor 594 (1:1000, A11037; Invitrogen) secondary antibody diluted in 1% NGS in HMK, followed by four washes with HMK.

DNA staining and mounting were performed as described in the section "Expression and localization of H2B-HaloTag or H3.3-HaloTag." Optical section images were acquired at a 0.2 μm step size using a DeltaVision microscope (Applied Precision) as described in the same section. The number of γH2AX foci per nucleus was automatically quantified using Fiji. A fixed threshold value, determined by the Otsu method, was applied to segment the foci, which were then counted within each nucleus.

Head-tethering of the cohesin

Cells were treated with 1.5 μM AP21967 (A/C Heterodimerizer; 635057; Clontech) for 2 h. For RAD21 depletion in the head-tetherable cohesin cell line, cells were treated with 1 μg/mL doxycycline (631311; Clontech) overnight, followed by 500 μM indole-3-acetic acid (IAA; a natural auxin, 19119-61; Nacalai) for 1 h. Subsequently, 1.5 μM AP21967 was added for 2 h in the continued presence of both doxycycline and IAA (total IAA exposure: 3 h). Control cells were treated with 0.1% MQ (solvent used to dissolve doxycycline), 0.3% EtOH (for AP21967),

and 0.1% DMSO (for IAA). RAD21 depletion was confirmed by the loss of mClover fluorescence during single-nucleosome imaging, and only cells with confirmed depletion were used for analysis.

RNA interference

siRNA reverse transfections into HeLa, HCT116, and HT1080 cells grown on poly-L-lysine–coated glass-based dishes were performed using Lipofectamine RNAiMAX (13778-075; Invitrogen) according to the manufacturer’s instructions. The medium was replaced with fresh medium 16 h after transfection. The transfected cells were used for subsequent experiments 48 h (RAD21, CTCF) or 72 h (WAPL, Sororin) after transfection. As a control, an oligonucleotide (4390843; Ambion; sequence undisclosed) was used. The siRNA oligo sequences and concentrations used were as follows: RAD21 (60 nM, 5'-CAGCUUGAAUCAGAGUAGAGUGGAAdTdT-3'), CTCF (60 nM, 5'-GCGCUCUAAGAAAGAAGAUUCCUCUdTdT-3'), WAPL (80 nM, 5'-GGUUAAGUGUUCCUCUUAUdTdT-3'), Sororin (60 nM, 5'-GCCUAGGUGUCCUUGAGCUdTdT-3').

Knockdown of target proteins was confirmed by immunostaining, performed as described in the section “Verification of DNA damage.” Cells were incubated for 1 h with primary antibodies diluted in 1% NGS in HMK: mouse anti-RAD21 (1:1000; 05-908; Upstate), mouse anti-CTCF (1:3000; 07-729; Millipore), mouse anti-WAPL (1:20000; MABE1106; Millipore), or rabbit anti-Sororin (CDCA5) (1:2500; ab192237; Abcam). After four washes with HMK, cells were incubated for 1 h with secondary antibodies diluted in 1% NGS in HMK: goat anti–mouse IgG Alexa Fluor 488 (1:1000, A11029; Invitrogen), goat anti–mouse IgG Alexa Fluor 594 (1:1000,

A11032; Invitrogen), goat anti–rabbit IgG Alexa Fluor 594 (1:1000, A11037; Invitrogen), or goat anti–rabbit IgG Alexa Fluor 647 (1:1000, A21245; Invitrogen).

Optical section images were acquired at a 0.2 µm step size using a DeltaVision microscope (Applied Precision) as described in the section "Expression and localization of H2B-HaloTag or H3.3-HaloTag in HeLa cells." To quantify the knockdown effect, the mean nuclear signal intensities were calculated after background subtraction (signals outside nuclei) and plotted.

Cell cycle synchronization

For synchronization of HCT116 cells treated with siSororin RNA in the late S–G2 phase, cells (1.0×10^5 cells/dish) were plated in medium containing siSororin RNA. Cells were then treated with 9 µM RO3306 (CS-3790; Funakoshi) for 16 h before observation. For synchronization of HCT116 or HT1080 cells in the late S–G2 phase, cells were initially plated at a concentration of 1.0×10^5 cells/mL, incubated for one day, and then treated with 9 µM RO3306 for 16 h.

Chromosome spreads

To assess the formation of sister chromatid cohesion, mitotic chromosome spreads were prepared. Cells (treated with siControl or siSororin RNA) were incubated with 0.2 µg/mL colcemid (045-16963; Wako) for 1 h at 37 °C. The following steps were performed at room temperature, except for the final fixation step. After washing with PBS, cells were trypsinized and resuspended in hypotonic buffer (75 mM KCl) for 10 min. Cells were then gently fixed by repeating the following treatment three times: incubation in fixation buffer (methanol:acetic acid, 3:1) for 5 min, centrifugation at $850 \times g$ for 4 min, and resuspension in fresh fixation buffer.

Finally, the cells were completely fixed by incubation in 200 µL of fixation buffer at –30 °C for at least 30 min.

For imaging, 5 µL of the fixed cell suspension was dropped onto coverslips, mixed, and completely dried at 60 °C for 30 min. Dried cells were stained with 0.5 µg/mL DAPI for 5 min, followed by PPDI mounting. Images of mitotic chromosome spreads were obtained using a DeltaVision Ultra microscope with an Olympus PlanApoN 60× objective (NA 1.42) and an sCMOS camera. The number of chromosomes per cell was counted manually. We confirmed the loss of sister chromatid cohesion in Sororin-depleted cells: the percentage of separated or X-shaped sister chromatids increased (Fig. 2b), consistent with a previous report [17].

lacO/EGFP-LacI and _tetO_/TetR-4×mCherry foci imaging and tracking

lacO/EGFP-LacI and *tetO*/TetR-4×mCherry foci in live HT1080 cells were imaged under the same conditions as described in "Single-nucleosome imaging," except that a lower laser power was used due to the higher fluorescence intensity of the foci compared with TMR-labeled single nucleosomes. EGFP-LacI was excited with a 488 nm laser, and TetR-mCherry with a 561 nm laser. Imaging was performed sequentially, first acquiring the 561 nm channel, followed by the 488 nm channel. Movies were recorded at a frame rate of 50 ms per frame, with a total of 1200 frames (1 min). Cells were treated with siRNA (see "RNA interference") and synchronized to the late S–G2 phase (see "Cell cycle synchronization"). The precise positions of the labeled genomic loci were identified and tracked using the TrackMate plugin [18,19]. Mean squared displacement (MSD) of the foci was calculated from the trajectories using the same procedure described in "Single-nucleosome tracking analysis." The *tetO* array, visualized with TetR-4×mCherry, more frequently appears as two separate dots compared to the *lacO* array, visualized with EGFP-LacI.

This suggests that the *lacO* array is located near a cohesin site, which likely prevents the sister loci from separating [20]. Consistently, ENCODE data [21] indicated that the *lacO* site lies within 5 kb of a cohesin-enriched region (e.g., SMC3 and RAD21; Fig. 2e), as shown in [20].

Angle-distribution analysis

For the tracked consecutive points $\{(x_0, y_0),\ (x_1, y_1),\ \cdots, (x_n, y_n),\ \cdots\}$ of a single nucleosome on the XY plane, we converted the data into a set of displacement vectors, $\Delta\boldsymbol{r}_n = (x_{n+1} - x_n, y_{n+1} - y_n)^t$. Then, we calculated the angle between two vectors $\Delta\boldsymbol{r}_n$ and $\Delta\boldsymbol{r}_{n+1}$. This procedure was carried out for all points in each trajectory. Finally, we plotted the normalized polar histogram by our Python program. The angle distribution was normalized by 2π, and the values correspond to the probability density. The colors indicate the angles. Asymmetry coefficient (AC) was calculated as indicated in Extended Data Fig. 1h.

Bayesian-based Richardson-Lucy algorithm (RL algorithm)

The RL algorithm is the iterative algorithm of Richardson [22] and Lucy [23] (RL) to derive smooth distributions from the noisy data used in image processing [24,25]. With sufficient observation, the algorithm converts raw, noisy trajectories into a smooth distribution of MSD, providing a denoised, population-level view of heterogeneous nucleosome mobility.

For the convenience of the reader, we briefly explain the mathematical principles of the RL algorithm. A more detailed description has been reported previously [26].

Here, the average MSD of nucleosomes, $\bar{M} = \langle M_i \rangle$, where $\langle \cdots \rangle$ is the average taken over *i* and along the observed trajectories.

The distribution of MSD is captured by

$P(M,t) = \langle \delta(M - M_i(t)) \rangle$

Due to the short lifetime of observed fluorescence of single nucleosomes, the individual nucleosome MSD data are insufficient to provide for a clear $P(M,t)$. However, this problem is overcome by using the RL algorithm. From the observed data, we first calculate the self-part of the van Hove correlation function (vHC),

$G_s(r,t) = A_s \langle \delta(r - |r_i(t + t_0) - r_i(t_0)|) \rangle$,

where r_i is the projected coordinate of the ith nucleosome on the 2-dimensional imaging plane and $A_s$is a constant to normalize G_s as $\int d^2 r G_s(r,t) = 1$. The calculated vHC is shown at t =0.5 s. G_s is expanded in Gaussian bases, $q(r,M) = \left(\frac{1}{\pi M}\right) \exp\left(-\frac{r^2}{M}\right)$, as

$$G_s(r,t) = \int dMP(M,t)q(r,M)$$

Given a noisy estimate of $G_s(r,t)$, $P(M,t)$ is extracted as coefficients of expansion using the RL iterative scheme. For this, $P(M,t)$ was calculated in an iterative way with the RL algorithm: starting from the initial distribution, $P^1(M,t) = 1/M_0 \exp(-\frac{M}{M_0})$, $P^{n+1}(M,t)$ at the (n+1)-th iteration was obtained by $P^{n+1}(M,t) = P^n(M,t) \int \left[\frac{G_s(r,t)}{G_s^n(r,t)}\right] q(r,M) d^2 r$, with $G_s^n(r,t) = \int P^n (M,t) q(r,M) dM$. This equation was iterated under the constraints $P^{n+1}(M,t) > 0$ and $\int P^{n+1} (M,t) dM = 1$.

The obtained distribution curves were fitted with multiple Gaussian functions, and the peak position and area of each Gaussian component were calculated. Subpopulation boundaries were estimated from the RL-curve: using the local minima for the "Super slow"–"Slow" and "Slow"–"Fast" transitions, and the midpoint between peaks for the "Fast"–"Super fast" transition. For MSD analysis, trajectories within each cell were categorized based on their MSD at $\Delta t = 0.5$ s, and mean squared displacement (MSD) was calculated separately for each subpopulation.

Centroids of the categorized trajectories were mapped to visualize their localization. It should be noted that reliable fitting of α values for the "Super slow" and "Slow" categories is difficult due to their limited mobility.

H3.3-Halo labeled nucleosome pulldown, purification of nucleosomal DNA, and library preparation

Cell nuclei were isolated as previously described [14-16], with minor modifications. Briefly, HCT116 or HeLa cells were maintained at 37 °C and 5% CO_2 in McCoy's 5A or DMEM supplemented with 10% FBS, respectively. Collected cells were washed with nuclei isolation buffer (3.75 mM Tris–HCl pH 7.5, 20 mM KCl, 0.5 mM EDTA, 0.05 mM spermine, 0.125 mM spermidine, 0.1% Trasylol (14716; Cayman Chemical), 0.1 mM PMSF (P7626-1G; Sigma-Aldrich)) with two cycles of centrifugation at 193 × *g* for 7 min at 23 °C. Pellets were resuspended in nuclei isolation buffer containing 0.05% Empigen BB detergent (45165-50ML; Sigma-Aldrich) (nuclei isolation buffer+) and homogenized with ten downward strokes using a tight Dounce pestle. After centrifugation at 440 × *g* for 5 min, the pellets were washed with nuclei isolation buffer+. This step was repeated four times before nucleosome purification.

Chromatin purification was performed as described by [27,28], with modifications. Nuclei (equivalent to ~2 mg of DNA) in buffer (10 mM Tris–HCl pH 7.5, 1.5 mM $MgCl_2$, 1.0 mM $CaCl_2$, 0.25 M sucrose, 0.1 mM PMSF) were digested with 50 U micrococcal nuclease (MNase, LS004797; Worthington) at 30 °C for 1 h. During digestion, 1 μM HaloTag PEG-Biotin Ligand (G859A; Promega) or an equivalent volume of DMSO was added. After centrifugation at 440 × *g* for 5 min at 4 °C, nuclei were lysed in lysis buffer (10 mM Tris–HCl pH 8.0, 5 mM EDTA, 0.1 mM PMSF) on ice for 5 min. Lysates were dialyzed overnight at 4 °C against dialysis buffer (10

mM HEPES–NaOH pH 7.5, 0.1 mM EDTA, 0.1 mM PMSF) using Slide-A-Lyzer (66380; Thermo Fisher Scientific). After centrifugation at 20,400 × *g* for 10 min at 4 °C, the supernatant was recovered as the purified nucleosome fraction (mainly mononucleosomes).

To verify MNase digestion, DNA was purified, run on a 1.5% agarose gel, and stained with ethidium bromide (315-90051; NIPPON GENE). The biotinylated or untreated nucleosome fraction (28 µg) was diluted with an equal volume of 2× binding buffer (10 mM HEPES–NaOH pH 7.5, 200 mM NaCl, 2 mM DTT, and cOmplete EDTA-free protease inhibitor cocktail (11873580001; Roche)). As described [29], chromatin solution was added to streptavidin-FG beads (TAS8848 N1170; Tamagawa-Seiki) pre-equilibrated with coating buffer (PBS, 2.5% BSA, 0.05% Tween 20) for 1 h. Mixtures were incubated at 5 °C for 2 h with shaking. Beads were collected with a magnetic rack and washed as described [30]. In brief cells were washed once with cold 1× binding buffer (10 mM HEPES–NaOH pH 7.5, 100 mM NaCl, 1 mM DTT), twice with cold wash buffer 1 (10 mM HEPES–NaOH pH 7.5, 140 mM NaCl, 1% Triton X-100, 0.5% NP-40 (11332473001; Roche), 0.1% SDS), twice with cold wash buffer 2 (10 mM HEPES–NaOH pH 7.5, 360 mM NaCl, 1% Triton X-100, 0.5% NP-40, 0.1% SDS), twice with cold wash buffer 3 (10 mM HEPES–NaOH pH 7.5, 250 mM LiCl, 0.5% Triton X-100, 0.5% NP-40), and once with TE buffer (10 mM Tris–HCl pH 8.0, 1 mM EDTA).

Beads were resuspended in 50 µL TE buffer. For DNA extraction, 40 µL of beads were treated with RNase A (1 mg/mL, 10109169001; Roche, 4 µL) for 30 min at 37 °C, followed by proteinase K digestion (20 mg/mL, 169-21041; FUJIFILM Wako, 4 µL) with 10 µL of 10% SDS at 50 °C for 1 h with shaking (1,200 rpm). After magnetic separation, DNA was extracted using AMPure XP beads (A63880; Beckman Coulter) and eluted in Milli-Q water. DNA concentration was quantified with the Qubit system (Q32851; Thermo Fisher Scientific), and quality was

assessed by Agilent 2100 Bioanalyzer using a High Sensitivity DNA kit (5067-4626; Agilent). Libraries were prepared using the ThruPLEX DNA-seq Kit (R400675; Takara Bio) and checked with an Agilent 2100 Bioanalyzer. Pooled libraries were sequenced (paired-end, 2 × 100 bp) on the Illumina NovaSeq 6000 platform.

For protein analysis, the remaining 10 µL of beads were resuspended in an equal volume of 2× Laemmli sample buffer [8] containing 10% 2-mercaptoethanol (133-1457; FUJIFILM Wako) and incubated at 99 °C for 10 min. After centrifugation at 10000 × *g* for 3 min, input and pulldown samples (biotinylated and negative control) were separated by SDS–PAGE and analyzed by western blotting with anti-HaloTag antibody (G9211; Promega).

For data analysis of purified H3.3-Halo nucleosomal DNA, the nf-core ChIP-seq pipeline (nfcore/chipseq v2.1.0) [31,32] was used with Docker configuration and default parameters. The human genome hg19 (Illumina iGenomes) was used as a reference. Peaks were called using MACS2 (broad mode) [33]. Overlap between H3.3-Halo regions and contact domains (Hi-C A compartments, histone modifications) was assessed against published datasets [34-36]. ChIP-seq peaks for histone modifications in HCT116 cells were downloaded from 4D Nucleome data portal[37,38]: Boundaries (4DNFIBKY9EG9), Compartment (4DNFIZHT1Y8P), RAD21 (4DNFIM7KV9EA), CTCF (4DNFIT3E2YZZ), RNAP II (4DNFIFNJW3NE), H3K4me1 (4DNFI27LAZBR), H3K4me3 (4DNFIRPBX1BM), H3K27ac(4DNFIGINV1VI), H3K36me3(4DNFIPYVJMFK), H3K79me2 (4DNFI7O6ZSBK), H4K16Ac (4DNFI6DW6GVA), H3K27me3 (4DNFI2EWSBH4), H3K9me3 (4DNFIV229BKJ), H4K20me3 (4DNFIMYKFY9Y). ATAC-seq data was generated in this study. Hi-C domains from HeLa [39] were obtained from GEO (GSE63525). Compartment annotations were from SNIPER predictions [40]. ChIP-seq peaks for histone modifications in HeLa cells were

downloaded from ENCODE [21,41]: H3K4me2 (ENCFF108DAJ), H3K4me3 (ENCFF447CLK), H3K9ac (ENCFF723WDR), H3K27ac (ENCFF144EOJ), H3K79me2 (ENCFF916VLX), H3K36me3 (ENCFF001SVY), H3K4me1 (ENCFF162RSB), and H3K27me3 (ENCFF252BLX).

Identification of broad H3.3-enriched regions

Broad H3.3-enriched regions were called using epic2 [42]. Replicate BAM files (Rep1 and Rep2) were processed independently with a 5-kb bin size, allowing up to two consecutive gaps, and using an FDR cutoff of 0.05. To derive a reproducible set of broad H3.3 domains, the two replicate callsets were first combined and overlapping intervals were merged to generate a union set. Subsequently, intervals that reciprocally overlapped by at least 30% of their length between Rep1 and Rep2 were retained, and the supported intervals from both replicates were merged to obtain the final intersection set. The resulting reproducible H3.3 domain set comprised 1,553 intervals with a median length of ~210 kb.

Peak sets used for distance-to-boundary analyses

Marker peak sets included ChIP-seq peaks for H3K27Ac, H3K4me1, H3K4me3, H3K36me3, H3K27me3, H3K9me3, H4K16Ac, H4K20me3, and H3K79me2, as well as ChIP-seq peaks for RNAP II, RAD21, and CTCF, and ATAC-seq peaks. Hi-C boundary annotations were obtained from in situ Hi-C [35]. RNAP II/RAD21/CTCF ChIP-seq datasets were taken from [36], while ATAC-seq was generated in this study.

Signed distance of peaks to H3.3 domain boundaries

H3.3 domains were represented as intervals (chr, s_i, e_i) and each marker peak (chr, a_j, b_j) was represented by its midpoint $m_j = (a_j + b_j)/2$. For each midpoint, the closest H3.3 domain on

the same chromosome was defined as the one minimizing the distance to either boundary (start or end). A signed distance d(bp) was computed as the minimum distance to the nearest boundary, assigned positive if the midpoint lies inside the domain $\left(s_i \leq m_j < e_i\right)$ and negative if it lies outside $\left(m_j < s_i \text{ or } m_j \geq e_i\right)$. Thus, $d = 0$ corresponds to the H3.3 boundary, negative values indicate outside positions, and positive values indicate inside positions, enabling direct comparison of distances on the same bp scale.

ATAC-seq experiments and data processing

ATAC-seq was performed as previously described with minor modification [43]. Briefly, HCT116 cells treated with DMSO or 5Ph-IAA (1 µM, 3 h) were harvested by trypsin dissociation and 50,000 viable cells per sample were collected, washed once with ice-cold phosphate-buffered saline (PBS), and lysed in 50 µL ATAC-Resuspension Buffer (RSB) containing 0.1% NP40, 0.1% Tween-20, and 0.01% Digitonin. Lysed nuclei were washed with 1 mL of cold ATAC-RSB containing 0.1% Tween-20. Isolated nuclei were immediately subjected to transposition using Tn5 transposase described previously [44]. Transposition reactions were carried out in a total volume of 50 µL containing 25 µL 2x Tagmentation buffer, 0.5 µL transposase (80nM final), 16.5 µL PBS, 0.5 µL 1% digitonin, 0.5 µL 10% Tween-20, 5 µL H_2O. The tagmentation reaction was incubated at 37°C for 30 min with gentle mixing. Following transposition, DNA was purified using Zymo DNA Clean and Concentrator-5 Kit (cat# D4014) according to the manufacturer's instructions.

Library amplification was performed using PCR with indexed primers described in [43] and NEBNext® High-Fidelity 2X PCR Master Mix (M0541L). The optimal number of amplification cycles was determined by quantitative PCR to minimize over-amplification. PCR was performed

involving an initial 5-min extension at 72°C to fill gaps, followed by 8-14 cycles (98°C for 10s, 63°C for 30s, 72°C for 30s). Amplified libraries were purified using AMPure XP beads and assessed for quality and fragment size distribution using an TapeStation. Libraries exhibiting the characteristic nucleosomal laddering pattern were sequenced on an Illumina platform (NovaSeq X Plus) using paired-end 150bp reads.

Paired-end 150-bp reads were collected for each sample. Nextera Transposase adapter sequences were removed using Cutadapt (v4.2) [45]. Trimmed reads were aligned to the human genome assembly hg38 using Bowtie2 (v2.5.2) [46] with the '--local' and '--no-discordant' options. Alignments with mapping quality scores < 7 were removed using SAMtools (v1.18) [47], and only properly paired reads were retained. Open chromatin regions were identified using MACS2 (v2.2.7.1) [33] with options: -f BAMPE -g hs -q 0.0001. Peaks across different conditions were merged to generate consensus peaks and HTSeq-count (v2.0.2) [48] was used to calculate fragment counts for the consensus peaks. The fragment count matrix was further normalized using DESeq2 (v1.40.1) [49] size factors, and the correlation analyses were performed using normalized count matrix in R environment (v4.3.2). ATACseqQC package (v1.34.0) [50] was used to calculate transcription start site (TSS) enrichment scores. Fragment size distributions and duplicates levels for each sample were calculated from BAM files using GenomicAlignments (v1.36.0) [51] and GenomicRanges (v1.52.0) [51] packages within the R/Bioconductor environment.

Coarse-grained polymer model

We simulate chromatin movement by using a bead-and-spring model of polymer chains. This coarse-grained polymer model was used to analyze how cohesin-mediated constraints influence chromatin movement during short microscopic observation times. In this model, the position of

each bead is represented by $\boldsymbol{r}_i$ for $i = 1, \cdots, 2n + 5$, where $n = 200$. Each bead corresponds to a coarse-grained 1-kb chromatin region, and the chain consists of two 200-kb domains ($i = 1, \cdots, n$ and $i = n + 6, \cdots, 2n + 5$), connected by a linker ($i = n + 1, \cdots, n + 5$). The temporal change of these positions is governed by the Brownian dynamics,

$$\frac{1}{\mu}\frac{d\boldsymbol{r}_i}{dt} = -\frac{\partial U}{\partial \boldsymbol{r}_i} + \boldsymbol{\xi}_i(t).$$

Here, $\boldsymbol{\xi}_i$ is a Gaussian random force, satisfying $\langle \boldsymbol{\xi}_i(t) \rangle = 0$ and $\langle \xi_{i\alpha}(t)\xi_{j\beta}(t') \rangle = (2k_{\mathrm{B}}T/\mu)\delta(t - t')\delta_{ij}\delta_{\alpha\beta}$, where k_{B} is the Boltzmann constant, T is temperature, and $\alpha, \beta = x, y,$ or z. The potential energy U consists of the following components,

$$U = \sum_i u_{\mathrm{spring}}(r_{ii+1}) + \sum_{(i,j)} u_{\mathrm{rep}}(r_{ij}) + \sum_{(i,j)\in G(t)} u_{\mathrm{assoc}}(r_{ij}) + \sum_{(i,j)\in L} u_{\mathrm{loop}}(r_{ij}),$$

with $r_{ij} = |\boldsymbol{r}_i - \boldsymbol{r}_j|$. Here, $u_{\mathrm{spring}}(r_{ii+1}) = \frac{K}{2}(r_{ii+1} - \sigma)^2$ represents the spring that connects the ith and $i + 1$th beads. $u_{\mathrm{rep}}(r_{ij}) = \varepsilon_{\mathrm{rep}}\left(1 - (r_{ij}/\sigma)^2\right)^3$ accounts for the soft, repulsive interaction between a pair of beads (i, j), with the sum taken over all pairs within the distance σ. $u_{\mathrm{assoc}}(r_{ij}) = -\varepsilon_{\mathrm{assoc}}/\left(1 + (r_{ij}/\sigma_{\mathrm{assoc}})^6\right)$ is the attractive interaction between beads (i, j) listed in a set $G(t)$. $u_{\mathrm{loop}}(r_{ij}) = u_{\mathrm{spring}}(r_{ij})$ represents the constraint imposed by cohesin, which connects beads (i, j) in a set L.

In the present simulation, attractive interactions between beads drive the condensation of chromatin, leading to the formation of cluster-like domains of beads, as observed in microscopy. These attractive interactions should depend on the structural characteristics of the nucleosome cluster represented by each bead, which vary stochastically. To account for this variability, we update the set $G(t)$ stochastically at each simulation step of the Brownian dynamics. To prevent instability caused by excessively dense attractive interactions, we introduce a valency parameter z, which represents the maximum number of attractive partner beads for each bead [52]. At each simulation step in the Brownian dynamics, we randomly select a pair (i, j) from those not already included in $G(t)$ and whose mutual distance r_{ij} is less than σ_{assoc}. We then add the selected pair to the set $G(t)$ at a rate k_{assoc} if the number of partners for either bead i or j does not exceed z. Conversely, a pair that is already included in $G(t)$ is removed from $G(t)$ at a rate k_{dissoc}. We consider nucleosomes in the linker chain to be either acetylated or depleted and refer to such an area as the acetylated region. We assume that a fraction φ of the chain within each domain also consists of acetylated regions. To determine the distribution pattern of the acetylated regions with the fraction φ and the average region size w, we generate a Markov chain of transitions between acetylated and deacetylated states along the chromatin chain. The distribution pattern is rejected and re-generated until the fraction of acetylated regions coincide with φ. We assign the value $z = 0$ to the linker region, $z = 1$ to the acetylated regions within each domain, and $z = 3$ to the deacetylated regions. Thus, acetylated regions were modeled as having reduced effective attractive capacity compared to deacetylated regions, consistent with weaker compaction propensity of acetylated chromatin.

The temporal variation of L depends on how cohesin interacts with the chromatin chain. In this study, we do not make specific assumptions about these interactions; instead, we focus on the

constraints that cohesin imposes during the observed short period (~0.5 s) of nucleosome movement. Therefore, we assume that L remains fixed for each chain we simulate. Cohesin is expected to bind more frequently in regions where transcription factors or CTCF molecules are enriched. We anticipate that nucleosomes in these regions are either acetylated or depleted. Because regions characterized by nucleosome depletion and acetylation are correlated and likely to overlap at the current 1-kb resolution, we randomly select $2m$ sites within the modeled acetylated regions of each domain as cohesin-bound sites and connect them with m loops under the condition that the loop length is larger than l_{min}. We generated 10 chains with varying acetylation patterns, defining a set L for each chain. Simulations were performed under two cohesin conditions: a loop-constrained condition (control; L defined as above, with five loops per 200-kb domain) and a cohesin-depleted condition (Δcohesin; $L = \emptyset$, i.e., no loop constraints). We consider two scenarios for generating L: one in which loops connect the randomly chosen $2m$ acetylated sites, allowing for mutual crossings of loops, and another in which loops are replaced until all loops are free of crossing. While crossings of loops may not occur under the loop-extrusion mechanism [53,54], they can happen through the loop-capture mechanism [55 56]. In Fig. 8 in the main text, loop crossing is prohibited. In Extended Data Fig. 10d-e, we compare two scenarios: one allowing loop crossing and the other prohibiting it. This comparison demonstrates that allowing loop crossings slightly enhances the looping effects on chromatin movement.

We define the parameter $\sigma = 30$–40 nm as a unit of spatial length, $\tau = 1$ s as a unit of time, and $k_{\mathrm{B}}T$ as a unit of energy. The parameters were set as $K = 100k_{\mathrm{B}}T/\sigma^2$, $\varepsilon_{\mathrm{rep}} = 20k_{\mathrm{B}}T$, $\varepsilon_{\mathrm{assoc}} = 5k_{\mathrm{B}}T$, and $\sigma_{\mathrm{assoc}} = 1.5\sigma$. The mobility parameter μ was chosen for the simulated MSD of the beads to align with the order of magnitude of the observed MSD, with $\mu = 200\sigma^2/(\tau k_{\mathrm{B}}T)$. We should note that $k_{\mathrm{B}}T$ is absorbed in the other parameters with this setting, allowing to put $k_{\mathrm{B}}T =$

1 without loss of generality. The rate of association of beads to form attractive pairs was set to approximate the frequency of diffusive movement of nucleosomes as $k_{\mathrm{assoc}} = 200\ \tau^{-1}$. We used $k_{\mathrm{dissoc}} = 50\ \tau^{-1}$, considering that the dissociation rate is lower in the crowded cellular environment than the value for the dissociation of attractive nucleosome pair observed in vitro [57]. We used the acetylation fraction $\varphi = 20\%$ and the average region size $w = 2$ kb. It was suggested that the distance between two cohesin molecules bound on chromatin is $30{\sim}70$ kb [58], and we assumed that the number of cohesin molecules bound on each 200-kb domain is $m = 5$ and set $l_{\mathrm{min}} = 30$ kb.

We conducted a simulation for each chain in an open boundary space. The initial conformation of the chain at $t = 0$ was generated as a random walk consisting of 40 consecutive steps. This structure was fine-grained by interpolating 40 positions with a third-order spline, resulting in 405 beads placed equidistantly along the curve. To monitor MSD, we imposed a constraint that the center of mass of the chain does not move, mimicking a scenario where the chain is gently confined within a finite space. The simulation time step was set to $\delta t = 10^{-5}\ \tau$, and both MSD and the mixing score were monitored over 5×10^{6}steps, following a relaxation run of 10^{7}steps. One trajectory was obtained with each of 10 chains having different acetylation and loop patterns, and the total 10 trajectories were used to analyze MSD and the mixing score in each scenario of simulations.

Mixing score: to quantify the spatial mixing between the two domains, we computed a mixing score $M = (R_{11} + R_{22})/R_{12}$, where R_{ab} is the average distance between beads in domain a and those in domain b: $R_{ab} = \frac{1}{N_0} \sum_{i \in a,\, j \in b} r_{ij}$. Here, r_{ij} is the distance between beads i and j, $N_0 =$

$n(n-1)$ for $a = b$, $N_0 = n^2$ for $a \neq b$, and $n = 200$ is the number of beads in each domain. With this definition, $M \approx 2$ indicates strong mixing (domains largely merged), whereas $M \approx 0.9$ indicates strong separation.

Intron-seqFISH probe selection and design

Candidate intron-seqFISH target genes were selected based on genome-wide transcriptional bursting parameters quantified in HCT116 cells, with an emphasis on genes showing no significant change in expression after acute RAD21 degradation [59]. To avoid regimes where co-burst differences become difficult to resolve, targets were further restricted to an intermediate bursting/expression range (mean expression 1–3 and burst frequency 0.0033–0.01). Because many genes meeting these criteria were located on chromosome 1, we prioritized chromosome 1 and computed pairwise genomic distances among candidates to maximize the number of analyzable gene pairs within tens of megabases (≤ 40 Mb).

To design intron-targeting primary oligonucleotides, we evaluated probe designability in PaintSHOP and, in practice, extracted intronic genomic intervals for each shortlisted gene and retrieved pre-designed oligonucleotides from a PaintSHOP "full probe file" (DNA-FISH oligo resource) that overlapped these intronic regions [60]. Genes with fewer than 15 available intronic probes were excluded to ensure robust detection sensitivity, yielding seven target genes (*BTF3L4, CAMTA1, PRPF38B, PTP4A2, RABGGTB, SYF2, and THRAP3*). For genes with many candidate probes, a maximum of 30 probes per gene were selected by prioritizing probes proximal to the transcription start site (TSS) and with favorable off-target/repeat metrics.

Probe selection was implemented as follows. Gene genomic coordinates and strand information were obtained via Ensembl BioMart export, and the TSS was defined as the gene start coordinate for + strand genes and the gene end coordinate for − strand genes. Candidate probes were ranked by the absolute distance between the probe midpoint and the TSS. Probes were first selected within ± 5 kb of the TSS; if fewer than 30 probes were obtained, the window was progressively expanded up to ± 50 kb, and if fewer than 30 probes were available even after expansion, all available probes were used. Candidate probes were filtered to prioritize probes with repeat_seq = 0 (relaxed to repeat_seq ≤ 1 if needed) and off_target = 0 (relaxed to off_target ≤ 1 if needed), with additional ranking based on sequence complexity (e.g., max_kmer) and melting temperature metrics as needed. To reduce local redundancy, a greedy thinning step enforcing a minimum probe–probe spacing of 150 bp was applied, after which 30 probes were retained when possible. To generate RNA-complementary probe sequences, probe sequences were oriented according to gene strand: because PaintSHOP outputs sequences corresponding to the genomic + strand, probes targeting + strand genes were reverse-complemented, whereas probes for − strand genes were used as provided (already matching the reverse-complement orientation relative to the transcribed sequence). Each final primary probe was constructed with a central gene-specific target-complementary sequence flanked by two copies of the secondary-probe binding sequence on both the 5′ and 3′ ends. The complete list of primary probe oligonucleotides, as well as corresponding secondary and readout probes, is provided in Supplementary Table 2. All probe pools were synthesized as oPools (Integrated DNA Technologies, IDT) and used for intron-seqFISH experiments.

Intron-seqFISH

Cell culture inserts (ibidi, 80209) were fitted on 24 mm × 50 mm No. 1S glass coverslips (thickness 0.16–0.19 mm; Matsunami) that were coated with poly-D-lysine (0.1 mg/mL; Thermo Fisher Scientific, A3890401). HCT116 CMV-OsTIR1(F74G) RAD21-mAID-mClover H2B-Halo cells were seeded at 1×10^4 cells per insert well in McCoy's 5A medium supplemented with 10% fetal bovine serum (FBS) and cultured overnight. Cells were then labeled with 100 nM JFX549-HaloTag ligand for 1 h at 37 °C, washed 3 times with fresh McCoy's 5A + 10% FBS, and allowed to recover for 1 h at 37 °C. For cohesin depletion, cells were incubated with 1 µM 5Ph-IAA or vehicle control for 3 h; two biological replicates were prepared for each condition. Cells were washed with D-PBS, fixed with 4% paraformaldehyde (PFA) for 10 min at room temperature, and washed in D-PBS (3 × 5 min). Samples were then exchanged into 70% ethanol and stored at -20°C prior to intron-seqFISH.

To enable repeated buffer exchange during sequential hybridization–imaging cycles, a thin flow chamber was assembled on the sample coverslip. Before flow-chamber assembly, ethanol was completely removed by aspiration and the samples were allowed to air-dry briefly at room temperature. A Fluorescent Friendly™ double-sided adhesive film (FL-S-2L; Grace Bio-Labs) was cut into a rectangular frame (24 mm × 50 mm) with a central elliptical aperture (major axis ≈ 22 mm, minor axis ≈ 19 mm; projected area ≈ 328 mm^2; film thickness 0.28 mm). One adhesive face was laminated onto the sample coverslip such that the specimen region was centered in the aperture. The opposite adhesive face was sealed with a custom cover glass (26 mm × 76 mm × 2 mm; Matsunami) bearing two circular access ports (Ø 1.3 mm, center-to-center spacing 18 mm) serving as inlet and outlet. This formed a leak-tight chamber (~0.28 mm internal height; ~92 µL volume) for sequential hybridization, washing, and imaging.

After rehydration in 2× SSC, samples were permeabilized with 0.5% Triton X-100 in PBS for 15 min at room temperature. Blocking was performed by incubating samples in PBS containing 10 mg/mL UltraPure bovine serum albumin (Invitrogen, AM2616), 0.3% Triton X-100, 0.1% dextran sulfate, and 0.5 mg/mL sheared salmon sperm DNA (Invitrogen, AM9680) for 15 min at room temperature. Samples were rinsed with 2× SSC three times.

Primary probe mixtures were prepared at 1 nM per probe in hybridization buffer consisting of 50% formamide, 10% dextran sulfate, and 2× SSC. Hybridization was performed at 37 °C for 72 h in a humidified chamber. Following hybridization, samples were washed in 55% formamide wash buffer (55% formamide, 2× SSC, 0.1% Triton X-100) for 30 min at room temperature, followed by three washes in 4× SSC.

Sequential imaging was performed as ten rounds of hybridization–washing–imaging. In each round, the sample was incubated with a target-specific secondary probe together with a universal Alexa Fluor 647-labeled readout oligonucleotide. Secondary and readout probes were diluted together in EC buffer (10% ethylene carbonate, 2× SSC) to final concentrations of 0.1 μM (secondary probe) and 0.4 μM (readout oligo) and hybridized for 20 min at room temperature. After each 20 min readout hybridization, excess probe was removed by three rinses in 4× SSC, followed by a brief wash in 4× SSC + 0.1% Triton X-100. Samples were then stringently washed in 12.5% wash buffer (12.5% formamide, 2× SSC, 0.1% Triton X-100) and rinsed three additional times in 4× SSC. The buffer was replaced with a DAPI staining solution (4× SSC, 0.1% Triton X-100, 5 μg/mL DAPI) and incubated for 1 min at room temperature. Finally, the chamber was filled with an anti-bleaching imaging buffer consisting of 50 mM Tris-HCl (pH 8.0), 4× SSC, 3 mM Trolox (Sigma-Aldrich, 238813), 10% D-(+)-glucose (Nacalai Tesque,

16806-25), catalase (Sigma-Aldrich, C3155; 1:100 dilution), and glucose oxidase (1 mg/mL) (Sigma-Aldrich, G2133).

Imaging was performed on a Nikon Ti-2 inverted microscope equipped with a Yokogawa CSU-W1 spinning-disk confocal scanner, a Plan Apo λD 60× oil-immersion objective (NA 1.42), and an ORCA-Fusion sCMOS camera (Hamamatsu Photonics), controlled by Nikon NIS-Elements. Excitation was provided by 405 nm, 561 nm, and 637 nm lasers (LightHUB+, Omicron). Z-stacks spanning 20 µm were acquired at 500 nm z-steps (41 optical sections), yielding a voxel size of 110 nm × 110 nm × 500 nm.

In the first cycle, imaging was performed for Alexa 647 (readout), HaloTag (JFX549), and DAPI. In subsequent cycles, imaging was performed for Alexa 647 (readout) and DAPI only. After imaging each round, the fluorophore-labeled readout oligo was stripped by incubation in 55% formamide stripping buffer (55% formamide, 2× SSC, 0.1% Triton X-100) for 15 min at room temperature, followed by rinsing in 4× SSC to remove formamide. The next round's readout oligo was then introduced, and the cycle was repeated.

Round order was: round 1 *BTF3L4*, round 2 no probe, round 3 *BTF3L4*, round 4 *CAMTA1*, round 5 *PRPF38B*, round 6 *PTP4A2*, round 7 *RABGGTB*, round 8 *SYF2*, round 9 *THRAP3*, round 10 no probe. "No-probe" rounds were included as negative controls to monitor residual signal and stripping efficiency. Round 03 repeated *BTF3L4* to validate round-to-round registration and detection reproducibility (see Extended Data Fig. 10h)

Raw images from all rounds were organized per field of view and channel. Image files for each round and field of view were imported into Fiji (ImageJ). For quality control and downstream segmentation, maximum-intensity projection (MIP) images were generated. After channel splitting, the readout (Alexa Fluor 647) and DAPI channels were re-merged into a two-channel stack, and all rounds were concatenated and converted into a 4D hyperstack (2 channels × Z × rounds).

Inter-round rigid drift was first corrected in Fiji using Correct 3D drift with the DAPI channel as the reference. After drift correction, stacks were cropped to a user-defined region of interest, and the Z-range containing the specimen was determined automatically from intensity profiles to exclude empty/zero-padded regions. The resulting drift-corrected stacks were exported as per-field-of-view TIFF files and used as input for subsequent deformable registration.
Deformable registration was performed in Python using FireANTs [61]. For deformation estimation, DAPI volumes were winsorized (clipped at the 0.5th and 99.5th percentiles) and rescaled to the [0,1] range. The DAPI volume from round 1 was used as the fixed reference, and each round was registered as a moving image using greedy optimization (cross-correlation similarity; kernel size 5) at a single resolution scale (factor 4; 200 iterations; Adam optimizer with learning rate 0.5) with Gaussian smoothing regularization of the deformation field (smooth_grad_sigma = 1.0; smooth_warp_sigma = 0.5). The resulting deformation field was then applied to both the unnormalized DAPI and readout (Alexa Fluor 647) volumes for each round.

Nuclei were segmented from DAPI maximum-intensity projections using Cellpose with the pretrained "nuclei" model. Resulting masks were inspected and manually corrected where needed before downstream quantification.

Nascent intronic RNA signals were detected from the registered Alexa 647 (FISH) volumes using bigFISH spot detection [62]. Spot detection was performed in 3D using the acquisition voxel size (110 × 110 × 500 nm) and an empirically selected spot radius in nanometers. Detected spots were optionally refined by subpixel localization. Spots were then assigned to nuclei using the nuclear masks. To reduce false positives and over-counting, intensity filtering was applied using empirically determined intensity thresholds, and for each gene in each cell, up to two brightest spots were retained to represent (at most) two alleles in a near-diploid cell line.

For each gene *A* and experimental group, the burst frequency was computed as:

$$F_A = \frac{N_b(A)}{N},$$

where N is the total number of analyzed cells in the group and $N_b(A)$ is the total number of detected nascent transcription spots (bursting sites) for gene *A* summed across all cells in that group.

For each gene pair (A, B), co-bursting was defined: a cell was classified as co-bursting if it contained at least one spot for gene A and at least one spot for gene B, and the minimum 3D distance between any A–B spot pair in that cell was ≤ 1 µm. The co-burst frequency was: co-burst frequency = N_{co}/N, where N_{co} is the number of co-bursting cells.

A normalized co-burst frequency was computed as:

$F_{co} = (N_{co}/N) / (F_A \cdot F_B)$.

For comparisons between conditions (e.g., RAD21 depleted vs control), ΔF_{co} was computed for each gene pair as the difference in F_{co} between groups.

Supplementary References

1. Yahata, K. *et al.* cHS4 insulator-mediated alleviation of promoter interference during cell-based expression of tandemly associated transgenes. *J Mol Biol* **374**, 580–90 (2007).
2. Yesbolatova, A. *et al.* The auxin-inducible degron 2 technology provides sharp degradation control in yeast, mammalian cells, and mice. *Nat Commun* **11**, 5701 (2020).
3. Emerson, D.J. *et al.* Cohesin-mediated loop anchors confine the locations of human replication origins. *Nature* **606**, 812–819 (2022).
4. Sakata, R. *et al.* Opening of cohesin's SMC ring is essential for timely DNA replication and DNA loop formation. *Cell Rep* **35**, 108999 (2021).
5. Bayle, J.H. *et al.* Rapamycin analogs with differential binding specificity permit orthogonal control of protein activity. *Chem Biol* **13**, 99–107 (2006).
6. Grunberg, R., Ferrar, T.S., van der Sloot, A.M., Constante, M. & Serrano, L. Building blocks for protein interaction devices. *Nucleic Acids Res* **38**, 2645–62 (2010).
7. Minami, K. *et al.* Replication-dependent histone labeling dissects the physical properties of euchromatin/heterochromatin in living human cells. *Sci Adv* **11**, eadu8400 (2025).
8. Laemmli, U.K. Cleavage of structural proteins during the assembly of the head of bacteriophage T4. *Nature* **227**, 680–5 (1970).
9. Maeshima, K. *et al.* Cell-cycle-dependent dynamics of nuclear pores: pore-free islands and lamins. *J Cell Sci* **119**, 4442–51 (2006).
10. Kimura, H. & Cook, P.R. Kinetics of core histones in living human cells: little exchange of H3 and H4 and some rapid exchange of H2B. *J Cell Biol* **153**, 1341–53 (2001).
11. Mito, Y., Henikoff, J.G. & Henikoff, S. Genome-scale profiling of histone H3.3 replacement patterns. *Nat Genet* **37**, 1090–7 (2005).
12. Goldberg, A.D. *et al.* Distinct factors control histone variant H3.3 localization at specific genomic regions. *Cell* **140**, 678–91 (2010).
13. Clement, C. *et al.* High-resolution visualization of H3 variants during replication reveals their controlled recycling. *Nat Commun* **9**, 3181 (2018).
14. Lewis, C.D. & Laemmli, U.K. Higher order metaphase chromosome structure: evidence for metalloprotein interactions. *Cell* **29**, 171–81 (1982).
15. Shimamoto, Y., Tamura, S., Masumoto, H. & Maeshima, K. Nucleosome-nucleosome interactions via histone tails and linker DNA regulate nuclear rigidity. *Mol Biol Cell* **28**, 1580–1589 (2017).
16. Semeigazin, A. *et al.* Behaviors of nucleosomes with mutant histone H4s in euchromatic domains of living human cells. *Histochem Cell Biol* (2024).
17. Nishiyama, T. *et al.* Sororin mediates sister chromatid cohesion by antagonizing Wapl. *Cell* **143**, 737–49 (2010).
18. Tinevez, J.Y. *et al.* TrackMate: An open and extensible platform for single-particle tracking. *Methods* **115**, 80–90 (2017).
19. Tinevez, J.-Y. *et al.* TrackMate: An open and extensible platform for single-particle tracking. *Methods* **115**, 80–90 (2017).
20. Eykelenboom, J.K. *et al.* Live imaging of marked chromosome regions reveals their dynamic resolution and compaction in mitosis. *J Cell Biol* **218**, 1531–1552 (2019).
21. Consortium, E.P. An integrated encyclopedia of DNA elements in the human genome. *Nature* **489**, 57–74 (2012).
22. Richardson, W.H. Bayesian-Based Iterative Method of Image Restoration*. *Journal of the Optical Society of America* **62**, 55–59 (1972).

23. Lucy, L.B. An iterative technique for the rectification of observed distributions. *The Astronomical Journal* **79**, 745–754 (1974).
24. Dey, N. *et al.* Richardson-Lucy algorithm with total variation regularization for 3D confocal microscope deconvolution. *Microsc Res Tech* **69**, 260–6 (2006).
25. Laasmaa, M., Vendelin, M. & Peterson, P. Application of regularized Richardson-Lucy algorithm for deconvolution of confocal microscopy images. *J Microsc* **243**, 124–40 (2011).
26. Ashwin, S.S., Nozaki, T., Maeshima, K. & Sasai, M. Organization of fast and slow chromatin revealed by single-nucleosome dynamics. *Proc Natl Acad Sci U S A* **116**, 19939–19944 (2019).
27. Ura, K. & Kaneda, Y. Reconstitution of chromatin in vitro. *Methods Mol Biol* **181**, 309–25 (2001).
28. Iida, S. *et al.* Single-nucleosome imaging reveals steady-state motion of interphase chromatin in living human cells. *Science Advances.* **8**, eabn5626 (2022).
29. Ide, S. *et al.* Telomere-specific chromatin capture using a pyrrole-imidazole polyamide probe for the identification of proteins and non-coding RNAs. *Epigenetics Chromatin* **14**, 46 (2021).
30. Gatto, A., Forest, A., Quivy, J.P. & Almouzni, G. HIRA-dependent boundaries between H3 variants shape early replication in mammals. *Mol Cell* **82**, 1909–1923 e5 (2022).
31. Ewels, P.A. *et al.* The nf-core framework for community-curated bioinformatics pipelines. *Nat Biotechnol* **38**, 276–278 (2020).
32. Ewels, P.A. *et al.* The nf-core framework for community-curated bioinformatics pipelines. *Zenodo.* (2022).
33. Zhang, Y. *et al.* Model-based analysis of ChIP-Seq (MACS). *Genome Biol* **9**, R137 (2008).
34. Nozaki, T. *et al.* Condensed but liquid-like domain organization of active chromatin regions in living human cells. *Sci Adv* **9**, eadf1488 (2023).
35. Rao, S.S.P. *et al.* Cohesin Loss Eliminates All Loop Domains. *Cell* **171**, 305–320 e24 (2017).
36. Kim, M. *et al.* Interplay between cohesin and RNA polymerase II in regulating chromatin interactions and gene transcription. *Nat Struct Mol Biol* (2026).
37. Dekker, J. *et al.* The 4D nucleome project. *Nature* **549**, 219–226 (2017).
38. Reiff, S.B. *et al.* The 4D Nucleome Data Portal as a resource for searching and visualizing curated nucleomics data. *Nat Commun* **13**, 2365 (2022).
39. Rao, S.S. *et al.* A 3D map of the human genome at kilobase resolution reveals principles of chromatin looping. *Cell* **159**, 1665–80 (2014).
40. Xiong, K. & Ma, J. Revealing Hi-C subcompartments by imputing inter-chromosomal chromatin interactions. *Nat Commun* **10**, 5069 (2019).
41. Davis, C.A. *et al.* The Encyclopedia of DNA elements (ENCODE): data portal update. *Nucleic Acids Res* **46**, D794–D801 (2018).
42. Stovner, E.B. & Saetrom, P. epic2 efficiently finds diffuse domains in ChIP-seq data. *Bioinformatics* **35**, 4392–4393 (2019).
43. Corces, M.R. *et al.* An improved ATAC-seq protocol reduces background and enables interrogation of frozen tissues. *Nat Methods* **14**, 959–962 (2017).
44. Xie, L. *et al.* 3D ATAC-PALM: super-resolution imaging of the accessible genome. *Nat Methods* **17**, 430–436 (2020).
45. Martin, M. Cutadapt removes adapter sequences from high-throughput sequencing reads. *2011* **17**, 3 (2011).

46. Langmead, B. & Salzberg, S.L. Fast gapped-read alignment with Bowtie 2. *Nat Methods* **9**, 357–9 (2012).
47. Li, H. *et al.* The Sequence Alignment/Map format and SAMtools. *Bioinformatics* **25**, 2078–9 (2009).
48. Anders, S., Pyl, P.T. & Huber, W. HTSeq--a Python framework to work with high-throughput sequencing data. *Bioinformatics* **31**, 166–9 (2015).
49. Love, M.I., Huber, W. & Anders, S. Moderated estimation of fold change and dispersion for RNA-seq data with DESeq2. *Genome Biol* **15**, 550 (2014).
50. Ou, J. *et al.* ATACseqQC: a Bioconductor package for post-alignment quality assessment of ATAC-seq data. *BMC Genomics* **19**, 169 (2018).
51. Lawrence, M. *et al.* Software for computing and annotating genomic ranges. *PLoS Comput Biol* **9**, e1003118 (2013).
52. Guha, S. Binder and monomer valencies determine the extent of collapse and reswelling of chromatin. *J Chem Phys* **162**(2025).
53. Fudenberg, G. *et al.* Formation of Chromosomal Domains by Loop Extrusion. *Cell Rep* **15**, 2038–49 (2016).
54. Mirny, L.A., Imakaev, M. & Abdennur, N. Two major mechanisms of chromosome organization. *Curr Opin Cell Biol* **58**, 142–152 (2019).
55. Guerin, T.M., Barrington, C., Pobegalov, G., Molodtsov, M.I. & Uhlmann, F. An extrinsic motor directs chromatin loop formation by cohesin. *EMBO J* **43**, 4173–4196 (2024).
56. Uhlmann, F. A unified model for cohesin function in sisterchromatid cohesion and chromatin loop formation. *Mol Cell* **85**, 1058–1071 (2025).
57. Kilic, S. *et al.* Single-molecule FRET reveals multiscale chromatin dynamics modulated by HP1alpha. *Nat Commun* **9**, 235 (2018).
58. Holzmann, J. *et al.* Absolute quantification of cohesin, CTCF and their regulators in human cells. *Elife* **8**(2019).
59. Trzaskoma, P. *et al.* 3D chromatin architecture, BRD4, and Mediator have distinct roles in regulating genome-wide transcriptional bursting and gene network. *Sci Adv* **10**, eadl4893 (2024).
60. Hershberg, E.A. *et al.* Author Correction: PaintSHOP enables the interactive design of transcriptome- and genome-scale oligonucleotide FISH experiments. *Nat Methods* **18**, 1265 (2021).
61. Jena, R., Chaudhari, P. & Gee, J.C. FireANTs: Adaptive Riemannian Optimization for Multi-Scale Diffeomorphic Matching. arXiv:2404.01249 (2024).
62. Imbert, A. *et al.* FISH-quant v2: a scalable and modular tool for smFISH image analysis. *RNA* **28**, 786–795 (2022).
