# Supplementary material for: Cohesin prevents local mixing of condensed euchromatic domains in living human cells
Source: Nat Genet. 2026 Sep 8;58(9):2335–49. doi: 10.1038/s41588-026-02736-2 (PMC13553310; doi:10.1038/s41588-026-02736-2)
Supplement: Supplementary file 2 [file 41588_2026_2736_MOESM2_ESM.pdf]

nature portfolio

Corresponding author(s): Kazuhiro Maeshima

Last updated by author(s): Jul 17, 2026

Reporting Summary

Nature Portfolio wishes to improve the reproducibility of the work that we publish. This form provides structure for consistency and transparency in reporting. For further information on Nature Portfolio policies, see our Editorial Policies and the Editorial Policy Checklist.

Statistics

For all statistical analyses, confirm that the following items are present in the figure legend, table legend, main text, or Methods section.

n/a	Confirmed	
☐	☒	The exact sample size (*n*) for each experimental group/condition, given as a discrete number and unit of measurement
☐	☒	A statement on whether measurements were taken from distinct samples or whether the same sample was measured repeatedly
☐	☒	The statistical test(s) used AND whether they are one- or two-sided<br>*Only common tests should be described solely by name; describe more complex techniques in the Methods section.*
☒	☐	A description of all covariates tested
☐	☒	A description of any assumptions or corrections, such as tests of normality and adjustment for multiple comparisons
☐	☒	A full description of the statistical parameters including central tendency (e.g. means) or other basic estimates (e.g. regression coefficient) AND variation (e.g. standard deviation) or associated estimates of uncertainty (e.g. confidence intervals)
☐	☒	For null hypothesis testing, the test statistic (e.g. *F*, *t*, *r*) with confidence intervals, effect sizes, degrees of freedom and *P* value noted<br>*Give P values as exact values whenever suitable.*
☐	☒	For Bayesian analysis, information on the choice of priors and Markov chain Monte Carlo settings
☒	☐	For hierarchical and complex designs, identification of the appropriate level for tests and full reporting of outcomes
☐	☒	Estimates of effect sizes (e.g. Cohen's *d*, Pearson's *r*), indicating how they were calculated

Our web collection on statistics for biologists contains articles on many of the points above.

Software and code

Policy information about availability of computer code

Data collection	Wide-field fluorescent images were acquired and processed with DeltaVision Elite or Ultra system (GE Healthcare) controlled by SoftWorx 7.X (GE Healthcare). Single-molecule imaging microscopy, SIM, and STORM microscopy were performed using NIS elements AR v5.30.03 software (Nikon).
Data analysis	All fluorescent images were analyzed by Fiji (ImageJ) (NIH, Schneider (2012); Schindelin (2012)). Single-molecule tracking was performed using TrackMate (Fiji plugin) (Tinevez (2017)) or MATLAB 2012b with packages including u-track v2.1.3 (Jaqaman (2008)), custom scripts written by Python 3.12. RL-algorythm is performed on Python. The analysis is briefly described in the Methods section. Detailed description is found in Ashwin et al. (2019). SIM images were analyzed using R (version 4.3.2). The software employed for DAPI or H3.3-Halo intensity classification (Fig. 6 and 7) is implemented in the R package nucim and the accompanying package bioimagetools. Additional information and development versions of both packages can be found on https:// bioimaginggroup.github.io/nucim and https://bioimaginggroup.github.io/bioimagetools. Detailed description is described by Schmid et al. (2017). All the analysis is briefly described in the Methods section and in detail as comments in the scripts deposited on Zenodo (https://doi.org/10.5281/zenodo.16959115 https://doi.org/10.5281/zenodo.18404051).

For manuscripts utilizing custom algorithms or software that are central to the research but not yet described in published literature, software must be made available to editors and reviewers. We strongly encourage code deposition in a community repository (e.g. GitHub). See the Nature Portfolio guidelines for submitting code & software for further information.

Data

Policy information about availability of data

All manuscripts must include a data availability statement. This statement should provide the following information, where applicable:

- Accession codes, unique identifiers, or web links for publicly available datasets
- A description of any restrictions on data availability
- For clinical datasets or third party data, please ensure that the statement adheres to our policy

The pull-down sequencing data are publicly available in the DDBJ BioProject database under accession numbers PRJDB17378 (HeLa H2B-HaloTag; SRA runs DRR527334–DRR527339; https://ddbj.nig.ac.jp/search/entry/bioproject/PRJDB17378/), PRJDB17380 (HeLa H3.3-HaloTag; SRA runs DRR527328–DRR527333; https://ddbj.nig.ac.jp/search/entry/bioproject/PRJDB17380/), and PRJDB39674 (HCT116 H2B-HaloTag and H3.3-HaloTag; SRA runs DRR801689–DRR801696; https://ddbj.nig.ac.jp/search/entry/bioproject/PRJDB39674/). ATAC-seq data have been deposited in GEO (GSE318385).

Research involving human participants, their data, or biological material

Policy information about studies with human participants or human data. See also policy information about sex, gender (identity/presentation), and sexual orientation and race, ethnicity and racism.

Reporting on sex and gender	N/A
Reporting on race, ethnicity, or other socially relevant groupings	N/A
Population characteristics	N/A
Recruitment	N/A
Ethics oversight	N/A

Note that full information on the approval of the study protocol must also be provided in the manuscript.

Field-specific reporting

Please select the one below that is the best fit for your research. If you are not sure, read the appropriate sections before making your selection.

☒ Life sciences ☐ Behavioural & social sciences ☐ Ecological, evolutionary & environmental sciences

For a reference copy of the document with all sections, see nature.com/documents/nr-reporting-summary-flat.pdf

Life sciences study design

All studies must disclose on these points even when the disclosure is negative.

Sample size	No statistical methods was used to predetermine the sample size. Sample size was based on similar studies in this field.
Data exclusions	No data was excluded.
Replication	At least 2 biological replicates were performed and 3 biological replicates were performed for most of the experiment.
Randomization	All cells for imaging were selected randomly, however, all cells that did not include experimental errors described above were analyzed equally, thus there was no requirement for randomization.
Blinding	No blinding was applied during data collection and analysis.

Reporting for specific materials, systems and methods

We require information from authors about some types of materials, experimental systems and methods used in many studies. Here, indicate whether each material, system or method listed is relevant to your study. If you are not sure if a list item applies to your research, read the appropriate section before selecting a response.

Materials & experimental systems

n/a	Involved in the study
☐	☒ Antibodies
☐	☒ Eukaryotic cell lines
☒	☐ Palaeontology and archaeology
☒	☐ Animals and other organisms
☒	☐ Clinical data
☒	☐ Dual use research of concern
☒	☐ Plants

Methods

n/a	Involved in the study
☐	☒ ChIP-seq
☒	☐ Flow cytometry
☒	☐ MRI-based neuroimaging

Antibodies

Antibodies used

1. anti-H2B;Abcam;rabbit;ab1790
2. anti-H3;Abcam;rabbit;ab1791
3. anti-HaloTag;Promega;mouse;G9211
4. anti-RAD21;Upstate;mouse;05-908
5. anti-γH2AX (phospho S139);Abcam;rabbit;ab2893
6. anti-CTCF;Millipore;mouse;07-729
7. anti-WAPL;Millipore;mouse;MABE1106
8. anti-Sororin (CDCA5);Abcam;rabbit;ab192237
9. anti-RNAPII CTD;Abcam;mouse;ab817
10. anti-acetyl H3;Millipore;rabbit;06-599
11. anti-acetyl H4;Millipore;rabbit;06-866
12. anti-laminA/C (636);Santa Cruz;mouse;sc-7292
13. anti-RNAPII Ser5P; from Hiroshi Kimura Lab (Science Tokyo);mouse;Pa57B7
14. anti-RNAPII Ser2P;from Hiroshi Kimura Lab (Science Tokyo);mouse;Pc26B5
15. anti-H3K27me3;from Hiroshi Kimura Lab (Science Tokyo);mouse;AB_2819243
16. anti-H3K9me3;from Hiroshi Kimura Lab (Science Tokyo);mouse;AB_2819245
17. anti-rabbit-HRP;Bio-Rad;goat;170-6515
18. anti-mouse-HRP;Bio-Rad;goat;170-6516
19. anti-rabbit Alexa594;Invitogen;goat;A11037
20. anti-rabbit Alexa647;Invitogen;goat;A21245
21. anti-mouse Alexa594;Invitogen;goat;A11032
22. anti-mouse Alexa647;Invitogen;goat;A21236
23. anti-mouse Alexa488;Invitogen;goat;A11029

Validation

All the commercially available antibody (1-12 and 17-23) has been validated by the manufacturer for use as stated on their product page. Antibodies from Hiroshi Kimura Lab (13-16) were validated by previous reports (Nagashima et al., 2019, Hayashi-Takanaka Y. et al., 2011).

Eukaryotic cell lines

Policy information about cell lines and Sex and Gender in Research

Cell line source(s)

HeLa S3 cells (Maeshima et al. (2006))
HCT116 cells (Yesbolatova et al. (2020))
HT1080 cells (Eykelenboom et al. 2019)

Authentication

N/A

Mycoplasma contamination

Cell lines used in this study were tested to be negative for mycoplasma contamination using DNA staining.

Commonly misidentified lines
(See ICLAC register)

N/A

Plants

Seed stocks

Report on the source of all seed stocks or other plant material used. If applicable, state the seed stock centre and catalogue number. If plant specimens were collected from the field, describe the collection location, date and sampling procedures.

Novel plant genotypes

Describe the methods by which all novel plant genotypes were produced. This includes those generated by transgenic approaches, gene editing, chemical/radiation-based mutagenesis and hybridization. For transgenic lines, describe the transformation method, the number of independent lines analyzed and the generation upon which experiments were performed. For gene-edited lines, describe the editor used, the endogenous sequence targeted for editing, the targeting guide RNA sequence (if applicable) and how the editor was applied.

Authentication

Describe any authentication procedures for each seed stock used or novel genotype generated. Describe any experiments used to assess the effect of a mutation and, where applicable, how potential secondary effects (e.g. second site T-DNA insertions, mosiacism, off-target gene editing) were examined.

ChIP-seq

Data deposition

☒ Confirm that both raw and final processed data have been deposited in a public database such as GEO.

☐ Confirm that you have deposited or provided access to graph files (e.g. BED files) for the called peaks.

Data access links
May remain private before publication.

The genomics data have been deposited with the link to BioProject accession number PRJDB17380 (HeLa H3.3-HaloTag pull-down), and PRJDB39674 (HCT116 H2B-HaloTag and H3.3-HaloTag pull-downs) in the DDBJ BioProject database.

Files in database submission

(Sample Name), (Description)
PRJDB17380
H3.3_biotin_input_rep1, input control for HeLaS3 H3.3-Halo (replicate #1)
H3.3_biotin_pull-down_rep1, HaloTag-ChIP-seq for HeLaS3 H3.3-Halo (replicate #1)
H3.3_DMSO_pull-down_rep1, negative control for HeLaS3 H3.3-Halo (replicate #1)
H3.3_biotin_input_rep2, input control for HeLaS3 H3.3-Halo (replicate #2)
H3.3_biotin_pull-down_rep2, HaloTag-ChIP-seq for HeLaS3 H3.3-Halo (replicate #2)
H3.3_DMSO_pull-down_rep2, negative control for HeLaS3 H3.3-Halo (replicate #2)

PRJDB39674
H3.3_biotin_input_rep1, input control for HCT116 H3.3-Halo (replicate #1)
H3.3_biotin_pull-down_rep1, HaloTag-ChIP-seq for HCT116 H3.3-Halo (replicate #1)
H3.3_biotin_input_rep2, input control for HCT116 H3.3-Halo (replicate #2)
H3.3_biotin_pull-down_rep2, HaloTag-ChIP-seq for HCT116 H3.3-Halo (replicate #2)
H2B_biotin_input_rep1, input control for HCT116 H2B-Halo (replicate #1)
H2B_biotin_pull-down_rep1, HaloTag-ChIP-seq for HCT116 H2B-Halo (replicate #1)
H2B_biotin_input_rep12, input control for HCT116 H2B-Halo (replicate #2)
H2B_biotin_pull-down_rep2, HaloTag-ChIP-seq for HCT116 H2B-Halo (replicate #2)

Genome browser session
(e.g. UCSC)

N/A

Methodology

Replicates

Reproducibility was confirmed with the two replicates.

Sequencing depth

All samples were sequenced in pair-end with the length of 100 bp. The total number of reads is given below.
(Sample), (Read Length), (Total number of reads), (Uniquely mapped reads)
PRJDB17380
H3.3_biotin_input_rep1, 100 bp (paired-end), 38 839 924, 35 289 348
H3.3_biotin_pull-down_rep1, 100 bp (paired-end), 43 521 152, 40 804 608
H3.3_DMSO_pull-down_rep1, 100 bp (paired-end), 40 004 270, 36 661 664
H3.3_biotin_input_rep2, 100 bp (paired-end), 34 387 530, 31 316 388
H3.3_biotin_pull-down_rep2, 100 bp (paired-end), 41 557 244, 38 984 406
H3.3_DMSO_pull-down_rep2, 100 bp (paired-end), 40 299 550, 36 910 118

PRJDB39674
H3.3_biotin_input_rep1, 100 bp (paired-end), 28077136, 23863583
H3.3_biotin_pull-down_rep1, 100 bp (paired-end), 25777387, 21336469
H3.3_biotin_input_rep2, 100 bp (paired-end), 29142620, 22812251
H3.3_biotin_pull-down_rep2, 100 bp (paired-end), 25960103, 20533893
H2B_biotin_input_rep1, 100 bp (paired-end), 22070224, 21587030
H2B_biotin_pull-down_rep1, 100 bp (paired-end), 22788743, 19245551
H2B_biotin_input_rep12, 100 bp (paired-end), 24777985, 25552926
H2B_biotin_pull-down_rep2, 100 bp (paired-end), 29334921, 19947916

Antibodies	H3.3-Halo-incorporated nucleosomes were labeled with 1 μM HaloTag PEG-biotin ligands (G859A; Promega) and pulled down using streptavidin magnetic beads (TAS8848 N1170; Tamagawa-Seiki). See Methods for details.
Peak calling parameters	Peaks were detected using the broad mode of MACS2.
Data quality	Reads were quality controlled and trimmed using fastqc and Trim_Galore. The PCR duplicates were processed using Picard PCR duplicate removal.
Software	The nf-core ChIP-seq pipeline (nfcore/chipseq: version 2.0.0), employing a Docker configuration profile with the default parameters. Peaks were detected using the broad mode of MACS2 (version 2.2.7.1).
