# Supplementary material for: Cohesin prevents local mixing of condensed euchromatic domains in living human cells
Source: Nat Genet. 2026 Sep 8;58(9):2335–49. doi: 10.1038/s41588-026-02736-2 (PMC13553310; doi:10.1038/s41588-026-02736-2)
Supplement: Supplementary file 3 [file 41588_2026_2736_MOESM3_ESM.pdf]

nature portfolio

Peer Review File

Cohesin prevents local mixing of condensed euchromatic domains in living human cells

Corresponding Author: Professor Kazuhiro Maeshima

Version 0:

Decision Letter:

3rd Oct 2025

Dear Professor Maeshima,

Your Article, "Cohesin prevents local mixing of condensed euchromatic domains in living human cells" has now been seen by 3 referees. You will see from their comments copied below that while they find your work of considerable potential interest, they have raised quite substantial concerns that must be addressed. In light of these comments, we cannot accept the manuscript for publication, but would be very interested in considering a revised version that addresses these serious concerns.

Reviewer#1 appreciates the study but asks for a major revision. They have specific guidance for improvement.

Reviewer #2 thinks the use of microscopy is elegant and the paper deserves to be considered for publication, but asks for clarifications and additional analyses.

Reviewer #3 thinks the study is conceptually relevant, but has various concerns. They think that the lack of functional work with regards to transcription is a major limitation of the study.

We hope you will find the referees' comments useful as you decide how to proceed. If you wish to submit a substantially revised manuscript, please bear in mind that we will be reluctant to approach the referees again in the absence of major revisions.

To guide the scope of the revisions, the editors discuss the referee reports in detail within the team, including with the chief editor, with a view to identifying key priorities that should be addressed in revision and sometimes overruling referee requests that are deemed beyond the scope of the current study. In this case, please address reviewers' requests in full. We hope that you will find the prioritised set of referee points to be useful when revising your study. Please do not hesitate to get in touch if you would like to discuss these issues further.

If you choose to revise your manuscript taking into account all reviewer and editor comments, please highlight all changes in the manuscript text file. At this stage we will need you to upload a copy of the manuscript in MS Word .docx or similar editable format.

We are committed to providing a fair and constructive peer-review process. Do not hesitate to contact us if there are specific requests from the reviewers that you believe are technically impossible or unlikely to yield a meaningful outcome.

If revising your manuscript:

*1) Include a “Response to referees” document detailing, point-by-point, how you addressed each referee comment. If no action was taken to address a point, you must provide a compelling argument. This response will be sent back to the referees along with the revised manuscript.

*2) If you have not done so already please begin to revise your manuscript so that it conforms to our Article format instructions, available here.
Refer also to any guidelines provided in this letter.

*3) Include a revised version of any required Reporting Summary: https://www.nature.com/documents/nr-reporting-

summary.pdf
It will be available to referees (and, potentially, statisticians) to aid in their evaluation if the manuscript goes back for peer review.
A revised checklist is essential for re-review of the paper.

Please be aware of our guidelines on digital image standards.

EXTENDED DATA FIGURES

When re-submitting your manuscript, please ensure that any supplementary figures and tables that are crucial to the manuscript's conclusions are converted into Extended Data figures and tables to increase visibility of these data. Extended Data figures and tables are online-only (present in the online PDF and full-text HTML versions of the paper), peer-reviewed display items that provide essential background to the article but are not included in the main article due to space constraints. A maximum of ten Extended Data display items (figures and tables) is permitted.

You may use the link below to submit your revised manuscript and related files:

Link Redacted

Note: This URL links to your confidential home page and associated information about manuscripts you may have submitted, or that you are reviewing for us. If you wish to forward this email to co-authors, please delete the link to your homepage.

If you wish to submit a suitably revised manuscript we would hope to receive it within 6 months. If you cannot send it within this time, please let us know. We will be happy to consider your revision so long as nothing similar has been accepted for publication at Nature Genetics or published elsewhere. Should your manuscript be substantially delayed without notifying us in advance and your article is eventually published, the received date would be that of the revised, not the original, version.

Please do not hesitate to contact me if you have any questions or would like to discuss the required revisions further.

Nature Genetics is committed to improving transparency in authorship. As part of our efforts in this direction, we are now requesting that all authors identified as 'corresponding author' on published papers create and link their Open Researcher and Contributor Identifier (ORCID) with their account on the Manuscript Tracking System (MTS), prior to acceptance. ORCID helps the scientific community achieve unambiguous attribution of all scholarly contributions. You can create and link your ORCID from the home page of the MTS by clicking on 'Modify my Springer Nature account'. For more information please visit please visit www.springernature.com/orcid.

Thank you for the opportunity to review your work.

Sincerely,
Chiara

Chiara Anania, PhD
Associate Editor
Nature Genetics
https://orcid.org/0000-0003-1549-4157

Referee expertise:

Referee #1: 3D genome architecture, super-resolution live-cell imaging

Referee #2: cohesin, chromosomal architecture

Referee #3: transcription, nuclear organization, single-molecule tracking

Reviewers' Comments:

Reviewer #1 (Remarks to the Author):

In this paper, Shiori Iida and collaborators, under the guidance of Kazuiro Maeshima, addressed with imaging-based biophysics methods the role of cohesin loop extrusion in euchromatin condensates. The main discovery of this research is that euchromatin appears to form condensates that are relatively structured and less "open" than expected, and that this organization is due to cohesin loop extrusion. They describe for the first time that loop extrusion organizes the condensed state of euchromatin. This finding goes against the classic textbook vision. However, even if this might appear contradictory, the discovery is in line with trends we have been observing in recent years from microscopy-based approaches using living samples instead of fixed ones, where new evidence often challenges "textbook" models and suggests refined alternatives.

The Maeshima lab is a recognized expert in chromatin imaging, and the results of this work deserve to be published in a journal with a broad readership.
The paper is written in excellent language. That said, before publication, I would gently suggest a few edits and pose some questions and suggest potential experiments that, if addressed, could increase both the clarity and the robustness of the study.

1. The question in the abstract is broader than what is truly addressed. More specifically, the main focus of the paper is: “To explore how cohesin organizes chromatin domains in living human cells, we..”
2. Consequently, the conclusion in the abstract may be too strong. For example, “Using this nanoscopic approach, we revealed that euchromatin is organized into condensed domains constrained by cohesin-mediated loops, challenging the established textbook view of euchromatin as largely open.” seems to oversell the contribution, as this is not the first paper describing euchromatin condensation. A more balanced phrasing might be: “we revealed that euchromatin condensed domains are contrained by cohesin-mediated loops,” which is consistent with prior work they also cite as “7,8,10,20-22.”
3. Figure references should be carefully revised: S1a appears mis-referenced (it concerns RAD21 depletion), and S1c seems not referenced.
4. The authors “tracked in 2D at 50 ms/frame for 15–20 s, yielding ~1400 trajectories per cell” but MSD analysis spans only 0.0 to 0.5 seconds. Could they clarify this choice? In particular, recent research highlights the importance of covering at least two orders of magnitude (see https://www.science.org/doi/full/10.1126/science.1216142 https://www.biorxiv.org/content/10.1101/2025.05.10.653248v1.full). A double-log plot might reveal a plateau. It would also help to report the exponent explicitly and explain whether longer-time data change the interpretation. In this case the authors seem to focus between the delta of different treatments but it would be interesting to understand why they decided to omit most of the tracks.
5. Please elaborate on how MSDs and alpha exponents were calculated.
6. Regarding the lacO described in Figure 2: is the Cohesin peak dependent on a CTCF site? It would be useful to report whether a CTCF binding site is present, its orientation, and whether a CTCF chromatin peak is available.
7. The HT1080 tetO/lacO system is valuable but underutilized. Here I am wondering, what is the contribution of the stalled cohesin? This experimental system could be used to strengthen the conclusion about the contribution of loop extrusion. Given the proximity of the lacO array to the Cohesin peak, including MSD measurements of tetO vs. lacO sites in WAPL and CTCF depletion with siRNA conditions could strengthen the argument that it is extruding cohesin, rather than stalled cohesin, that drives the observed effects. Despite rare, such analysis might also distinguish “looped alleles” from “non-looped alleles.”
8. This additional experiment in “7” could support another part of their paper: “CTCF loss did not affect nucleosome motion (Figs. 3f–h, S3f,g),” but what about nucleosomes positioned in proximity to cohesin peaks? the lacO locus could be a proxy to study the CTCF contribution (with a siRNA experiments) of nucleosomes in proximity to stalled cohesin sites. Since fully closed cohesin loops are rare and short-lived, this experiment might require the acquisition of many tracks, but within this, it could be possible to identify the two different subpopulation after CTCF depletion (this could be high risk since it could require complex data analysis that go beyond the scope of this paper, still it could be a huge addition).
9. Please provide quantitative protein reduction levels for siRNA knockdowns (CTCF, WAPL, Sororin) via western blotting, not only immunofluorescence.
10. The authors claim “implying that cohesin constrains movements of nucleosomes mainly in euchromatin.” is intriguing but may be premature. Since “Super-slow” and “Slow” trajectories are only presumably heterochromatin (as they also state), stronger evidence (e.g., perturbing H1 or laminB) would help.
11. Figure 5 shows strong evidence for euchromatin, but does not exclude similar roles in heterochromatin. Testing H1-Halo could be informative.
12. Supplemental figures S5a and S5b would benefit from clearer labeling and inclusion of uncut membranes with ladders.
13. Figure S7b shows small differences at the nuclear periphery after RAD21 depletion. Was alpha calculated for these experiments? It would be interesting to see the extent of the delta between different classes.
14. The statement “These results demonstrate that cohesin primarily constrains euchromatic regions.” may be too strong, as conclusions apply only to lamina-associated domains. Acknowledging constitutive vs. facultative heterochromatin differences would be helpful.
15. H3.3 is enriched but not exclusive to euchromatin. It is also observed in facultative heterochromatin (Banaszynski, L. auraA. et al. Hira-dependent histone H3.3 deposition facilitates PRC2 recruitment at developmental loci in ES cells. Cell 155, 107–120 (2013)., ang, H.-T. et al. Global H3.3 dynamic deposition defines its bimodal role in cell fate transition. Nat. Commun. 9, 1537 (2018).). Please acknowledge this nuance.
16. Figures 6 and related analyses would benefit from clarification: number of cells analyzed, logic behind signal density as a measure of condensation, quantification of “frequently” in cohesin localization, and clearer explanation of DAPI class definitions. Cell cycle synchronization effects should also be discussed.
17. The two-point MSD analysis is elegant but could be expanded:
   a. “Two-point MSD analysis showed much greater motion between neighboring H3.3-Halonucleosomes in living cells than in FA-fixed cells (Fig. 7i),” but what about unrelated nucleosomes? In double-stained H3.3-Halo experiments, do unrelated nucleosomes behave similarly to neighboring ones?
   b. Could the same analysis be done in the tetO/lacO line?
   c. Could this be repeated with H2B to compare euchromatin vs. general chromatin?
   d. How long after RAD21 depletion were experiments performed? Would longer depletion change results further? It would be interesting to allow chromatin to mix even more and observe if the two-point MSD of neighboring nucleosomes starts to behave like unrelated nucleosomes.
18. Please clarify how “same-domain” nucleosomes were identified, beyond proximity alone.
19. The conclusion “suggesting a transition to a more liquid-like state of chromatin.” is compelling, but it may also reflect loss

of tethering. It would be helpful to contemplate alternative explanations, such as an “unrestricted state of the chromatin polymer”
20. Finally, please be cautious in generalizing. Since experiments were performed in HCT116 and HeLa cells, the statement that “euchromatin is condensed in living human cells” should be phrased more carefully, noting possible cell-type–specific differences.

Reviewer #1 (Remarks on code availability):

The code is well annotated with the README with instructions for the "Trackmate_multiplefile".
However, the twopoint_MSD.zip does not have a README with instructions. Before publications I strongly suggest to include this as well.

Reviewer #2 (Remarks to the Author):

In the manuscript ‘Cohesin prevents local mixing of condensed euchromatic domains in living human cells’ the authors broadcast a high level of imaging, which gives a nice peek in the nucleus. The authors show how the movement of nucleosomes is dependent on the binding of cohesin to chromatin. In particular, the loop extruding cohesin is crucial for the movement. The second part of the paper goes more in depth on the different populations of nucleosomes, differences between H2B and H3.3 bound nucleosomes and how they are affected by cohesin binding.

The paper contains state-of-the-art imaging techniques, however for me it is difficult to interpret the differences in measurements as currently presented. I would like to see more intuitive figures to understand the data before I would say the manuscript is ready for publication in Nature Genetics. The finding that cohesin is involved in the nucleosome movement might be expected from their localisation, however to see this experimentally with very elegant microscopy is interesting for the field. Figure 6 does not add too much as it has been merely already shown by Miron et al., I think the paper would benefit it there is more focus and to explain figure 7 to the bigger scientific field. By focussing more on this one point the manuscript would be more appealing for a broader audience.

If my points are taken into account I would consider publishing this manuscript in Nature Genetics.

Major points:

In figure 1e the authors show the MSD plots of H2B nucleosomes. There is a differences between the cohesin depleted and not depleted. A similar measurement is shown in figure 1i, however the two graphs are identitical to me, except for the number that is written below this. The authors than claim there is a significant differences between the two. How would a difference plot between the two look like? With these plots it is impossible for the reader to assess the data and you need to believe the number below.

In figure 3b the authors show a depletion of WAPL, it has been previously reported that this will lead to ‘Vermicelli’ in the cohesin staining (Tedeschi et al.). Do the authors observe this? It is not visible in the example pictures.

Figure 3d,h and k are again identical.

In figure 3i,j,k the authors try to assess whether cohesin translocation or active loop extrusion by cohesin is required for the chromatin constraint. They use a head-tethered cohesin system, this did not change the nucleosome motion. This is quite a difficult experiment to interpret and to make such strong conclusions.
First I think it is required to make a timeline to explain the reader how the experiment is executed.
Secondly, with the fusion the cohesin is stuck on the DNA, similar to WAPL-depletion, in which it can also be discussed how much active loop extrusion is still ongoing when the cohesin rings are trapped at CTCF sites. Why does the fusion not give a similar result to WAPL depletion?
The reason for the addition of RAD21 depletion on top is not clear to me. Do the authors think SMC1 and SMC3 are than still fused to the chromatin or is the complex not intact at all and nothing is present on the chromatin? I would assume the second, it is just the same as in figure 1.
But I really don’t understand how the authors can claim here it is not active loop extrusion but translocation? The cohesin complex is too complex to simply say we fuse it here and see what nucleosome do.
Looking at the effect of loop extrusion inside cells is until now very difficult, an option would be to deplete NIPBL once the cells are in G2, however it will not only effect loop extrusion but also cohesin loading which will affect the nucleosome movement.

In figure 4 d the authors show 4 different subpopulations of nucleosome movement. Upon depletion of cohesin not only the fast population becomes faster also there is a clear difference in this plot in the height of the super-slow fraction which here only reaches to 2.5 while with cohesin it almost reaches 3. Also the area under the curve in the fast population seems bigger than in the DMSO control. However the authors show in figure 4h no differences in population, could the authors explain this?

The authors show that histone 3.3 is more enriched in euchromatin and underpin this with the histone marks from published ChIPs. However the histone 3 k27me3, mark shows the opposite and they don't show ChIP results for H3 k9me3. Is it known that H3.3 does not have these repressive marks, which indicate heterochromatin domains?

In figure 5, I'm missing a ChIP for cohesin here. Is cohesin enriched at the (borders of these) domains of which they say to be affected by depletion of cohesin? A thorough analysis of this would be informative.

Reviewer #3 (Remarks to the Author):

In this manuscript titled "Cohesin prevents local mixing of condensed euchromatic domains in living human cells", Iida et al investigate how cohesion influences the mobility of nucleosome scale chromatin dynamics and the organization of active chromatin in live human cells. The authors use single molecule tracking of H2B, H3.3 and SIM imaging of H3.3, RAD21, RNAPII and DAPI along with depletion or knockdown of RAD21, Sororoin, WAPL, and CTCF. Their primary conclusion is that euchromatin is organized into condensed domains constrained by cohesin, loss of which leads to "fluidization" and local mixing of euchromatin domains without compaction.
The study is highly relevant and important for understanding how local genome organization has functional implications in gene regulation. The conclusions are largely in line with previous reports on the effects of cohesin loss on chromatin mobility but the main potential conceptual advance of "mixing" is underdeveloped. This is because there is a lack of mechanistic connection between "mixing", loop extrusion and transcriptional activity. Several key conclusions also lack appropriate validation because of technical concerns, data analysis, lack of appropriate controls (most importantly with regards to effects on transcription).
Major Comments:
The presentation of data is confusing, in most cases just MSDs are presented. Although it seems that the MSDs were fit inferred alpha values and diffusion coefficients are not presented appropriately with error analyses/statistics. It is unclear if the exponent increasing from 0.43 to 0.48 is significant even more so with a lack of what distributions of values are obtained across multiple replicates. The authors should report fold-changes in alpha values and diffusion coefficients with confidence intervals, and emphasize biological relevance over statistical over-powering.
The authors should consider alternative analyses to MSD which is not appropriate to analyze SMT data as it has been shown in several publications (e.g. Hansen et al., elife, 2028) that it is error prone especially when there are mixtures of diffusion states. The authors should consider analyzing their data using fitting of displacement distributions or other alternatives to determine if there is indeed a change in diffusion coefficients. The authors can also calculate metrics like radius of gyration.
-Regarding figure 4, the authors should consider overlaid analysis with heterochromatin markers such as HP1 to be confident that changes are restricted to euchromatic regions. As in the above comment, why aren't diffusion coefficients calculated and presented instead of "Peak MSD values" this is confusing, the small shifts here are hard to interpret.
The H3.3 single molecule experiments should also be presented as MSDs along with fitted values and compared to H2B statistically, it is hard to compare these results to the rest of the paper. Where does the H3.3 data lie compared to different mobility classes referred to in figure 4?
Anisotropy analysis on chromatin bound molecules is not typically performed because of the issues with calculating angles at small displacements. The authors should report errors on calculating angles, and what is the error acoss samples in calculating the AC value. It would also be important to determine the AC value at multiple time and space scales. It is unclear if a change in the AC of -1.218 to -1.095 is meaningful and the authors should comment on what it means biologically. The anisotropy distributions as presented are hard to read, the authors should consider representing them as a standard line graph/histograms that are overlaid so changes can be more easily assessed.
It is clear from the single molecule movies that 50ms exposure time does not fully exclude non chromatin bound H2B. The authors have a few options to explore potential error from this, one is to subsample their movies averaging frames to create synthetic long exposure time data and perform analysis on that. Second the authors could repeat a few measurements at longer exposure times. Third the authors could repeat data at even faster frame rates and analyze the full population so that they can assess the contributions of chromatin incorporated and potentially non-incorporated histones. The third option may be the best as it is important to determine whether perturbations to Cohesin, CTCF, WAPL etc. influence nucleosome incorporation, this should also be addressed biochemically.
As mentioned in the above comment the authors should first validate that the chromatin-incorporated fraction of H2B/H3.3 is not affected by RAD21 degradation to prove that enhanced mobility is due to change in chromatin motion rather than due to increased contribution from non-chromatinized H2B/H3.3. Based on the raw movie provided at 50ms, Halo dye molecules outside the nucleus are also clearly visible as distinct spots raising concerns that the authors are actually detecting only the low mobility chromatinized H2B at 50 ms exposure. In addition to using faster imaging as suggested above the can also use a NLS-HaloTag control to show that free molecules are not tracked at 50 ms.
Common quality control metrics should be presented for all SMT data, not just one experiment, including localization precisions, detections per frame, track length distributions.
The movies are quite dense, from even the presented videos that show tracking several errors are visible. Given the low mobility of histones this may not be a huge concern but some error analysis should be presented.
The RAD21 depletion should be followed by time-course depletion vs mobility experiment rather only 1 hour time point. Do the effects become prominent over time?
Cohesin loss often leaves transcription surprisingly unaffected in the short term (e.g., Rao et al., 2017). If cohesin loss

increases euchromatic domain mixing, does this affect transcription? The authors should discuss why domain mixing might be primarily a biophysical effect without immediate transcriptional consequences. Ideally, include nascent RNA (EU-seq/PRO-seq) or ATAC-seq under the same perturbations
What does mixing mean? How is this quantified? A consistent metric needs to be developed and applied and tested rigorously. For example loss of boundary insulation, increased collisions across domains.
The authors refer to all cohesin activity as “loop formation”, it would be more technically accurate to simply refer to it as extrusion as loop formation itself is not tested or quantified in this manuscript. The authors should consider connecting their measurements to a polymer model with and without cohesin activity, several such models have been developed over the years. For example by explicitly connecting mixing to extrusion. Polymer simulations incorporating loop extrusion, with and without cohesin, could be compared to both MSD plots and a mixing index.
The effects of the AP21967 treatment need to be assessed, the authors should show controls that extrusion is effectively inhibited.
-The claim that local compaction of H3.3 is unchanged upon RAD21 depletion should be validated with higher resolution imaging such as DNA PAINT, STORM that has up to ~20 nm resolution rather than simply from chromatin classes quantification.
Authors conclude local mixing of euchromatin domains upon RAD21 depletion without direct quantification. Does RAD21 depletion make domains bigger in size without density changes? DNA PAINT is well suited to answer that. Also, does this have functional consequence such as co-expression of genes from neighboring domains? Can authors test this? The lack of functional analysis with regards to transcription is a major shortcoming of this manuscript.
Authors conclude Pol II localization on domain surfaces without proper quantification. Authors need to perform more rigorous and quantitative analysis on the spatial mapping of RNA Pol II (and elongating and initiating forms) and euchromatin domains which also could be perhaps better answered with two color DNA PAINT of H3.3 and Pol II variants. How does this spatial relation change upon triptolide and alpha- amanitin treatment?

Minor Comments:
Multiple groups (e.g., Hansen et al., Science 2022; Wutz et al., Nature 2017) have shown cohesin loss increases mobility and reduces loop structures. The novelty here is the “euchromatic domain mixing” angle. The authors should position their findings relative to these. Is “mixing” essentially another readout of weakened insulation, or does it capture a new phenomenon at the nucleosome scale?
Can authors explain the higher variability in the locus tracking measurement ? Why is the MSD shown only up to 0.5s for these since these loci could potentially be tracked for much longer ?
RAD21 loss significantly increases H3.3 mobility in the nuclear interior but has little effect on peripheral H2B. This may be due to technical limits in dense chromatin rather than true invariance. The authors should discuss this caveat explicitly and consider additional assays to support the biological interpretation.
Abstract:“Physical properties remain elusive” is a bit vague please specify which physical properties.
Introduction:
Line 6> genome -> genomic
Page 6, line 7: loop formation and extrusion are not necessarily the same, the former being rare. The authors should be careful on which they are attributing changes to.

Reviewer #3 (Remarks on code availability):

Code is well commented, documented, and is executable.

Version 1:

Decision Letter:

13th Mar 2026

Dear Prof. Maeshima,

Your Article, entitled "Cohesin prevents local mixing of condensed euchromatic domains in living human cells", has now been seen by the 3 original referees. You will see from their comments below that while reviewers #1 and #2 are satisfied overall, some important points are raised by reviewer #3. We are interested in the possibility of publishing your study in Nature Genetics, but would like to consider your response to these concerns in the form of a revised manuscript before we make a final decision on publication.

We therefore invite you to revise your manuscript taking into account all reviewer comments. Please highlight all changes in the manuscript text file. At this stage we will need you to upload a copy of the manuscript in MS Word .docx or similar editable format.

We are committed to providing a fair and constructive peer-review process. Do not hesitate to contact me if there are specific requests from the reviewers that you believe are technically impossible or unlikely to yield a meaningful outcome.

When revising your manuscript:

*1) Include a “Response to referees” document detailing, point-by-point, how you addressed each referee comment. If no

action was taken to address a point, you must provide a compelling argument. This response will be sent back to the referees along with the revised manuscript.

*2) If you have not done so already please begin to revise your manuscript so that it conforms to our Article format instructions, available
here.
Refer also to any guidelines provided in this letter.

*3) Include a revised version of any required Reporting Summary: https://www.nature.com/documents/nr-reporting-summary.pdf
It will be available to referees (and, potentially, statisticians) to aid in their evaluation if the manuscript goes back for peer review.
A revised checklist is essential for re-review of the paper.

Please be aware of our guidelines on digital image standards.

EXTENDED DATA FIGURES

When re-submitting your manuscript, please ensure that any supplementary figures and tables that are crucial to the manuscript's conclusions are converted into Extended Data figures and tables to increase visibility of these data. Extended Data figures and tables are online-only (present in the online PDF and full-text HTML versions of the paper), peer-reviewed display items that provide essential background to the article but are not included in the main article due to space constraints. A maximum of ten Extended Data display items (figures and tables) is permitted.

Please use the link below to submit your revised manuscript and related files:

Link Redacted

Note: This URL links to your confidential home page and associated information about manuscripts you may have submitted, or that you are reviewing for us. If you wish to forward this email to co-authors, please delete the link to your homepage.

Please do not hesitate to contact me if you have any questions or would like to discuss these revisions further.

Nature Genetics is committed to improving transparency in authorship. As part of our efforts in this direction, we are now requesting that all authors identified as 'corresponding author' on published papers create and link their Open Researcher and Contributor Identifier (ORCID) with their account on the Manuscript Tracking System (MTS), prior to acceptance. ORCID helps the scientific community achieve unambiguous attribution of all scholarly contributions. You can create and link your ORCID from the home page of the MTS by clicking on 'Modify my Springer Nature account'. For more information please visit please visit www.springernature.com/orcid.

We look forward to seeing the revised manuscript and thank you for the opportunity to review your work.

Sincerely,

Tiago

Tiago Faial, PhD
Chief Editor
Nature Genetics
https://orcid.org/0000-0003-0864-1200

Reviewers' Comments:

Reviewer #1 (Remarks to the Author):

The authors addressed all the major points and improved the text with relevant details and the graphs with informative data, enabling a better understanding of their conclusions. The additional work done to address all reviewers' comments further improved the already high quality of this manuscript.
The extra experiments I suggested would indeed be a valuable improvement, but I agree with the authors' reply. Even if they would provide good advice for future experiments, they are not required to support the current conclusions, and their absence does not preclude publication of this work.
My only remaining concern is that the updated figures S2 and S3 would benefit from quantification of acute protein depletion

from the Western blot analysis to demonstrate consistency with the fluorescence measurements. Currently, they provide only a qualitative visual representation in a single replicate. Once this is addressed, I strongly recommend publication of this article.

Reviewer #1 (Remarks on code availability):

Now the code description is complete.

Reviewer #2 (Remarks to the Author):

In the revised manuscript of Shimazoe et al., 'Cohesin prevents local mixing of condensed euchromatic domains in living human cells' the authors show the effect of loop extrusion by cohesin on the movement of euchromatin nucleosomes. They observe cohesin restricts nucleome mixing and thereby affects transcription, as transcription is normally mainly at the borders of the domains created by cohesin. They find that this is due to the loop extruding pool of cohesin and not the cohesive cohesin. The tracking of nucleosomes is of high quality and performed elegantly. The authors took my remarks into account and answered them accordingly. Conclusions are toned down and mutants are explained in the context of literature. Also the use of models helps the reader to understand the manuscript. I think the manuscript improved and is now more accessible to the broader public, therefore I support publication of the manuscript.

Reviewer #3 (Remarks to the Author):

The authors have done well in addressing many of my concerns. However, most of the concerns about causality and mechanism have mostly been addressed by reframing and softening claims. While the paper is technically strong in many aspects, a direct mechanistic link between cohesin's extrusion activity, to mixing, and transcription is still not demonstrated. In my opinion the current data can support the conclusion of cohesin-dependence on the physical properties of H3.3 rich domains but does not support mechanistic conclusions on domain mixing and transcription insulation. Thus overall, the paper is convincing on phenomenology rather than mechanism and is thus perceived as model-forward.

-The increased co-bursting despite small per-gene expression changes is interesting and the mixing defined in context of Hi-C changes, polymer modelling and FISH analysis is a far improved mechanistic connection than in the original submission. That being said, the intron-seqFISH results are relevant but are selective and indirect, genes are chosen with minimal per-gene changes and then enhanced co-bursting is shown. This data supports the weakened insulation but does not address the concern that mixing is the primary driver versus the changes being a correlated consequence of cohesin loss. So, the result supports altered coordination but doesn't directly support a functional consequence of domain mixing. The jump from increased chromatin motion to domain mixing, to transcription insulation is still not fully convincing.
-Regarding the head-tethering experiment. The authors decide to tone down their claims and clarify the comparison to the WAPL deletion. This is correct but the mechanism is not resolved. The experiment does not resolve extrusion vs. other cohesin-dependent constraints which was the concern. The sororin result rules out the role for sister chromatic cohesion, but the head tethering does not isolate extrusion, so the data doesn't justify the claim that extrusion is necessary. The conceptual implication seems to extend beyond what the experiments show.

- Statistics across replicates are still not shown in the revised manuscript for any figures. The RC and AC changes do not seem meaningful given the errors. It is unclear how significance is being or what the cell to cell or replicate to replicate variability is. The relatively changes in all SMT derived parameters are still hard to assess as being significant, there is no discussion of why this is a meaningful change it is still just reported as large.

-The authors use genomics to show that H3.3 is mostly present in euchromatin. However, the recommendation was to perform imaging which is more relevant to the rest of the single cell analysis in the paper. I still recommend an imaging control as suggested to show the relative exclusion from heterochromatin regions by imaging. I think such an experiment showing the extent of spatial colocalization with heterochromatin markers would make it clear what confounding effects there may be in the analysis presented.

One other concern is that the Hi-C and ATAC evidence that is presented is integrated from prior datasets rather than in the same pipeline as is applied in this study, the limitations of this cross analysis and potential caveats are not discussed.

Version 2:

Decision Letter:

29th Apr 2026

Dear Professor Maeshima,

Your Article, "Cohesin prevents local mixing of condensed euchromatic domains in living human cells" has now been seen

by 1 referee. You will see from their comments below that while they find your work of interest, some remaining points regarding the statistical analyses performed are raised. We are interested in the possibility of publishing your study in Nature Genetics, but would like to consider your response to these concerns in the form of a revised manuscript before we make a final decision on publication.

To guide the scope of the revisions, the editors discuss the referee reports in detail within the team, including with the chief editor, with a view to identifying key priorities that should be addressed in revision and sometimes overruling referee requests that are deemed beyond the scope of the current study. In this case, please address Reviewer #3's request in full. We hope that you will find the prioritized set of referee points to be useful when revising your study. Please do not hesitate to get in touch if you would like to discuss these issues further.

We therefore invite you to revise your manuscript taking into account all reviewer and editor comments. Please highlight all changes in the manuscript text file. At this stage we will need you to upload a copy of the manuscript in MS Word .docx or similar editable format.

We are committed to providing a fair and constructive peer-review process. Do not hesitate to contact us if there are specific requests from the reviewers that you believe are technically impossible or unlikely to yield a meaningful outcome.

When revising your manuscript:

*1) Include a “Response to referees” document detailing, point-by-point, how you addressed each referee comment. If no action was taken to address a point, you must provide a compelling argument. This response will be sent back to the referees along with the revised manuscript.

*2) If you have not done so already please begin to revise your manuscript so that it conforms to our Article format instructions, available
here.
Refer also to any guidelines provided in this letter.

*3) Include a revised version of any required Reporting Summary: https://www.nature.com/documents/nr-reporting-summary.pdf
It will be available to referees (and, potentially, statisticians) to aid in their evaluation if the manuscript goes back for peer review.
A revised checklist is essential for re-review of the paper.

Please be aware of our guidelines on digital image standards.

EXTENDED DATA FIGURES

When re-submitting your manuscript, please ensure that any supplementary figures and tables that are crucial to the manuscript’s conclusions are converted into Extended Data figures and tables to increase visibility of these data. Extended Data figures and tables are online-only (present in the online PDF and full-text HTML versions of the paper), peer-reviewed display items that provide essential background to the article but are not included in the main article due to space constraints. A maximum of ten Extended Data display items (figures and tables) is permitted.

Please use the link below to submit your revised manuscript and related files:

Link Redacted

Note: This URL links to your confidential home page and associated information about manuscripts you may have submitted, or that you are reviewing for us. If you wish to forward this email to co-authors, please delete the link to your homepage.

We hope to receive your revised manuscript within four to eight weeks. If you cannot send it within this time, please let us know.

Please do not hesitate to contact me if you have any questions or would like to discuss these revisions further.

Nature Genetics is committed to improving transparency in authorship. As part of our efforts in this direction, we are now requesting that all authors identified as ‘corresponding author’ on published papers create and link their Open Researcher and Contributor Identifier (ORCID) with their account on the Manuscript Tracking System (MTS), prior to acceptance. ORCID helps the scientific community achieve unambiguous attribution of all scholarly contributions. You can create and link your ORCID from the home page of the MTS by clicking on ‘Modify my Springer Nature account’. For more information please visit please visit www.springernature.com/orcid.

We look forward to seeing the revised manuscript and thank you for the opportunity to review your work.

Sincerely,
Chiara

Chiara Anania, PhD
Associate Editor
Nature Genetics
https://orcid.org/0000-0003-1549-4157

Reviewers' Comments:

Reviewer #3 (Remarks to the Author):

I appreciate the authors' efforts to add disclaimers and limitations statements that make clear that a direct mechanism in support of the proposed model is not established in this manuscript.

I remain concerned, however, about how the statistics are reported, as the newly supplied table still does not show replicate-level variability. What is explicitly missing is statistical analysis across biological replicates rather than statistics calculated across pooled cells.

Was each experiment performed only once, with 30 cells measured from that single experiment? If not, the relevant metrics should be calculated for each independent experiment and the statistics should be performed across those experiments. If only one experiment was performed, then it should be repeated, as cells collected within the same experiment cannot be treated as independent biological replicates.

This issue is especially important given the statistical tests presented in Supplementary Table 1 and the relatively small differences being reported. For the two-sided K-S test, many cells are pooled, and the test is highly sensitive to small shifts in the distribution. It therefore does not address the replicate-level issue. At most, it shows that the pooled distributions differ, but not whether the effect is robust across independent biological replicates.

Similarly, the Welch's t test appears to have been performed at the cell level rather than at the biological replicate level. Cells from the same experiment are not independent biological replicates. As a result, the reported p values may appear much stronger than they would if replicate-to-replicate variability were properly taken into account.

A replicate-aware statistical analysis should be provided for Rc, AC, and all other SMT-derived metrics across all experimental conditions.

Version 3:

Decision Letter:

Our ref: NG-A70127R2

25th Jun 2026

Dear Dr. Maeshima,

Thank you for submitting your revised manuscript "Cohesin prevents local mixing of condensed euchromatic domains in living human cells" (NG-A70127R2). It has now been seen by the original referees and their comments are below. The reviewers find that the paper has improved in revision, and therefore we'll be happy in principle to publish it in Nature Genetics, pending minor revisions to satisfy the referees' final requests and to comply with our editorial and formatting guidelines.

If the current version of your manuscript is in a PDF format, please email us a copy of the file in an editable format (Microsoft Word or LaTex)-- we can not proceed with PDFs at this stage.

We are now performing detailed checks on your paper and will send you a checklist detailing our editorial and formatting requirements soon. Please do not upload the final materials and make any revisions until you receive this additional information from us.

Thank you again for your interest in Nature Genetics Please do not hesitate to contact me if you have any questions.

Congratulations!

Sincerely,
Chiara

Chiara Anania, PhD
Associate Editor

Nature Genetics
https://orcid.org/0000-0003-1549-4157

Reviewer #3 (Remarks to the Author):

The author's have addressed my concerns adequately. My only recommendation is that in the final version of the paper the authors should clarify in the Methods or figure legends what constituted a biological replicate, how many cells were analyzed per replicate, and why a paired statistical test was appropriate for each comparison.

INTERNAL USE ONLY AIP modules required: ["Clinical","Life Science"]

Open Access This Peer Review File is licensed under a Creative Commons Attribution 4.0 International License, which permits use, sharing, adaptation, distribution and reproduction in any medium or format, as long as you give appropriate credit to the original author(s) and the source, provide a link to the Creative Commons license, and indicate if changes were made.
In cases where reviewers are anonymous, credit should be given to 'Anonymous Referee' and the source.
The images or other third party material in this Peer Review File are included in the article's Creative Commons license, unless indicated otherwise in a credit line to the material. If material is not included in the article's Creative Commons license and your intended use is not permitted by statutory regulation or exceeds the permitted use, you will need to obtain permission directly from the copyright holder.
To view a copy of this license, visit https://creativecommons.org/licenses/by/4.0/

We would like to thank the editor and the three reviewers for their insightful and critical comments. We believe that we have improved the quality of our manuscript by constructively responding to their suggestions and criticisms. Our point-by-point responses are provided below.

Reviewer #1 (Remarks to the Author):

In this paper, Shiori Iida and collaborators, under the guidance of Kazuhiro Maeshima, addressed with imaging-based biophysics methods the role of cohesin loop extrusion in euchromatin condensates. The main discovery of this research is that euchromatin appears to form condensates that are relatively structured and less “open” than expected, and that this organization is due to cohesin loop extrusion). They describe for the first time that loop extrusion organizes the condensed state of euchromatin. This finding goes against the classic textbook vision. However, even if this might appear contradictory, the discovery is in line with trends we have been observing in recent years from microscopy-based approaches using living samples instead of fixed ones, where new evidence often challenges “textbook” models and suggests refined alternatives. The Maeshima lab is a recognized expert in chromatin imaging, and the results of this work deserve to be published in a journal with a broad readership. The paper is written in excellent language. That said, before publication, I would gently suggest a few edits and pose some questions and suggest potential experiments that, if addressed, could increase both the clarity and the robustness of the study.

We are very grateful for Reviewer #1’s efforts and for his/her support and comments, which were helpful in improving our paper.

1. The question in the abstract is broader than what is truly addressed. More specifically, the main focus of the paper is: “To explore how cohesin organizes chromatin domains in living human cells, we..”

We agree with Reviewer #1 and have modified the corresponding part as follows:
"However, how cohesin organizes these domains in living cells, especially in active euchromatin, remains elusive."

2. Consequently, the conclusion in the abstract may be too strong. For example, “Using this nanoscopic approach, we revealed that euchromatin is organized into condensed domains constrained by cohesin-mediated loops, challenging the established textbook view of euchromatin as largely open.” seems to oversell the contribution, as this is not the first paper describing euchromatin condensation. A more balanced phrasing might be: “we revealed that euchromatin condensed domains are constrained by cohesin-mediated loops,” which is consistent with prior work they also cite as “7,8,10,20-22.”

We agree with Reviewer #1 and modified the corresponding part as follows:
"Using this nanoscopic approach, we revealed that euchromatin forms condensed domains that

are constrained by cohesin-mediated loops. This organization refines the classical textbook view of euchromatin as largely open, in line with emerging evidence."

"Abstract
The human genome is folded into chromatin loops by the cohesin complex, forming functional chromatin domains that underlie transcription and DNA replication/repair. However, how cohesin organizes these domains in living cells, especially in active euchromatin, remains elusive. To address these questions, we combined single-nucleosome imaging/tracking and super-resolution 3D-structured illumination microscopy (3D-SIM) with euchromatin-specific labeling of histone H3.3. Using this nanoscopic approach, we revealed that euchromatin forms condensed domains that are constrained by cohesin-mediated loops. This organization refines the classical textbook view of euchromatin as largely open, in line with emerging evidence. Transcription machinery appears to be located near the condensed domain surfaces/borders. Cohesin loss increased nucleosome fluidity of these domains without altering their overall compaction, leading to local mixing of domains and compromising transcriptional insulation. These findings uncover an unexpected physical role of cohesin in maintaining the integrity of condensed euchromatic domains and ensuring proper higher-order regulation of gene expression."

3. Figure references should be carefully revised: S1a appears mis-referenced (it concerns RAD21 depletion), and S1c seems not referenced.

We thank the reviewer for pointing this out. Following the suggestion, we revised the Fig. S1 legend and corrected the figure references. In addition, we changed the order of Fig. S1b and Fig. S1c to make the figure more consistent with the text.

4. The authors "tracked in 2D at 50 ms/frame for 15–20 s, yielding ~1400 trajectories per cell" but MSD analysis spans only 0.0 to 0.5 seconds. Could they clarify this choice? In particular, recent research highlights the importance of covering at least two orders of magnitude (see https://www.science.org/doi/full/10.1126/science.1216142 https://www.biorxiv.org/content/10.1101/2025.05.10.653248v1.full). A double-log plot might reveal a plateau. It would also help to report the exponent explicitly and explain whether longer-time data change the interpretation. In this case the authors seem to focus between the delta of different treatments but it would be interesting to understand why they decided to omit most of the tracks.

We appreciate Reviewer #1 for pointing out this important issue. First, we would like to clarify that "15–20 s" refers to the recording time for a single cell, not the tracking duration of individual nucleosomes. Due to photobleaching, the actual tracking time of each nucleosome is much shorter, typically only a few seconds.

We agree that longer tracking can, in principle, provide additional information. To also address Reviewer #3's comment, we have added longer-time data, up to ~3 s, and the Rc value (radius of constraint) (Dion and Gasser, 2013) in Fig. S1g.

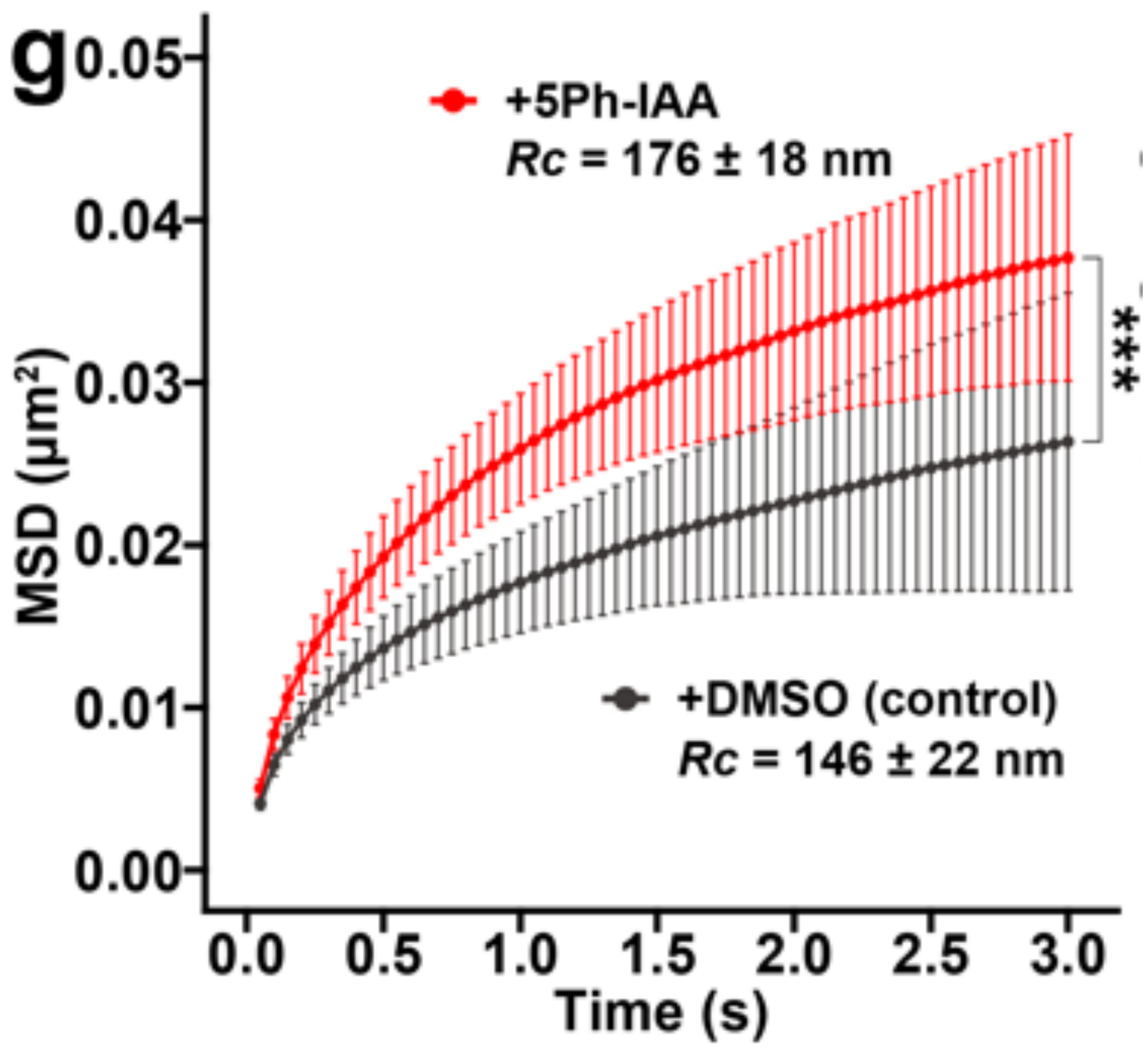

We mainly focused on the 0–0.5 s time window, which corresponds to the spatial range of typical chromatin domain sizes (100–300 nm), because of the following reason:
At longer time scales, other factors, such as nuclear movements, become more influential. Longer-time data, up to ~3 s, were also shown in Fig. 1f of our previous paper, Iida et al. (Iida et al., 2022). As further demonstrated in Fig. 1g of that study, a log–log MSD plot showed that local nucleosome motion within the first 0.5 s followed a power law with an exponent of ~0.45. Beyond 0.5 s, the plot could not be fitted linearly, indicating a different motion regime, presumably reflecting contributions from higher-order nuclear organization (Shinkai et al., 2016). Therefore, to study nucleosome motion within chromatin domains accurately, we mainly focused on the 0–0.5 s time scale. We have now clarified this point explicitly in the Methods section：

“Single-nucleosome tracking analysis

To study nucleosome motion within chromatin domains accurately, we mainly focused on the 0–0.5 s time window, which corresponds to the spatial range of typical chromatin domain sizes (up to ~300 nm). At longer time scales, other factors, such as higher-order structures (e.g., compartments, territories) and nuclear movements, become more influential (for details, see (Iida et al., 2022; Shinkai et al., 2017)). To obtain the *Rc* value (radius of constraint) (Dion and Gasser, 2013), we also analyzed longer-time data, up to ~3 s, in some cases.”

5. Please elaborate on how MSDs and alpha exponents were calculated.

We have now added a clear description of how MSDs and anomalous exponents were calculated in the Methods section, including the equation and fitting range:

“For single-nucleosome movement analysis, the displacement and MSD of the fluorescent dots were calculated based on their trajectory using a Python script. In our tracking, trajectories of single nucleosome dots on the XY plane $(\boldsymbol{x}_i)_{i=0}^{N}$ were acquired, where $\boldsymbol{x}_i$ indicates XY-coordinates at the time point i. Then, we calculated the MSD for the lag time Δ by:

$$MSD(lag\ time) = \frac{1}{N-\Delta}\sum_{i=1}^{N-\Delta}|x_i - x_{i+\Delta}|^2 \quad (1)$$

The originally calculated MSD was in 2D. To obtain the 3D value, the 2D value was multiplied by 1.5 (4 to 6 Dt). The calculated MSDs were fitted to a subdiffusive model $MSD(t) = 6D \cdot t$^α, where α is an anomalous diffusion exponent (0 < α < 1). Statistical analyses of the obtained single-nucleosome MSD captured through each histone protein were performed using Python.

The anomalous exponents (α) were obtained from the slope of the log–log MSD plots by linear regression within the 0–0.5 s time window.”

6. Regarding the lacO described in Figure 2: is the Cohesin peak dependent on a CTCF site? It would be useful to report whether a CTCF binding site is present, its orientation, and whether a CTCF chromatin peak is available.

We appreciate Reviewer #1’s comment regarding the possible role of CTCF at the lacO site. Eykelenboom et al. reported that a CTCF ChIP peak is indeed present near the cohesin peak (Eykelenboom et al., 2019). However, when CTCF binding sites were deleted, cohesion at the lacO locus (measured by fused LacI-GFP dots) was not reduced. Thus, the observed cohesion at this locus does not appear to depend on CTCF (Eykelenboom et al., 2019). We therefore do not pursue CTCF further here. For clarification, we have added an arrow indicating a putative cohesion site near the lacO array, but not near the tetO array.

7. The HT1080 tetO/lacO system is valuable but underutilized. Here I am wondering, what is the contribution of the stalled cohesin? This experimental system could be used to strengthen the conclusion about the contribution of loop extrusion. Given the proximity of the lacO array to the Cohesin peak, including MSD measurements of tetO vs. lacO sites in WAPL and CTCF depletion with siRNA conditions could strengthen the argument that it is extruding cohesin, rather than stalled cohesin, that drives the observed effects. Despite rare, such analysis might also distinguish “looped alleles” from “non-looped alleles.”

We appreciate Reviewer #1’s suggestion regarding the use of the HT1080 tetO/lacO system to further dissect the contribution of stalled versus extruding cohesin. In our current study, our main aim in using this system was to show that nucleosome motion near a cohesion site is more constrained, as evidenced by the increased mobility upon Sororin knockdown. Although ChIP signals of cohesin and CTCF are present near the lacO site, previous work (Eykelenboom et al., 2019) suggests that these sites are not essential for cohesion itself. We agree that a deeper analysis of the role of stalled versus extruding cohesin at this locus would be of interest. However, we feel that pursuing such an investigation would divert the focus of the current manuscript, which is to establish the general principle that cohesin constrains condensed

euchromatin domains. To avoid potential confusion for readers, we have therefore chosen not to extend this line of analysis further here.

8. This additional experiment in "7" could support another part of their paper: "CTCF loss did not affect nucleosome motion (Figs. 3f–h, S3f,g)," but what about nucleosomes positioned in proximity to cohesin peaks? the lacO locus could be a proxy to study the CTCF contribution (with a siRNA experiments) of nucleosomes in proximity to stalled cohesin sites. Since fully closed cohesin loops are rare and short-lived, this experiment might require the acquisition of many tracks, but within this, it could be possible to identify the two different subpopulation after CTCF depletion (this could be high risk since it could require complex data analysis that go beyond the scope of this paper, still it could be a huge addition).

We greatly appreciate Reviewer #1's excellent suggestion to use the lacO/tetO system as a proxy to study the contribution of CTCF at stalled cohesin sites. However, in the HT1080 cells, both LacI-GFP and TetR-mCherry are highly expressed to label the lacO and tetO arrays, respectively. This results in very high background fluorescence that interferes with the detection of the much weaker single-nucleosome signals. Unfortunately, this makes it technically unfeasible to combine nucleosome single-molecule imaging with this system. We therefore could not pursue this otherwise interesting direction in the present study.

9. Please provide quantitative protein reduction levels for siRNA knockdowns (CTCF, WAPL, Sororin) via western blotting, not only immunofluorescence.

We agree with Reviewer #1's suggestion and performed western blot analyses for CTCF, WAPL, and Sororin to quantify the knockdown efficiency, as shown in Figs. S2, S3.

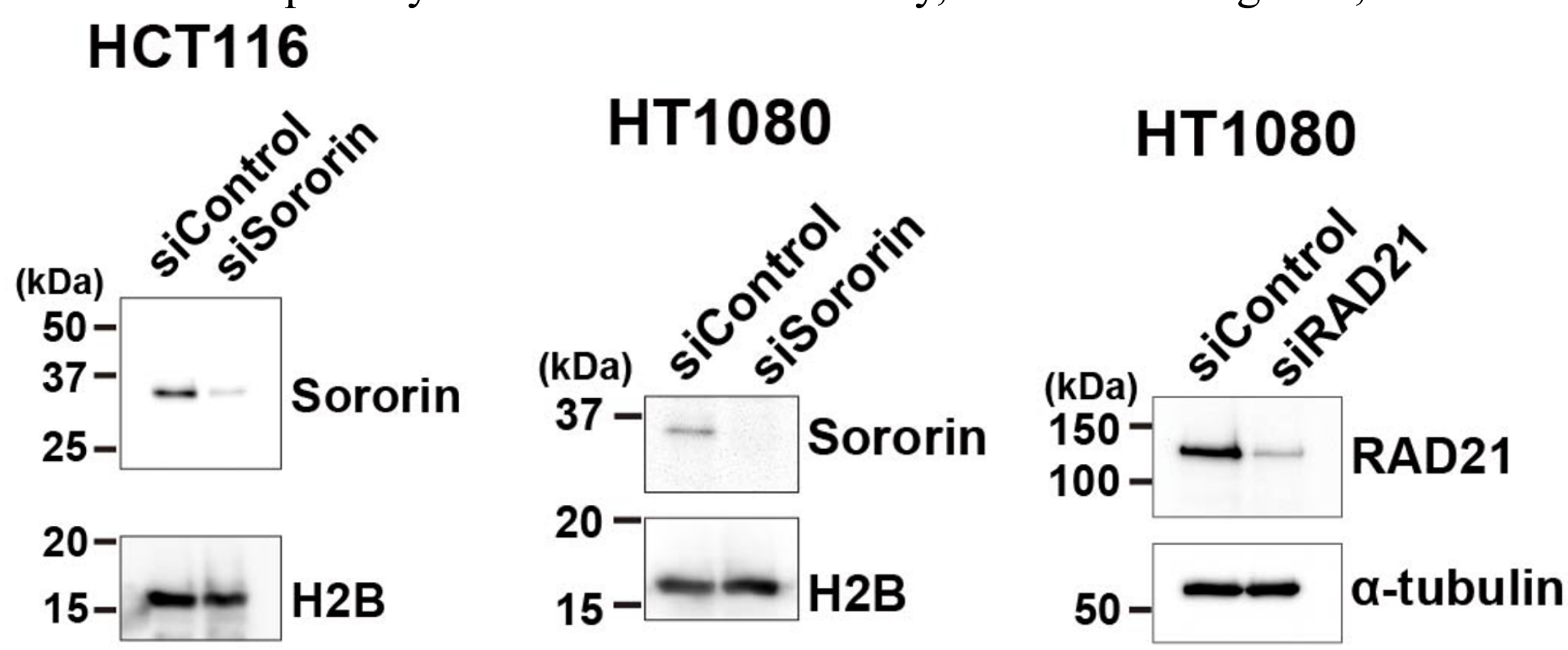

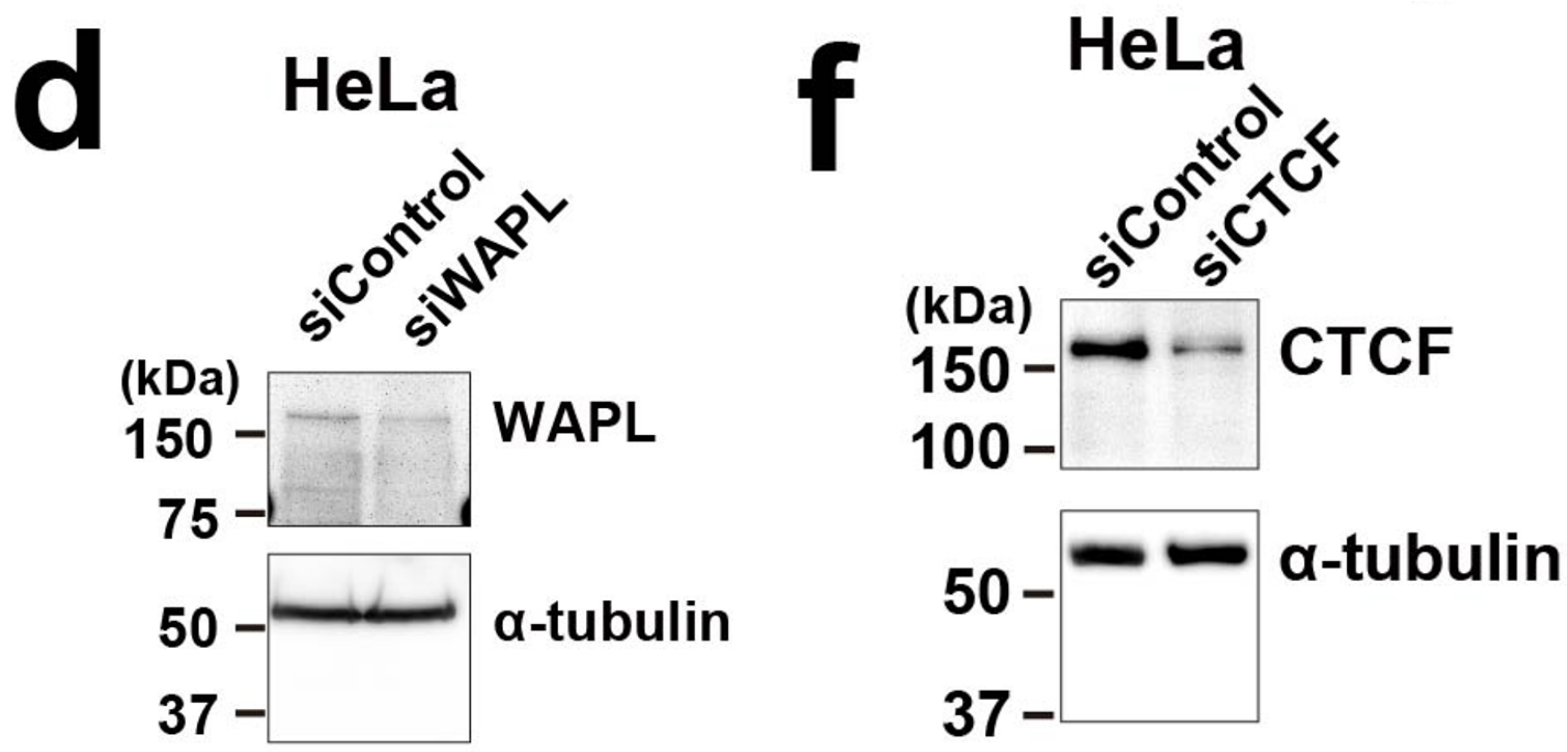

10. The authors claim “implying that cohesin constrains movements of nucleosomes mainly in euchromatin.” is intriguing but may be premature. Since “Super-slow” and “Slow” trajectories are only presumably heterochromatin (as they also state), stronger evidence (e.g., perturbing H1 or laminB) would help.

We agree that the biological identities of these subgroups are not yet fully defined. However, the differential sensitivity of the “fast” and “super-fast” subgroups to cohesin depletion provides an important clue that cohesin primarily affects euchromatin dynamics. This motivated us to proceed with euchromatin-specific analysis using H3.3-Halo. To preserve the manuscript’s flow and avoid overextending this section, we preferred not to delve too deeply into its mechanistic interpretation in this manuscript and moved this data to Supplementary Fig. S4. Further analyses such as perturbations of H1 or laminB, as suggested by Reviewer #1, will be valuable directions for future work to investigate physical nature of heterochromatin. We sincerely appreciate this constructive suggestion.

11. Figure 5 shows strong evidence for euchromatin, but does not exclude similar roles in heterochromatin. Testing H1-Halo could be informative.

We agree with Reviewer #1’s point regarding whether H3.3–Halo truly excludes heterochromatin. Because high-quality public H3K9me3 ChIP–seq datasets in HeLa suitable for our comparison are limited, we instead performed a pull-down of H3.3–Halo nucleosomes in HCT116 cells (BioProject accession number: PRJDB39674), mapped them genome-wide, and compared them with multiple histone marks, including H3K9me3 (Fig. 4b-d, see below). We obtained consistent results, showing depletion in the Hi-C B compartment marked by repressive histone marks such as H3K9me3 and H3K27me3. As described later, these genome-wide maps of H3.3–Halo nucleosomes in HCT116 cells, together with available Hi-C, ChIP-seq, and ATAC-seq datasets (including Rao et al., Cell 2017 (Rao et al., 2017)) (Figs. 6 and 8a), further support our imaging-based conclusions on condensed euchromatin domains and their local mixing upon cohesin depletion.

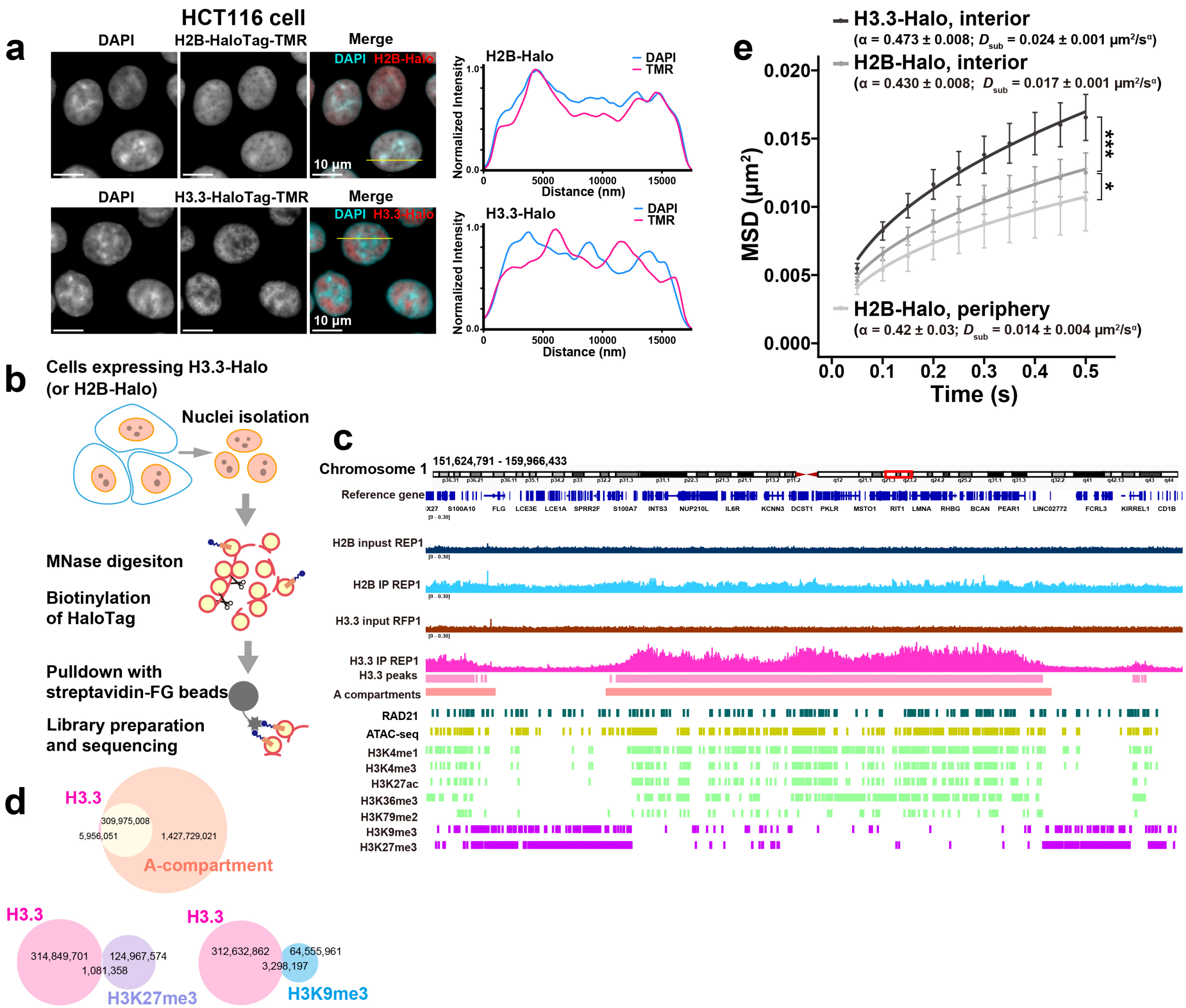

We also examined the behavior of linker histone H1-Halo in our posted preprint: https://www.biorxiv.org/content/10.1101/2025.03.05.641622v2.full
In that study, H1.2-Halo is dynamically associated with chromatin genome-wide.

12. Supplemental figures S5a and S5b would benefit from clearer labeling and inclusion of uncut membranes with ladders.

We have added the uncut western blot results, including molecular weight ladders, for all western blot results (Figs. S1a, S1b, 3a,d, S2, S3, S5a,b,c, S9c,d) to improve clarity (Fig. S11-15).

13. Figure S7b shows small differences at the nuclear periphery after RAD21 depletion. Was alpha calculated for these experiments? It would be interesting to see the extent of the delta between different classes.

We have added the MSD exponents for nucleosome motion at the nuclear interior and periphery (Fig. S7) to address this point.

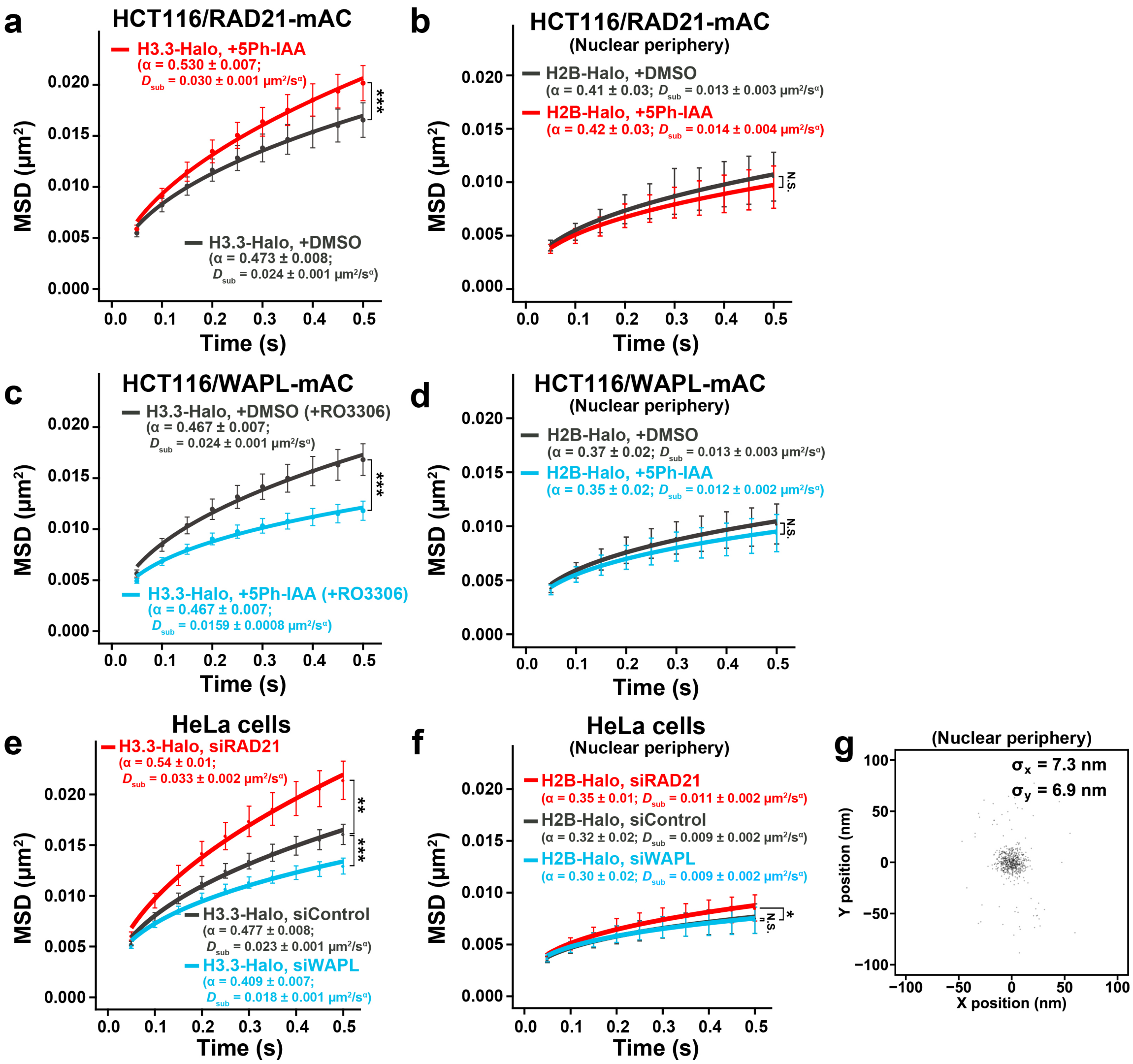

14. The statement “These results demonstrate that cohesin primarily constrains euchromatic regions.” may be too strong, as conclusions apply only to lamina-associated domains. Acknowledging constitutive vs. facultative heterochromatin differences would be helpful.

We appreciate the Reviewer #1’s suggestion and agree that our conclusion should be more carefully stated. In this study, our analysis of the nuclear periphery mainly represents lamina-associated domains (LADs), which correspond to constitutive heterochromatin. We have revised the corresponding Results section to acknowledge this point and to clarify that our results do not exclude the possibility that cohesin may also influence facultative heterochromatin regions under certain conditions:

“These results suggest that cohesin primarily constrains euchromatic regions while our results do not exclude the possibility that cohesin may also influence facultative heterochromatin regions under certain conditions.”

15. H3.3 is enriched but not exclusive to euchromatin. It is also observed in facultative heterochromatin (Banaszynski, L. auraA. et al. Hira-dependent histone H3.3 deposition facilitates PRC2 recruitment at developmental loci in ES cells. Cell 155, 107–120 (2013)., ang, H.-T. et al. Global H3.3 dynamic deposition defines its bimodal role in cell fate transition. Nat. Commun. 9, 1537 (2018).). Please acknowledge this nuance.

We appreciate Reviewer #1’s comment regarding the distribution of H3.3. We agree that although H3.3 is predominantly incorporated into euchromatin, it is not strictly exclusive to it. We now explicitly acknowledge this nuance in the “Limitations of the study” section:

“Our experiments were performed in somatic human cells (HCT116 and HeLa), where H3.3 incorporation predominantly marks euchromatin. H3.3 has also been reported in the telomeric region (Goldberg et al., 2010), or facultative heterochromatin in specific contexts, such as embryonic stem cells and during cell-fate transitions (Banaszynski et al., 2013; Fang et al., 2018). The degree of condensation may also differ across cell types. Exploring these contexts will be an interesting next step.”

16. Figures 6 and related analyses would benefit from clarification: number of cells analyzed, logic behind signal density as a measure of condensation, quantification of “frequently” in cohesin localization, and clearer explanation of DAPI class definitions. Cell cycle synchronization effects should also be discussed.

We appreciate Reviewer #1’s helpful suggestions regarding clarification of the Figure 6 (current Fig. 5) analyses.

We have now added detailed descriptions in the Methods and figure legends as follows:

1. Number of analyzed cells:
   We analyzed $N = 37$ cells for DAPI/H3.3-Halo-TMR classification and $N = 37$ (RAD21), 15 (RNAPII CTD), 19 (RNAPII Ser5P), and 23 (RNAPII Ser2P) for localization analyses. The sample numbers are now indicated in each figure legend.

2. Logic behind signal density as a measure of condensation:
   We clarified that signal density (fluorescence intensity per voxel) reflects local chromatin packing density, which has been widely used as a proxy for chromatin condensation in previous SIM studies (Cremer et al., 2017; Miron et al., 2020).

3. Quantification of “frequently” in cohesin localization:
   It is difficult to precisely quantify the fraction of cohesin foci located within H3.3-Halo domains because of resolution limitations. Instead, we demonstrated genomic data showing that cohesin locates around the H3.3-Halo domain boundaries, rather than domain interiors (Fig. 7).

4. Explanation of DAPI class definitions:
   We have expanded the Methods section to define each DAPI signal class (1–7) according to the intensity thresholds used by (Miron et al., 2020; Schmid et al., 2017).

5. Cell-cycle synchronization effects:
   All SIM (also STORM) analyses were performed using asynchronous populations of HCT116 cells. While cohesin localization patterns and H3.3 distribution did not show noticeable variation among interphase cells, this remains an interesting question for future investigation, as genomic DNA content doubles and nuclear volume increases during cell cycle progression (Iida et al., 2022). We note this point in the "Limitations of the study" section.

17. The two-point MSD analysis is elegant but could be expanded:
  a. "Two-point MSD analysis showed much greater motion between neighboring H3.3-Halonucleosomes in living cells than in FA-fixed cells (Fig. 7i)," but what about unrelated nucleosomes? In double-stained H3.3-Halo experiments, do unrelated nucleosomes behave similarly to neighboring ones?
  b. Could the same analysis be done in the tetO/lacO line?
  c. Could this be repeated with H2B to compare euchromatin vs. general chromatin?
  d. How long after RAD21 depletion were experiments performed? Would longer depletion change results further? It would be interesting to allow chromatin to mix even more and observe if the two-point MSD of neighboring nucleosomes starts to behave like unrelated nucleosomes.

We appreciate Reviewer #1's insightful comments regarding the two-point MSD analysis. Below we address each point.

(a) Neighboring vs. unrelated nucleosomes:
We thank Reviewer #1 for highlighting this important control. We quantified two-point MSD for unrelated H3.3–Halo pairs within the same nucleus (initial separation distance d, 300 nm < d < 1000 nm) alongside neighboring pairs. For the unrelated control pairs, two-point MSD was approximately twice the one-point MSD at matched lags, as theory predicts for uncorrelated motion. By contrast, neighboring pairs showed lower two-point MSD, consistent with positively correlated motion within the same domain. In both cases, RAD21 depletion increased two-point MSD. The results have been added to Fig. S9j and into the Results text.

"As a control, we analyzed two-point MSD for unrelated (long distance) H3.3–Halo pairs (300 nm < d < 1000 nm) (Fig. S9j)."

"Two-point MSD for unrelated (long distance) control pairs was approximately twice the one-point MSD at matched lags (Fig. S9j), as theory predicts for uncorrelated motion. By contrast, neighboring pairs showed lower two-point MSD, consistent with positively correlated motion within domains. In both classes, RAD21 depletion increased two-point MSD. Taken together, neighboring-pair two-point MSD reveals the nature of local physical constraints within euchromatic domains."

(b) Analysis in the tetO/lacO line:
We agree that this system could in principle probe local loop-related motion. However, while we

analyze ~1,000 H3.3–Halo pairs per cell, the tetO/lacO line provides only a single LacI–GFP/TetR–mCherry pair. Since two-point MSD analysis is much more sensitive to sample size than normal MSD analysis, we did not pursue this analysis further.

(c) Comparison with H2B–Halo:
We performed two-point MSD on H2B–Halo and have added the new data (Fig. S9m).

(d) RAD21 depletion duration:
We conducted RAD21 depletion for 1, 3, and 6 h and obtained similar two-point MSD values; these results have been added.

"Notably, two-point MSD increased upon acute cohesin depletion (1 h 5Ph-IAA) (Figs. 7i, S9k-m). Longer depletions (3 h or 6 h 5Ph-IAA) did not further change the two-point MSD (Fig. S9k)."

We also extended the two-point analysis for H3.3–Halo to 2 s, which supports increased domain mixing in the absence of cohesin (Fig. S9l).

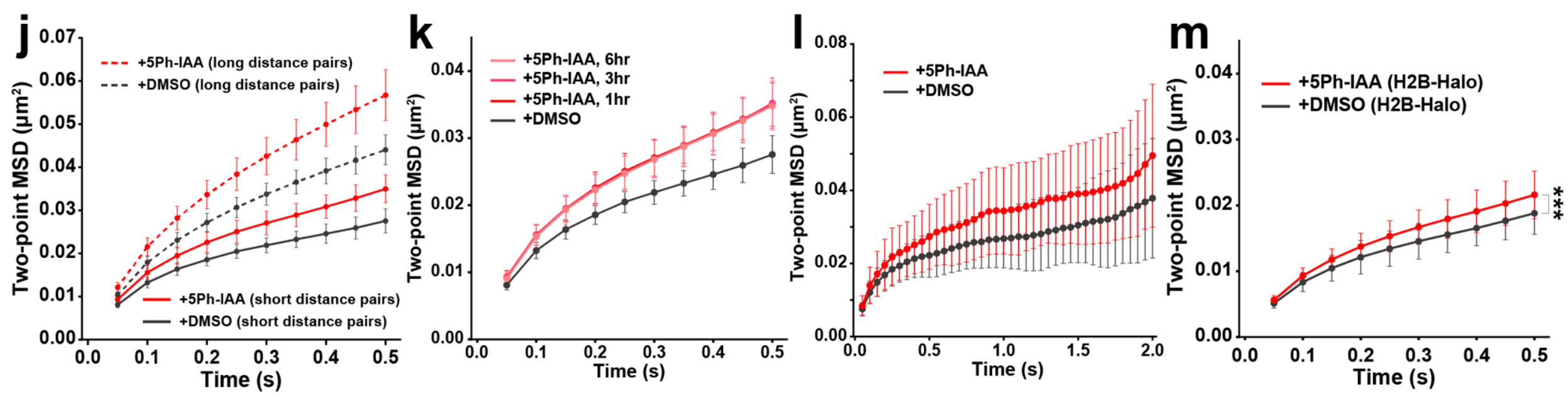

18. Please clarify how "same-domain" nucleosomes were identified, beyond proximity alone.

We thank Reviewer #1 for this helpful comment. In our study, "same-domain" nucleosomes were identified based on spatial proximity within a 150-nm distance (identified in our previous study (Nozaki et al., 2023)). This threshold is consistent with the peak diameters (200 to 240 nm) of H3.3-Halo domains (Fig. 7d). We realized that nucleosomes that are spatially adjacent or locally clustered—whether strictly within a single domain or between nearby domains—could both contribute to the phenomenon of local mixing described in this work. We have clarified this conceptual point in the text and appreciate Reviewer #1's suggestion, which helped us refine our description.

"Since simple single-nucleosome imaging cannot distinguish whether individual nucleosomes fluctuate within the domain or whether the domain itself moves, we measured the distances between two neighboring H3.3-Halo-nucleosomes within euchromatic domains or possibly between adjacent domains (average <150 nm; Fig. 7e) (Nozaki et al., 2023) and obtained the MSD of their relative positions (two-point MSD) (Gabriele et al., 2022; Minami et al., 2025; Nozaki et al., 2023)."

19. The conclusion "suggesting a transition to a more liquid-like state of chromatin." is compelling, but it may also reflect loss of tethering. It would be helpful to contemplate

alternative explanations, such as an “unrestricted state of the chromatin polymer”

We agree with Reviewer #1’s point and have added the phrase “unrestricted state of the chromatin polymer” in the Results:

“These results… induces a transition to a more liquid-like state of chromatin, or to an unrestricted state of the chromatin polymer.”

20. Finally, please be cautious in generalizing. Since experiments were performed in HCT116 and HeLa cells, the statement that “euchromatin is condensed in living human cells” should be phrased more carefully, noting possible cell-type–specific differences.

We appreciate Reviewer #1’s suggestion and added the following sentence in “Limitations of the study” section in Discussion:

“Our experiments were performed in somatic human cells (HCT116 and HeLa), where H3.3 incorporation predominantly marks euchromatin. H3.3 has also been reported in the telomeric region (Goldberg et al., 2010), or in facultative heterochromatin in specific contexts, such as embryonic stem cells and during cell-fate transitions (Banaszynski et al., 2013; Fang et al., 2018). The degree of condensation may also differ across cell types. Exploring these contexts will be an interesting next step.”

Reviewer #1 (Remarks on code availability):

The code is well annotated with the README with instructions for the "Trackmate_multiplefile".
However, the twopoint_MSD.zip does not have a README with instructions. Before publications I strongly suggest to include this as well.

We have included the information.

Reviewer #2 (Remarks to the Author):

In the manuscript ‘Cohesin prevents local mixing of condensed euchromatic domains in living human cells’ the authors broadcast a high level of imaging, which gives a nice peek in the nucleus. The authors show how the movement of nucleosomes is dependent on the binding of cohesin to chromatin. In particular, the loop extruding cohesin is crucial for the movement. The second part of the paper goes more in depth on the different populations of nucleosomes, differences between H2B and H3.3 bound nucleosomes and how they are affected by cohesin binding.

The paper contains state-of-the-art imaging techniques, however for me it is difficult to interpret the differences in measurements as currently presented. I would like to see more intuitive figures to understand the data before I would say the manuscript is ready

for publication in Nature Genetics. The finding that cohesin is involved in the nucleosome movement might be expected from their localisation, however to see this experimentally with very elegant microscopy is interesting for the field. Figure 6 does not add too much as it has been merely already shown by Miron et al., I think the paper would benefit it there is more focus and to explain figure 7 to the bigger scientific field. By focusing more on this one point the manuscript would be more appealing for a broader audience.

If my points are taken into account I would consider publishing this manuscript in Nature Genetics.

We are very grateful for Reviewer #2's efforts and supportive comments, which were helpful in improving our paper. The point of Fig. 6, which builds on Miron et al., is that euchromatin labeling reveals condensed domains in live cells. We have also added new data showing the decondensation of these domains after HDAC inhibitor TSA treatment, which induces histone-tail hyperacetylation and weakens local nucleosome–nucleosome interactions. Combined TSA treatment and cohesin depletion markedly decondensed H3.3–Halo–labeled euchromatin domains (Fig. S9). This is analogous to recent findings that mitotic chromosomes are "condensed" by local nucleosome–nucleosome interactions and the condensin complex (Hibino et al., 2024; Schneider et al., 2022). These data provide further evidence that euchromatin domains are truly "condensed" via nucleosome–nucleosome interactions and cohesin, and exclude the possibility that the observed condensation is an imaging artifact. Following the suggestion, we have placed greater emphasis on Fig. 7 and the new Fig. 8:

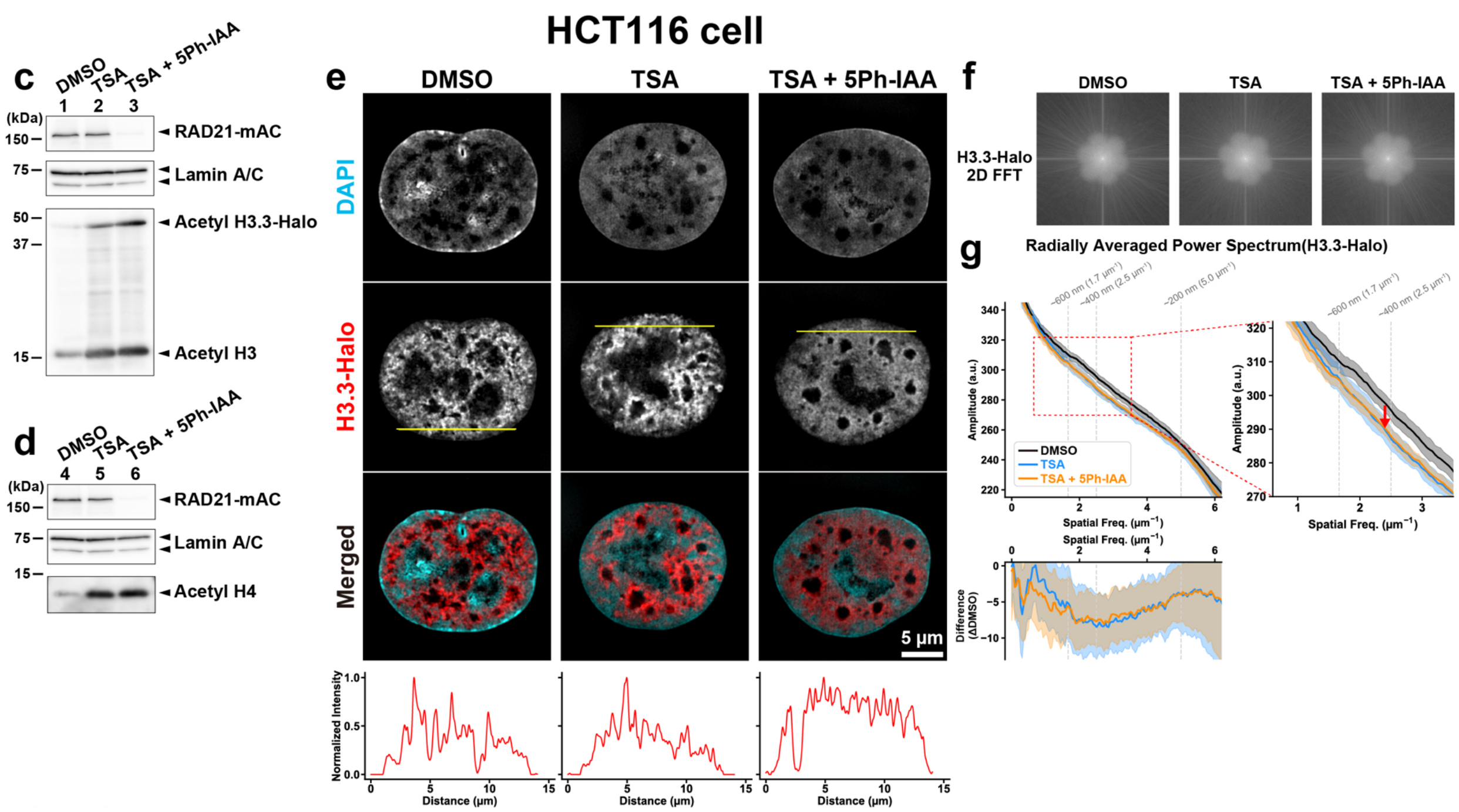

We have also added the following to the Results section:

"**Nucleosome–nucleosome interactions and cohesin are involved in condensed domain formation**

To investigate which factors condense euchromatic domains, we treated the cells with the HDAC inhibitor trichostatin A (TSA) (Yoshida et al., 1990), which induces hyperacetylation of histone tails (Fig. S9c,d) (Toth et al., 2004). Tail hyperacetylation weakens local nucleosome–nucleosome interactions (Kalashnikova et al., 2013) and decondenses chromatin (Nozaki et al., 2017; Otterstrom et al., 2019; Ricci et al., 2015). Indeed, mitotic chromosomes were highly decondensed and expanded in volume in condensin-depleted and TSA-treated cells (Hibino et al., 2024; Schneider et al., 2022). Following treatment with TSA for 7 h and acute depletion of RAD21 (Fig. S9c,d), apparent decondensation of H3.3-Halo domains was observed by 3D-SIM (Fig. S9e). Power-spectrum analysis showed that the amplitude at spatial scales of ~300–600 nm (domain scale) decreased substantially (Fig. S9f,g). Together, these results indicate that electrostatic interactions between positively charged histone tails and DNA/histones, together with cohesin, contribute to condensed domain formation. Moreover, these results show that our 3D-SIM was able to detect chromatin decondensation and confirm that the condensed euchromatic domains were not an imaging artifact. Our observations provide compelling evidence that challenges the established view of euchromatin as largely open (Fig. S8g,h)."

Major points:

In figure 1e the authors show the MSD plots of H2B nucleosomes. There is a differences between the cohesin depleted and not depleted. A similar measurement is shown in figure 1i, however the two graphs are identitical to me, except for the number that is written below this. The authors than claim there is a significant differences between the two. How would a difference plot between the two look like? With these plots it is impossible for the reader to assess the data and you need to believe the number below.

We would like to thank Reviewer #2 for raising this issue. We agree that the two plots look similar and may be confusing. By contrast, the asymmetry coefficient (AC), derived from trajectory angles, provides a quantitative and informative measure of how much nucleosomes are constrained. We therefore removed the redundant plots and now present AC values alongside the MSD plots, except for one representative plot. Following Reviewer #3's suggestion, we also introduce the *Rc* (radius of constraint) metric (Dion and Gasser, 2013), which reports the spatial extent of nucleosome motion. *Rc* is more intuitive and statistically easier to compare across conditions.

RAD21-mAC	*Rc*	AC
+5Ph-IAA	176 ± 18 nm	-1.095 ± 0.021
+DMSO	146 ± 22 nm	-1.218 ± 0.025

In figure 3b the authors show a depletion of WAPL, it has been previously reported that this will lead to 'Vermicelli' in the cohesin staining (Tedeschi et al.). Do the authors observe this? It is not visible in the example pictures.

Yes, we indeed observed the "vermicelli" pattern in cohesin staining upon acute WAPL depletion, consistent with (Tedeschi et al., 2013). Because it is not readily visible at the scale

shown in Fig. 3b, we have added an enlarged image highlighting the vermicelli-like structure and noted this in the corresponding figure legend.

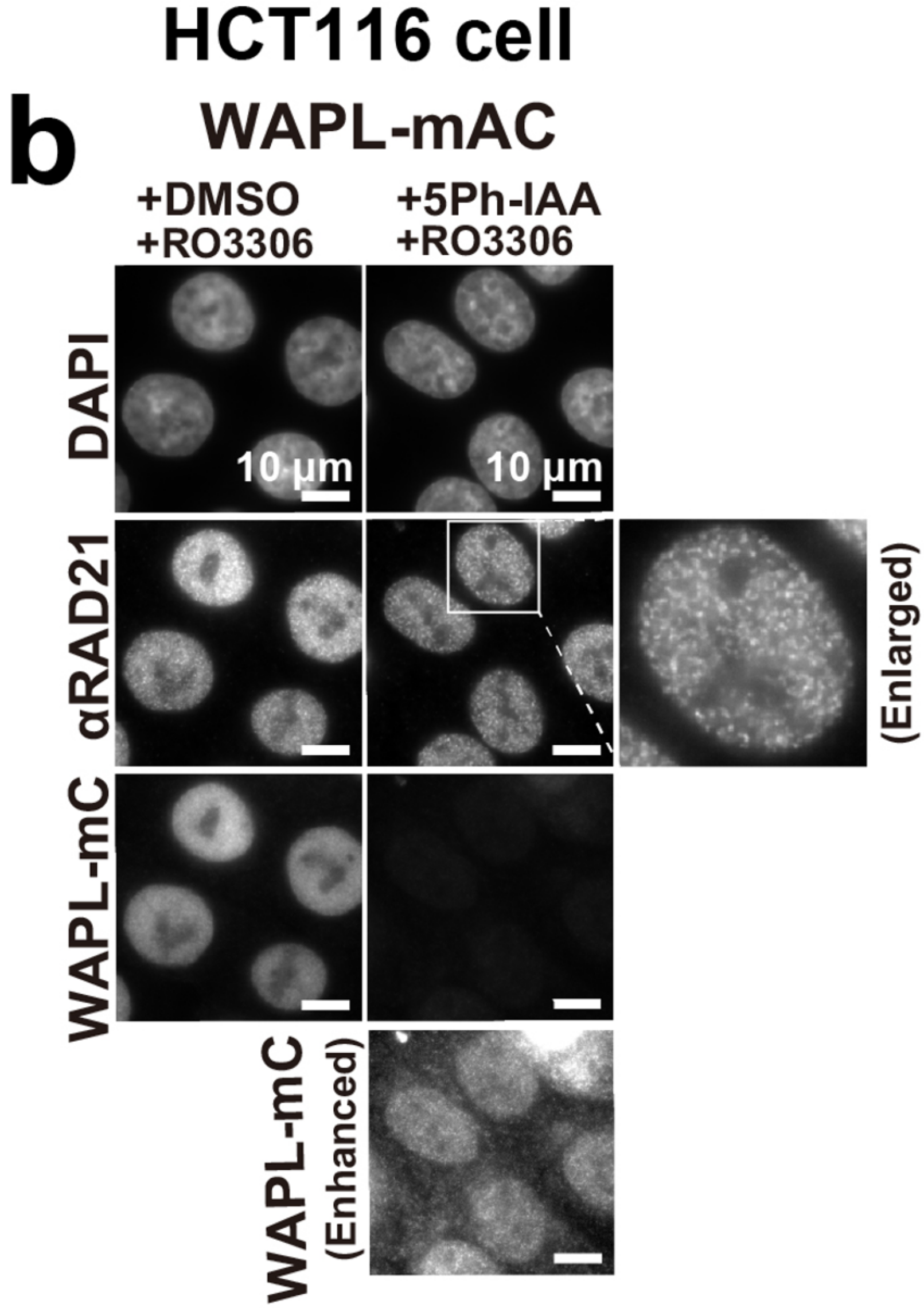

"The enlarged image in panel (b) highlights the vermicelli-like structure in RAD21 staining, consistent with (Tedeschi et al., 2013)."

Figure 3d,h and k are again identical.

We agree that the plots in Fig. 3d, 3h, and 3k appear similar and may be confusing. As noted above, we have addressed this point.

In figure 3i,j,k the authors try to assess whether cohesin translocation or active loop extrusion by cohesin is required for the chromatin constraint. They use a head-tethered cohesin system, this did not change the nucleosome motion. This is quite a difficult experiment to interpret and to make such strong conclusions.

We fully agree that the result cannot make these strong conclusions. We have described the result more carefully and toned down the conclusion.

"Finally, to examine the possible involvement of active loop extrusion (Davidson and Peters, 2021; Uhlmann, 2025) in the constraining process, we used a head-tethered cohesin system (Sakata et al., 2021) that stabilizes cohesin via forced head domain closure using AP21967 (Fig. 3g,h) (Bayle et al., 2006; Grunberg et al., 2010). Head-tethering weakened loop-extrusion activity *in vitro* (Sakata et al., 2021). Head-tethering alone did not change nucleosome motion on the 0.5 s timescale, but head-tethering after RAD21 depletion led to the loss of the entire cohesin complex (Sakata et al., 2021) and increased motion (Figs. 3i, S3h,i). These results suggest that chromatin constraint by cohesin may not require active loop extrusion."

First I think it is required to make a timeline to explain the reader how the experiment is executed.

We agree that a timeline would help clarify the workflow. We have added a schematic timeline detailing the sequence and timing of perturbations and measurements (Fig. 3g).

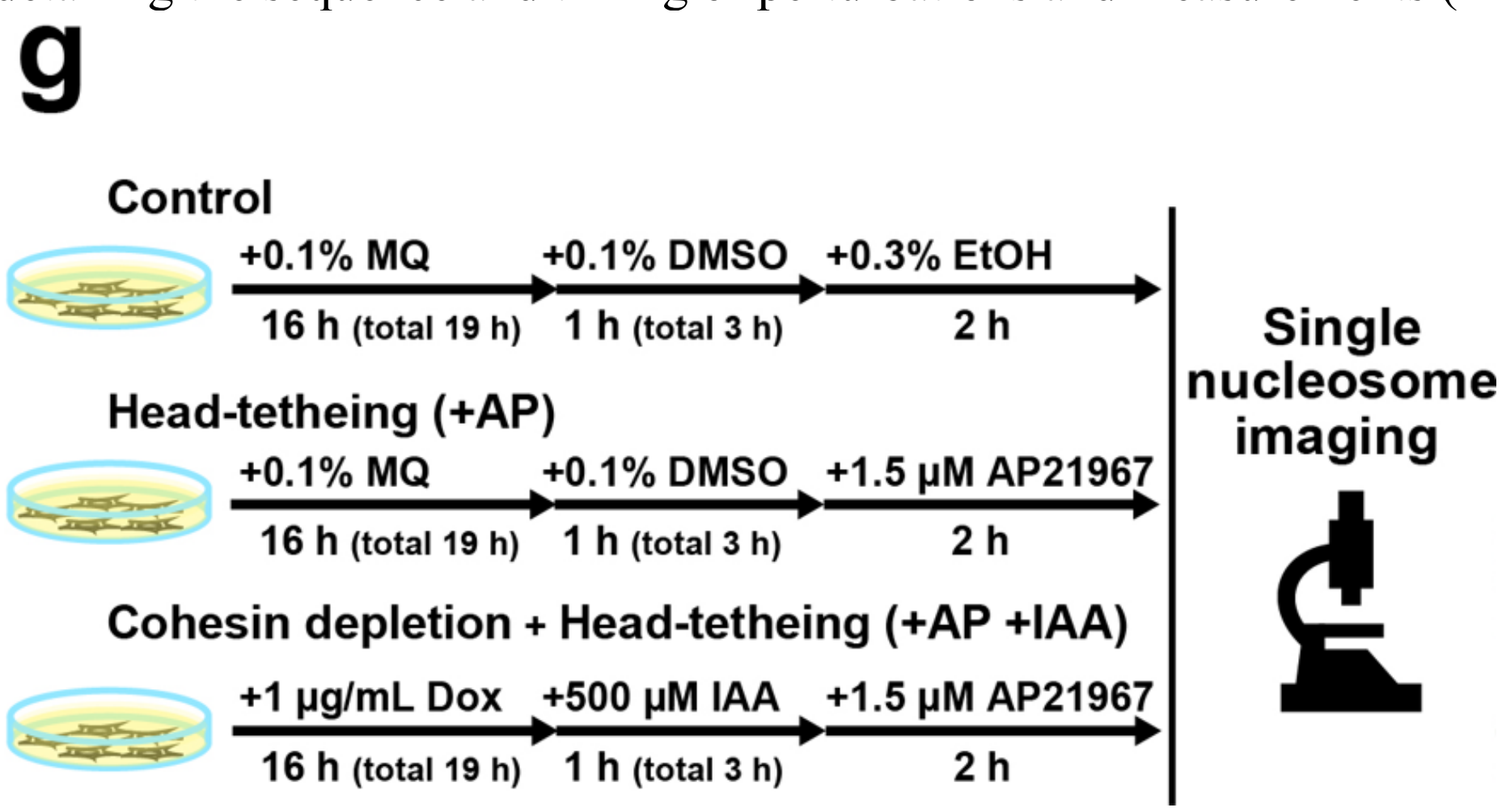

Secondly, with the fusion the cohesin is stuck on the DNA, similar to WAPL-depletion, in which it can also be discussed how much active loop extrusion is still ongoing when the cohesin rings are trapped at CTCF sites. Why does the fusion not give a similar result to WAPL depletion?

We thank Reviewer #2 for raising this point. The difference between head-tethering cohesin and WAPL depletion is as follows: WAPL depletion suppresses cohesin dissociation from chromatin, allowing cohesin to remain bound and continue to accumulate on chromatin. By contrast, head-tethered cohesin is immobilized and does not promote new chromatin loading. Therefore, WAPL depletion leads to much greater cohesin occupancy and stronger chromatin constraint than head-tethering. We have described this in the main text:

"Also, the difference between head tethering and WAPL depletion explains the distinct outcomes. WAPL depletion suppresses cohesin dissociation, allowing cohesin to remain bound and continue to accumulate on chromatin. By contrast, head-tethered cohesin is immobilized at existing sites and does not promote new chromatin loading. Consequently, WAPL depletion

yields much higher cohesin occupancy and stronger chromatin constraint than head tethering (Fig. 3c,i)."

The reason for the addition of RAD21 depletion on top is not clear to me. Do the authors think SMC1 and SMC3 are than still fused to the chromatin or is the complex not intact at all and nothing is present on the chromatin? I would assume the second, it is just the same as in figure 1.

We appreciate the comment. The latter is correct: depletion of RAD21 in the head-tethered condition leads to loss of the entire cohesin complex (Sakata et al., 2021), and the effect is the same as in Fig. 1. We have clarified this in the Results:

"Head-tethering alone did not change nucleosome motion on the 0.5 s timescale, but head-tethering after RAD21 depletion led to the loss of the entire cohesin complex (Sakata et al., 2021) and increased motion (Figs. 3i, S3h,i)."

But I really don't understand how the authors can claim here it is not active loop extrusion but translocation? The cohesin complex is too complex to simply say we fuse it here and see what nucleosome do.
Looking at the effect of loop extrusion inside cells is until now very difficult, an option would be to deplete NIPBL once the cells are in G2, however it will not only effect loop extrusion but also cohesin loading which will affect the nucleosome movement.

We thank Reviewer #2 for this thoughtful comment. We agree that distinguishing active loop extrusion from other cohesin dynamics in vivo is difficult, and our data do not support a decisive conclusion. We have therefore weakened the conclusion described above and revised the text to read more cautiously.

WAPLKD increase bound cohesin and constrain more. Misunderstanding

We apologize for the misunderstanding. To clarify, WAPL depletion increases chromatin-bound cohesin and further constrains chromatin, whereas the head-tethered condition immobilizes cohesin on chromatin without increasing genome-wide occupancy or residence time, and therefore does not phenocopy WAPL depletion:

"Head-tethering alone did not change nucleosome motion on the 0.5 s timescale, but head-tethering after RAD21 depletion led to the loss of the entire cohesin complex (Sakata et al., 2021) and increased motion (Figs. 3i, S3h,i)."

In figure 4 d the authors show 4 different subpopulations of nucleosome movement. Upon depletion of cohesin not only the fast population becomes faster also there is a clear difference in this plot in the height of the super-slow fraction which here only reaches to 2.5 while with cohesin it almost reaches 3. Also the area under the curve in the fast population seems bigger than in the DMSO control. However the authors show in figure 4h no differences in population, could the authors explain this?

We thank Reviewer #2 for this careful observation. Fig. 4d shows normalized probability densities of MSD at $\tau = 0.5$ s. Because each curve is normalized to unit area, peak height reflects both the location and the spread of the underlying modes and is not a direct measure of subpopulation fractions. Upon cohesin depletion, the fast mode shifts to larger MSD values and slightly broadens, and the super-slow mode also broadens, which lowers its peak height even when its weight is unchanged.

In old Fig. 4h (current Fig. S4l), we reported subpopulation fractions estimated per cell and compared the inferred weights across biological replicates. The plots show that the component weights do not differ significantly between conditions, whereas the fast and super-fast component peak positions shift. We have clarified this in the figure legend:

"Fractions of each subpopulation. Cohesin depletion did not alter the relative proportions of the subpopulations, whereas the fast and super-fast components shifted."

Ratio of euchromatin to heterochromatin is not changed?

Yes, we think so. However, please note that from this RL analysis we could not conclude anything about euchromatin or heterochromatin because we observed only subfractions of nucleosome motion. We therefore moved the RL analysis data to Supplementary Fig. S4 and focused on histone H3.3-HaloTag-labeled nucleosomes to investigate euchromatin (Hi-C A compartment) more specifically.

The authors show that histone 3.3 is more enriched in euchromatin and underpin this with the histone marks from published ChIPs. However the histone 3 k27me3, mark shows the opposite and they don't show ChIP results for H3 k9me3. Is it known that H3.3 does not have these repressive marks, which indicate heterochromatin domains?

We appreciate Reviewer #2 for raising this critical issue. H3K9me3 was not shown in the previous manuscript because high-quality H3K9me3 ChIP data in HeLa cells were not publicly available. To clarify the relationship between H3.3–Halo domains and heterochromatin regions, we performed a pull-down of H3.3–Halo nucleosomes in HCT116 cells, mapped them genome-wide, and compared them with multiple histone marks, including H3K9me3. These analyses show that H3K9me3 is depleted over H3.3–Halo domains, supporting that our H3.3 signal predominantly reflects euchromatin.

In figure 5, I'm missing a ChIP for cohesin here. Is cohesin enriched at the (borders of these) domains of which they say to be affected by depletion of cohesin? A thorough analysis of this would be informative.

We appreciate this helpful suggestion. We have now added cohesin (RAD21) ChIP-seq overlays in HCT116 to the new Fig. 4. Using the H3.3–Halo domain maps, we show that cohesin is enriched at domain boundaries. We also included distributions for CTCF, RNA Pol II, ATAC-seq, and multiple histone marks aligned to domain borders (±100 kb). These analyses are presented in the new Figs. 6, S5, and S8, and the Methods have been updated accordingly. The added

genomic data on H3.3–Halo localization in HCT116 further strengthen our conclusions.

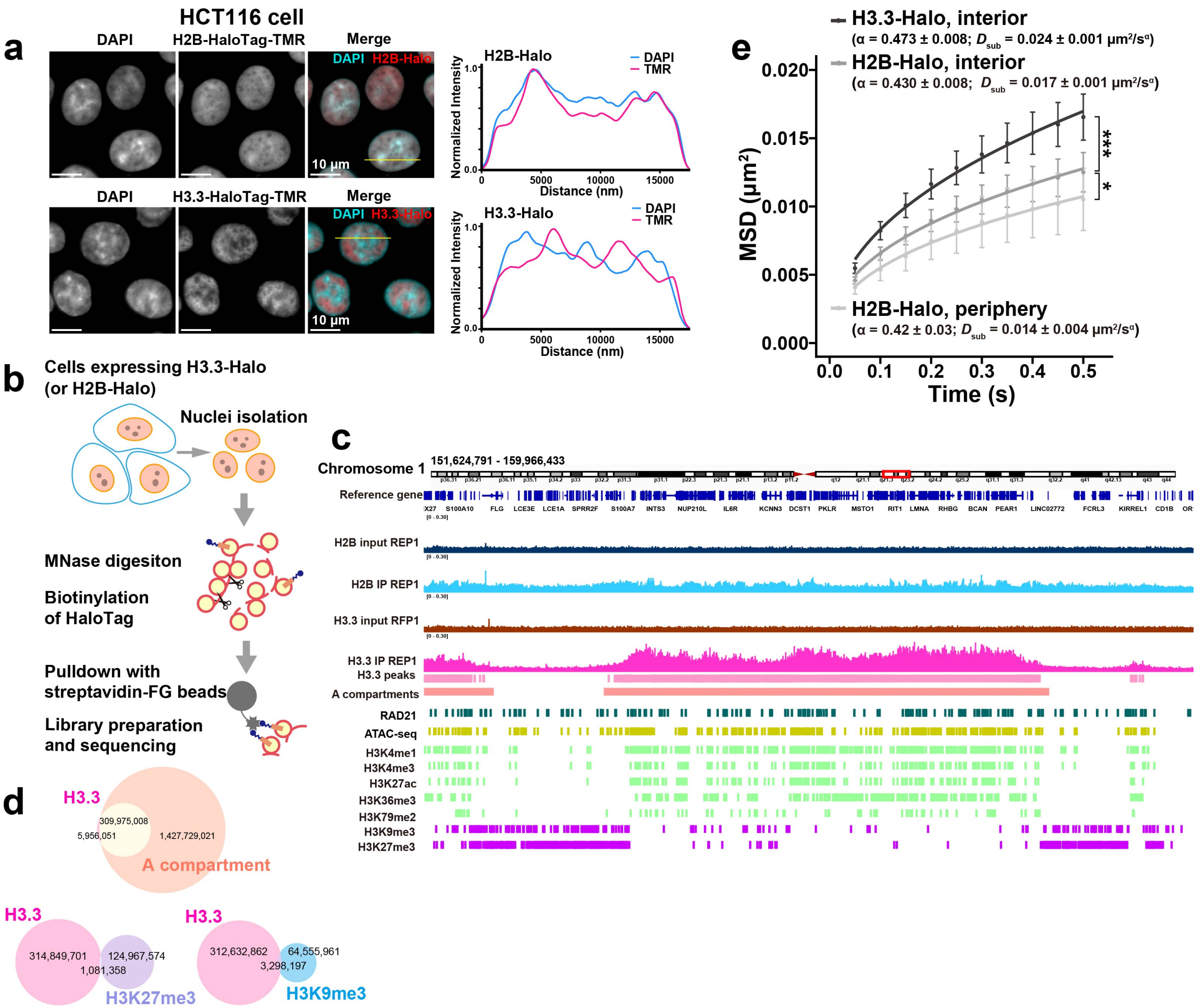

New Fig. 4

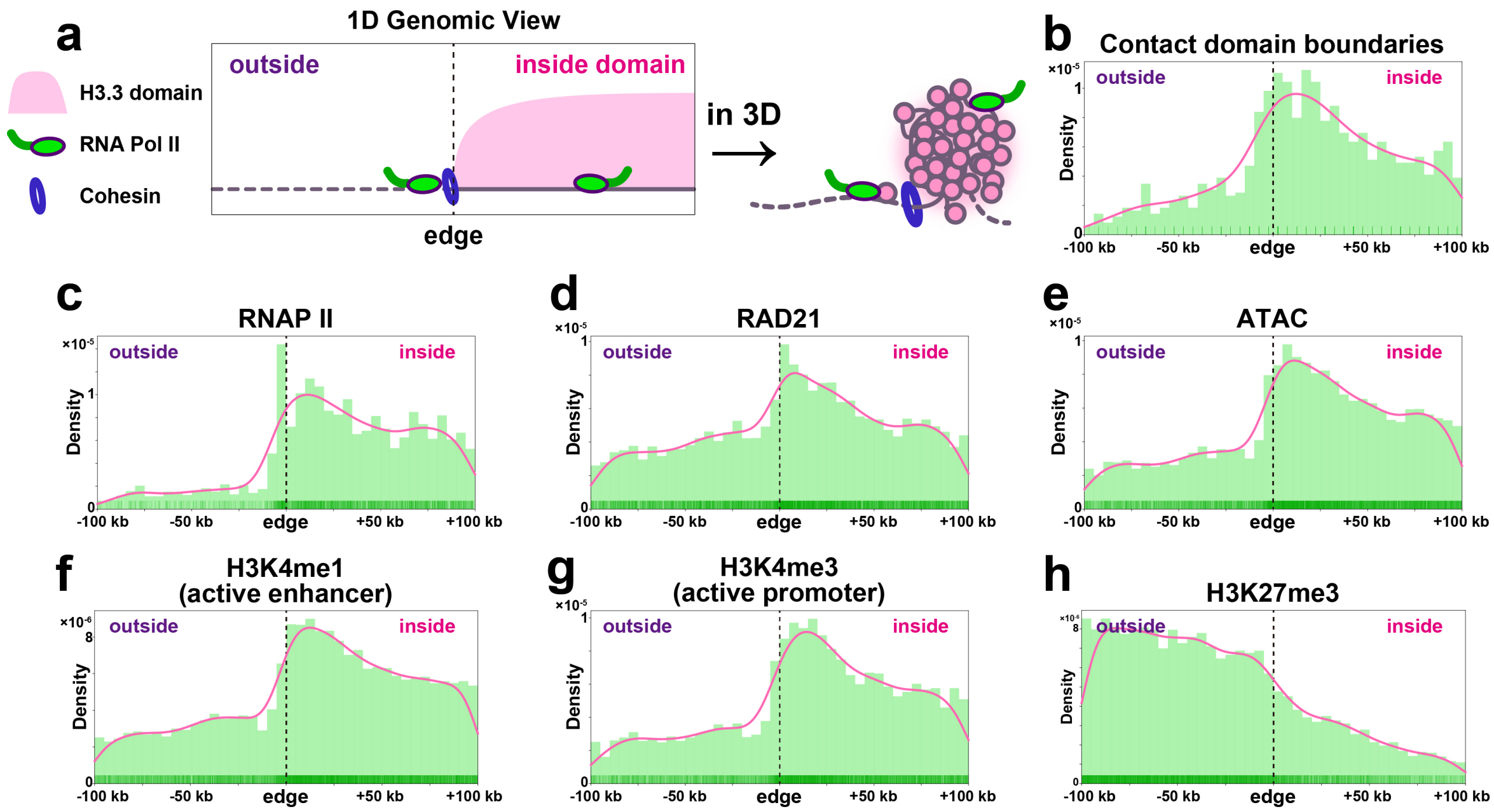

New Fig. 6

Reviewer #3 (Remarks to the Author):

In this manuscript titled “Cohesin prevents local mixing of condensed euchromatic domains in living human cells”, Iida et al investigate how cohesion influences the mobility of nucleosome scale chromatin dynamics and the organization of active chromatin in live human cells. The authors use single molecule tracking of H2B, H3.3 and SIM imaging of H3.3, RAD21, RNAPII and DAPI along with depletion or knockdown of RAD21, Sororoin, WAPL, and CTCF. Their primary conclusion is that euchromatin is organized into condensed domains constrained by cohesin, loss of which leads to “fluidization” and local mixing of euchromatin domains without compaction.

The study is highly relevant and important for understanding how local genome organization has functional implications in gene regulation. The conclusions are largely in line with previous reports on the effects of cohesin loss on chromatin mobility but the main potential conceptual advance of “mixing” is underdeveloped. This is because there is a lack of mechanistic connection between “mixing”, loop extrusion and transcriptional activity. Several key conclusions also lack appropriate validation because of technical concerns, data analysis, lack of appropriate controls (most importantly with regards to effects on transcription).

We really appreciate Reviewer #3’s critical but thoughtful comments. They were invaluable in improving our paper. We agree that the concept of “local mixing” needs to be defined more clearly and that its connection to cohesin activity and transcription should be strengthened. We have therefore clarified the definition, softened our claims, and added extensive genomics analyses and controls to address these points.

Major Comments:
The presentation of data is confusing, in most cases just MSDs are presented. Although it seems that the MSDs were fit inferred alpha values and diffusion coefficients are not presented appropriately with error analyses/statistics. It is unclear if the exponent increasing from 0.43 to 0.48 is significant even more so with a lack of what distributions of values are obtained across multiple replicates. The authors should report fold-changes in alpha values and diffusion coefficients with confidence intervals, and emphasize biological relevance over statistical over-powering.

The authors should consider alternative analyses to MSD which is not appropriate to analyze SMT data as it has been shown in several publications (e.g. Hansen et al., elife, 2028) that it is error prone especially when there are mixtures of diffusion states. The authors should consider analyzing their data using fitting of displacement distributions or other alternatives to determine if there is indeed a change in diffusion coefficients. The authors can also calculate metrics like radius of gyration.

We thank Reviewer #3 for these important points. We agree that for single-molecule tracking (SMT) of transcription factors (TFs), MSD can be error-prone when multiple diffusion states (free 3D diffusion, 1D sliding, specific binding) interconvert, and that displacement-based analyses are often preferable (e.g., (Hansen et al., 2018)). However, our system differs in a key respect: state mixing is minimal. In single-nucleosome imaging, the vast majority of the labeled histones are incorporated into nucleosomes constrained along a very long polymer rather than freely diffusing, so state mixing is substantially reduced and MSD captures the relevant nucleosome dynamics. We clarify this point in the revised Methods:

"For single-nucleosome imaging/tracking, we calculated mean squared displacement (MSD) of nucleosomes, because MSD captures the relevant constrained nucleosome dynamics, for the following reasons: the vast majority of the labeled histones are incorporated into nucleosomes constrained along a very long polymer rather than freely diffusing, and state mixing is minimal. In this respect, single-nucleosome imaging is very different from single-molecule tracking (SMT) of transcription factors (TFs). MSD in SMT of TFs can be error-prone because multiple diffusion states (free 3D diffusion, 1D sliding, specific binding) interconvert, and displacement-based analyses are often preferable (e.g., (Hansen et al., 2018))."

We also agree that quantifying the free H2B-Halo or H3.3-Halo fraction is critical. In our cells, prior work showed that about 90% of H2B-Halo resides in nucleosomes, with only a minor free pool (Hibino et al., 2024). In addition, we acquired data at a 10 ms frame rate and applied Spot-On analysis (Hansen et al., 2018) to estimate the freely diffusing fraction for H2B-Halo and H3.3-Halo. The free fraction was ≤5% under our conditions, indicating that at the 50 ms imaging used for MSD-based analyses the free component is negligible. These details are now described in the text and new Figs. S1f and S5s.

	Slow bound	Fast bound	Free
H2B-Halo +DMSO	0.001 μm²/s	0.034 μm²/s	0.862 μm²/s
	43.7%	53.0%	3.3%
H2B-Halo +5-Ph IAA	0.002 μm²/s	0.034 μm²/s	1.052 μm²/s
	47.7%	49.7%	2.7%

Fig. S1f

Metrics related to spatial extent were reported previously for H2B-Halo–labeled nucleosomes using the radius of constraint (*Rc*) (Iida et al., 2022). In the revision, we now report *Rc* as well (Figs. 1h and S1g).

RAD21-mAC	*Rc*	AC
+5Ph-IAA	176 ± 18 nm	-1.095 ± 0.021
+DMSO	146 ± 22 nm	-1.218 ± 0.025

Fig. 1h

Following Reviewer #3's suggestions, we now present α values and diffusion coefficients with appropriate statistics.

We also appreciate the request to report diffusion coefficients. We recognize that diffusion coefficients are a common comparison metric in TF SPT. Because nucleosome motion is subdiffusive ($\alpha < 1$), a classical Brownian *D* is not well defined; therefore we emphasize α and *Rc*. To facilitate cross-field comparison, we also present an apparent diffusion coefficient (D_{sub}), estimated from the intercept of log-log MSD plots. While provided for reference, this measure has limited interpretive value for subdiffusive, confined motion.

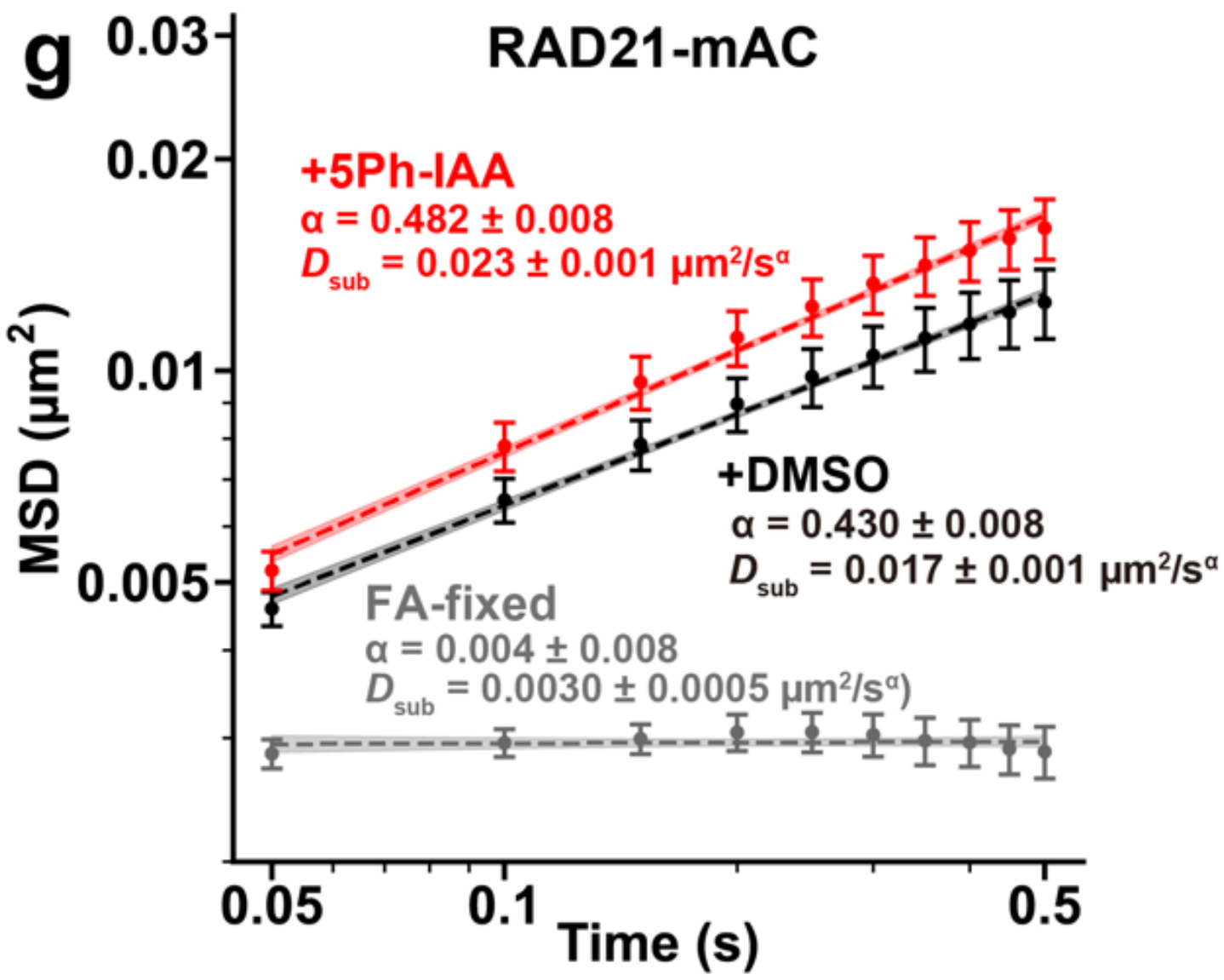

Fig. 1g

We are grateful to Reviewer #3 for highlighting these points, which helped improve the manuscript.

-Regarding figure 4, the authors should consider overlaid analysis with heterochromatin markers such as HP1 to be confident that changes are restricted to euchromatic regions. As in the above comment, why aren't diffusion coefficients calculated and presented instead of "Peak MSD values" this is confusing, the small shifts here are hard to interpret.

The H3.3 single molecule experiments should also be presented as MSDs along with fitted values and compared to H2B statistically, it is hard to compare these results to the rest of the paper. Where does the H3.3 data lie compared to different mobility classes referred to in figure 4?

We thank Reviewer #3 for the thoughtful comments. Figure 4 was intended as a global, statistical summary of single-nucleosome motion. The four-component mixture reveals subpopulations, and upon cohesin depletion the fast and super-fast components shift to larger displacements. This pattern suggests that cohesin constrains nucleosome motion within euchromatin, but we treat this as suggestive rather than definitive. Therefore, in the revised paper, we put these findings in a supplemental figure (Fig. S4) and put more emphasis on "local domain mixing" in the latter part of the paper as Reviewer #3 suggests.

To pursue the euchromatin-specific hypothesis without overextending RL analysis in the previous Fig. 4, we therefore turned to H3.3-Halo–labeled nucleosomes, which more specifically mark the Hi-C A compartment, and analyzed their MSDs and fitted parameters in subsequent figures. We believe this preserves the manuscript's flow and avoids confusing the reader, while acknowledging Reviewer #3's interest in deeper subpopulation annotation.

Anisotropy analysis on chromatin bound molecules is not typically performed because of the issues with calculating angles at small displacements. The authors should report errors on calculating angles, and what is the error across samples in calculating the AC value. It would also be important to determine the AC value at multiple time and space scales. It is unclear if a change in the AC of -1.218 to -1.095 is meaningful and the authors should comment on what it means biologically. The anisotropy distributions as presented are hard to read, the authors should consider representing them as a standard line graph/histograms that are overlaid so changes can be more easily assessed.

We thank Reviewer #3 for the comments on anisotropy. Consistent with Reviewer #2's concern that the plots were hard to interpret beyond indicating increased constraint, we have removed the angle distribution plots and now report only the AC values alongside the MSD. We do not extend this analysis further. Instead, as described above, we presented *Rc* (radius of constraint) values (Dion and Gasser, 2013) with appropriate statistics in Fig. 1h.

It is clear from the single molecule movies that 50ms exposure time does not fully exclude non chromatin bound H2B. The authors have a few options to explore potential error from this, one is to subsample their movies averaging frames to create synthetic long exposure time data and perform analysis on that. Second the authors could repeat a few measurements at longer exposure times. Third the authors could repeat data at even faster frame rates and analyze the full population so that they can assess the contributions of chromatin incorporated and potentially non-incorporated histones. The third option may be the best as it is important to determine whether perturbations to Cohesin, CTCF, WAPL etc. influence nucleosome incorporation, this should also be addressed biochemically.

We thank Reviewer #3 for this point. To directly quantify any non–chromatin-bound pool, we acquired fast SPT at 10 ms and applied Spot-On (Hansen et al., 2018). The freely diffusing fraction for H2B-Halo and H3.3-Halo is ≤5% in our cells, consistent with prior work showing ~90% nucleosomal incorporation of H2B-Halo (Hibino et al., 2024). This free fraction does not change upon RAD21 depletion (cohesin depletion). Given this minimal and invariant free pool, nuclear masking, step-length/track-length filters, its contribution to the 50-ms MSD analyses is negligible. We state these points explicitly in the Methods. We agree that assessing how perturbations to CTCF, WAPL, etc., influence nucleosome incorporation is an intriguing question, but it is beyond the main scope of this paper.

As mentioned in the above comment the authors should first validate that the chromatin-incorporated fraction of H2B/H3.3 is not affected by RAD21 degradation to prove that enhanced mobility is due to change in chromatin motion rather than due to increased contribution from non-chromatinized H2B/H3.3. Based on the raw movie provided at 50ms, Halo dye molecules outside the nucleus are also clearly visible as distinct spots raising concerns that the authors are actually detecting only the low mobility chromatinized H2B at 50 ms exposure. In addition to using faster imaging as suggested

above the can also use a NLS-HaloTag control to show that free molecules are not tracked at 50 ms.

We directly quantified the non–chromatin-bound pool using 10 ms SPT with Spot-On and found the free fraction for H2B-Halo and H3.3-Halo to be ≤5% and unchanged by RAD21 depletion. At 50 ms, nuclear masking and step-length/track-length filters exclude freely diffusing molecules (described in Methods); hence contributions to the MSD are negligible. Given these controls and the agreement with prior FRAP-based incorporation (≈90%) (Hibino et al., 2024), we did not pursue synthetic long-exposure averaging.

Common quality control metrics should be presented for all SMT data, not just one experiment, including localization precisions, detections per frame, track length distributions.

We respectfully disagree with this point. All primary SMT datasets were acquired and analyzed on the same system. For datasets obtained on a different microscope or processed with a different pipeline, we provided validation data demonstrating comparability, including localization precision within the same acceptance windows. We believe these validations are practically sufficient to support our conclusions.

We also note that the data and analyses presented here build on our single-nucleosome imaging/tracking system, developed and validated across multiple publications since 2012 (e.g., (Hibino et al., 2024; Hihara et al., 2012; Iida et al., 2022; Minami et al., 2025; Nagashima et al., 2019; Nozaki et al., 2017).

The movies are quite dense, from even the presented videos that show tracking several errors are visible. Given the low mobility of histones this may not be a huge concern but some error analysis should be presented.

We thank Reviewer #3 for this point. Although the movies are dense by design because nucleosomes are much more constrained than transcription factors, all datasets were acquired on the same system with identical parameters, and the localization precision and detection density fall within our predefined acceptance windows (see Methods). Our conclusions rely on relative comparisons under matched conditions and on statistics that are insensitive to rare linking errors (e.g., MSD at 0.5 s (or 3 s)). We therefore did not perform additional error analyses.

The RAD21 depletion should be followed by time-course depletion vs mobility experiment rather only 1 hour time point. Do the effects become prominent over time?

We thank Reviewer #3 for this suggestion. A RAD21 depletion time course using the AID2 system has been characterized previously (Yesbolatova et al., 2020), showing that 1 h is sufficient to achieve near-complete cohesin depletion in HCT116 cells. Because shorter depletion minimizes secondary (indirect) effects, we used a 1 h depletion to examine cohesin's impact on nucleosome motion. In response to this point, also raised by Reviewer #1, we performed 1, 3, and 6 h depletions and obtained results consistent with the 1 h condition, indicating that the

effect is largely established by 1 h (Fig. S9k).

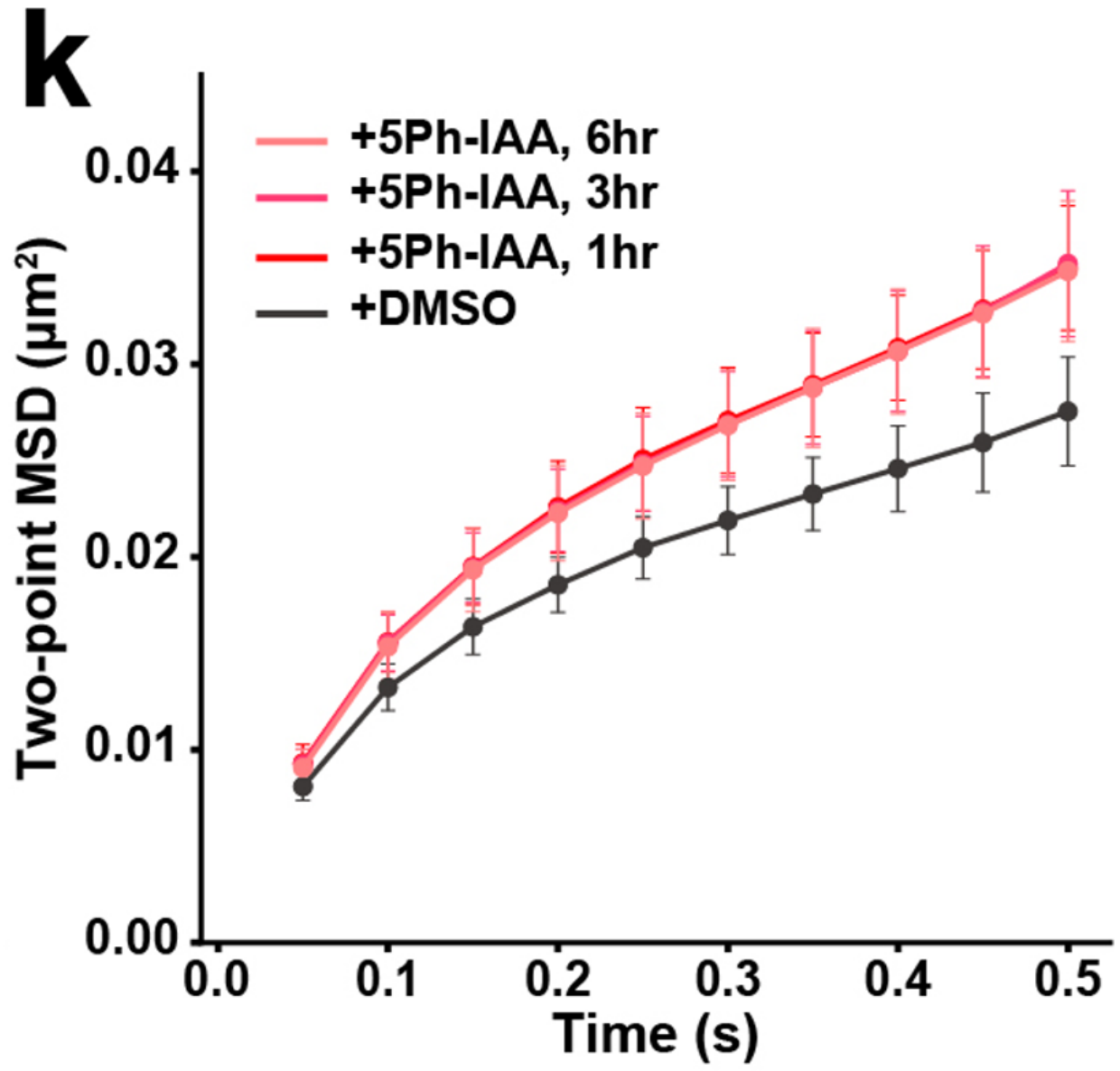

Cohesin loss often leaves transcription surprisingly unaffected in the short term (e.g., Rao et al., 2017). If cohesin loss increases euchromatic domain mixing, does this affect transcription? The authors should discuss why domain mixing might be primarily a biophysical effect without immediate transcriptional consequences. Ideally, include nascent RNA (EU-seq/PRO-seq) or ATAC-seq under the same perturbations
What does mixing mean? How is this quantified? A consistent metric needs to be developed and applied and tested rigorously. For example loss of boundary insulation, increased collisions across domains.

We thank Reviewer #3 for raising these important points. They prompted us to clarify what we mean by domain mixing (i.e., increased domain liquidity/fluidity). We pulled down H3.3-Halo–labeled nucleosomes in HCT116 cells, mapped them genome-wide, and integrated genomic datasets collected with and without cohesin (Rao et al., 2017), including Hi-C, ChIP-seq (cohesin, CTCF, NIPBL, RNA Pol II), and ATAC-seq (Figs. 4 and S5). We then quantified euchromatic domain mixing in Hi-C terms as follows:

1. H3.3-Halo domains overlap Hi-C A compartment with active histone marks and are excluded from the region with repressive marks (Figs. 4 and S5).
2. Cohesin, CTCF, and TAD boundaries are enriched near borders between H3.3-Halo domains; RNA Pol II also shows enrichment (Figs. 6 and S8n-s).
3. Hi-C (Rao et al., 2017) reveals that cohesin loss decreases short-range contacts (~0.1–1.5 Mb, TAD scale) and increases long-range contacts (Mb scale), providing genomic evidence of domain mixing and clarifying our definition in Hi-C terms (Fig. 8a).

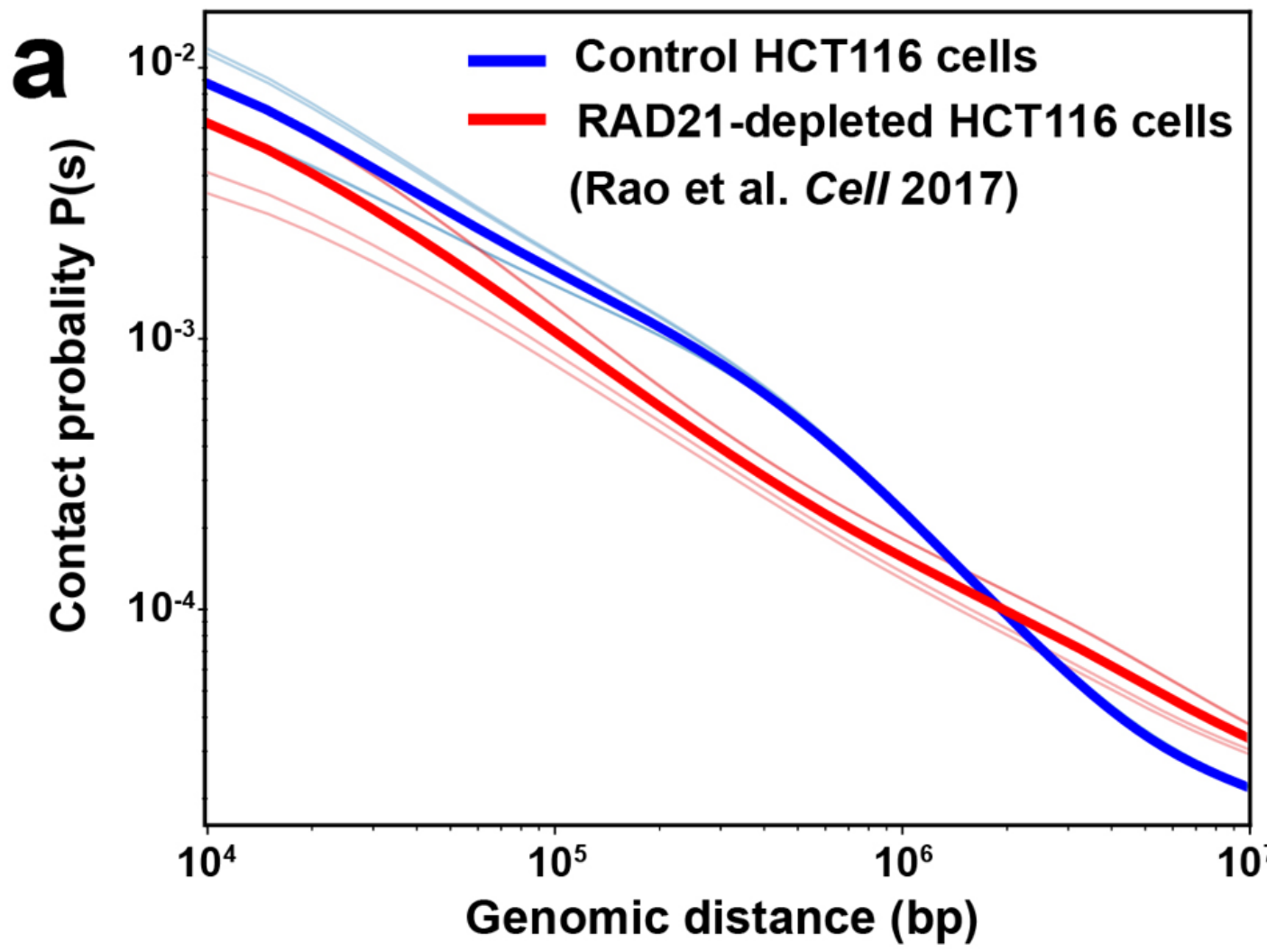

"**Local domain mixing upon cohesin depletion**
As described above, cohesin loss increased nucleosome fluidity within condensed euchromatic domains. In this state, domains may become less constrained and more flexible, permitting inter-domain mixing. Consistently, Hi-C contact probability plot in HCT116 cells (Rao et al., 2017) shows that cohesin loss decreases short-range contacts (~0.1–1.5 Mb, TAD scale) and increases long-range contacts (Mb scale) (Fig. 8a). This effect is slightly enhanced within A compartment regions (Fig. S10a). These findings provide genomic evidence of "domain mixing" and align with previous studies (Dong et al., 2024; Hafner et al., 2023)."

We address transcriptional effects (including ATAC-seq) below.

The authors refer to all cohesin activity as "loop formation", it would be more technically accurate to simply refer to it as extrusion as loop formation itself is not tested or quantified in this manuscript. The authors should consider connecting their measurements to a polymer model with and without cohesin activity, several such models have been developed over the years. For example by explicitly connecting mixing to extrusion. Polymer simulations incorporating loop extrusion, with and without cohesin, could be compared to both MSD plots and a mixing index.

We thank Reviewer #3 for the helpful suggestions on terminology and modeling.

Regarding terminology: because our experiments do not distinguish among mechanisms by which cohesin organizes loops (e.g., active loop extrusion versus loop capture (Davidson and Peters, 2021; Uhlmann, 2025)), we avoid mechanism-specific wording. To remain neutral and fair, we retain "loop formation," and use "loop extrusion" only when citing prior work. We also state explicitly that our data do not test extrusion per se.

Regarding modeling: as suggested, we have added a minimal polymer model with and without cohesin activity and compared simulations to our readouts (new Fig. 8b-d). The model produces condensed loop domains under cohesin activity. Removing cohesin activity reproduces the experimental signatures: increased mixing (mixing scores) (Fig. 8d), and an MSD upshift (Fig. S10b,c). Simulation parameters and code are provided in the Methods and zenodo (https://doi.org/10.5281/zenodo.18404051).

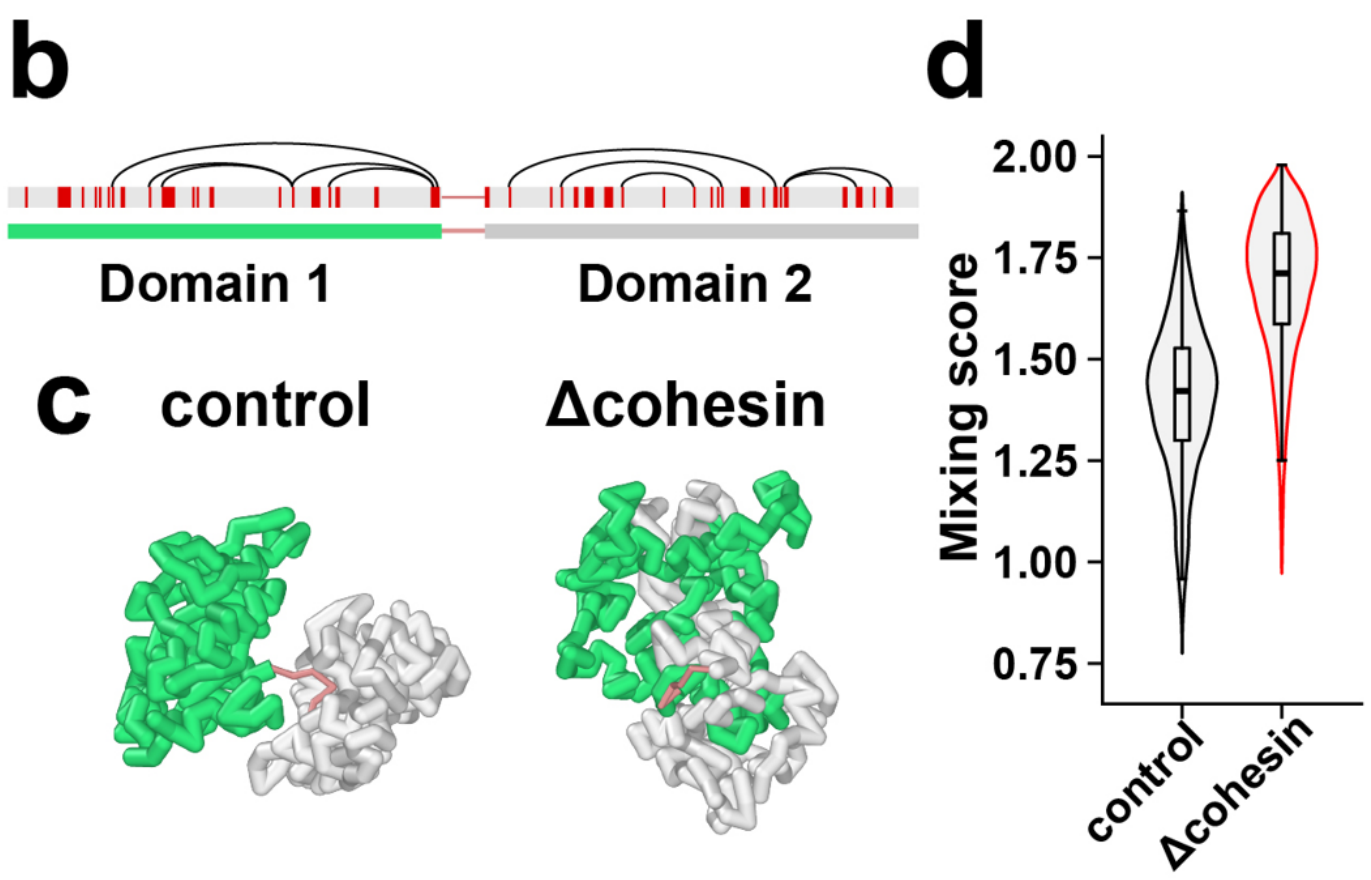

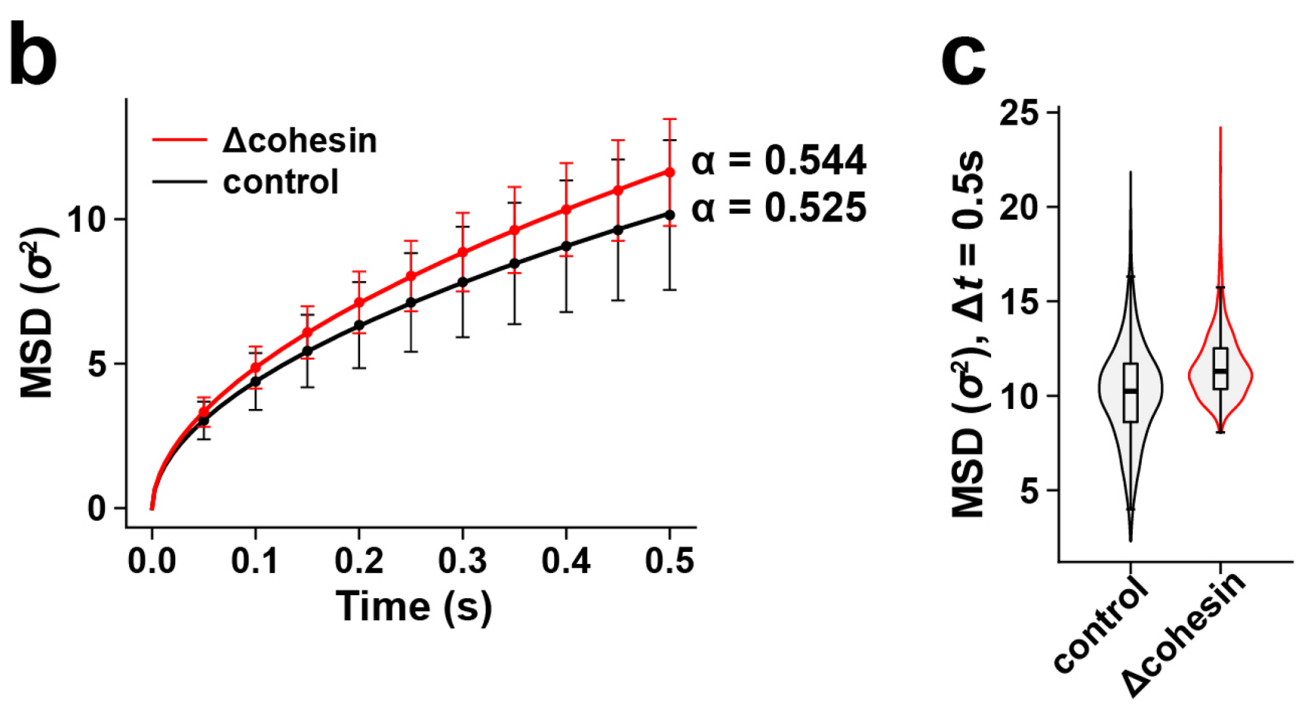

The effects of the AP21967 treatment need to be assessed, the authors should show controls that extrusion is effectively inhibited.

We thank Reviewer#3 for this point. AP21967 alone had no detectable effect on MSD in our hands (Fig. S3i).

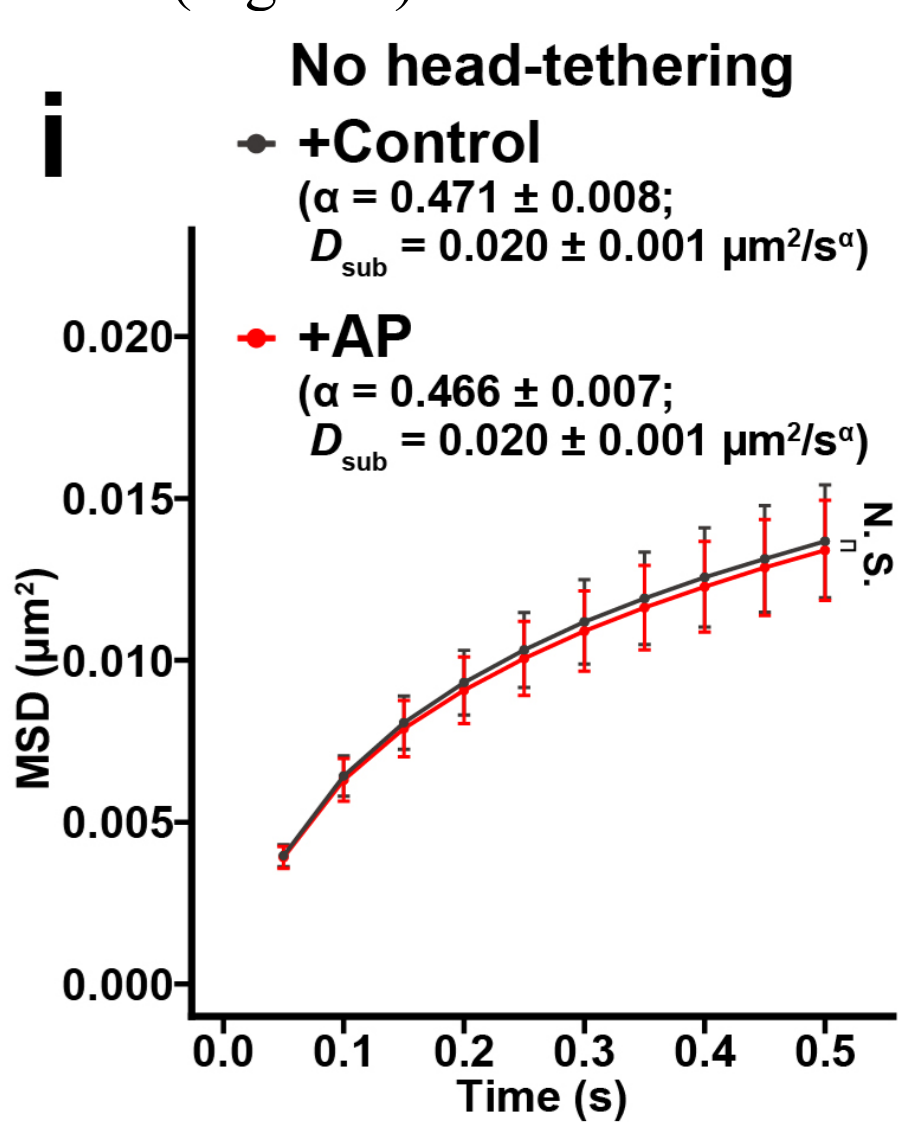

As for extrusion, the same AP (rapamycin analog)-inducible FKBP–FRB head-engagement used here was shown to block cohesin loop extrusion *in vitro* (Sakata et al., 2021); we used the same components and dosing. Following Reviewer#2's suggestion, we toned down the conclusion. We described this point in the Results:

"Head-tethering weakened loop-extrusion activity *in vitro* (Sakata et al., 2021). Head-tethering alone did not change nucleosome motion on the 0.5 s timescale, but head-tethering after RAD21 depletion led to the loss of the entire cohesin complex (Sakata et al., 2021) and increased motion (Fig. 3j,k, S3h). These results suggest that chromatin constraint by cohesin may not require active loop extrusion."

-The claim that local compaction of H3.3 is unchanged upon RAD21 depletion should be validated with higher resolution imaging such as DNA PAINT, STORM that has up to ~20 nm resolution rather than simply from chromatin classes quantification.

We agree with Reviewer #3 that adding data from an independent super-resolution modality would strengthen the claim. We therefore complemented the chromatin-class analysis with higher-resolution imaging: STORM of H3.3–Halo. Nucleosome-cluster analysis showed only a slight change in local H3.3 compaction upon RAD21 depletion. These results are provided in Fig. 7f and detailed in the Methods.

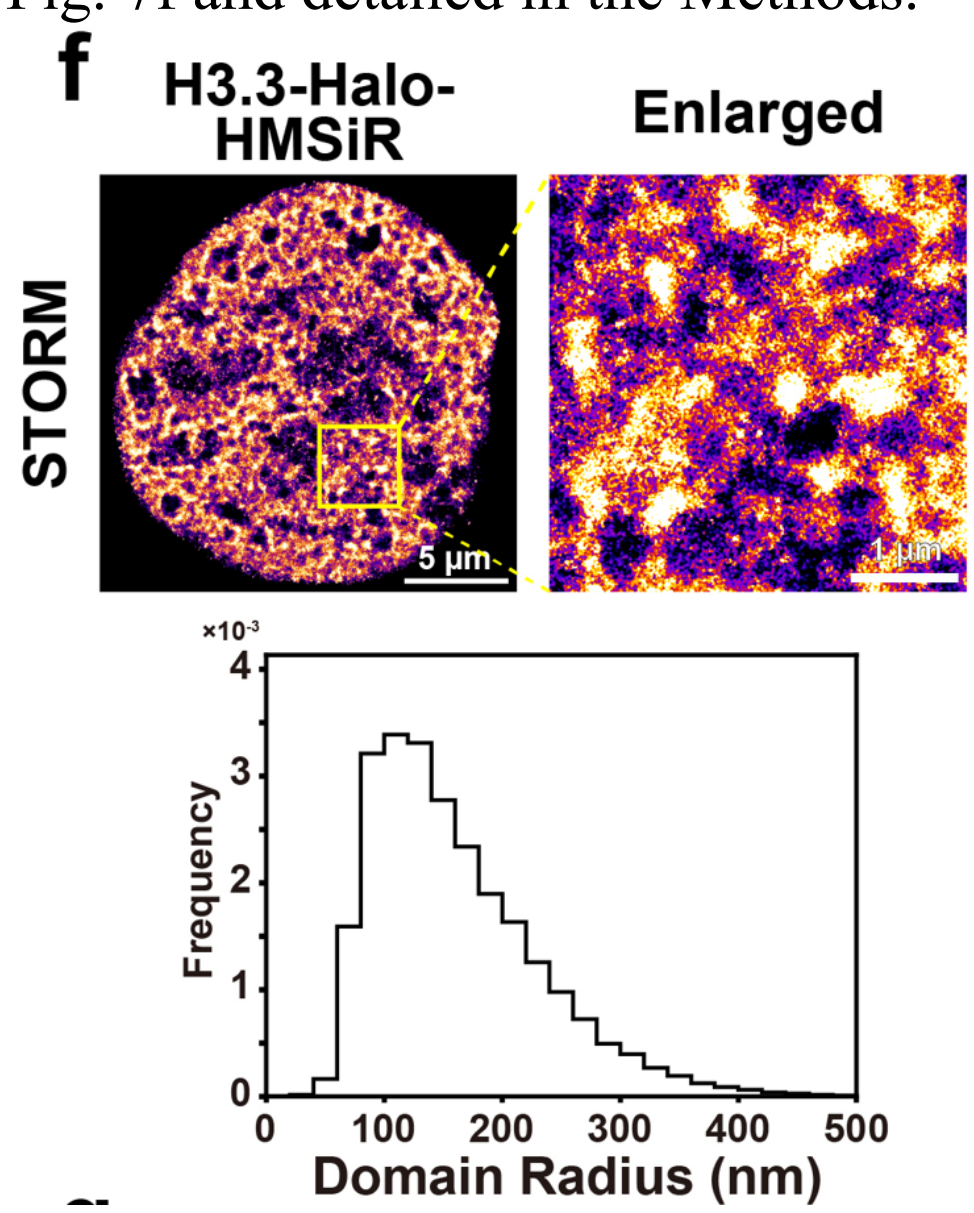

"Furthermore, we used STORM imaging of H3.3-Halo-HMSiR in HCT116 cells, another super-resolution method with ~5 nm resolution (Fig. S8f) (Rust et al., 2006), to investigate euchromatic domain organization. Reconstructed STORM images revealed condensed euchromatin domains similar to those observed by 3D-SIM. Clustering analysis of nucleosomes using SR-Tesseler-DBSCAN pipeline (Levet et al., 2015) yielded a peak domain radius of ~100–120 nm (diameter of ~200–240 nm, Fig. 5f)."

Authors conclude local mixing of euchromatin domains upon RAD21 depletion without direct quantification. Does RAD21 depletion make domains bigger in size without density changes? DNA PAINT is well suited to answer that. Also, does this have functional consequence such as co-expression of genes from neighboring domains? Can authors test this? The lack of functional analysis with regards to transcription is a major shortcoming of this manuscript.

We thank Reviewer #3 for these points. We quantified possible structural changes of euchromatic domains using STORM and image-based metrics. As described above, nucleosome-cluster analysis shows a slight decrease in local H3.3 cluster size upon RAD21 depletion, consistent with higher liquidity rather than domain enlargement (Fig. 7d). We also provided genomic evidence of domain mixing and clarified our definition in Hi-C terms (Fig. 8a): Cohesin loss decreases short-range contacts (~0.1–1.5 Mb, TAD scale) and increases long-range contacts (Mb scale).

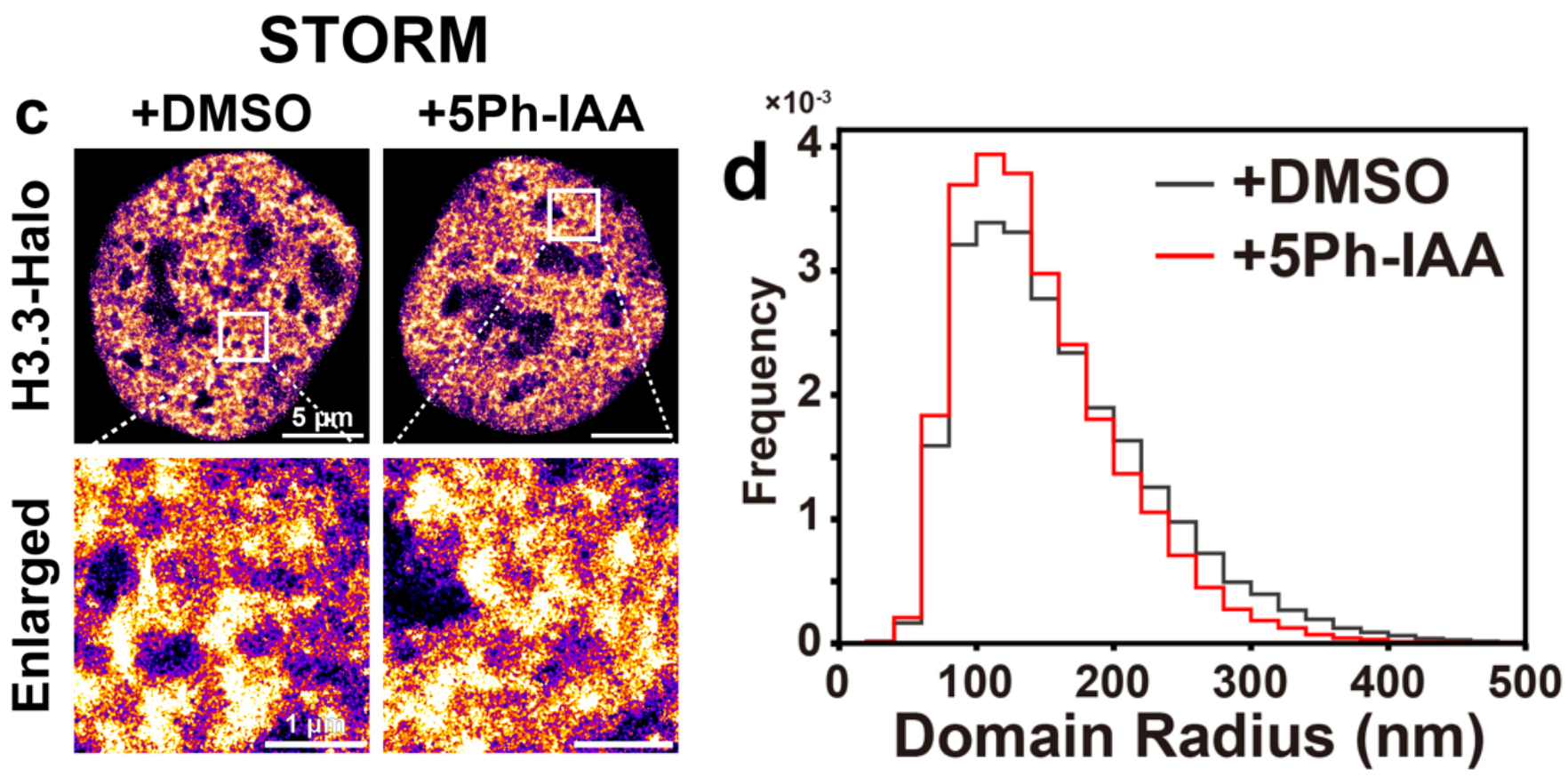

"Reconstructed STORM images after cohesin depletion showed a similar condensed domain organization (Fig. 7c). SR-Tesseler-DBSCAN analysis (Levet et al., 2015) of the reconstructed images further reveals that cohesin depletion slightly increased the fraction of domains with smaller radii (~100–120 nm) and decreased the large-radius tail (~200–400 nm) (Fig. 7d), consistent with domain splitting upon cohesin loss (Nozaki et al., 2023; Paggi and Zhang, 2025). The modal radius was largely maintained. Together, these super-resolution results indicate that cohesin depletion retains overall domain compaction while increasing nucleosome mobility."

Furthermore, two-point MSD of nearby nucleosomes extended to 2 s shows increased fluctuation of nucleosomes with RAD21 depletion (Fig. S9l), strengthening the increase in the liquidity of nucleosomes.

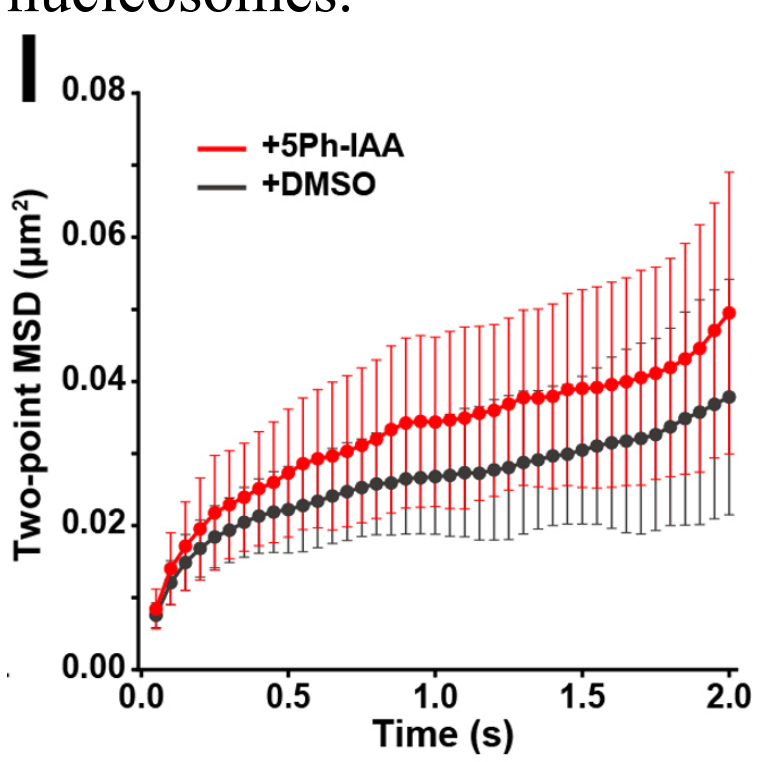

Regarding functional readouts. In HCT116, intron-FISH reveals increased co-activation of neighboring genes with only modest changes in means (Fig. 8e-j). These additions provide compact, quantitative evidence of mixing and its consequences in our system.

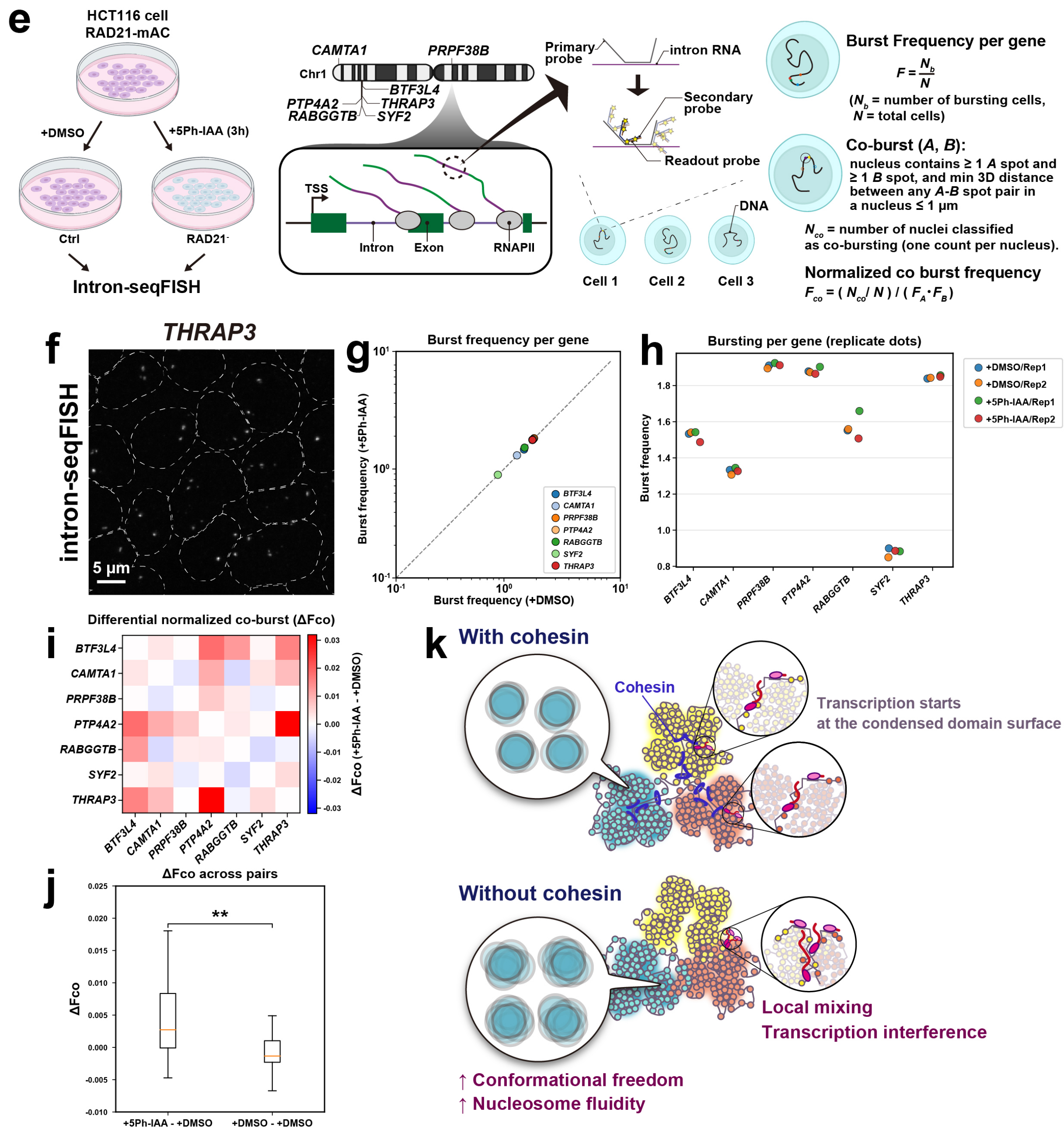

"Because acute cohesin loss often produces only modest changes in bulk expression (Rao et al., 2017) and in ATAC-seq signals in HCT116 cells (Fig. S10f), we reasoned that transcriptional bursting—particularly its *cis* coordination between loci—might provide a more sensitive functional readout. We therefore quantified this *cis* coordination of transcriptional bursting in HCT116 using intron-seqFISH (intron sequential fluorescence *in situ* hybridization) (Figs. 8e,f, S10g,h) (Ohishi et al., 2024; Shah et al., 2018). We selected 7 genes on Chromosome 1 whose per-gene burst frequency changed minimally upon RAD21 depletion (Fig. 8g,h; see Methods for details), enabling us to assess changes in co-bursting independently of large shifts in single-gene activity. Notably, the co-burst frequency of gene pairs significantly increased upon cohesin depletion (Fig. 8i,j), suggesting that cohesin helps prevent gene co-bursting along the chromosome, consistent with a previous study in mouse ES cells (Dong et al., 2024). Thus,

cohesin depletion increases burst coupling between genes even when per-gene bursting remains largely unchanged, plausibly via local domain mixing (Fig. 8k)."

Authors conclude Pol II localization on domain surfaces without proper quantification. Authors need to perform more rigorous and quantitative analysis on the spatial mapping of RNA Pol II (and elongating and initiating forms) and euchromatin domains which also could be perhaps better answered with two color DNA PAINT of H3.3 and Pol II variants. How does this spatial relation change upon triptolide and alpha- amanitin treatment?

We thank Reviewer #3 for this point. Following the SIM analysis by Miron et al. (Miron et al., 2020), we quantitatively show that Pol II Ser5P signal is enriched toward the periphery of H3.3 domains. Because domain "surfaces" also exist along the z-axial (top and bottom) directions, our current axial resolution does not allow unambiguous assignment to the true 3D surface, so we avoid stronger claims.

Consistently, genomic analyses in H3.3-Halo domains in HCT116 shows RNAPII and other active transcription markers enriched near H3.3-Halo domain borders (new Fig. 6). Taken together, these analyses support that initiating Pol II concentrates around the periphery of condensed euchromatin domains, which we note as a new observation.

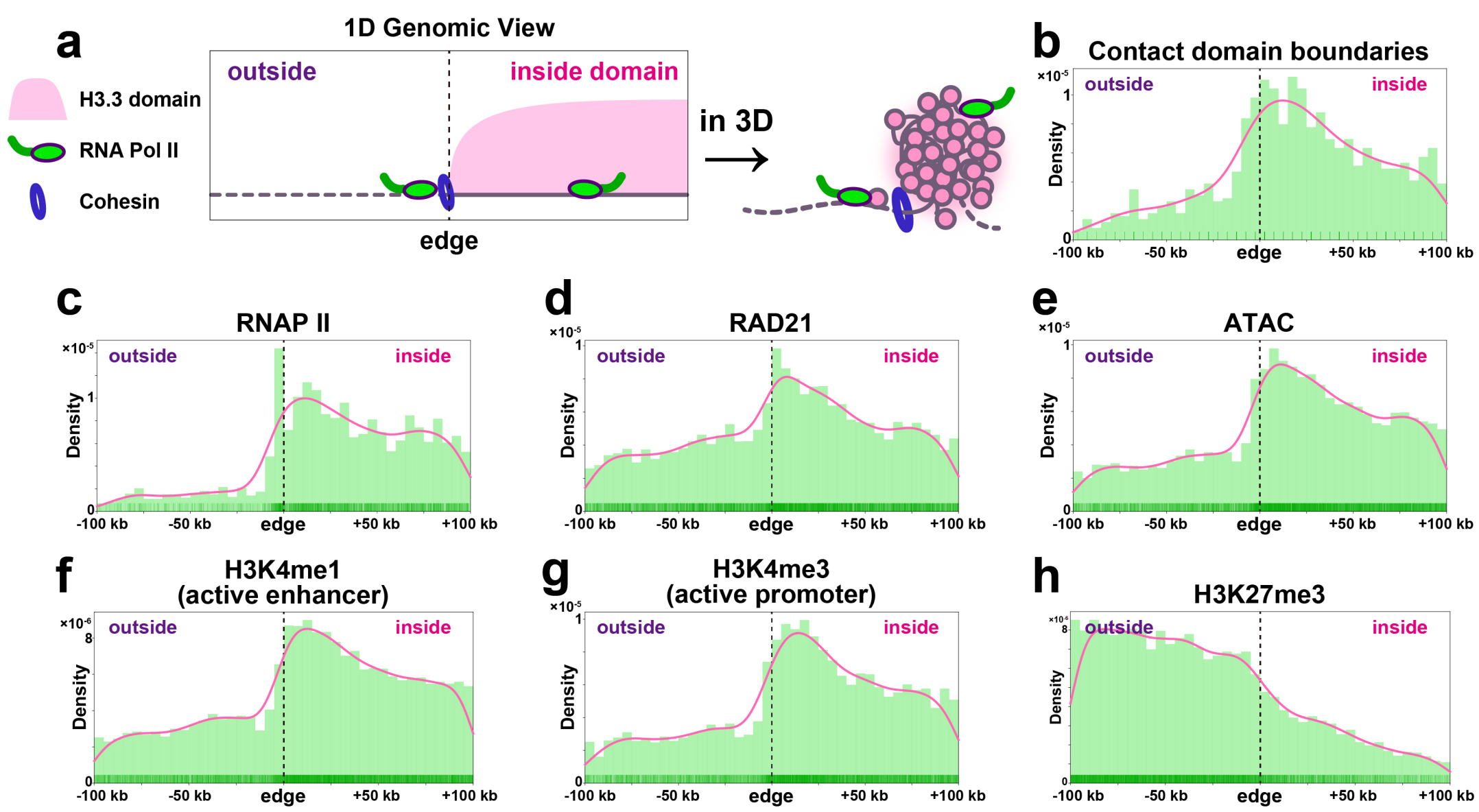

Regarding transcription-inhibitor treatments, we agree they are intriguing; however, such inhibitors are known to alter chromatin dynamics/structure (condensed) in complex ways (Neguembor et al., 2021). A systematic analysis is beyond the scope of this study, and we consider it a priority for future work.

Minor Comments:

Multiple groups (e.g., Hansen et al., Science 2022; Wutz et al., Nature 2017) have shown cohesin loss increases mobility and reduces loop structures. The novelty here is

the “euchromatic domain mixing” angle. The authors should position their findings relative to these. Is “mixing” essentially another readout of weakened insulation, or does it capture a new phenomenon at the nucleosome scale?

We thank Reviewer #3 for this excellent suggestion. Together with the genomic data described above, we now emphasize “euchromatic domain mixing” and clarify that “mixing” is essentially another readout of weakened insulation:

“Together, domain mixing upon cohesin loss is essentially another readout of weakened insulation between domains. Condensed euchromatic domains constrained by cohesin ensure effective insulation (Hnisz et al., 2016; Wendt et al., 2008) of transcriptional programs between domains.”

Can authors explain the higher variability in the locus tracking measurement ? Why is the MSD shown only up to 0.5s for these since these loci could potentially be tracked for much longer ?

We thank Reviewer #3 for the question.

Higher variability: Each locus provides only one trajectory per cell, so sampling is sparse. The *lacO*/EGFP-LacI and *tetO*/TetR-4×mCherry foci are a multi-emitter spot (many LacI/TetR) whose shape/intensity fluctuate, reducing localization precision. These factors make locus MSD noisier than single-nucleosome imaging/tracking. This point is described in the Fig. 2f legend.

Why MSD to 0.5 s: We restrict lags to 0.5 s to match the spatiotemporal resolution of single-nucleosome imaging/tracking and to minimize contamination from nuclear/cell drift, which dominates at longer lags.

“Note that the following two factors make locus MSD noisier than single-nucleosome imaging/tracking. First, each locus provides only one trajectory per cell, so sampling is sparse. Another factor is that the *lacO*/EGFP-LacI- and *tetO*/TetR-4×mCherry foci are multi-emitter spots (many LacI/TetR) whose shape/intensity fluctuate, reducing localization precision.”

RAD21 loss significantly increases H3.3 mobility in the nuclear interior but has little effect on peripheral H2B. This may be due to technical limits in dense chromatin rather than true invariance. The authors should discuss this caveat explicitly and consider additional assays to support the biological interpretation.

We thank Reviewer #3 for pointing out this issue. We realized that the Methods description was not sufficiently clear. We used an established peripheral single-nucleosome imaging/tracking method with oblique illumination (HILO) at a lower angle (Nozaki et al., 2017) and Fig. 4f in (Nagashima et al., 2019). Localization precision, detection density, and track lengths at the nuclear bottom surface are comparable to, or even better than, those in the interior, indicating no technical limits (Position determination accuracy: 7.3 nm). The small effect on peripheral H2B is therefore likely biological, consistent with low cohesin at the nuclear periphery and enrichment of lamina-associated B compartment chromatin. We have clarified this point in the Methods.

g

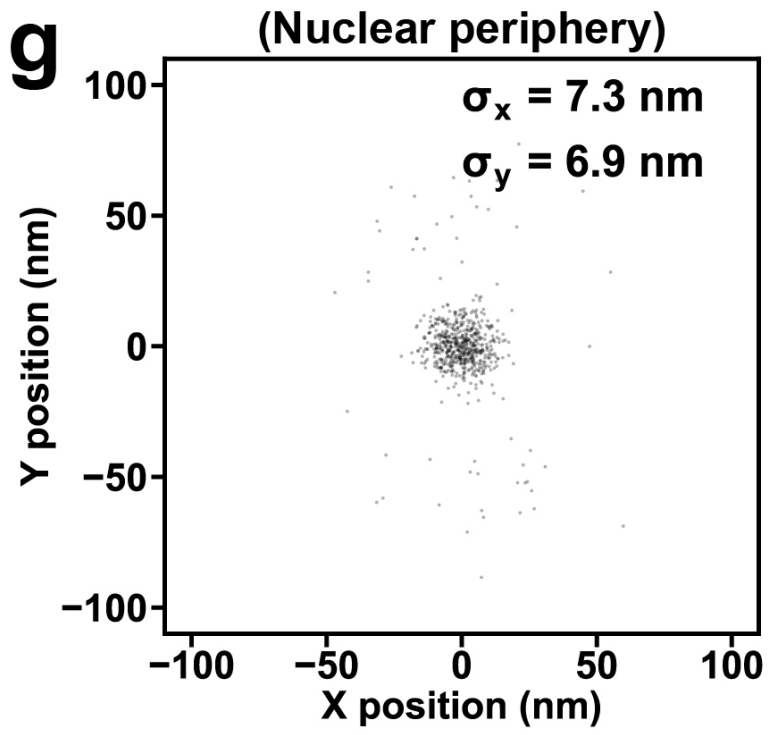

“To visualize the basal nuclear surface (nuclear periphery), we adjusted the angle of laser illumination to efficiently capture a nuclear membrane marker, NUP107-Venus (Maeshima et al., 2010). Almost uniform distributions of NUP107-Venus were observed in the nuclear periphery condition, while the nuclear interior condition showed a rim of NUP107-Venus (Nagashima et al., 2019). As reported previously (Nagashima et al., 2019; Shinkai et al., 2016), nucleosome dynamics on the nuclear surface were lower than those of the nuclear interior, which contained more euchromatin regions. Position determination accuracy is 7.3 nm (Fig. S7g).”

Abstract:“Physical properties remain elusive” is a bit vague please specify which physical properties.

As suggested by Reviewer #1, we changed the sentence as follows:
“However, how cohesin organizes these domains in living cells, especially in active euchromatin, remains elusive.”

”Abstract
The human genome is folded into chromatin loops by the cohesin complex, forming functional chromatin domains that underlie transcription and DNA replication/repair. However, how cohesin organizes these domains in living cells, especially in active euchromatin, remains elusive. To address these questions, we combined single-nucleosome imaging/tracking and super-resolution 3D-structured illumination microscopy (3D-SIM) with euchromatin-specific labeling of histone H3.3. Using this nanoscopic approach, we revealed that euchromatin forms condensed domains that are constrained by cohesin-mediated loops. This organization refines the classical textbook view of euchromatin as largely open, in line with emerging evidence. Transcription machinery appear to be located near the condensed domain surfaces/borders. Cohesin loss increased nucleosome fluidity within these domains without altering their compaction, leading to local mixing of domains and compromising transcriptional insulation. These findings uncover an unexpected physical role of cohesin in maintaining the integrity of condensed euchromatic domains and ensuring proper higher-order regulation of gene expression.”

Introduction:

Line 6> genome -> genomic

We corrected it.

Page 6, line 7: loop formation and extrusion are not necessarily the same, the former being rare. The authors should be careful on which they are attributing changes to.

As we described above, our experiments do not distinguish among mechanisms by which cohesin organizes loops (e.g., active loop extrusion versus loop capture). To avoid mechanism-specific wording and keep a balanced view, we would like to retain "loop formation," and use "loop extrusion" only when citing prior work.

Reviewer #3 (Remarks on code availability):

Code is well commented, documented, and is executable.

Uncategorized References

Banaszynski, L.A., D. Wen, S. Dewell, S.J. Whitcomb, M. Lin, N. Diaz, S.J. Elsasser, A. Chapgier, A.D. Goldberg, E. Canaani, S. Rafii, D. Zheng, and C.D. Allis. 2013. Hira-dependent histone H3.3 deposition facilitates PRC2 recruitment at developmental loci in ES cells. *Cell*. 155:107–120.

Bayle, J.H., J.S. Grimley, K. Stankunas, J.E. Gestwicki, T.J. Wandless, and G.R. Crabtree. 2006. Rapamycin analogs with differential binding specificity permit orthogonal control of protein activity. *Chem Biol*. 13:99–107.

Cremer, M., V.J. Schmid, F. Kraus, Y. Markaki, I. Hellmann, A. Maiser, H. Leonhardt, S. John, J. Stamatoyannopoulos, and T. Cremer. 2017. Initial high-resolution microscopic mapping of active and inactive regulatory sequences proves non-random 3D arrangements in chromatin domain clusters. *Epigenetics Chromatin*. 10:39.

Davidson, I.F., and J.M. Peters. 2021. Genome folding through loop extrusion by SMC complexes. *Nat Rev Mol Cell Biol*. 22:445–464.

Dion, V., and S.M. Gasser. 2013. Chromatin movement in the maintenance of genome stability. *Cell*. 152:1355–1364.

Dong, P., S. Zhang, V. Gandin, L. Xie, L. Wang, A.L. Lemire, W. Li, H. Otsuna, T. Kawase, A.D. Lander, H.Y. Chang, and Z.J. Liu. 2024. Cohesin prevents cross-domain gene coactivation. *Nat Genet*. 56:1654–1664.

Eykelenboom, J.K., M. Gierlinski, Z. Yue, N. Hegarat, H. Pollard, T. Fukagawa, H. Hochegger, and T.U. Tanaka. 2019. Live imaging of marked chromosome regions reveals their dynamic resolution and compaction in mitosis. *J Cell Biol*. 218:1531–1552.

Fang, H.T., C.A. El Farran, Q.R. Xing, L.F. Zhang, H. Li, B. Lim, and Y.H. Loh. 2018. Global H3.3 dynamic deposition defines its bimodal role in cell fate transition. *Nat Commun*. 9:1537.

Gabriele, M., H.B. Brandao, S. Grosse-Holz, A. Jha, G.M. Dailey, C. Cattoglio, T.S. Hsieh, L. Mirny, C. Zechner, and A.S. Hansen. 2022. Dynamics of CTCF- and cohesin-mediated chromatin looping revealed by live-cell imaging. *Science*. 376:496–501.

Goldberg, A.D., L.A. Banaszynski, K.M. Noh, P.W. Lewis, S.J. Elsaesser, S. Stadler, S. Dewell, M. Law, X. Guo, X. Li, D. Wen, A. Chapgier, R.C. DeKelver, J.C. Miller, Y.L. Lee, E.A. Boydston, M.C. Holmes, P.D. Gregory, J.M. Greally, S. Rafii, C. Yang, P.J. Scambler, D. Garrick, R.J. Gibbons, D.R. Higgs, I.M. Cristea, F.D. Urnov, D. Zheng, and C.D. Allis. 2010. Distinct factors control histone variant H3.3 localization at specific genomic regions. *Cell*. 140:678–691.

Grunberg, R., T.S. Ferrar, A.M. van der Sloot, M. Constante, and L. Serrano. 2010. Building blocks for protein interaction devices. *Nucleic Acids Res*. 38:2645–2662.

Hafner, A., M. Park, S.E. Berger, S.E. Murphy, E.P. Nora, and A.N. Boettiger. 2023. Loop stacking organizes genome folding from TADs to chromosomes. *Mol Cell*. 83:1377–1392 e1376.

Hansen, A.S., M. Woringer, J.B. Grimm, L.D. Lavis, R. Tjian, and X. Darzacq. 2018. Robust model-based analysis of single-particle tracking experiments with Spot-On. *eLife*. 7.

Hibino, K., Y. Sakai, S. Tamura, M. Takagi, K. Minami, T. Natsume, M.A. Shimazoe, M.T. Kanemaki, N. Imamoto, and K. Maeshima. 2024. Single-nucleosome imaging unveils that condensins and nucleosome-nucleosome interactions differentially constrain chromatin to organize mitotic chromosomes. *Nat Commun*. 15:7152.

Hihara, S., C.G. Pack, K. Kaizu, T. Tani, T. Hanafusa, T. Nozaki, S. Takemoto, T. Yoshimi, H. Yokota, N. Imamoto, Y. Sako, M. Kinjo, K. Takahashi, T. Nagai, and K. Maeshima. 2012. Local nucleosome dynamics facilitate chromatin accessibility in living mammalian cells. *Cell Rep*. 2:1645–1656.

Hnisz, D., D.S. Day, and R.A. Young. 2016. Insulated Neighborhoods: Structural and Functional Units of Mammalian Gene Control. *Cell*. 167:1188–1200.

Iida, S., S. Shinkai, Y. Itoh, S. Tamura, M.T. Kanemaki, S. Onami, and K. Maeshima. 2022. Single-nucleosome imaging reveals steady-state motion of interphase chromatin in living human cells. *Science Advances.* 8:eabn5626.

Kalashnikova, A.A., M.E. Porter-Goff, U.M. Muthurajan, K. Luger, and J.C. Hansen. 2013. The role of the nucleosome acidic patch in modulating higher order chromatin structure. *J R Soc Interface*. 10:20121022.

Levet, F., E. Hosy, A. Kechkar, C. Butler, A. Beghin, D. Choquet, and J.B. Sibarita. 2015. SR-Tesseler: a method to segment and quantify localization-based super-resolution microscopy data. *Nat Methods*. 12:1065–1071.

Maeshima, K., H. Iino, S. Hihara, T. Funakoshi, A. Watanabe, M. Nishimura, R. Nakatomi, K. Yahata, F. Imamoto, T. Hashikawa, H. Yokota, and N. Imamoto. 2010. Nuclear pore formation but not nuclear growth is governed by cyclin-dependent kinases (Cdks) during interphase. *Nat Struct Mol Biol*. 17:1065–1071.

Minami, K., K. Nakazato, S. Ide, K. Kaizu, K. Higashi, S. Tamura, A. Toyoda, K. Takahashi, K. Kurokawa, and K. Maeshima. 2025. Replication-dependent histone labeling dissects the physical properties of euchromatin/heterochromatin in living human cells. *Sci Adv*. 11:eadu8400.

Miron, E., R. Oldenkamp, J.M. Brown, D.M.S. Pinto, C.S. Xu, A.R. Faria, H.A. Shaban, J.D.P. Rhodes, C. Innocent, S. de Ornellas, H.F. Hess, V. Buckle, and L. Schermelleh. 2020. Chromatin arranges in chains of mesoscale domains with nanoscale functional topography independent of cohesin. *Sci Adv*. 6.

Nagashima, R., K. Hibino, S.S. Ashwin, M. Babokhov, S. Fujishiro, R. Imai, T. Nozaki, S. Tamura, T. Tani, H. Kimura, M. Shribak, M.T. Kanemaki, M. Sasai, and K. Maeshima.

2019. Single nucleosome imaging reveals loose genome chromatin networks via active RNA polymerase II. *J Cell Biol*. 218:1511–1530.

Neguembor, M.V., L. Martin, A. Castells-Garcia, P.A. Gomez-Garcia, C. Vicario, D. Carnevali, J. AlHaj Abed, A. Granados, R. Sebastian-Perez, F. Sottile, J. Solon, C.T. Wu, M. Lakadamyali, and M.P. Cosma. 2021. Transcription-mediated supercoiling regulates genome folding and loop formation. *Mol Cell*. 81:3065–3081 e3012.

Nozaki, T., R. Imai, M. Tanbo, R. Nagashima, S. Tamura, T. Tani, Y. Joti, M. Tomita, K. Hibino, M.T. Kanemaki, K.S. Wendt, Y. Okada, T. Nagai, and K. Maeshima. 2017. Dynamic Organization of Chromatin Domains Revealed by Super-Resolution Live-Cell Imaging. *Mol Cell*. 67:282–293 e287.

Nozaki, T., S. Shinkai, S. Ide, K. Higashi, S. Tamura, M.A. Shimazoe, M. Nakagawa, Y. Suzuki, Y. Okada, M. Sasai, S. Onami, K. Kurokawa, S. Iida, and K. Maeshima. 2023. Condensed but liquid-like domain organization of active chromatin regions in living human cells. *Sci Adv*. 9:eadf1488.

Ohishi, H., S. Shinkai, H. Owada, T. Fujii, K. Hosoda, S. Onami, T. Yamamoto, Y. Ohkawa, and H. Ochiai. 2024. Transcription-coupled changes in genomic region proximities during transcriptional bursting. *Sci Adv*. 10:eadn0020.

Otterstrom, J., A. Castells-Garcia, C. Vicario, P.A. Gomez-Garcia, M.P. Cosma, and M. Lakadamyali. 2019. Super-resolution microscopy reveals how histone tail acetylation affects DNA compaction within nucleosomes in vivo. *Nucleic Acids Res*. 47:8470–8484.

Paggi, J.M., and B. Zhang. 2025. Euchromatin forms condensed domains with short active regions on the surface. *bioRxiv*.

Rao, S.S.P., S.C. Huang, B. Glenn St Hilaire, J.M. Engreitz, E.M. Perez, K.R. Kieffer-Kwon, A.L. Sanborn, S.E. Johnstone, G.D. Bascom, I.D. Bochkov, X. Huang, M.S. Shamim, J. Shin, D. Turner, Z. Ye, A.D. Omer, J.T. Robinson, T. Schlick, B.E. Bernstein, R. Casellas, E.S. Lander, and E.L. Aiden. 2017. Cohesin Loss Eliminates All Loop Domains. *Cell*. 171:305–320 e324.

Ricci, M.A., C. Manzo, M.F. Garcia-Parajo, M. Lakadamyali, and M.P. Cosma. 2015. Chromatin fibers are formed by heterogeneous groups of nucleosomes in vivo. *Cell*. 160:1145–1158.

Rust, M.J., M. Bates, and X. Zhuang. 2006. Sub-diffraction-limit imaging by stochastic optical reconstruction microscopy (STORM). *Nat Methods*. 3:793–795.

Sakata, R., K. Niwa, D. Ugarte La Torre, C. Gu, E. Tahara, S. Takada, and T. Nishiyama. 2021. Opening of cohesin's SMC ring is essential for timely DNA replication and DNA loop formation. *Cell Rep*. 35:108999.

Schmid, V.J., M. Cremer, and T. Cremer. 2017. Quantitative analyses of the 3D nuclear landscape recorded with super-resolved fluorescence microscopy. *Methods*. 123:33–46.

Schneider, M.W.G., B.A. Gibson, S. Otsuka, M.F.D. Spicer, M. Petrovic, C. Blaukopf, C.C.H. Langer, P. Batty, T. Nagaraju, L.K. Doolittle, M.K. Rosen, and D.W. Gerlich. 2022. A mitotic chromatin phase transition prevents perforation by microtubules. *Nature*.

Shah, S., Y. Takei, W. Zhou, E. Lubeck, J. Yun, C.L. Eng, N. Koulena, C. Cronin, C. Karp, E.J. Liaw, M. Amin, and L. Cai. 2018. Dynamics and Spatial Genomics of the Nascent Transcriptome by Intron seqFISH. *Cell*. 174:363–376 e316.

Shinkai, S., T. Nozaki, K. Maeshima, and Y. Togashi. 2016. Dynamic Nucleosome Movement Provides Structural Information of Topological Chromatin Domains in Living Human Cells. *PLoS Comput Biol*. 12:e1005136.

Shinkai, S., T. Nozaki, K. Maeshima, and Y. Togashi. 2017. Bridging the dynamics and organization of chromatin domains by mathematical modeling. *Nucleus*. 8:353–359.
Tedeschi, A., G. Wutz, S. Huet, M. Jaritz, A. Wuensche, E. Schirghuber, I.F. Davidson, W. Tang, D.A. Cisneros, V. Bhaskara, T. Nishiyama, A. Vaziri, A. Wutz, J. Ellenberg, and J.M. Peters. 2013. Wapl is an essential regulator of chromatin structure and chromosome segregation. *Nature*. 501:564–568.
Toth, K.F., T.A. Knoch, M. Wachsmuth, M. Frank-Stohr, M. Stohr, C.P. Bacher, G. Muller, and K. Rippe. 2004. Trichostatin A-induced histone acetylation causes decondensation of interphase chromatin. *J Cell Sci*. 117:4277–4287.
Uhlmann, F. 2025. A unified model for cohesin function in sisterchromatid cohesion and chromatin loop formation. *Mol Cell*. 85:1058–1071.
Wendt, K.S., K. Yoshida, T. Itoh, M. Bando, B. Koch, E. Schirghuber, S. Tsutsumi, G. Nagae, K. Ishihara, T. Mishiro, K. Yahata, F. Imamoto, H. Aburatani, M. Nakao, N. Imamoto, K. Maeshima, K. Shirahige, and J.M. Peters. 2008. Cohesin mediates transcriptional insulation by CCCTC-binding factor. *Nature*. 451:796–801.
Yesbolatova, A., Y. Saito, N. Kitamoto, H. Makino-Itou, R. Ajima, R. Nakano, H. Nakaoka, K. Fukui, K. Gamo, Y. Tominari, H. Takeuchi, Y. Saga, K.I. Hayashi, and M.T. Kanemaki. 2020. The auxin-inducible degron 2 technology provides sharp degradation control in yeast, mammalian cells, and mice. *Nat Commun*. 11:5701.
Yoshida, M., M. Kijima, M. Akita, and T. Beppu. 1990. Potent and specific inhibition of mammalian histone deacetylase both in vivo and in vitro by trichostatin A. *J Biol Chem*. 265:17174–17179.

We thank the editor and the three reviewers for their careful and constructive comments. We have revised the manuscript accordingly and believe that it has been substantially strengthened. Our point-by-point responses are provided below.

Reviewers' Comments:

Reviewer #1 (Remarks to the Author):

The authors addressed all the major points and improved the text with relevant details and the graphs with informative data, enabling a better understanding of their conclusions. The additional work done to address all reviewers' comments further improved the already high quality of this manuscript.
The extra experiments I suggested would indeed be a valuable improvement, but I agree with the authors' reply. Even if they would provide good advice for future experiments, they are not required to support the current conclusions, and their absence does not preclude publication of this work.
My only remaining concern is that the updated figures S2 and S3 would benefit from quantification of acute protein depletion from the Western blot analysis to demonstrate consistency with the fluorescence measurements. Currently, they provide only a qualitative visual representation in a single replicate. Once this is addressed, I strongly recommend publication of this article.

We are grateful for the supportive comments from Reviewer #1.
As suggested, we increased the number of replicates for the Western blot analyses in Figs. S2 and S3 (Current Extended Data Fig. 2 and 3), added the corresponding quantification and statistics, and described them in the figure legends.

Exdended Data Fig. 2

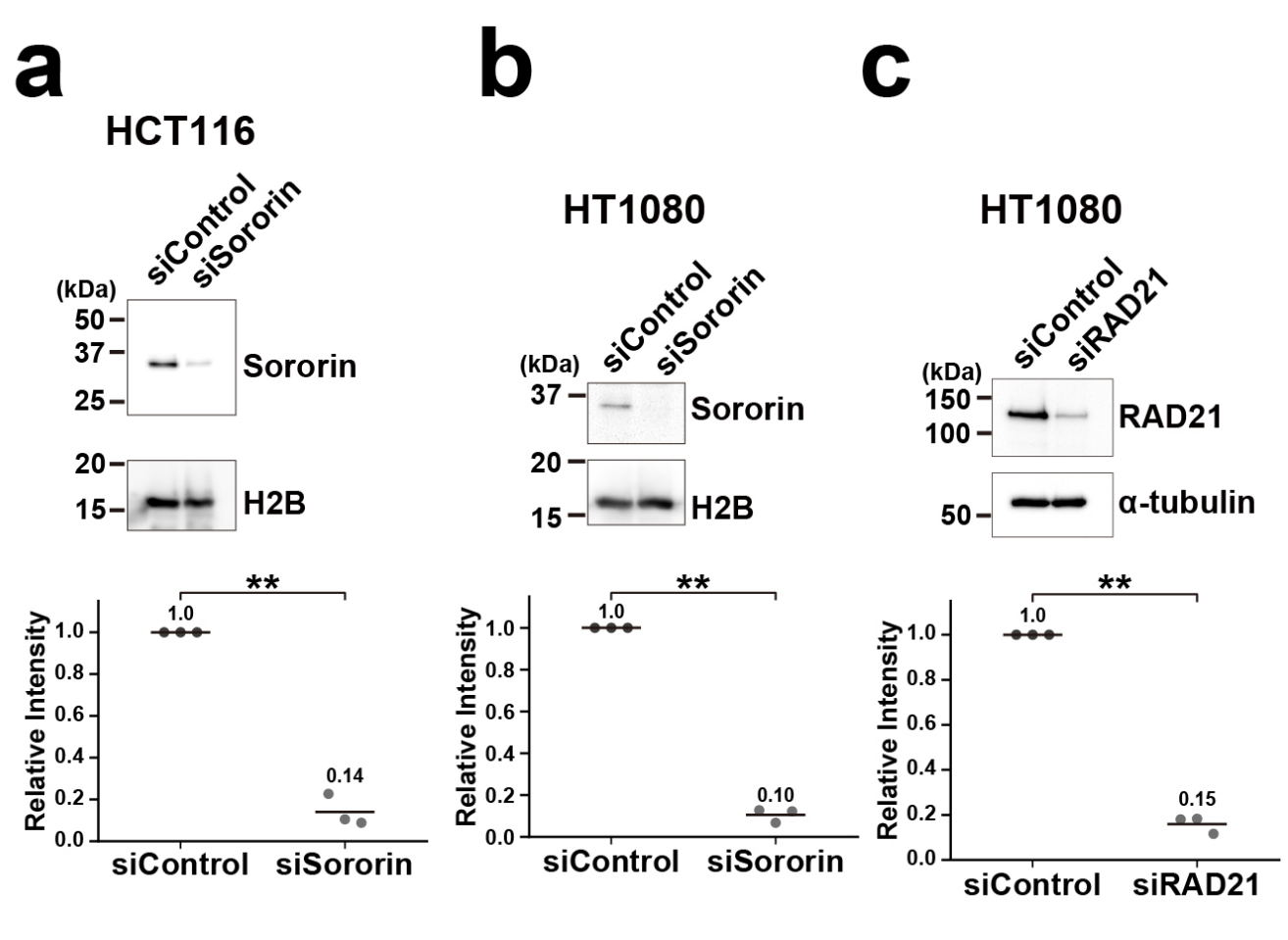

Exdended Data Fig. 3

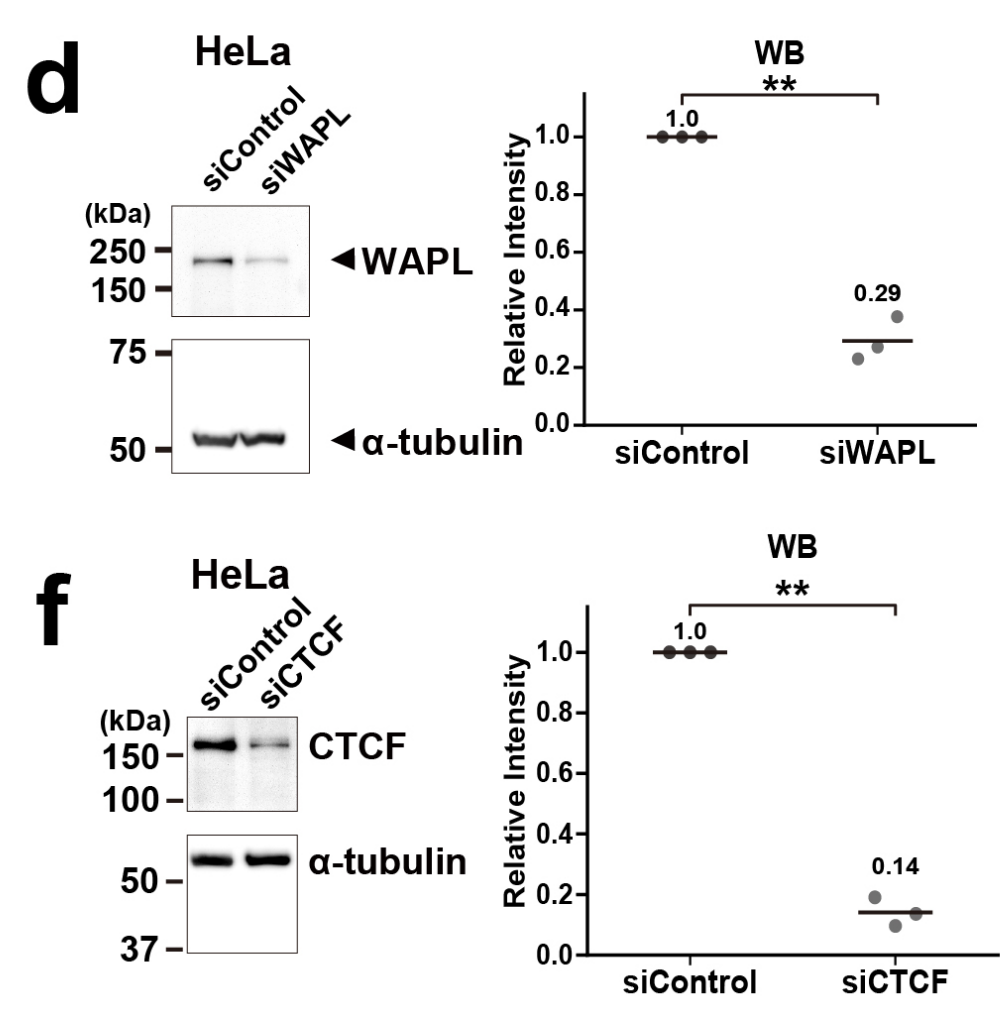

Reviewer #1 (Remarks on code availability):

Now the code description is complete.

Reviewer #2 (Remarks to the Author):

In the revised manuscript of Shimazoe et al., ‘Cohesin prevents local mixing of condensed euchromatic domains in living human cells’ the authors show the effect of loop extrusion by cohesin on the movement of euchromatin nucleosomes. They observe cohesin restricts nucleome mixing and thereby affects transcription, as transcription is normally mainly at the borders of the domains created by cohesin. They find that this is due to the loop extruding pool of cohesin and not the cohesive cohesin. The tracking of nucleosomes is of high quality and performed elegantly. The authors took my remarks into account and answered them accordingly. Conclusions are toned down and mutants are explained in the context of literature. Also the use of models helps the reader to understand the manuscript. I think the manuscript improved and is now more accessible to the broader public, therefore I support publication of the manuscript.

We appreciate the positive comments from Reviewer #2 and the support for publication.

Reviewer #3 (Remarks to the Author):

The authors have done well in addressing many of my concerns. However, most of the concerns about causality and mechanism have mostly been addressed by reframing and softening claims. While the paper is technically strong in many aspects, a direct mechanistic link between cohesin’s extrusion activity, to mixing, and transcription is still not demonstrated. In my opinion the current data can support the conclusion of cohesin-dependence on the physical properties of H3.3 rich domains but does not support mechanistic conclusions on domain mixing and transcription insulation. Thus overall, the paper is convincing on phenomenology rather than mechanism and is thus perceived as model-forward.

We appreciate the Reviewer #3’s thoughtful comments. We agree that our data do not fully establish a direct mechanistic link. Our main conclusion is that cohesin physically constrains H3.3-rich condensed euchromatic domains, a previously underappreciated feature of euchromatin, while the links to local mixing and altered transcriptional coordination are best interpreted as a phenomenological model rather than a fully demonstrated mechanism. We have revised the text accordingly and addressed Reviewer #3’s comments point by point.

-The increased co-bursting despite small per-gene expression changes is interesting and the mixing defined in context of Hi-C changes, polymer modelling and FISH analysis is a far improved mechanistic connection than in the original submission. That being said, the intron-seqFISH results are relevant but are selective and indirect, genes

are chosen with minimal per-gene changes and then enhanced co-bursting is shown. This data supports the weakened insulation but does not address the concern that mixing is the primary driver versus the changes being a correlated consequence of cohesin loss. So, the result supports altered coordination but doesn't directly support a functional consequence of domain mixing. The jump from increased chromatin motion to domain mixing, to transcription insulation is still not fully convincing.

We appreciate Reviewer #3's critical comments, which helped improve our manuscript. We realized that the description of the intron-seqFISH analysis in the manuscript was not sufficiently clear. We defined co-bursting not simply as a temporal co-occurrence, but as a spatiotemporal co-occurrence (Fig. 8e and Methods). The increase in ΔFco upon cohesin depletion (Figs. 8i,j) is therefore not merely an indirect transcriptional readout, since it explicitly incorporates spatial proximity information. Thus, it provides a more direct link between local intermingling and altered transcriptional coupling than a purely indirect transcriptional readout. However, we agree that this important nuance was not sufficiently explained in the main text. We have therefore revised the text as follows:

Page 16.
"Here, co-bursting was defined only when at least one A spot and one B spot were detected in the same nucleus and the minimum 3D distance between the A-B spot pair was ≤ 1 μm, thereby focusing the analysis on spatially proximal co-bursting events (see Methods)."

"Notably, the proximal co-burst frequency of gene pairs significantly increased upon cohesin depletion (Fig. 8i,j), indicating increased cis burst coupling among spatially proximal gene pairs, consistent with a previous study in mouse ES cells [19]. Thus, cohesin depletion increases burst coupling between spatially proximal genes even when per-gene bursting remains largely unchanged, consistent with weakened local transcriptional insulation (Fig. 8k)."

In addition, we also realized that we needed to clarify the significance and purpose of the 2-point MSD analysis (Fig. 7i), which measures the relative motion between neighboring nucleosomes (<150 nm). It provides not only the magnitude of local nucleosome motion but also the extent of local intermingling between nearby nucleosomes. We therefore consider this assay to provide a more direct physical readout of local mixing than conventional one-point nucleosome motion alone. We have clarified this point in the text:

Page 14.
"It should also be noted that two-point MSD provides not only the magnitude of local nucleosome motion but also the extent of local intermingling between nearby nucleosomes."

"These results indicate that cohesin depletion increases nucleosome mobility within condensed euchromatic domains and local intermingling between nearby nucleosomes."

-Regarding the head-tethering experiment. The authors decide to tone down their claims and clarify the comparison to the WAPL deletion. This is correct but the mechanism is not resolved. The experiment does not resolve extrusion vs. other cohesin-dependent constraints which was the concern. The sororin result rules out the

role for sister chromatic cohesion, but the head tethering does not isolate extrusion, so the data doesn't justify the claim that extrusion is necessary. The conceptual implication seems to extend beyond what the experiments show.

We appreciate Reviewer #3's careful comment. We agree that the head-tethering experiment does not resolve active loop extrusion versus other cohesin-dependent constraints. Our intention was not to claim that active loop extrusion itself is necessary. Rather, together with the Sororin result, these data support the conclusion that cohesin constrains chromatin mainly through loop formation rather than sister chromatid cohesion. To avoid extending the mechanistic implication beyond what the experiment directly shows, we have revised the text and added the following statement to the "Limitations of the study" section:

Page 20.

"Our data support the idea that cohesin physically constrains condensed euchromatic domains through loop formation. However, the precise physical mechanism by which cohesin constrains chromatin remains unclear and will require further investigation."

- Statistics across replicates are still not shown in the revised manuscript for any figures. The RC and AC changes do not seem meaningful given the errors. It is unclear how significance is being or what the cell to cell or replicate to replicate variability is. The relatively changes in all SMT derived parameters are still hard to assess as being significant, there is no discussion of why this is a meaningful change it is still just reported as large.

We thank Reviewer #3 for pointing out this important issue. We agree that the statistical presentation of the SMT-derived metrics was not sufficiently clear in the manuscript. In the revised manuscript, all SMT-derived metrics are presented in terms of cell-to-cell variability, and statistical analyses across biological replicates have now been added. Statistical significance has been added to the relevant figures and legends, and full details of the AC analyses are now provided in Supplementary Table 1. The changes in *Rc*, AC, and the other SMT-derived metrics remain consistent with the MSD-based results, and we have revised the text to clarify their interpretation.

Supplementary Table 1:

Fig	cell	condition_1	condition_2	n1 (cells)	n2 (cells)	mean1	mean2	sd1	sd2	welch_t_p_holm
Fig. 1h	RAD21-cAC	H2B_DMSO	H2B_IAA	30	30	-1.22153	-1.09925	0.078177045	0.081720655	3.68E-07
Fig. 3c	WAPL-mAC	H2B_DMSO	H2B_IAA	30	30	-1.22377	-1.38676	0.063908195	0.06292528	1.48E-13
Fig. 3f	CTCF-mAC	H2B_DMSO	H2B_IAA	21	30	-1.21931	-1.21338	0.058835482	0.08782702	1
Fig. 3i	Head-tethering	AP	AP_IAA	30	20	-1.1333	-0.98601	0.070463842	0.074156976	2.48E-05
Fig. 3i	Head-tethering	AP	EtOH	30	20	-1.1333	-1.11385	0.070463842	0.070373394	1
Fig. 3i	Head-tethering	AP_IAA	EtOH	20	20	-0.98601	-1.11385	0.074156976	0.070373394	0.000157301
Extened Data Fig. 3e	HCT116 (siRNA treated)	H2B_siControl	H2B_siWAPL	15	15	-1.20723	-1.31518	0.051677838	0.06616328	0.001059098
Extened Data Fig. 3g	HCT116 (siRNA treated)	H2B_siControl	H2B_siCTCF	15	15	-1.20723	-1.25589	0.043140992	0.051677838	0.130252903

-The authors use genomics to show that H3.3 is mostly present in euchromatin. However, the recommendation was to perform imaging which is more relevant to the rest of the single cell analysis in the paper. I still recommend an imaging control as suggested to show the relative exclusion from heterochromatin regions by imaging. I

think such an experiment showing the extent of spatial colocalization with heterochromatin markers would make it clear what confounding effects there may be in the analysis presented.

We are grateful for this suggestion. In response, we added immunofluorescence images of representative heterochromatin marks (H3K27me3 and H3K9me3) together with H3.3-Halo as Extended Data Fig. 5b. These data show that H3.3 exhibits anticorrelated spatial patterns relative to heterochromatin markers, which we further quantified by negative Pearson's correlation values. We believe this imaging control directly addresses the reviewer's concern by showing that H3.3 is relatively excluded from heterochromatin regions, thereby clarifying the extent of potential confounding in the single-cell imaging analyses. We also added the following to the main text:

Page 8.
"Expressed H3.3-Halo was properly incorporated into nucleosomes (Extended Data Fig. 5a), localized to DAPI-poor nuclear regions (Fig. 4a) and to regions largely complementary to repressive histone marks (Extended Data Fig. 5b). "

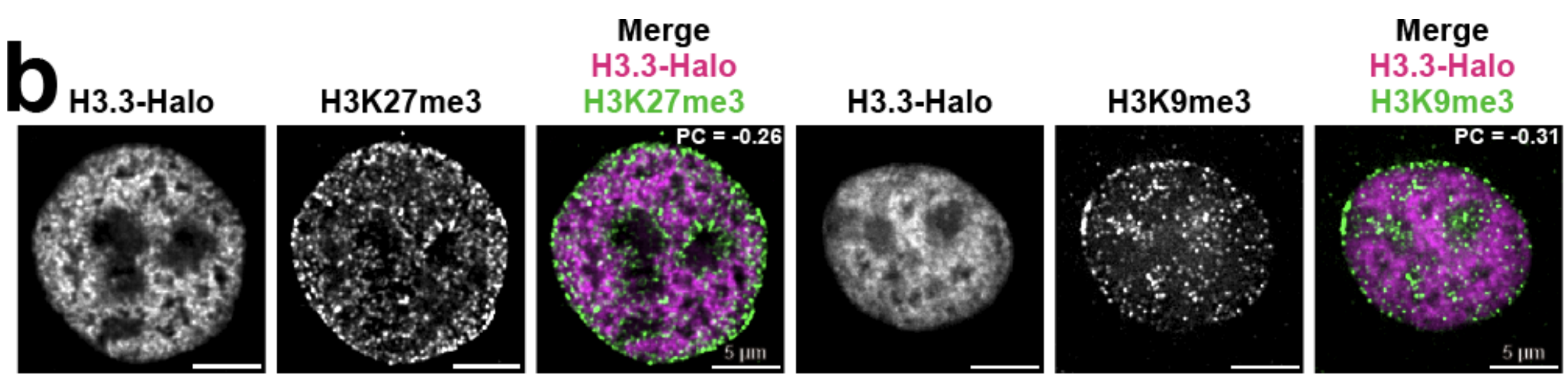

One other concern is that the Hi-C and ATAC evidence that is presented is integrated from prior datasets rather than in the same pipeline as is applied in this study, the limitations of this cross analysis and potential caveats are not discussed.

We thank Reviewer #3 for this important point. We agree that the Hi-C and ChIP-seq evidence discussed here was integrated from previously published datasets (Rao et al. Cell 2017) rather than generated within the same experimental pipeline as our imaging analyses. By contrast, the ATAC-seq data with and without RAD21 depletion were generated in the present study and analyzed within our current experimental framework. To make this point and its limitations explicit, we have now added the following sentence to the "Limitations of the study" section:

Page 20.
"Finally, the Hi-C and ChIP-seq evidence discussed here was integrated from previously published datasets [87,101] rather than generated within the same experimental pipeline as our imaging analyses. Therefore, potential differences in experimental conditions and data processing should be considered, and these cross-platform comparisons are interpreted here as orthogonal support."

We thank Reviewer #3 for the thoughtful comments and the Editor for the helpful guidance. Following these comments and suggestions, we have revised the manuscript accordingly and believe that it has been substantially strengthened. Our point-by-point responses are provided below.

Reviewers' Comments:
Reviewer #3 (Remarks to the Author):
I appreciate the authors' efforts to add disclaimers and limitations statements that make clear that a direct mechanism in support of the proposed model is not established in this manuscript.

We appreciate Reviewer #3's understanding and appreciation for our previous revised manuscript.

I remain concerned, however, about how the statistics are reported, as the newly supplied table still does not show replicate-level variability. What is explicitly missing is statistical analysis across biological replicates rather than statistics calculated across pooled cells.

We fully agree that statistical analysis across biological replicates was missing in the previous manuscript, although we had carefully confirmed the reproducibility of all experiments throughout the study.

Was each experiment performed only once, with 30 cells measured from that single experiment? If not, the relevant metrics should be calculated for each independent experiment and the statistics should be performed across those experiments. If only one experiment was performed, then it should be repeated, as cells collected within the same experiment cannot be treated as independent biological replicates.

We performed all experiments independently at least twice, and usually three times. However, we agree that most of the MSD plots presented in the previous manuscript were based on 30 cells from 1 or 2 experiments and did not include statistical analyses across biological replicates, although sufficient biological replicates were available.

This issue is especially important given the statistical tests presented in Supplementary Table 1 and the relatively small differences being reported. For the two-sided K-S test, many cells are pooled, and the test is highly sensitive to small shifts in the distribution. It therefore does not address the replicate-level issue. At most, it shows that the pooled distributions differ, but not whether the effect is robust across independent biological replicates.

Similarly, the Welch's t-test appears to have been performed at the cell level rather than at the biological replicate level. Cells from the same experiment are not independent biological replicates. As a result, the reported p values may appear much stronger than they would if replicate-to-replicate variability were properly taken into account.

We agree that our previous presentation showed differences in the pooled distributions, but did not establish whether these effects were robust across independent biological replicates. At the same time, we consider the pooled cell-level distributions to be informative, as they capture the substantial cell-to-cell variability that is an important feature of single-nucleosome motion. In the revised manuscript, we therefore retain the cell-level distributions to show this heterogeneity, while now providing statistical inference across biological replicates.

Regarding statistical testing, in the previous manuscript both the two-sided Kolmogorov-Smirnov test (MSD values at 0.5 s) and Welch's *t*-test (Rc and AC values) were applied to pooled cell-level data and therefore did not address the replicate-level issue. In the revised manuscript, we retain the two-sided Kolmogorov-Smirnov test to assess differences in pooled cell-level distributions of MSD values, but now additionally perform replicate-aware statistical analyses across biological replicates using paired two-sided t-tests.

A replicate-aware statistical analysis should be provided for Rc, AC, and all other SMT-derived metrics across all experimental conditions.

We fully agree with Reviewer #3's suggestion. To provide biological replicate-aware statistics in full, we repeated experiments where needed (Figs. 2c, 2f, 2g, 3c, 4e, and Extended Data Figs. 1g, 7c, and 7d) and reanalyzed the SMT-derived metrics using biological replicates as the statistical unit.

We now provide biological replicate-aware statistical analyses for the SMT-derived metrics. In particular, MSD at 0.5 s and two-point MSD at 0.5 s, which are the most important metrics supporting the conclusions of this manuscript, have been added as new right-side panels in the corresponding main figures.

Notably, the replicate-aware statistical analyses of MSD values at 0.5 s and two-point MSD values at 0.5 s in the main figures all supported the conclusions of the manuscript.

Other supportive metrics that are directly relevant to the main conclusions of the manuscript, including Rc and AC values mentioned in the main text and presented in the main figures, are reported in the updated Supplementary Table 1 together with the corresponding raw replicate-level values, statistical tests, p values, and related details.

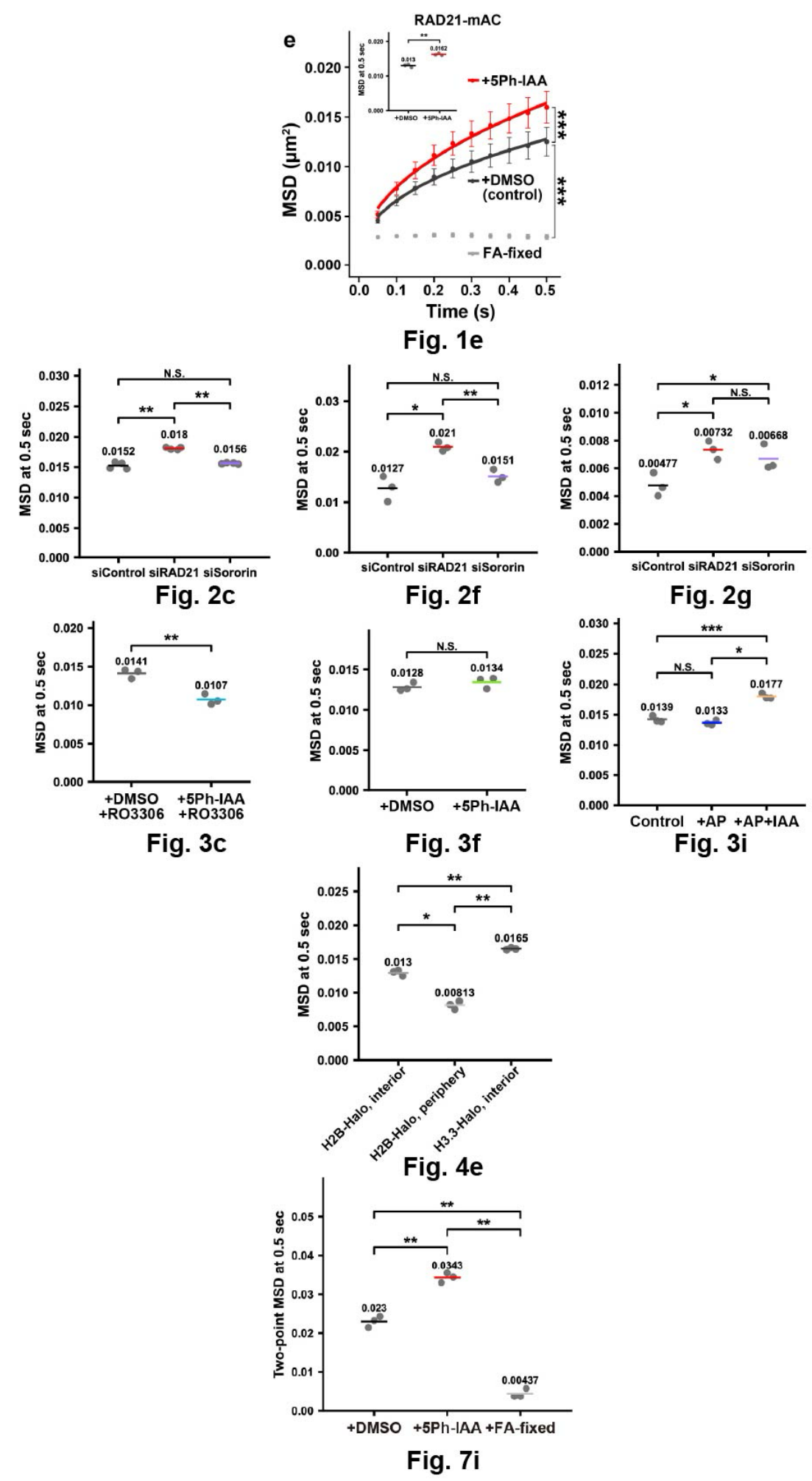

Main Figures

Results section was accordingly modified:
"Cohesin depletion via 5Ph-IAA treatment for 1 hour (Fig. 1f; Extended Data Fig. 1a) significantly increased motion across biological replicates…"

"Sororin knockdown (Extended Data Fig. 2b) significantly increased EGFP-LacI mobility across biological replicates but not TetR-4×mCherry (Fig. 2f,g)…"

"and increased RAD21 chromatin binding (Extended Data Fig. 3b), which led to significantly decreased MSD and AC values across biological replicates (Fig. 3c; Supplementary Table 1)."

"Single-nucleosome imaging in HCT116 cells showed that H3.3-Halo nucleosomes were significantly more mobile than H2B-Halo ones across biological replicates (Fig. 4e)..."

"Notably, two-point MSD significantly increased upon acute cohesin depletion (1 h 5Ph-IAA) across biological replicates (Fig. 7i, Extended Data Fig. 9j-l)."

MSD at 0.5 s and two-point MSD at 0.5 s in the Extended Data figures are also shown on the right.

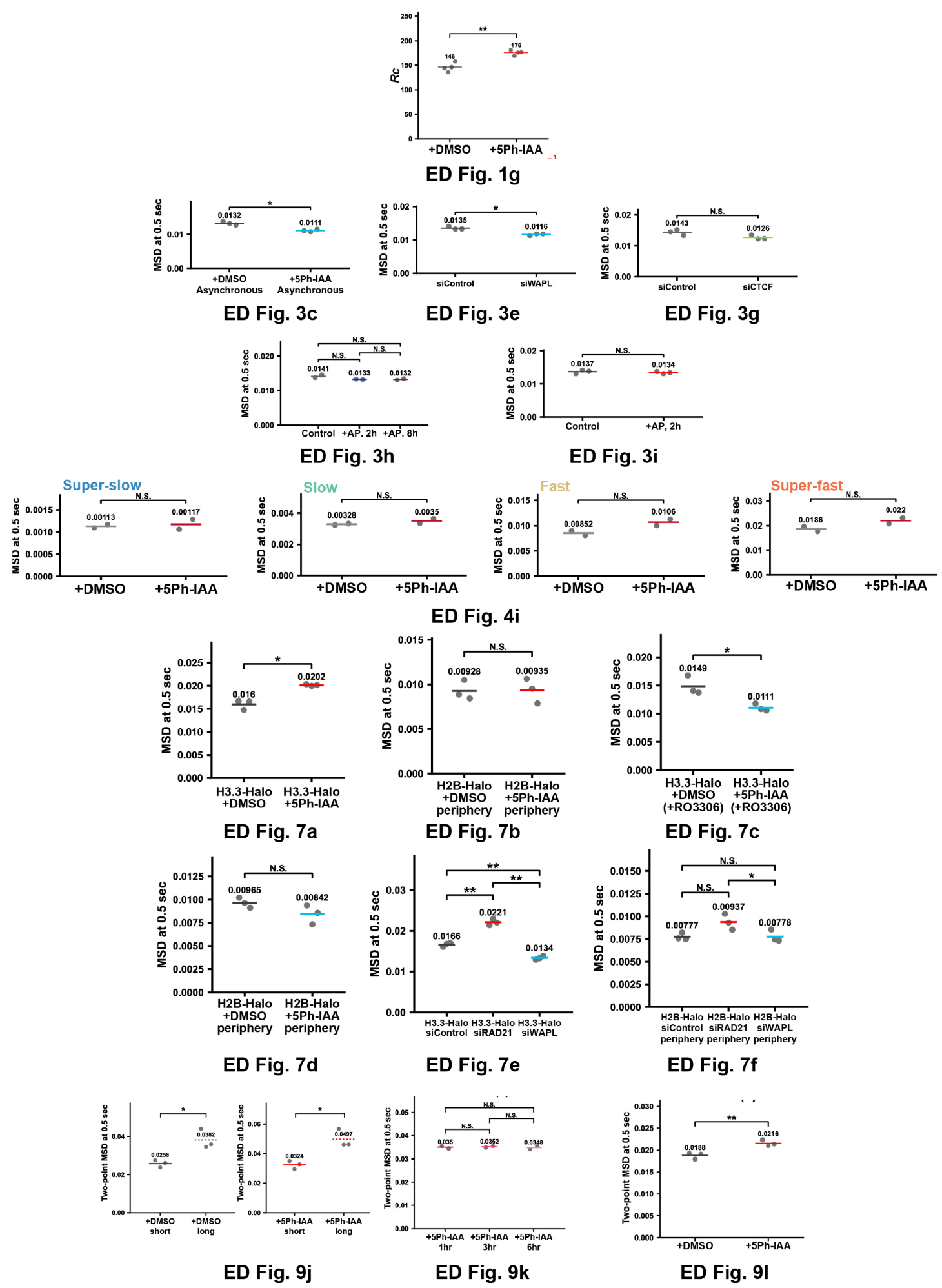

ED Figures

Notably, all results of statistical analyses across biological replicates for MSD values at 0.5 s and two-point MSD values at 0.5 s in the Extended Data figures reinforced the conclusions in the manuscript.

An exception is the RL analysis in Extended Data Fig. 4i. These data suggested a consistent trend toward increased MSD in the “Super-fast” and “Fast” subpopulations, but this did not reach statistical significance.

We therefore noted this point in the text, simplified the description, and weakened the claim as follows:

“In this single-cell analysis, cohesin depletion was associated with a rightward shift of these populations (Extended Data Fig. 4g,i), implying that cohesin might constrain movements of nucleosomes mainly in euchromatin.”

In the legend, we added:
“Note that reproducible trends toward increases were observed in the ‘Super-fast’ and ‘Fast’ fractions, but these differences were not statistically significant. Statistical details are provided in Supplementary Table 1.”

At the same time, as mentioned above, we retain the cell-level distributional analyses because cell-to-cell variability is also informative for SMT measurements.

Updated Supplementary Table 1:

Sheet	Metric	Condition_1	Condition_2	n_rep_condition_1	n_rep_condition_2	Mean_condition_1	Mean_condition_2	SD_condition_1	SD_condition_2	Test	p_value	p_value_Holm
Fig. 1e, g, h	MSD at 0.5 sec	DMSO	5Ph-IAA	3	3	0.01297	0.016216667	0.000410731	0.000340343	Paired two-sided t-test on biological replicate values	0.001594196	0.001594196
Fig. 1e, g, h	Exponent	DMSO	5Ph-IAA	3	3	0.427333333	0.478333333	0.020132892	0.019553346	Paired two-sided t-test on biological replicate values	0.000128131	0.000128131
Fig. 1e, g, h	AC value	DMSO	5Ph-IAA	3	3	-1.256666667	-1.125666667	0.097520938	0.085189984	Paired two-sided t-test on biological replicate values	0.003305027	0.003305027
Fig. 1e, g, h	Dsub	DMSO	5Ph-IAA	3	3	0.017666667	0.023	0.00057735	4.24919E-18	Paired two-sided t-test on biological replicate values	0.00388351	0.00388351
Fig. 2c	MSD at 0.5 sec	siControl	siRAD21	4	4	0.01523	0.01804	0.000557674	0.000205751	Paired two-sided t-test on biological replicate values	0.004985383	0.009970767
Fig. 2c	MSD at 0.5 sec	siControl	siSororin	4	4	0.01523	0.0155875	0.000557674	0.000118708	Paired two-sided t-test on biological replicate values	0.203059452	0.203059452
Fig. 2c	MSD at 0.5 sec	siRAD21	siSororin	4	4	0.01804	0.0155875	0.000205751	0.000118708	Paired two-sided t-test on biological replicate values	0.000575539	0.001726616
Fig. 2c	Exponent	siControl	siRAD21	4	4	0.47825	0.51475	0.009742518	0.0105	Paired two-sided t-test on biological replicate values	0.021594996	0.043189992
Fig. 2c	Exponent	siControl	siSororin	4	4	0.47825	0.4935	0.009742518	0.006244998	Paired two-sided t-test on biological replicate values	0.0547059	0.0547059
Fig. 2c	Exponent	siRAD21	siSororin	4	4	0.51475	0.4935	0.0105	0.006244998	Paired two-sided t-test on biological replicate values	0.007599581	0.022798744
Fig. 2c	Dsub	siControl	siRAD21	4	4	0.0225	0.02675	0.001	0.0005	Paired two-sided t-test on biological replicate values	0.006627177	0.013254355
Fig. 2c	Dsub	siControl	siSororin	4	4	0.0225	0.02275	0.001	0.0005	Paired two-sided t-test on biological replicate values	0.717685644	0.717685644
Fig. 2c	Dsub	siRAD21	siSororin	4	4	0.02675	0.02275	0.0005	0.0005	Paired two-sided t-test on biological replicate values	0.002259504	0.006778511
Fig. 2f	MSD at 0.5 sec	siControl	siRAD21	3	3	0.012746667	0.020953333	0.002501226	0.00090257	Paired two-sided t-test on biological replicate values	0.013146947	0.026293894
Fig. 2f	MSD at 0.5 sec	siControl	siSororin	3	3	0.012746667	0.015106667	0.002501226	0.001271626	Paired two-sided t-test on biological replicate values	0.089370802	0.089370802
Fig. 2f	MSD at 0.5 sec	siRAD21	siSororin	3	3	0.020953333	0.015106667	0.00090257	0.001271626	Paired two-sided t-test on biological replicate values	0.001325809	0.003977428
Fig. 2g	MSD at 0.5 sec	siControl	siRAD21	3	3	0.004773333	0.007316667	0.000840555	0.000670622	Paired two-sided t-test on biological replicate values	0.024032508	0.048065016
Fig. 2g	MSD at 0.5 sec	siControl	siSororin	3	3	0.004773333	0.006676667	0.000840555	0.000939805	Paired two-sided t-test on biological replicate values	0.007589661	0.022768984
Fig. 2g	MSD at 0.5 sec	siRAD21	siSororin	3	3	0.007316667	0.006676667	0.000670622	0.000939805	Paired two-sided t-test on biological replicate values	0.185137518	0.185137518
Fig. 3c	MSD at 0.5 sec	RO+DMSO	RO+5Ph-IAA	3	3	0.014123333	0.010716667	0.00060575	0.000675599	Paired two-sided t-test on biological replicate values	0.004304331	0.004304331
Fig. 3c	Exponent	RO+DMSO	RO+5Ph-IAA	3	3	0.472333333	0.399	0.029143324	0.033286634	Paired two-sided t-test on biological replicate values	0.001072652	0.001072652
Fig. 3c	AC value	RO+DMSO	RO+5Ph-IAA	3	3	-1.081	-1.244	0.123660826	0.123559702	Paired two-sided t-test on biological replicate values	1.25457E-05	1.25457E-05
Fig. 3c	Dsub	RO+DMSO	RO+5Ph-IAA	3	3	0.0203	0.014566667	0.000793725	0.000321455	Paired two-sided t-test on biological replicate values	0.003463534	0.003463534
Fig. 3f	MSD at 0.5 sec	DMSO	5Ph-IAA	3	3	0.012823333	0.01343	0.000523673	0.000712671	Paired two-sided t-test on biological replicate values	0.261771105	0.261771105
Fig. 3f	Exponent	DMSO	5Ph-IAA	3	3	0.43	0.446666667	0.01	0.005773503	Paired two-sided t-test on biological replicate values	0.12961172	0.12961172
Fig. 3f	AC value	DMSO	5Ph-IAA	3	3	-1.361666667	-1.354	0.123589374	0.122405065	Paired two-sided t-test on biological replicate values	0.144620796	0.144620796
Fig. 3f	Dsub	DMSO	5Ph-IAA	3	3	0.017666667	0.018666667	0.001154701	0.001527525	Paired two-sided t-test on biological replicate values	0.225403331	0.225403331
Fig. 3i	MSD at 0.5 sec	EtOH(Control)	AP_2h	3	3	0.013913333	0.013343333	0.000528804	0.000397157	Paired two-sided t-test on biological replicate values	0.331842611	0.331842611
Fig. 3i	MSD at 0.5 sec	EtOH(Control)	IAA_AP	3	3	0.013913333	0.01769	0.000528804	0.00044238	Paired two-sided t-test on biological replicate values	0.000192359	0.000577076
Fig. 3i	MSD at 0.5 sec	AP_2h	IAA_AP	3	3	0.013343333	0.01769	0.000397157	0.00044238	Paired two-sided t-test on biological replicate values	0.008266657	0.016533313
Fig. 3i	Exponent	EtOH(Control)	AP_2h	3	3	0.473	0.466	0.013	0.009643651	Paired two-sided t-test on biological replicate values	0.093783111	0.093783111
Fig. 3i	Exponent	EtOH(Control)	IAA_AP	3	3	0.473	0.532666667	0.013	0.007637626	Paired two-sided t-test on biological replicate values	0.003477299	0.006954599
Fig. 3i	Exponent	AP_2h	IAA_AP	3	3	0.466	0.532666667	0.009643651	0.007637626	Paired two-sided t-test on biological replicate values	0.00159617	0.004788511
Fig. 3i	AC value	EtOH(Control)	AP_2h	3	3	-1.082666667	-1.102666667	0.04336281	0.032715949	Paired two-sided t-test on biological replicate values	0.147197135	0.147197135
Fig. 3i	AC value	EtOH(Control)	IAA_AP	3	3	-1.082666667	-0.953333333	0.04336281	0.042712215	Paired two-sided t-test on biological replicate values	0.000205856	0.000617569
Fig. 3i	AC value	AP_2h	IAA_AP	3	3	-1.102666667	-0.953333333	0.032715949	0.042712215	Paired two-sided t-test on biological replicate values	0.002387983	0.004775966
Fig. 3i	Dsub	EtOH(Control)	AP_2h	3	3	0.02	0.019	0.001	0	Paired two-sided t-test on biological replicate values	0.225403331	0.225403331
Fig. 3i	Dsub	EtOH(Control)	IAA_AP	3	3	0.02	0.026	0.001	0.001	Paired two-sided t-test on biological replicate values	3.83124E-32	1.14937E-31
Fig. 3i	Dsub	AP_2h	IAA_AP	3	3	0.019	0.026	0	0.001	Paired two-sided t-test on biological replicate values	0.006734083	0.013468167
Fig. 4e	MSD at 0.5 sec	H2B_interior	H2B_periphery	3	3	0.012966667	0.008133333	0.000416333	0.000602771	Paired two-sided t-test on biological replicate values	0.014016012	0.014016012
Fig. 4e	MSD at 0.5 sec	H2B_interior	H3.3_interior	3	3	0.012966667	0.016533333	0.000416333	0.000152753	Paired two-sided t-test on biological replicate values	0.003734759	0.007469518
Fig. 4e	MSD at 0.5 sec	H2B_periphery	H3.3_interior	3	3	0.008133333	0.016533333	0.000602771	0.000152753	Paired two-sided t-test on biological replicate values	0.002447523	0.00734257
Fig. 4e	Exponent	H2B_interior	H2B_periphery	3	3	0.425333333	0.44	0.01747379	0.02	Paired two-sided t-test on biological replicate values	0.232661883	0.268675286
Fig. 4e	Exponent	H2B_interior	H3.3_interior	3	3	0.425333333	0.504333333	0.01747379	0.027135463	Paired two-sided t-test on biological replicate values	0.061229679	0.183689038
Fig. 4e	Exponent	H2B_periphery	H3.3_interior	3	3	0.44	0.504333333	0.02	0.027135463	Paired two-sided t-test on biological replicate values	0.134337643	0.268675286
Fig. 4e	Dsub	H2B_interior	H2B_periphery	3	3	0.017666667	0.011	0.00057735	0.001	Paired two-sided t-test on biological replicate values	0.017053626	0.017053626
Fig. 4e	Dsub	H2B_interior	H3.3_interior	3	3	0.017666667	0.024666667	0.00057735	0.000585947	Paired two-sided t-test on biological replicate values	6.80203E-05	0.000204061
Fig. 4e	Dsub	H2B_periphery	H3.3_interior	3	3	0.011	0.024666667	0.001	0.000585947	Paired two-sided t-test on biological replicate values	0.004332184	0.008664369
Fig. 7i	Two-point MSD at 0.5 sec	DMSO	5Ph-IAA	3	3	0.022973333	0.034323333	0.001409976	0.00125906	Paired two-sided t-test on biological replicate values	0.002971687	0.002971687
Fig. 7i	Two-point MSD at 0.5 sec	DMSO	FA	3	3	0.022973333	0.004373333	0.001409976	0.001148927	Paired two-sided t-test on biological replicate values	0.000738669	0.002216007
Fig. 7i	Two-point MSD at 0.5 sec	5Ph-IAA	FA	3	3	0.034323333	0.004373333	0.00125906	0.001148927	Paired two-sided t-test on biological replicate values	0.000991868	0.002216007
ED Fig. 1g	Rc	DMSO	5Ph-IAA	4	4	146	175.75	9.092121131	4.991659711	Paired two-sided t-test on biological replicate values	0.004538373	0.004538373
ED Fig. 3c	MSD at 0.5 sec	DMSO	5Ph-IAA	3	3	0.013236667	0.01112	0.000515978	0.000361663	Paired two-sided t-test on biological replicate values	0.041505885	0.041505885
ED Fig. 3e	MSD at 0.5 sec	siControl	siWAPL	3	3	0.01353	0.011623333	0.000477598	0.000288848	Paired two-sided t-test on biological replicate values	0.049742109	0.049742109
ED Fig. 3g	MSD at 0.5 sec	siControl	siCTCF	3	3	0.014323333	0.01265	0.000957305	0.000693109	Paired two-sided t-test on biological replicate values	0.119923747	0.119923747
ED Fig. 3h	MSD at 0.5 sec	EtOH(Control)	AP_2h	2	2	0.01414	0.01327	0.000537401	8.48528E-05	Paired two-sided t-test on biological replicate values	0.298087489	1
ED Fig. 3h	MSD at 0.5 sec	EtOH(Control)	AP_8h	2	2	0.01414	0.01324	0.000537401	0.000268701	Paired two-sided t-test on biological replicate values	0.13245265	0.662263252
ED Fig. 3h	MSD at 0.5 sec	AP_2h	AP_8h	2	2	0.01327	0.01324	8.48528E-05	0.000268701	Paired two-sided t-test on biological replicate values	0.923969184	1
ED Fig. 3i	MSD at 0.5 sec	EtOH(Control)	AP_2h	3	3	0.01367	0.013393333	0.000576888	0.000340196	Paired two-sided t-test on biological replicate values	0.651865361	0.651865361
ED Fig.4i_ss	MSD at 0.5 sec	DMSO_superslow	5Ph-IAA_superslow	2	2	0.001130674	0.001172062	5.27784E-05	0.000158709	Paired two-sided t-test on biological replicate values	0.678637916	0.678637916
ED Fig.4i_s	MSD at 0.5 sec	DMSO_slow	5Ph-IAA_slow	2	2	0.003283023	0.003499463	6.60735E-05	0.000209519	Paired two-sided t-test on biological replicate values	0.278992658	0.278992658
ED Fig.4i_f	MSD at 0.5 sec	DMSO_fast	5Ph-IAA_fast	2	2	0.008519349	0.010648823	0.000658096	0.000886574	Paired two-sided t-test on biological replicate values	0.301712618	0.301712618
ED Fig.4i_sf	MSD at 0.5 sec	DMSO_superfast	5Ph-IAA_superfast	2	2	0.018620318	0.021956884	0.001373768	0.001644752	Paired two-sided t-test on biological replicate values	0.36230261	0.36230261
ED Fig. 7a	MSD at 0.5 sec	DMSO	5Ph-IAA	3	3	0.01599	0.020156667	0.001057733	0.000185023	Paired two-sided t-test on biological replicate values	0.015059572	0.015059572
ED Fig. 7b	MSD at 0.5 sec	DMSO_peri	5Ph-IAA_peri	3	3	0.00928	0.009346667	0.001096175	0.00139012	Paired two-sided t-test on biological replicate values	0.944622341	0.944622341
ED Fig. 7c	MSD at 0.5 sec	DMSO	5Ph-IAA	3	3	0.01487	0.011063333	0.001687691	0.000657673	Paired two-sided t-test on biological replicate values	0.023732627	0.023732627
ED Fig. 7d	MSD at 0.5 sec	DMSO_peri	5Ph-IAA_peri	3	3	0.00965	0.008423333	0.000550273	0.001027878	Paired two-sided t-test on biological replicate values	0.152658351	0.152658351
ED Fig. 7e	MSD at 0.5 sec	siControl	siRAD21	3	3	0.016616667	0.022103333	0.00049339	0.000752684	Paired two-sided t-test on biological replicate values	0.003356204	0.005118119
ED Fig. 7e	MSD at 0.5 sec	siControl	siWAPL	3	3	0.016616667	0.013363333	0.00049339	0.000470567	Paired two-sided t-test on biological replicate values	0.001553205	0.004659615
ED Fig. 7e	MSD at 0.5 sec	siRAD21	siWAPL	3	3	0.022103333	0.013363333	0.000752684	0.000470567	Paired two-sided t-test on biological replicate values	0.002559059	0.005118119
ED Fig. 7f	MSD at 0.5 sec	siControl_peri	siRAD21_peri	3	3	0.007766667	0.009373333	0.000395769	0.000876717	Paired two-sided t-test on biological replicate values	0.097406764	0.194813528
ED Fig. 7f	MSD at 0.5 sec	siControl_peri	siWAPL_peri	3	3	0.007766667	0.007783333	0.000395769	0.000666658	Paired two-sided t-test on biological replicate values	0.976598437	0.976598437
ED Fig. 7f	MSD at 0.5 sec	siRAD21_peri	siWAPL_peri	3	3	0.009373333	0.007783333	0.000876717	0.000666658	Paired two-sided t-test on biological replicate values	0.015908965	0.047726894
ED Fig. 9j_DMSO	Two-point MSD at 0.5 sec	DMSO_short	DMSO_long	3	3	0.02581	0.038183333	0.001928963	0.005103649	Paired two-sided t-test on biological replicate values	0.027238237	0.027238237
ED Fig. 9j_IAA	Two-point MSD at 0.5 sec	5Ph-IAA_short	5Ph-IAA_long	3	3	0.032436667	0.04967	0.002719455	0.006105825	Paired two-sided t-test on biological replicate values	0.019159066	0.019159066
ED Fig. 9k	Two-point MSD at 0.5 sec	5Ph-IAA, 1hr	5Ph-IAA, 3hr	2	2	0.034975	0.035195	0.000770746	0.000445477	Paired two-sided t-test on biological replicate values	0.840562977	1
ED Fig. 9k	Two-point MSD at 0.5 sec	5Ph-IAA, 1hr	5Ph-IAA, 6hr	2	2	0.034975	0.03481	0.000770746	0.00086267	Paired two-sided t-test on biological replicate values	0.909665529	1
ED Fig. 9k	Two-point MSD at 0.5 sec	5Ph-IAA, 3hr	5Ph-IAA, 6hr	2	2	0.035195	0.03481	0.000445477	0.00086267	Paired two-sided t-test on biological replicate values	0.416228387	1
ED Fig. 9l	Two-point MSD at 0.5 sec	DMSO	5Ph-IAA	3	3	0.018806667	0.021576667	0.000755932	0.00070316	Paired two-sided t-test on biological replicate values	0.009722248	0.009722248

These replicate-aware analyses support the robustness of the main conclusions and strengthen the revised manuscript. We very much appreciate Reviewer #3’s suggestion.
